# Scalable Synthesis and Cancer Cell Cytotoxicity of Rooperol and Analogues

**DOI:** 10.3390/molecules27061792

**Published:** 2022-03-09

**Authors:** Zachary T. Schwartz, Peter D. Theisen, Olaf T. Bjornstal, Mary Rodebaugh, Mauricio A. Jemal, Dallas Lee, Spencer D. Shelton, Zhenze Zhao, Liqin Du, Sean M. Kerwin

**Affiliations:** 1 Department of Chemistry & Biochemistry, Texas State University, San Marcos, TX 78666, USA; zts1624@gmail.com (Z.T.S.); skerwin@austin.utexas.edu (P.D.T.); maryleerodebaugh@yahoo.com (M.R.); maujemal@txstate.edu (M.A.J.); beta68@gmail.com (D.L.); spencerd.shelton@utsouthwestern.edu (S.D.S.); zhense3zhao@yahoo.com (Z.Z.); liqindu6@txstate.edu (L.D.); 2 Division of Chemical Biology and Medicinal Chemistry, University of Texas at Austin, Austin, TX 78712, USA; otbjornstal@gmail.com; 3 Materials Science, Engineering, and Commercialization Program, Texas State University, San Marcos, TX 78666, USA

**Keywords:** catechol, polyphenol, anticancer

## Abstract

Plant polyphenols, such as the African potato (*Hypoxis hemerocallidea*)-derived bis-catechol rooperol, can display promising anticancer activity yet suffer from rapid metabolism. Embarking upon a program to systematically examine potentially more metabolically stable replacements for the catechol rings in rooperol, we report here a general, scalable synthesis of rooperol and analogues that builds on our previous synthetic approach incorporating a key Pd-catalyzed decarboxylative coupling strategy. Using this approach, we have prepared and evaluated the cancer cell cytotoxicity of rooperol and a series of analogues. While none of the analogues examined here were superior to rooperol in preventing the growth of cancer cells, analogues containing phenol or methylenedioxyphenyl replacements for one or both catechol rings were nearly as effective as rooperol.

## 1. Introduction

**Figure 1:**
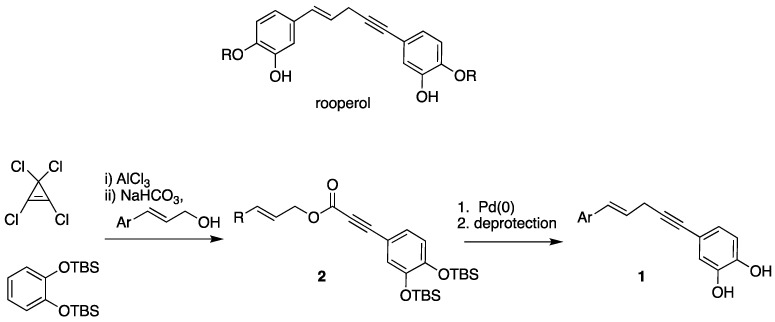
Structure of rooperol (**top**) and prior synthetic scheme for rooperol analogues **1** (**bottom**).

African potato (*Hypoxis hemerocallidea*) is a widely used medicinal plant in southern Africa [1]. Ethanolic extracts of the corms of *H. hemerocallidea* contain 10–15% of hypoxoside, a bis-glucoside of the aglycone rooperol, **1** (Figure 1) [1]. Rooperol has demonstrated a cytotoxicity against cancer cell lines [2,3,4], and an orally administered extract of *H. hemercallidea* has been the subject of Phase I clinical trial in advanced lung cancer patients [5,6]. Interestingly, in this Phase I trial, no dose-limiting toxicity was identified, and 5 of the 24 patients enrolled showed some signs of response, including one complete response (>5 years) [6]. Despite this hint of clinical promise, pharmacokinetic studies demonstrated the very rapid metabolism of hypoxoside and rooperol, with only Phase II metabolites of rooperol detected in the blood of these patients [5].

Rooperol is one of a number of plant polyphenols that have demonstrated promise as anticancer agents [7,8,9]. However, a common issue with these anticancer polyphenols is metabolic instability [10]. Our work has focused on the design and evaluation of analogues of biologically interesting plant polyphenols with the goal of improved metabolic stability [11,12]. In the present case, our efforts focused on rooperol analogues with more metabolically stable replacements for this natural product’s catechol moieties.

A number of synthetic studies of rooperol and analogues have been reported. The first total synthesis by Drewes and co-workers employed a problematic Csp-Csp3 coupling step [13]. We subsequently reported a very concise synthesis of rooperol and analogues, which incorporated two key strategies: a Friedel–Crafts reaction involving tetrachlorocyclopropene and electron-rich aromatics and a Pd-catalyzed decarboxylative coupling of the resulting 3-arylprop-2-ene-1-yl arylpropiolates **2** (Figure 1) [14]. Although this route could afford rooperol analogues in as few as three steps, it suffers from two limitations. First, the route shown in Figure 1 lacks generality in that only very electron-rich aromatic compounds participate in the initial Friedel–Crafts reaction, and only certain arylprop-2-ene-1-ols directly afford the esters **2** upon alcoholysis of the initially formed Friedel–Crafts product. Second, the Friedel–Crafts reaction did not proceed well above 1 mmole scale, limiting the amount of material that could be prepared for biological assay. Thus, in order to continue our efforts to prepare and evaluate metabolically stable rooperol analogues, we first had to redesign this synthesis. Here, we report a more general, scalable synthesis of rooperol and analogs that retain the key Pd-catalyzed decarboxylative coupling from our earlier work. Using this improved synthesis, we prepared a number of rooperol analogs and report here their activity against HeLa cancer cells.

## 2. Results

**Figure 2:**
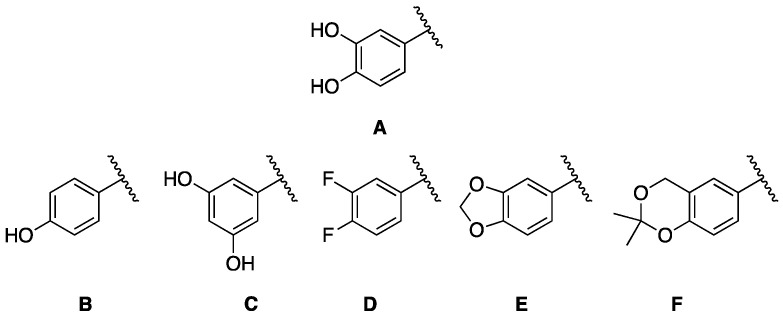
Replacements for the catechol groups (A) of rooperol.

We set out to evaluate a variety of potential replacements for the catechol moieties of rooperol. As shown in Figure 2, these replacements included variations in the number (B) and placement (C) of the catechol hydroxyl groups and substitution of these groups with fluorine (D) or methylenedioxy groups (E). In addition, we also explored the 4*H*-benzo[*d*][1,3]dioxine group (F) as a replacement for the catechol groups in rooperol.

**Scheme 1:**
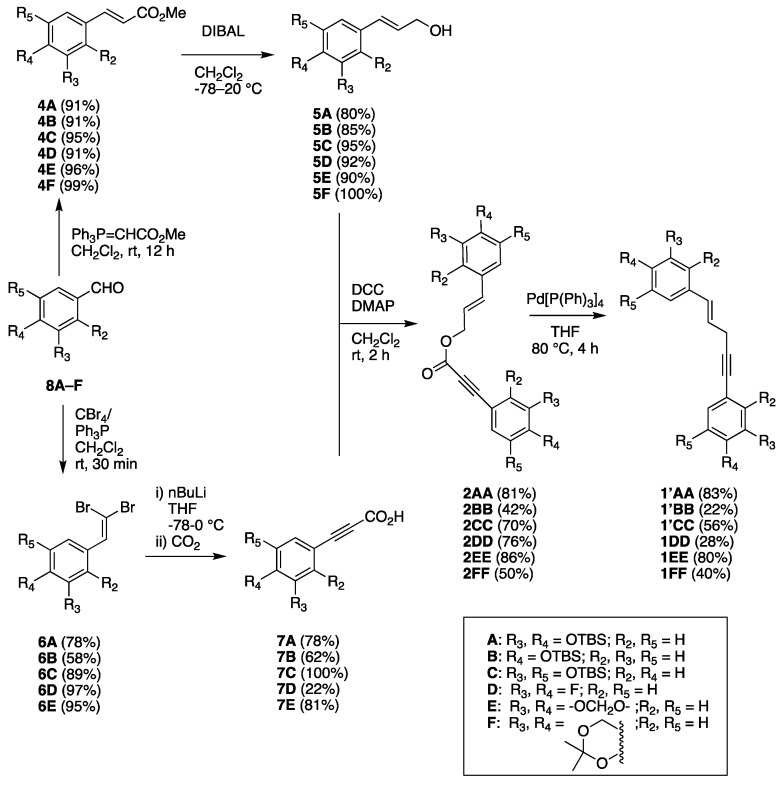
Scalable and general synthesis of protected rooperol (**1′AA**) and analogues.

Given the limitations of our previous route to rooperol and analogues (Figure 1), the preparation of the analogues contemplated in Figure 2 required an alternative synthetic scheme. Our revised synthesis of protected rooperol and analogs is shown in Scheme 1. We prepared arylprop-2-ene-1-ols **5** from the corresponding aldehydes **8** via reduction of the methyl esters **4**, obtained via Wittig reaction. Separately, the same aldehydes **8** were subjected to Corey–Fuchs alkynylation [15] via the vinyl dibromides **6**, which were subjected to elimination with nBuLi, followed by trapping of the resulting alkynyl anions with CO_2_ to afford the arylpropiolic acids **7**. All of these transformations occurred without incident to afford good yields of the products **4A–F**, **5A–F**, **6A–E**, and **7A–E** with the exception of the nBuLi elimination/trapping of the vinyl dibromide **6D** derived from 3,4-difluorobenzaldehyde. This reaction proved somewhat capricious, and at best, only modest yields of the corresponding acid **7D** were obtained.

**Scheme 2:**
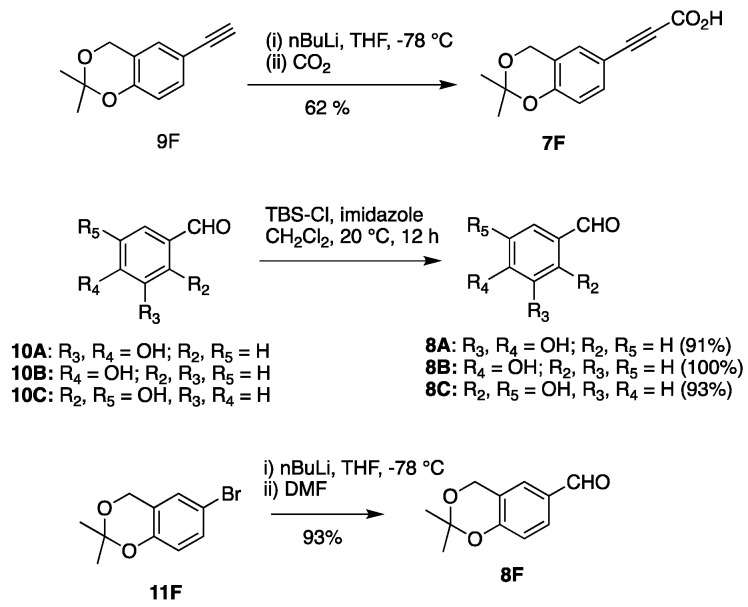
Preparation of carboxylic acid **7F**, aldehyde **8F**, and the protected aldehydes **8A, 8B**, and **8C**.

The preparation of the carboxylic acid **7F** followed a different route, as shown in Scheme 2. The previously reported alkyne **9F** [16] was deprotonated with nBuLi, and the resulting anion was trapped with CO_2_ to afford **7F** in good yield. While the aldehydes **8D** and **8E** are commercially available, the *tert*-butyldimethylsilyl-protected aldehydes **8A–C** were prepared by reacting the commercially available hydroxy-substituted benzaldehydes **10A–C** with *tert*-butyldimethylsilyl chloride in the presence of imidazole (Scheme 2). The previously reported aldehyde **8F** was prepared from the corresponding bromide **11F** following literature precedent [16] (Scheme 2).

We prepared a number of symmetrical esters **2** in which the catechol ring or replacement was the same on both the alcohol and acid moieties via DCC coupling of the acids **7** and alcohols **5** (Scheme 1). Each of these esters was then subjected to Pd-catalyzed decarboxylative coupling to afford protected rooperol **1′AA** and the protected symmetrical rooperol derivatives **1′BB** and **1′CC** as well as the rooperol analogues **1DD**, **1EE**, and **1FF** (Scheme 1).

**Scheme 3:**
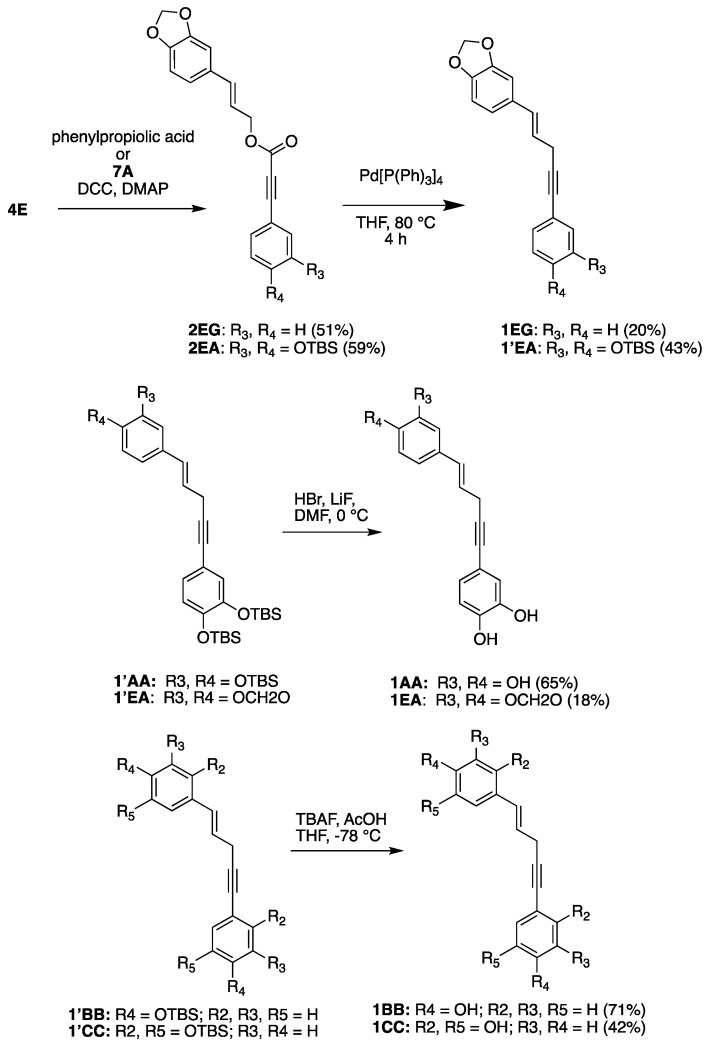
Synthesis of unsymmetrical rooperol analogs and deprotection reactions.

In addition to these symmetrical rooperol analogues, two analogues with two different replacements for the catechol moieties were also prepared (Scheme 3). DCC coupling of the alcohol **4E** with phenylpropiolic acid afforded the ester **2EG**, while coupling with the acid **7A** afforded the ester **2EA**. Each of these was subjected to Pd-catalyzed decarboxylative coupling to afford the rooperol analog **1EG** and the protected rooperol analog **1′EA** (Scheme 3).

The protected rooperol analogues **1′** were deprotected by two different methods. For the analogs containing a catechol group, we used our previously described silyl deprotection method [14] using HBr and KF, which afforded rooperol (**1AA**) and analogue **1EA** (Scheme 3). The other analogues were prepared by AcOH-buffered TBAF deprotection to afford **1BB** and **1CC** (Scheme 3).

With these rooperol analogues in hand, we evaluated the utility of the various catechol replacements by determining the cancer cell cytotoxicity of these compounds versus rooperol using a MTT assay (Table 1). Rooperol displays cytotoxicity against all three cell lines examined: HeLa (cervical adenocarcinoma), H460 (lung carcinoma), and A549 (lung carcinoma). All of the rooperol analogues examined here were also tested against HeLa cells, which were slightly more sensitive to rooperol compared to the other cell lines. All of the analogues, with the exception of **1DD**, show some activity against HeLa cells, with analogues **1CC** and **1EA** displaying activity close to that of rooperol. Interestingly, while the symmetrical analogue **1EE** lacks good activity against HeLa cells, the two asymmetrical analogues containing the same methylenedioxyphenyl catechol replacement, **1EA** and **1EB**, show better cytotoxicity. It is also interesting that **1EE** has nearly the same activity as rooperol against A549 cells, while **1DD** is much less active against both A549 and H460 cells compared to rooperol.

## 3. Discussion

A scalable and general synthesis of rooperol and analogues has been developed and used to evaluate the cancer cell cytotoxicity of a number of analogues that may have improved metabolic stability compared to rooperol. The synthesis of rooperol presented here is longer (seven total steps, longest linear sequence of five steps) but higher yielding (27% vs. 17%) compared to our previous total synthesis [14]. In addition to preparing rooperol, this route was employed to prepare a series of symmetrical and unsymmetrical analogues that were evaluated for cancer cell cytotoxicity compared to rooperol. The symmetrical compound **1BB**, bearing 4-hydroxyl substituents on each aromatic ring, displays activity against HeLa cells that is approximately one-half that of rooperol (GI_50_ = 33.2 vs. 18 µM) and similar to an unsymmetrical analog bearing a methylenedioxyphenyl group (**1EA**) in place of one catechol group of rooperol. Interestingly, while the symmetrical analogue bearing two methylenedioxyphenyl groups, **1EE**, is much less active against HeLa cells compared to rooperol, **1EE** is very similar to rooperol in activity against A459 cells (GI_50_ = 28.4 vs. 26.0 µM). Notably, because **1EE** lacks the catechol moieties of rooperol, it is not as prone to redox cycling and therefore chemically more stable than rooperol. Together, these results indicate that a more extensive search for symmetrical and asymmetrical rooperol analogues is warranted. In addition, the results obtained here indicate that the phenol and methylenedioxyphenyl catechol replacements are promising, the latter particularly against lung cancer cells. Work establishing the metabolic stability of these analogues versus rooperol is on-going.

## 4. Materials and Methods

All reactions were carried out under argon in oven-dried glassware with magnetic stirring. Unless otherwise noted, all materials were obtained from commercial suppliers and were used without further purification. THF was distilled from sodium/benzophenone prior to use. Flash chromatography was performed with EM Reagent silica gel (230–400 mesh) using the mobile phase indicated. Melting points (open capillary) are uncorrected. Unless otherwise noted, ^1^H and ^13^C NMR spectra were determined in CDCl_3_ on a spectrometer operating at 400 and 100 MHz, respectively, and are reported in ppm using solvent as internal standard (7.26 ppm for ^1^H and 77.0 ppm for ^13^C in CDCl_3_). Mass spectra were obtained by atmospheric pressure chemical ionization (APCI), chemical ionization using methane as the ionizing gas (CI), or by electrospray ionization (ESI). Copies of all NMR and MS spectra are available in the Supporting Information.

**General Procedure-Silyl Protection of Benzaldehydes: 1,2-bis-**((***tert*-butyldimethyl-silyl**)**oxy**)**benzaldehyde** (**8A**) [17]: To solution of 3,4-dihydroxybenzaldehyde (4 g, 29 mmol) in dichloromethane (120 mL) under argon was added imidazole (7.9 g, 4 equivalent). The reaction mixture was cooled in an ice bath and tert-butyldimethyl-silyl chloride (12 g, 2.5 equivalent) was added. The reaction mixture was allowed to stir overnight, then diluted with dichloromethane and washed twice with 1 N HCl, once with saturated aqueous NaHCO_3_, once with brine, and then, the organic layer was dried over Na_2_SO_4_. After filtration, the solution was evaporated under vacuum and the residue subjected to flash chromatography purification (SiO_2_, 10% EtOAc/ hexanes) to yield the product (9.7 g, 91%) as a viscous oil, which crystallized to a pale yellow solid. Mp: 42–43 °C (lit: 39–41 °C [18]); IR (neat, ATR): cm^−1^ 2953, 2926, 2856, 1694, 1569, 1504, 1298, 1284, 1269, 1248, 1213, 1157, 1104, 973, 897, 825, 781, 731; ^1^H NMR (400 MHz, CDCl_3_): δ 9.81 (s, 1H), 7.38–7.34 (m, 2H), 6.94 (d, 1H, *J* = 8.0 Hz), 0.99 (s, 18H), 0.25 (s, 6H), 0.23 (s, 6H); ^13^C{^1^H} NMR (100 MHz, CDCl_3_): δ 190.7, 153.3, 147.6, 130.7, 125.2, 120.7, 120.5, 25.8, 25.7, 18.4, 18.2, −4.0, −4.2; MS (APCI, pos) *m/z* (%): 367(67) [M + H]^+^.

**4-**((***tert*-butyldimethylsilyl**)**oxy**)**benzaldehyde** (**8B**) [19]: Prepared from 4-hydroxybenzaldehyde following the general procedure described for **8A**, which afforded a quantitative yield (9.69 g) of a colorless oil. IR (neat, ATR): cm^−1^ 2955, 2930, 2858, 1697, 1506, 1255, 1154, 902; ^1^H NMR (500 MHz, CDCl_3_): δ 9.89 (s, 1H), 7.79 (d, 2H, *J* = 8.6 Hz), 6.94 (d, 2H, J = 8.6 Hz), 0.99 (s, 9H), 0.25 (s, 6H); ^13^C{^1^H} NMR (125 MHz, CDCl_3_): δ 190.8, 161.5, 131.9, 130.4, 120.5, 25.5, 18.2, −4.3; MS (ESI, pos) *m/z* (%): 237(55) [M + H]^+^.

**3,5-bis**((***tert*-butyldimethylsilyl**)**oxy**)**benzaldehyde** (**8C**) [20]: Prepared from 3,5-dihydroxybenzaldehyde following the general procedures described above for **8A**, which afforded a 93% yield (1.28 g) of a slightly yellow oil that slowly crystalized in the freezer. Mp = 28–29 °C; IR (neat, ATR, cm^−1^) 2955, 2930, 2857, 1702, 1587, 1330, 1253, 1165, 826; ^1^H NMR (500 MHz, CDCl_3_): δ 9.86 (s, 1H), 6.95 (d, 2H, *J* = 2.3 Hz), 6.59 (t, 1H, *J* = 2.3 Hz), 0.99 (s, 18H), 0.22 (s, 12H); ^13^C{^1^H} NMR (125 MHz, CDCl_3_): δ 191.7, 157.3, 138.4, 118.4, 114.3, 25.6, 18.2, −4.4; MS (APCI, pos) *m/z* (%): 367(17) [M + H]^+^.

**2,2-dimethyl-4*H*-benzo[*d*][1,3]dioxine-6-carbaldehyde 8**(**F**): Prepared as previously reported [21] from 4-bromosalicyl alcohol isopropylidene acetal to afford 11.17 g (93% yield) of colorless oil that slowly recrystallized in the freezer to afford off-white crystals. Mp = 55–58 °C (lit. 56–58 °C [21]); IR (neat, ATR): cm^−1^ 2992, 2869, 1690, 1496, 1384, 1269; ^1^H NMR (500 MHz, CDCl_3_): δ 9.85 (s, 1H), 7.70 (dd, 1H, *J* = 8.5, 1.0 Hz), 7.55 (t, 1H, *J* = 1.0 Hz), 6.93 (d, 1H, *J* = 8.5 Hz), 4.90 (s, 2H), 1.57 (s, 6H); ^13^C{^1^H} NMR (125 MHz, CDCl_3_): δ 190.7, 156.8, 130.5, 129.4, 126.6, 119.7, 117.7, 100.8, 60.6, 24.8; MS (ESI, pos) *m/z* (%): 215(14) [M + Na]^+^, 191(15), 161(11).

**General Procedure—Wittig reaction: Methyl** (***E***)**-3-**(**3,4-bis**((***tert*-butyldimethylsilyl**)**oxy**)**phenyl**)**acrylate** (**4A**) [22]: To a solution of methyl (triphenylphosphoranylidene)acetate (18.4 g, 55 mmol) in 200 mL of DCM at room temperature was added a ca. 1 M solution of 3,4-bis(*tert*-butyldimethylsilyloxy)benzaldehyde (**8A**, 6.33 g, 45.8 mmol) in DCM dropwise over 5 min. Upon completion of the addition, the mixture was stirred for an additional 18 h at room-temperature, and the solvent was removed by evaporation. The resulting pasty oil was diluted with 40 mL of hexanes, and the Ph_3_PO that precipitated was removed by filtration, and the filter cake was washed with two 40 mL portions of hexanes. The combined organic layers were concentrated under reduced pressure. Flash chromatography on silica gel (11:1 hexanes/EtOAc) of the residue afforded 4.17 g (91%) of 4′A0 as a white solid. Mp = 63.0–64.0 °C; IR (ATR, neat): cm^−1^ 2947, 2930, 2857, 1721, 1631, 1506, 1472, 1422, 1289, 1249, 1161, 1126, 911, 838, 813, 777; ^1^H NMR (500 MHz, CDCl_3_): δ 6.72 (d, 2H, *J* = 2.1 Hz), 7.64 (d, 1H, *J* = 15.9 Hz), 6.46 (t, 1H, *J* = 2.1 Hz), 6.44 (d, 1H, *J* = 15.9 Hz), 3.88 (s, 3H), 0.99 (s, 18H), 0.19 (s, 12H); ^13^C{^1^H} NMR (125 MHz, CDCl_3_): δ 167.7, 149.4, 147.1, 144.8, 128.0, 122.2, 121.1, 120.4, 115.4, 51.5, 25.9, 25.8, 18.5, 18.4, −4.0, −4.1; MS (APCI, pos) *m/z* (%): 423(66) [M + H]^+^.

**Methyl** (***E***)**-3-**(**4-**((***tert*-butyldimethylsilyl**)**oxy**)**phenyl**)**acrylate** (**4B**) [23]: Prepared from aldehyde **8B** (2.22 g, 9.4 mmol) following the general procedure described for **4A**, which afforded a **4B** as a white solid (2.73 g, 91% yield). Mp = 34.0–36.0 °C; IR (ATR, neat): cm^−1^ 2953, 2928, 2856, 1710, 1635, 1598, 1508, 1436, 1324, 1251, 1192, 1166, 988, 908, 834, 780; ^1^H NMR (400 MHz, CDCl_3_): δ 7.64 (d, 1H, *J* = 15.9), 7.41 (d, 2H, *J* = 8.6), 6.84 (d, 3H, *J* = 8.6), 6.30 (d, 1H, *J* = 15.9), 3.79 (s, 3H), 0.99 (s, 9H), 0.21 (s, 6H); ^13^C{^1^H} NMR (100 MHz, CDCl_3_): δ 167.6, 157.8, 144.5, 129.6, 127.6, 120.4, 120.4, 115.4, 51.5, 25.6, 18.2, −4.4; MS (ESI, pos) *m/z* (%): 293(24) [M + H]^+^.

**Methyl** (***E***)**-3-**(**3,5-bis**((***tert*-butyldimethylsilyl**)**oxy**)**phenyl**)**acrylate** (**4C**) [24]: Prepared from aldehyde **8C** following the general procedure described for **4A**, which afforded **4C** as a colorless oil, (3.93 g, 95% yield). IR (ATR, neat) cm^−1^ 2951, 2928, 2857, 1716, 1638, 1578, 1437, 1281, 1154, 1002, 828, 778; ^1^H NMR (500 MHz, CDCl_3_): δ 7.55 (d, 1H, *J* = 15.9 Hz), 6.62 (dd, 2H, *J* = 2.2, 0.4 Hz), 6.36 (t, 1H, *J* = 2.2 Hz), 6.34 (d, 1H, *J* = 15.9 Hz), 3.80 (s, 3H), 0.98 (s, 18H), 0.20 (s, 12H); ^13^C{^1^H} NMR (125 MHz, CDCl_3_): δ 167.4, 156.9, 144.8, 136.1, 117.9, 114.2, 113.2, 51.7, 25.6, 18.2, −4.4; MS (APCI, pos) *m/z* (%): 423(58) [M + H]^+^.

**Methyl** (***E***)**-3-**(**3,4-difluorophenyl**)**acrylate** (**4D**) [25]: Prepared from 3,4-difluorobenzaldehyde following the general procedure described for **4A**, which afforded **4D** as a white solid (3.17 g, 91%). Mp = 76.9–77.9 °C; IR (ATR, neat): cm^−1^ 3057, 2957, 2920, 1704, 1640, 1514, 1494, 1435, 1330, 1270, 1250, 1220, 1189, 1175, 1144, 1111, 987, 870, 814, 790; ^1^H NMR (400 MHz, CDCl_3_): δ 7.57 (d, 1H, *J* = 16.0 Hz), 7.32 (ddd, 1H, *J* = 11.0, 7.6, 2.1 Hz), 7.25–7.21 (m, 1H), 7.20–7.12 (m, 1H), 6.33 (dd, 1H, *J* = 16.0, 0.3 Hz), 3.79 (s, 3H); ^13^C{^1^H} NMR (100 MHz, CDCl_3_): δ 166.8, 151.5 (dd, *J* = 253, 13 Hz), 153.4 (dd, *J* = 249, 13 Hz), 142.4, 131.6 (dd, *J* = 5, 5 Hz), 124.7 (dd, *J* = 6, 3 Hz), 118.9 (d, *J* = 2 Hz), 117.8 (d, *J* = 17 Hz), 116.3 (d, *J* = 17 Hz), 51.7; ^19^F{^1^H} NMR (376 MHz, CDCl_3_): δ −134.2 (d, *J* = 21 Hz), −136.6 (d, *J* = 21 Hz); MS (APCI, pos) *m/z* (%): 199(5) [M + H]^+^.

**Methyl** (***E***)**-3-**(**benzo[*d*]**[1,3]**dioxol-5-yl**)**acrylate** (**4E**) [26]: Prepared from piperonal following the general procedure described for **4A**, which afforded **4E** as a white solid (5.4 g, 96%). Mp: 133–135 °C; IR (ATR, neat): cm^−1^ 2950, 2901, 1699, 1622, 1597, 1594, 1495, 1454, 1438, 1367, 1304, 1255, 1201, 1169, 1124, 1105, 1035, 1004, 931, 916, 821; ^1^H NMR (400 MHz, CDCl_3_): δ 7.53 (d, 1H, *J* = 16.0 Hz), 6.98–7.04 (m, 2H), 6.82 (d, 1H, *J* = 8.4 Hz), 6.26 (d, 1H, J = 16.0 Hz), 6.01 (s, 2H), 3.79 (s, 3H); ^13^C{^1^H} NMR (100 MHz, CDCl_3_): δ 167.6, 149.6, 148.3, 144.5, 128.8, 124.4, 115.7, 108.5, 106.4, 101.5, 51.6; MS (ESI, pos) *m/z* (%): 207(78) [M + H]^+^.

**Methyl** (***E***)**-3-**(**2,2-dimethyl-4*H*-benzo[*d*]**[1,3]**dioxin-6-yl**)**acrylate** (**4F**): Prepared from 2,2-dimethyl-4*H*-benzo(*d*)(1,3)dioxine-6-carbaldehyde (**8F**) following the general procedure described for **4A**, which afforded **4F** as a white, waxy solid (249 mg, 99%). IR (ATR, neat): cm^−1^ 2992, 2947, 2860, 1716, 1627, 1609, 1582, 1497, 1448, 1463, 1384, 1374, 1267, 1191, 1166, 1114, 1062, 953, 863, 830, 796; ^1^H NMR (400 MHz, CDCl_3_): δ 7.60 (1H, dd, *J* = 16.0, 3.0 Hz), 7.33–7.35 (m, 1H), 7.13 (s, 1H), 6.81 (dd, 1H, *J* = 8.4, 3.3 Hz), 6.26–6.29 (m, 1H), 4.83 (s, 2H), 3.77 (s, 3H), 1.53 (s, 6H); ^13^C{^1^H} NMR (100 MHz, CDCl_3_): δ 167.6, 153.3, 144.4, 128.0, 126.7, 124.9, 119.7, 117.6, 115.4, 100.1, 60.6, 51.5, 24.7; MS (ESI, pos) *m/z* (%): 271(10) [M + Na]^+^, 304(71) [M + Na + CH_3_OH]^+^; HRMS (ESI, pos): *m/z* calculated for C_14_H_16_O_4_ [M + H]^+^ 249.1121, found 249.1124.

**General Procedure–DIBAL-H Reduction:** (***E***)**-3-**(**3,4-bis**((***tert*-butyldimethylsilyl**)**oxy**)**phenyl**)**prop-2-en-1-ol** (**5A**): To a solution of **4A** (3.0 g, 7.1 mmol) in 70 mL of dry DCM in a 250 mL rb flask at −78 °C under Ar was added dropwise a solution of DIBAL-H (1.2 M solution in hexane, 18 mL, 21.6 mmol). Upon completion of the addition, the reaction mixture was allowed to warm to room temperature over 2 h and then carefully added dropwise to a stirred mixture of 2 M HCl (100 mL) and ice. After stirring for 30 min, the aqueous layer was extracted with CH_2_Cl_2_ (3 × 100 mL). The combined organic layers were washed with brine, dried over Na_2_SO_4_, and concentrated under reduced pressure to afford a colorless oil that was subjected to flash chromatography purification (SiO_2_, 0–10% EtOAc/hexanes) to yield the product (2.25 g, 80%) as a colorless oil. IR (ATR, neat): cm^−1^ 3360(br), 2929, 2886, 2857, 1598, 1511, 1471, 1419, 1301, 1254, 1124, 989, 905, 840, 781; ^1^H NMR (400 MHz, CDCl_3_): δ 6.88 (d, 1H, *J* = 2.2 Hz), 6.85 (dd, 1H, *J* = 8.3, 2.2 Hz), 6.77 (d, 1H, *J* = 8.2 Hz), 6.48 (dt, 1H, *J* = 15.8, 1.3 Hz), 6.18 (dt, 1H, *J* = 15.8, 5.9 Hz), 4.30–4.27 (m, 1H), 1.38 (t, 1H, *J* = 5.7 Hz), 0.99 (s. 9H), 0.98 (s, 9H), 0.20 (s, 6H), 0.19 (s, 6H); ^13^C{^1^H} NMR (100 MHz, CDCl_3_): δ 146.9, 131.2, 130.2, 126.3, 121.0, 119.9, 119.0, 63.9, 25.9, 25.8, 18.5, 18.4, −4.1; MS (ESI, pos) *m/z* (%): 417(25) [M + Na]^+^, 377(15); HRMS (ESI, pos): *m/z* calculated for C_21_H_38_NaO_3_Si_2_ [M + Na]^+^ 417.2257, found 417.2253.

(***E***)**-3-**(**4-**((***tert*-butyldimethylsilyl**)**oxy**)**phenyl**)**prop-2-en-1-ol** (**5B**) [23]: Prepared from **4B** following the general procedure described for **5A**, which afforded **5B** as a colorless oil, 1.3 g (85%). IR (ATR, neat): cm^−1^ 3325(br), 2954, 2928, 2857, 2602, 1507, 1252, 1168, 908, 835, 799, 778; ^1^H NMR (400 MHz, CDCl_3_): δ 7.26 (d, 2H, *J* = 8.5 Hz), 6.79 (d, 2H, *J* = 8.5 Hz), 6.55 (d, 1H, *J* = 15.9 Hz), 6.24 (dt, 1H, *J* = 15.5, 5.9 Hz), 4.39 (dd, 2H, *J* = 5.9, 1.2 Hz), 2.36 (d, 1H, *J* = 0.3 Hz), 0.98 (s, 9H), 0.20 (s, 6H); ^13^C{^1^H} NMR (100 MHz, CDCl_3_): δ 155.5, 131.0, 129.9, 127.6, 126.4, 120.2, 63.9, 25.7, 18.2, -4.4; MS (APCI, pos) *m/z* (%): 264(5) [M]^+^, 247(67).

(***E***)**-3-**(**3,5-bis**((***tert*-butyldimethylsilyl**)**oxy**)**phenyl**)**prop-2-en-1-ol** (**5C**) [24]. Prepared from **4C** following the general procedure described for **5A**, which afforded **5C** as a colorless oil, 914 mg (95%). IR (neat, ATR): cm^−1^ 3292(br), 2928, 2857, 1581, 1435, 1251, 1164, 1021, 827, 777; ^1^H NMR (400 MHz, CDCl_3_): δ 6.50–6.46 (m, 3H), 6.29 (dt, 1H, *J* = 10.0, 5.0 Hz), 6.25 (t, 1H, *J*= 2.1 Hz), 4.29 (dd, 2H, *J* = 5.6, 1.3Hz), 0.98 (s, 18H), 0.19 (s, 12H); ^13^C{^1^H} NMR (100 MHz, CDCl_3_): δ 156.6, 138.4, 131.0, 128.6, 111.7, 111.6, 63.7, 25.7, 18.2, −4.4.

(***E***)**-3-**(**3,4-difluorophenyl**)**prop-2-en-1-ol** (**5D**)**.** Prepared from **4D** following the general procedure described for **5A**, which afforded **5D** as a colorless oil, 1.18 g (92%). IR (neat, ATR): cm^−1^ 3271(br), 2987, 2845, 1603, 1514, 1289, 1270, 1090, 1018, 967; ^1^H NMR (400 MHz, CDCl_3_): δ 7.21–7.16 (m, 1H), 7.13–7.06 (m, 2H), 6.53 (d, 1H, *J* = 15.9 Hz), 6.27 (dt, 1H, *J* = 15.9, 5.5 Hz), 4.32 (dd, 2H, *J* = 5.5, 1.4 Hz); ^13^C{^1^H} NMR (100 MHz, CDCl_3_): δ 150.5 (dd, *J* = 247, 13 Hz), 149,8 (dd, *J* = 249, 13 Hz), 133.9 (dd, *J* = 6, 4 Hz), 129.6 (d, *J* = 2 Hz), 128.8, 122.6 (dd, *J* = 6, 3 Hz) 117.3 (d, *J* = 18 Hz), 114.7 (d, *J* = 8 Hz), 63.2; ^19^F NMR (376 MHz, CDCl_3_) δ: −137.9 (d, *J* = 21 Hz), −139.0 (d, *J* = 21 Hz); MS (ESI, pos) *m/z* (%): 171 [M + H]^+^(9), 157(12); HRMS (EI, pos): *m/z* calculated for C_9_H_8_OF_2_ [M]^+^ 170.0543, found 170.0539.

(***E***)**-3-**(**benzo[*d*]**[1,3]**dioxol-5-yl**)**prop-2-en-1-ol** (**5E**)**.** Prepared from **4E** following the general procedure described for **5A**, which afforded **5E** as a white solid, 1.94 g (90%). Mp: 79.5–80.0 °C; IR (neat, ATR): cm^−1^ 3350, 2920, 2895, 2851, 1499, 1441, 1243, 1083, 1034, 1003, 965, 920, 909; ^1^H NMR (500 MHz, CDCl_3_): δ 6.93 (d, 1H, *J* = 1.5 Hz), 6.81 (1H, dd, *J*= 8.0, 1.5 Hz), 6.75 (d, 1H, J = 8.0 Hz), 6.52 (dd, 1H, *J* = 15.8, 1.3 Hz) 6.20 (dd, 1H, *J* = 15.8, 5.9 Hz), 5.96 (s, 2H), 4.29 (td, 2H, *J* = 5.8, 1.3 Hz), 1.44 (t, 1H, *J* = 5.9 Hz); ^13^C{^1^H} NMR (100 MHz, CDCl3): δ 148.0, 147.3, 131.1, 131.0, 126.6, 121.1, 108.2, 105.7, 101.1, 63.7; MS (ESI, pos) *m/z* (%): 177(20) [M-H]^+^.

(***E***)**-3-**(**2,2-dimethyl-4*H*-benzo[*d*]**[1,3]**dioxin-6-yl**)**prop-2-en-1-ol** (**5*F***)**.** Prepared from 4′C3 following the general procedure described for 4A0, which afforded a white solid, 248 mg (quant.). Mp: 130.0–130.9 °C; IR (neat, ATR): cm^−1^ 3355(br), 2992, 2854, 1498, 1383, 1260, 1200, 1142, 1115, 1056, 1004, 975, 958, 868, 831; ^1^H NMR (500 MHz, CDCl_3_): δ 7.20 (dd, 1H, *J* = 8.5, 1.8 Hz), 6.98 (d, 1H, *J* = 2.0 Hz), 6.77 (d, 1H, *J* = 8.5 Hz), 6.49–6.52 (m, 1H), 6.21 (dt, 1H, *J* = 15.8, 5.9 Hz), 4.82 (s, 2H), 4.28 (dd, 2H, *J* = 5.9, 1.5 Hz), 1.54 (s, 6H); ^13^C{^1^H} NMR (125 MHz, CDCl_3_): δ 150.9, 130.8, 129.1, 125.5, 126.2, 122.7, 119.4, 117.3, 99.7, 63.8, 60.8, 24.7; MS (APCI, pos) *m/z* (%): 203(87) [M − H_2_O + H]^+^; HRMS (CI, pos) calculated for C_13_H_16_O_3_ [M]^+^ 220.1099, found 220.1101.

**General Procedure–Vinyl Dibromide Formation:** ((**4-**(**2,2-dibromovinyl**)**-1,2-phenylene**)**bis**(**oxy**))**bis**(***tert*-butyldimethylsilane**) (**6A**) [27]. To a 250 mL round-bottom flask with stirbar under Ar was placed 8.92 g (26.9 mmole) of CBr_4_. DCM (30 mL) was added to the flask via syringe, and the reaction flask was cooled in an ice bath. Triphenylphosphine (14.09 g, 53.7 mmole) was added the flask in portions, and the resulting mixture was stirred under Ar for 5 min. A solution of 4.94 g (13.5 mmole) of the aldehyde **8A** in 10 mL DCM was added to the reaction mixture, and the ice bath was removed. The mixture was allowed to stir at room temperature for 30 min, when TLC monitoring indicated the reaction was complete. Saturated sodium bicarbonate solution was carefully added until the aqueous layer was neutral by pH paper. The aqueous layer was then extracted with three 80 mL portions of DCM. The combined organic extracts were washed with saturated brine and then dried over anhydrous sodium sulfate. The dried solution was filtered and then evaporated under reduced pressure, and the residue subjected to chromatography (0–5% EtOAc/hex) to afford 6.36 g (91 % yield) of **6A** as a slightly yellow oil. IR (ATR, neat): cm^−1^ 2952, 2928, 2857, 1503, 1471, 1294, 1251, 1128, 881, 903, 835, 777; ^1^H NMR (400 MHz, CDCl_3_): δ 7.38 (s, 1H), 7.28 (d, 1H, *J* = 2.3 Hz), 6.99 (ddd, 1H, *J* = 8.3, 2.3, 0.6 Hz), 6.85 (d, 1H, *J* = 8.3 Hz), 1.05 (s, 9H), 1.04 (s, 9H), 0.28 (s, 6H), 0.27 (s, 6H); ^13^C{^1^H} NMR (100 MHz, CHCl_3_): δ 147.5, 145.6, 136.4, 128.4, 122.5, 120.7, 120.6, 86.8, 25.9, 25.8, 18.5, 18.4, −4.1; MS (ESI, neg) *m/z* (%): 521(2), 519(1), 523(1) [M − H]^–^.

***tert*-butyl**(**4-**(**2,2-dibromovinyl**)**phenoxy**)**dimethylsilane** (**6B**): Prepared from **8B** following the general procedure described for **6A** to afford 2.26 g (58% yield) of **8B** as a colorless oil: IR (ATR, neat): cm^−1^ 2953, 2928, 2856, 1601, 1505, 1462, 1252, 1171, 908, 870, 836, 775, 691; ^1^H NMR (500 MHz, CDCl_3_): δ 7.44–7.46 (m, 2H), 7.40 (s, 1H), 6.82–6.83 (m, 2H), 0.99 (s, 9H), 0.22 (s, 6H); ^13^C{^1^H} NMR (125 MHz, CHCl_3_): δ 156.0, 136.4, 129.8, 128.3, 119.9, 87.2, 25.6, 18.2, −4.4; MS (ESI, neg) *m/z* (%):393(8), 391(15), 389(7) [M − H] ^–^; HRMS (EI, pos): *m/z* calculated for C_14_H_20_OSiBr_2_ [M]^+^ 391.9630, found 391.9636.

((**5-**(**2,2-dibromovinyl**)**-1,3-phenylene**)**bis**(**oxy**))**bis**(***tert*-butyldimethylsilane**) (**6C**): Prepared from **8C** following the general procedure described for **6A** to afford 3.83 g (89% yield) of **6C** as a pale yellow oil. IR (ATR, neat): cm^−1^ 2854, 2929, 2857, 1580, 1431, 1334, 1252, 1163, 1030, 827, 810, 338; ^1^H NMR (400 MHz, CDCl_3_) δ: 7.53 (2, 1H), 6.65 (dd, 2H, *J* = 2.1, 0.4 Hz), 6.33 (t, 1H, *J*= 2.1 Hz), 0.98 (s, 18H), 0.20 (s, 12H); ^13^C{^1^H} NMR (100 MHz, CDCl_3_) δ 156.5, 136.7, 136.6, 113.4, 112.7, 89.4, 25.7, 18.2, −4.38; MS (ESI, neg) *m/z* (%): 521(6) [M − H]^-^; HRMS (EI, pos): *m/z* calculated for C_20_H_34_O_2_Si_2_Br_2_ (M)^+^ 522.0444, found 522.0441.

**4-**(**2,2-dibromovinyl**)**-1,2-difluorobenzene** (**6D**) [28] Prepared from 3,4-difluorobenzaldehyde following the general procedure described for **6A** to afford 10.13 g (97% yield) of a **6D** as a colorless oil. IR (neat, ATR): cm^−1^ 3019, 1604, 1513, 1432, 1418, 1287, 1214, 1115, 882; ^1^H NMR (400 MHz, CDCl_3_): δ 7.22–7.15 (m, 1H), 7.39 (d, 1H, *J* = 0.27), 7.39–7.48 (m, 2H); ^13^C{1H} NMR (100 MHz, CDCl_3_): δ 91.4 (d, *J* = 2.52), 117.1 (d, J = 16.2 Hz), 117.3 (d, *J* = 14.8 Hz), 125.5 (dd, *J* = 6.5, 3.6), 132.1 (dd, *J* = 6.5, 4.3), 135.1 (d, *J* = 1.6), 150.0 (dd, *J* = 247.9, 11.7 Hz), 150.1 (dd, *J* = 249.9, 10.7 Hz); ^19^F{^1^H} NMR (376 MHz, CDCl_3_) δ: 136.2 (d, J = 20.7 Hz), 136.8 (d, J = 20.7 Hz).

**5-**(**2,2-dibromovinyl**)**benzo(*d*)(1,3)dioxole** (**6E**) [29]: Prepared from piperonal following the general procedure described for **6A** to afford 3.87 g (95% yield) of **6E** as a crystalline yellow solid. Mp = 50.9–51.7 °C; IR (ATR, neat): 2962, 2907, 1500, 1486, 1443, 1305, 1255, 1192, 1098, 1032, 924, 864, 840, 798, 752; ^1^H NMR (400 MHz, CDCl3) δ: 5.98 (2H, s), 6.80 (1H, d, *J* = 8.1 Hz), 6.95 (1H, ddd, *J* = 8.1, 1.7, 0.7 Hz), 7.18 (1H, dd, *J* = 1.7, 0.4), 7.37 (1H, s); ^13^C{^1^H} NMR 100 MHz, CDCl_3_) δ: 87.9, 101.4, 108.1, 108.2, 123.4, 129.2, 136.3, 147.6, 147.8; MS (APCI, pos) *m/z* (%): 309(1), 307(2), 305(1) [M + H]^+^.

**General Procedure–Corey-Fuchs Elimination with Trapping by CO_2_: 3-**(**3,4-bis**((***tert*-butyldimethylsilyl**)**oxy**)**phenyl**)**propiolic acid** (**7A**): To a solution of 5.0 g (9.57 mmol) of the vinyl dibromide **6A** in 40 mL of THF under argon at −78 °C was added dropwise 8.8 mL of a 2.6 M solution (2.4 equivalent) of n-butyllithium in hexane. The reaction mixture was stirred at −78 °C for 1h, the ice bath was removed, and the reaction mixture was allowed to warm to room temperature. Chips of clean dry ice (ca. 5 g, large excess) were carefully added to the reaction mixture and allowed to fully dissolve/sublime while the mixture returned to room temperature. Water (20 mL) was added to the reaction mixture, and the THF was removed under reduced pressure. The residue was transferred to a separatory funnel, and ice-cold 1 M HCl (75 mL) and DCM (100 mL) were added. The aqueous phase was acidified with 6N HCl until the organic phase was no longer cloudy. The organic phase was collected. The aqueous phase was extracted twice more with 50 mL DCM, and extracts were combined, washed with saturated brine, dried over anhydrous sodium sulfate, and evaporated. Recrystallization (EtOAc/hex) of the residue afford 2.77g (78%) of **7A** as a white solid. Mp = 143–145 °C. IR (KBr): cm^−1^ 3100, 2955, 2932, 2859, 2208, 1676, 1507; ^1^H NMR (400 MHz, CDCl_3_): δ 7.13 (1H, dd, *J =* 8.3, 2.0 Hz), 7.07 (1H, d, *J =* 2.0 Hz), 6.81 (1H, d, *J =* 8.3 Hz), 0.99 (9H, s), 0.98 (9H, s), 0.22 (6H, s), 0.21 (6H, s). ^13^C{^1^H} NMR (100 MHz, CDCl_3_): δ 158.5, 150.8, 147.0, 127.9, 125.7, 121.2, 111.4, 90.1, 79.3, 25.8, 18.5, 18.4, −4.1, −4.2; MS (ESI, neg) *m/z* (%): 405(18) [M − H]^–^; Matches lit [14].

**3-**(**4-**((***tert*-butyldimethylsilyl**)**oxy**)**phenyl**)**propiolic acid** (**7B**)**.** Prepared from **6B** following the general procedure described for **7A** to afford 560 mg (62% yield) of **7B** as a tan solid. Mp = 104.2–106.3 °C; IR (ATR, neat): 2951, 2928, 2857, 2197, 1667, 1595, 1507, 1255, 1208m 1163, 903, 835, 781 cm^−1^; ^1^H NMR (400 MHz, CDCl_3_) δ: 7.51 (d, 2H, *J* = 8.7 Hz), 6.83 (d, 2H, *J* = 8.7 Hz), 0.98 (s, 9H), 0.22 (s, 6H). ^13^C{^1^H} NMR (100 MHz, CDCl_3_): δ 158.8, 158.6, 135.3, 120.5, 111.5, 90.0, 79.8, 25.5, 18.2, −4.4; MS (ESI, neg) *m/z* (%): 275(15) [M − H]^–^, 231(46), 160(6), 135(16), 117(7); HRMS (ESI, neg): *m/z* calculated for C_15_H_20_O_3_Si [M − H]^–^ 275.1109, found 275.1112.

**3-**(**3,5-bis**((***tert*-butyldimethylsilyl**)**oxy**)**phenyl**)**propiolic acid** (**7C**): Prepared from **6C** following the general procedure described for **7A** to afford 1.6 g (quant. yield) of **7C** as a white semi-solid. IR (ATR, neat): cm^−1^ 2954, 2928, 2857, 2223, 1677, 1578, 1427, 1251, 1167, 1028, 825, 776; ^1^H-NMR (CDCl_3_, 500 MHz): δ 6.71 (2H, d, J = 2.2 Hz), 6.45 (1H, t, J = 2.2 Hz), 0.97 (18H, s), 0.21 (12H, s); ^13^C{^1^H} NMR (100 MHz, CHCl_3_): δ 158.2, 156.7, 120.0, 118.1, 116.1, 88.9, 79.4, 25.6, 18.2, −4.4; MS (ESI, neg) *m/z* (%): 405(11) (M-H), 361(49), 247(13),101(8); HRMS (ESI, neg): *m/z* calculated for C_9_H_4_F_2_O_2_ [M − H]^–^ 181.0107, found 181,0110.

**3-**(**3,4-difluorophenyl**)**propiolic acid** (**7D**)**.** Prepared from **6D** following the general procedure described for **7A** to afford 193 mg (23% yield) of **7D** as a pale yellow solid. Mp 169.0–171.6 °C; IR (ATR, neat): cm^−1^ 3078, 2901, 2851, 2221, 1795, 1685, 1629, 1601, 1511, 1438, 1277, 1251, 1207, 1042, 916, 865; ^1^H-NMR (CDCl_3_, 400 MHz): δ 10.18 (s(br), 1H), 7.46–7.42 (m, 1H), 7.40–7.39 (m, 1H), 7.38 (q, 1H, *J* = 9.0 Hz); ^13^C{^1^H} NMR (100 MHz, CHCl_3_): δ 167.7, 151.6 (dd, *J* = 253, 12 Hz), 149.8 (dd, *J* = 248, 13 Hz), 130.6, 121.8 (d, *J* = 19 Hz), 118.6 (d, *J* = 18 Hz), 116.7 (d, *J* = 18 Hz), 116.3 (dd, *J* = 4, 3 Hz), 82.1 (d, *J* = 3 Hz), 82.0; MS (ESI, neg) *m/z* (%): 181(21) (M-H); HRMS (ESI, neg): *m/z* calculated for C_9_H_4_F_2_O_2_ [M − H]^–^ 181.0107, found 181.0110.

**3-**(**benzo(*d*)(1,3)dioxol-5-yl**)**propiolic acid** (**7E**) [30]. Prepared from **6E** following the general procedure described for **7A** to afford 1.9 g (81% yield) of **7E** as an orange solid. Mp = 162.8–165.6 °C (dec); IR (ATR, neat): cm^−1^ 2982, 2915, 2871, 2206, 1667, 1489, 1446, 1410, 1305, 1243, 1193, 1098, 1035, 923, 860, 811 cm^−1^;^1^H NMR (400 MHz, CD3OD) δ: 6.02 (2H, s); 6.86 (1H, dd, J = 8.06, 0.36), 7.01 (1H, dd, J = 1.61, 0.35), 7.15 (1H, dd, J = 8.06, 1.64); ^13^C NMR (400 MHz, CD3OD) δ: 80.7, 87.2, 103.3, 109.8, 113.1, 113.8, 129.7, 149.3, 151.6, 156.8; MS (ESI, neg) *m/z* (%): 189(9) [M − H]^–^, 145(14), 75(54).

**3-**(**2,2-dimethyl-4*H*-benzo(*d*)(1,3)dioxin-6-yl**)**propiolic acid** (**7F**): A solution of 300 mg (1.6 mmole) of 6-ethynyl-2,2-dimethyl-4*H*-benzo(*d*)(1,3)dioxine [16] in 1 mL of THF was cooled to −78 °C. To this solution was added dropwise 637 µL (1.6 mmole) of 2.5 M BuLi in hexanes. The solution was warmed to −20 °C, stirred at −20 °C for 30 min, and then, CO_2_ was bubbled through the reaction mixture over 4 h during which the reaction was allowed to warm to rt. The reaction was quenched by addition of a small amount of water, the solvent was evaporated, and the residue was subjected to flash chromatography (10% EtOAc/Hex + 1% AcOH) to afford 229 mg (62%) of **7F** as a white solid. Mp = 119.5–121.0 °C; IR (ATR, neat): cm^−1^ 2989, 2938, 2866, 2205, 1659, 1608, 1495, 1327, 1273, 1252, 1218, 1120, 884; ^1^H-NMR (CDCl_3_, 500 MHz): δ 7.32 (1H, dd, *J* = 8.8, 2.0 Hz), 7.28 (1H, d, *J* = 2.0 Hz), 6.81 (1H, d, *J* = 8.5 Hz), 4.83 (2H, s), 1.55 (6H, s); ^13^C{^1^H} NMR (125 MHz, CHCl_3_): δ 157.6, 154.0, 144.6, 130.4, 119.9, 117.8, 110.6, 100.6, 89.6, 79.5, 60.4, 24.8; MS (ESI, neg) *m/z* (%): 231(4) [M − H]^–^, 187(38), 129(11), 75(4), 69(14), 59(20); HRMS (ESI, neg): *m/z* calculated for C_13_H_12_O_4_ [M − H]^-^ 231.0663, found 231.0665.

**General Procedure – DCC Coupling:** (***E***)**-3-**(**3,4-bis**((***tert*-butyldimethylsilyl**)**oxy**)**phenyl**)**allyl 3-**(**3,4-bis**((***tert*-butyldimethylsilyl**)**oxy**)**phenyl**)**propiolate** (**2AA**): The carboxylic acid (**7A**) (2.413 g, 5.93 mmol) and DMAP (112 mg, 0.92 mmol) were placed in a round-bottomed flask that was then purged with argon. To the flask was added 40 mL of dry DCM and ca. 1 g of 4Å molecular sieves. A solution of the alcohol (**4A**) (1.815 g, 4.60 mmol) in 15 mL DCM was added via syringe. The flask was placed in an ice bath, and a solution of 1.43g (6.90 mmol) of DCC in 3 mL DCM was added dropwise via syringe. The ice bath was removed, and the reaction was allowed to stir at room temperature for 2 h. The crude reaction mixture was filtered through a plug of silica gel, which was washed with 50 mL of 30% ethyl acetate/hexanes. The filtrate was evaporated and purified by flash chromatography (0–2% ethyl acetate/hexanes) to afford 2.91 g (81% yield) of ester **2AA** as a pale yellow oil. IR (ATR, neat): cm^−1^ 2955, 2930, 2216, 1711, 1511; ^1^H NMR (400 MHz, CDCl_3_): δ 7.10 (1H, dd, *J =* 8.3, 2.0 Hz), 7.04 (1H, d, *J =* 2.0 Hz), 6.88 (2H, m), 6.78 (2H, m), 6.59 (1H, d, *J =* 15.8 Hz), 6.14 (1H, dt, *J =* 15.8, 6.8 Hz), 4.84 (2H, dd, 6.8, 1.1 Hz), 0.99 (9H, s), 0.97 (18H, s), 0.96 (9H, s), 0.22 (6H, s), 0.21 (6H, s), 0.20 (6H, s), 0.19 (6H, s); ^13^C{1H} NMR (100 MHz, CD_3_COCD_3_): δ 154.1, 151.1, 148.0, 147.9, 147.7, 135.6, 131.1, 128.3, 126.0, 122.4, 122.0, 121.5, 121.3, 120.1, 112.9, 86.9, 80.5, 67.1, 26.4, 26.3, 26.2, 19.2, 19.09, 19.07, 19.04, −3.81, −3.84, −3.9; HRMS (ESI, pos) calc. for C_42_H_70_NaO_6_Si_4_ [M + Na]^+^ 805.4142, found 805.4148.

(***E***)**-3-**(**4-**((***tert*-butyldimethylsilyl**)**oxy**)**phenyl**)**allyl 3-**(**4-**((***tert*-butyldimethylsilyl**)**oxy**)**phenyl**)**propiolate** (**2BB**)**.** Prepared from **4B** and **7B** following the general procedure described for **2AA** to afford 64 mg (42% yield) of **2BB** as a colorless oil. IR(ATR, neat): cm^−1^ 2914, 1848, 2236, 1734, 1623, 1568, 1559, 1309, 1268, 1241, 1178, 1086, 640; ^1^H-NMR (CDCl_3_, 500 MHz): δ 7.48 (d, 2H, *J* = 8.8 Hz), 7.28 (d, 2H, *J* = 8.5 Hz), 6.83–6.79 (m, 4H), 6.65 (d, 1H, *J* = 15.8 Hz), 6.19 (dt, 1H, *J* = 15.8, 6.7 Hz), 4.85 (dd, 2H, *J*= 6.7, 1.1 Hz), 0.99 (s, 9H), 0.98 (s, 9H), 0.21 (s, 6H), 0.20 (s, 6H); ^13^C{^1^H} NMR (125 MHz, CDCl_3_): δ 158.1, 155.9, 154.1, 135.1, 134.9, 129.3, 127.9, 120.4, 120.2, 120.0, 112.0, 87.3, 80.1, 66.6, 25.6, 25.5, 18.2, −4.4; MS (ESI, pos) *m/z* (%): 523(3) [M + H]^+^.

(***E***)**-3-**(**3,5-bis**((***tert*-butyldimethylsilyl**)**oxy**)**phenyl**)**allyl 3-**(**3,5-bis**((***tert*-butyldimethylsilyl**)**oxy**)**phenyl**)**propiolate** (**2CC**): Prepared from **4C** and **7C** following the general procedure described for **2AA** to afford 349 mg (70% yield) of **2CC** as off-white needles. Mp = 77.4–80.7 °C; IR (ATR, neat): cm^−1^ 2951, 1919, 1857, 2219, 1706, 1579, 1437, 1427, 1250, 1233, 1163, 1153, 1026, 825, 811, 777; ^1^H NMR (400 MHz, CD3Cl): δ 6.69 (d, 2H, *J* = 2.2 Hz), 6.59 (d, 1H, *J* = 15.8 Hz), 6.51 (d, 2H, *J* = 2.2 Hz), 6.43 (t, 1H, *J* = 2.2 Hz), 6.27–6.20 (m, 2H), 4.86 (dd, 1H, *J* = 6.6, 1.0 Hz), 0.98 (s, 18H), 0.97 (s, 18H), 0.19 (s, 12H), 0.18 (s, 12H); ^13^C{1H} NMR (100 MHz, CDCl_3_): δ 156.7, 156.6, 153.7, 137.7, 135.3, 122.1, 120.4, 177.9, 115.7, 112.2, 112.9, 86.6, 79.8, 66.5, 25.7, 25.6, 18.1, −4.4, −4.5; MS (ESI, pos) *m/z* (%): 783(9) (M + H), 395(11), 377(14); HRMS (APCI, pos) calculated for C_42_H_70_O_6_Si_4_ [M + H]^+^ 783.4322, found 783.4303.

(***E***)**-3-**(**3,4-difluorophenyl**)**allyl 3-**(**3,4-difluorophenyl**)**propiolate** (**2DD**): Prepared from **4D** and **7D** following the general procedure described for **2AA** to afford 154 mg (76% yield) of **2DD** as an amber oil. IR (ATR, neat): cm^−1^ 2949, 2938, 2856, 2215, 1724, 1644, 1603, 1513, 1435, 1293, 1269, 1242, 1165, 1142, 965, 811, 785; ^1^H NMR (400 MHz, CDCl_3_): δ 7.42–7.33 (2H, m), 7.23–7.09 (4H, m), 6.63 (1H, d, *J* = 15.87), 6.23 (1H, dt, *J* = 15.84, 6.46), 4.87 (2H, dd, *J* = 6.47, 1.23); ^13^C{^1^H} NMR (100 MHz, CDCl_3_): δ 153.2, 152.1 (dd, *J* = 256, 13 Hz), 150.4 (dd, *J* = 251, 16 Hz), 150.2 (dd, *J* = 158, 23 Hz), 150.0 (dd, *J* = 251 13 Hz), 133.2 (dd, *J* = 6, 4 Hz), 133.1, 130.0 (dd, J = 7, 4 Hz), 123.1 (d, *J* = 2Hz), 123.0 (dd, *J* = 7, 4 Hz), 121.9 (d, *J* = 19 Hz), 118.0 (d, *J* = 17 Hz), 117.4 (d, *J* = 17 Hz), 116.2 (dd, *J* = 7, 4 Hz), 115.0 (d, *J* = 17 Hz), 84.1 (d, *J* = 2 Hz), 80.5, 66.1; ^19^F{^1^H} NMR (376 MHz, CDCl_3_) δ: −137.0 (d, *J* = 20 Hz), −139.3 (d, *J* = 21 Hz), −140.1 (d, *J* = 20 Hz), −140.9 (d, *J* = 21 Hz); MS (APCI, pos) *m/z* (%): 389(13) (M + MeOH + Na^+^); HRMS (CI, pos) calculated for C_18_H_12_F_4_O_2_ [M]^+^ 336.0773, found 336.0771.

(***E***)**-3-**(**benzo(*d*)(1,3)dioxol-5-yl**)**allyl 3-**(**benzo(*d*)(1,3)dioxol-5-yl**)**propiolate** (**2EE**) [31]: Prepared from **4E** and **7E** following the general procedure described for **2AA** to afford 1.58 g (86% yield) of **2EE** as an off-white, chalky solid. 86%. Mp = 104.9–106.5 °C; IR(ATR, neat): cm^−1^ 2916, 2901, 2851, 2207, 1692, 1618, 1490, 1444, 1303, 1236, 1185, 1096, 1034, 931, 804; ^1^H NMR (400MHz, CDCl_3_): δ 7.15 (1H, dd, *J* = 8.1, 1.6), 7.00–6.93 (2H, m), 6.84 (1H, dd, *J* = 8.2, 1.6), 6.80–6.75 (2H, m), 6.62 (1H, d, *J* = 15.8), 6.15 (1H, d, *J* = 15.8), 5.95 (2, 2H), 5.94 (s, 2H), 4.83 (2H, dd, J = 6.7, 1.2); ^13^C{^1^H} NMR (100 MHz, CDCl_3_): δ 153.8, 150.0, 148.0, 147.7, 147.5, 135.0, 130.3, 128.8, 121.6, 120.2, 112.4, 108.7, 108.2, 105.8, 101.7, 101.1, 87.0, 79.4, 66.4. MS (ESI, pos) *m/z* (%): 373(3) [M + Na]^+^.

(***E***)**-3-**(**2,2-dimethyl-4*H*-benzo(*d*)(1,3)dioxin-6-yl**)**allyl 3-**(**2,2-dimethyl-4*H*-benzo(*d*)(1,3)dioxin-6-yl**)**propiolate** (**2FF**): Prepared from **4F** and **7F** following the general procedure described for **2AA** to afford 15 mg (50% yield) of **2FF** as a colorless oil. IR(ATR, CHCl_3_): cm^−1^ 2993, 2940, 2856, 2211, 1704, 1612, 1580, 1497, 1384, 1375, 1312, 1290, 1270, 1254, 1234, 1198, 1144, 1115, 956, 873; ^1^H NMR (400 MHz, CDCl_3_): δ 7.39 (dd, 1H, *J* = 8.5, 1.9 Hz), 7.24–7.21 (m, 2H), 7.02 (s, 1H), 6.79 (t, 2H, *J* = 8.1 Hz), 6.62 (d, 1H, *J* = 15.8 Hz), 6.17 (dt, 1H, *J* = 15.8, 6.7), 4.85–4.81 (m, 4H), 4.80 (s, 2H), 1.54 (s, 12H); ^13^C{^1^H} NMR (100 MHz, CDCl_3_): δ 154.0, 153.6, 151.4, 134.9, 133.3, 130.1, 128.5, 126.6, 123.1, 120.2, 119.8, 119.4, 117.6, 117.3, 111.0, 100.4, 99.8, 87.1, 79.8, 66.6, 60.8, 60.4, 24.8, 24.7; MS (ESI, pos) *m/z* (%): 435(3) [M + H]^+^; HRMS (APCI, pos) calculated for C_26_H_26_O_6_ [M + H]^+^ 435.1802, found 435.1788.

(***E***)**-3-**(**benzo(*d*)(1,3)dioxol-5-yl**)**allyl 3-**(**3,4-bis**((***tert*-butyldimethylsilyl**)**oxy**)**phenyl**)**propiolate** (**2EA**): Prepared from **4E** and **7A** following the general procedure described for **2AA** to afford 139 mg (59% yield) of **2EA** as slightly yellow needles. Mp 95.3–96.7 °C; IR(ATR, neat): cm^−1^ 2927, 2855, 2208, 1556, 1493, 1437, 1411, 1318, 1275, 1238, 1155, 1119, 1035, 935, 896, 839, 783; ^1^H NMR (500MHz, CDCl_3_): δ 7.10 (dd, 1H, *J* = 8.3, 2.1 Hz), 7.05 (d, 1H, *J* = 2.1 Hz), 6.94 (d, 1H, *J* = 1.7 Hz), 6.84 (dd, 1H, *J* = 8.0, 1.7 Hz), 6.79 (d, 1H, *J* = 8.3 Hz), 6.76 (8.0 Hz), 6.63 (d, 1H, *J* = 15.8 Hz), 6.16 (dt, 1H, *J* = 15.8, 6.7 Hz), 5.96 (s, 2H), 4.84 (dd, 2H, *J* = 6.7, 1.2 Hz), 0.99 (s, 9H), 0.98 (s, 9H), 0.22 (s, 6H), 0.21 (s, 6H);^13^C{1H} NMR (125 MHz, CDCl_3_): δ 154.1, 150.2, 148.1, 147.7, 146.9, 135.1, 130.4, 127.4, 125.4, 121.6, 121.1, 120.3, 111.9, 108.2, 105.9, 101.1, 87.5, 79.6, 66.4, 25.8, 18.5, 18.4, −4.1, −4.2; MS (ESI, pos) *m/z* (%): 567(21) [M + H]^+^; HRMS (APCI, pos) calculated for C_31_H_42_O_6_Si_2_ [M + H]^+^ 567.2593, found 567.2586.

(***E***)**-3-**(**benzo(*d*)(1,3)dioxol-5-yl**)**allyl 3-phenylpropiolate** (**2EG**) [30]: Prepared from **4E** and phenylpropiolic acid following the general procedure described for **2AA** to afford 179 mg (51% yield) of **2EG** as a white solid. Mp = 55.7–57.2 °C. IR(ATR, neat) cm^−1^: 2996, 2896, 2210, 1697, 1501, 1490, 1444, 1299, 1280, 1251, 1182, 1170, 1125, 1036, 953, 930, 911, 752; ^1^H NMR (400MHz, CDCl_3_): δ 7.60–7.58 (m, 2H), 7.45 (t, 1H, *J* = 7.5 Hz), 7.37 (t, 2H, *J* = 7.6 Hz), 6.95 (d, 1H, *J* = 1.4 Hz), 6.85 (dd, 1H, *J* = 8.0, 1.4 Hz), 6.77 (d, 1H, *J* = 8.0 Hz), 6.63 (d, 1H, *J* = 15.8 Hz), 6.16 (dt, 1H, *J* = 15.8, 6.7 Hz), 5.96 (s, 2H), 4.86 (d, 2H, *J* = 6.7 Hz); ^13^C{^1^H} NMR (100 MHz, CDCl_3_): δ 153.8, 148.0, 147.8, 135.2, 132.9, 130.6, 128.5, 121.7, 120.2, 119.5, 108.3, 105.8, 101.1, 86.5, 80.5, 66.6; MS (ESI, pos) *m/z* (%): 329(6) [M + Na]^+^.

**General Procedure—Pd-Catalyzed Decarboxylative Coupling:** (***E***)**-**((**pent-1-en-4-yne-1,5-diylbis**(**benzene-4,1,2-triyl**))**tetrakis**(**oxy**))**tetrakis**(***tert*-butyldimethylsilane**) (**1′AA**) [14]: A solution of 134 mg (0.171 mmol) of ester **2AA** in 2.5 mL of freshly distilled THF was transferred under argon to a reaction tube containing 10 mg (0.0086 mmol) of Pd(P(Ph)_3_)_4_, and the tube was sealed. After heating for 4 h in an 80 °C oil bath, the contents of the tube were transferred to a round-bottomed flask with EtOAc, the solvent evaporated, and the residue subjected to flash chromatography (0–1% EtOAc/hexanes) to afford **1′AA** as a yellow oil (105 mg, 0.142 mmol, 83%). ^1^H NMR and IR matched, which was previously reported [14]. ^13^C{^1^H} NMR (100 MHz, CDCl_3_): δ 147.3, 146.8, 146.6, 146.4, 130.9, 125.3, 124.3, 122.4, 121.0, 120.9, 119.6, 118.9, 116.5, 105.0, 85.2, 82.4, 26.0, 25.9, 22.9, 18.5, 18.4, −4.1. HRMS (ESI^+^) calculated for C_41_H_71_O_4_Si_4_ [M + H]^+^ 739.4424, found 739.4422.

(***E***)**-**((**pent-1-en-4-yne-1,5-diylbis**(**4,1-phenylene**))**bis**(**oxy**))**bis**(***tert*-butyldimethylsilane**) (**1′BB**): Prepared from **2BB** following the general procedure described for **1′AA** to afford 12 mg (22% yield) of a yellow oil. IR(ATR, neat): cm^−1^ 2954, 2929, 2857, 2220, 1588, 1252, 1164, 906, 835, 779; ^1^H NMR (500 MHz, CD3Cl): δ 7.33–7.31 (m, 2H), 7.26–7.24 (m, 2H), 6.79–6.76 (m, 4H), 6.62 (d, 1H, *J* = 15.7 Hz), 6.12–6.07 (m, 1H), 3.32 (dd, 2H, *J* = 5.6, 1.6 H), 0.99 (s, 9H), 0.98 (s, 9H), 0.19 (s, 12H); ^13^C{^1^H} NMR (125 MHz, CDCl_3_): δ 155.5, 155.1, 132,9, 130.7, 130.5, 127.3, 122.4, 120.1, 120.0, 116.5, 85.5, 82.4, 25.7, 25.6, 22.9, 18.2, –4.4; MS (APCI, pos) *m/z* (%): 477(16) [M + H]^+^; HRMS (ESI^+^) calculated for C_29_H_40_O_2_Si_2_ [M + H]^+^ 477.2640, found 477.2640.

(***E***)**-**((**pent-1-en-4-yne-1,5-diylbis**(**benzene-5,1,3-triyl**))**tetrakis**(**oxy**))**tetrakis**(***tert*-butyldimethylsilane**) (**1′CC**): Prepared from **2CC** following the general procedure described for **1′AA** to afford 54 mg (56% yield) of a colorless oil. IR(ATR, neat): cm^−1^ 2954, 2929, 2857, 1578, 1426, 1343, 1252, 1162, 1026, 827, 778; ^1^H NMR (500 MHz, CD3Cl): δ 6.57–6.50 (m, 3H), 6.50 (d, 2H, *J* = 2.2 Hz), 6.30 (t, 1H, *J* = 2.2 Hz), 6.23 (t, 1H, *J* = 2.2 Hz), 6.16 (dt, 1H, *J =* 15.7, 5.6 Hz), 3.32 (dd, 2H, *J* = 5.6, 1.7 Hz), 0.98 (s, 18H), 0.97 (s, 18H), 0.99 (s, 24H); ^13^C{1H} NMR (125 MHz, CDCl_3_): δ 156.6, 156.3, 138.9, 131.3, 124.6, 124.3, 116.8, 112.8, 111.6, 111.3, 86.2, 82,7, 25.7, 25.6, 22.9, 18.2, 18.1, −4.2, −4.4; MS (APCI, pos) *m/z* (%): 739(34) [M + H]^+^; HRMS (APCI, pos) calculated for C_41_H_70_O_4_Si_4_ [M + H]^+^ 739.4424, found 739.4396.

(***E***)**-4,4’-**(**pent-1-en-4-yne-1,5-diyl**)**bis**(**1,2-difluorobenzene**) (**1DD**): Prepared from **2DD** following the general procedure described for **1′AA** to afford 50 mg (28% yield) of **1DD** as a yellow oil. IR(ATR, neat): cm^−1^ 2930, 2204, 1599, 1513, 1430, 1290, 1216, 1114, 965, 872, 820, 773; ^1^H NMR (400 MHz, CDCl_3_): δ 7.25–7.06 (6H, m), 6.59 (1H, d, *J* = 15.7 Hz), 6.15 (1H, dtd, *J* = 15.7, 5.6), 3.34 (dd, 2H, *J* = 5.6, 1.3 Hz); ^13^C{^1^H} NMR (100 MHz, CDCl_3_): δ 150.4 (dd, J = 247.6, 12.9 Hz), 150.3 (dd, J = 250.6, 12.5 Hz), 149.9 (dd, J = 248.8, 12.9 Hz), 149.7 (dd, J = 248.8, 12.8 Hz), 134.2 (dd, J = 5.7, 4.1 Hz), 129.7, 128.2 (dd, J = 6.2, 3.6 Hz), 124.9 (d, J = 1.9 Hz), 122.4 (dd, J = 6.1, 3.4 Hz), 120.5 (d, J = 18.3 Hz), 120.3 (dd, J = 7.6, 4.2 Hz), 117.3 (d, J = 17.7 Hz), 117.2 (d, J = 17.3 Hz), 114.6 (d, J = 17.6 Hz), 86.9 (d, J = 1.3 Hz Hz), 81.0, 22.7; MS (ESI, pos) *m/z* (%): 289(8) [M − H]^+^; HRMS (ESI, pos) calculated for C_17_H_10_F_4_ [M]^+^ 290.0719, found 290.0714.

(***E***)**-5,5’-**(**pent-1-en-4-yne-1,5-diyl**)**bis**(**benzo(*d*)(1,3)dioxole**) (**1EE**): Prepared from **2EE** following the general procedure described for **1′AA** to afford 350 mg (80% yield) of **1EE** as thin yellow needles. Mp = 58.2–60.0 °C; IR(ATR, neat): cm^−1^ 2908, 2854, 1486, 1440, 1246, 1207, 1097, 1036, 967, 925, 813; ^1^H NMR (400 MHz, CD_3_COCD_3_): δ 7.03 (d, 1H, *J =* 1.7 Hz), 6.98 (dd, 1H, *J* = 8.0, 1.6 Hz), 6.91–6.90 (m, 1H), 6.88 (dd, 1H, *J* = 8.0, 1.7 Hz), 6.82 (dd, 1H, *J* = 8.0, 0.4 Hz), 6,79 (d, 1H, *J* = 8.0 Hz), 6.65 (dt, 1H, *J* = 15.7, 1.8 Hz), 6.18 (dt, 1H, *J* = 15.7, 5.7), 6.02 (s, 2H), 5.98 (s, 2H), 3.32 (dd, 2H *J* = 5.7, 1.8); ^13^C{^1^H} NMR (100 MHz, CD_3_COCD_3_): δ 149.0, 148.6, 148.4,148.0, 132.6, 131.6, 126.7, 123.5, 121.7, 117.9, 112.0, 109.1, 108.9, 106.3, 102.3, 102.0, 85.8, 82.2, 23.0; MS (APCI, pos) *m/z* (%): 307(29) [M + H]^+^; HRMS (CI^+^) calculated for C_19_H_14_O_4_ [M]^+^ 306.0892, found 306.0886.

(***E***)**-6,6’-**(**pent-1-en-4-yne-1,5-diyl**)**bis**(**2,2-dimethyl-4*H*-benzo(*d*)(1,3)dioxine**) (**1FF**): Prepared from **2FF** following the general procedure described for **1′AA** to afford 4 mg (40% yield) of **1FF** as a yellow oil: IR(ATR, CHCl_3_): cm^−1^ 2988, 2923, 2220, 1613, 1582, 1496, 1384, 1265, 1202, 1142, 1117, 1065, 956; ^1^H NMR (500 MHz, CD3Cl): δ 7.24 (dd, 1H, *J* = 8.5, 1.9 Hz), 7.20 (dd, 1H, *J* = 8.5, 1.9 Hz), 7.08 (s, 1H), 6.99 (s, 1H), 6.76 (t, 2H, *J* = 9.0 Hz), 6.59 (d, 1H, *J* = 15.7 Hz), 6.08 (dt, 1H, *J* = 15.7, 5.7 Hz), 4.83 (s, 2H), 4.81 (s, 2H), 3.30 (dd, 2H, *J* = 5.7, 1.7 Hz), 1.54 (s, 12H); ^13^C{^1^H} NMR (125 MHz, CD_3_COCD_3_): δ 151.0, 150.7, 131.6, 130.7, 129.7, 128.0, 126.0, 122.5, 122.4, 119.4, 119.3, 117.2, 117.2, 115.5, 99.9, 99.6, 85.3, 82.4, 60.9, 60.6, 29.7, 24.7, 22.9; MS (APCI, pos) *m/z* (%): 391(1) [M + H]^+^, 423(5) [M + MeOH + H]^+^; HRMS (APCI, pos) calculated for C_25_H_26_O_4_ [M + H]^+^ 391.1904, found 391.1898.

(***E***)**-**((**4-**(**5-**(**benzo(*d*)(1,3)dioxol-5-yl**)**pent-4-en-1-yn-1-yl**)**-1,2-phenylene**)**bis**(**oxy**))**bis**(***tert*-butyldimethylsilane**) (**1′EA**): Prepared from **2EA** following the general procedure described for **1′AA** to afford 52 mg (43% yield) of **1′EA** as a yellow oil. IR(ATR, neat): cm^−1^ 2929, 2857, 2193, 1593, 1557, 1505, 1488, 1407, 1308, 1250, 1099, 1038, 895, 828, 780; ^1^H NMR (500 MHz, CDCl_3_): δ 6.94–6.92 (m, 3H), 6.81 (dd, 1H, *J* = 8.0, 1.2 Hz), 6.76–6.74 (m, 2H), 6.60 (d, 1H, *J* = 15.7 Hz), 6.07 (dt, 1H, *J* = 15.7, 5.7 Hz), 5.95 (s, 2H), 3.31 (dd, 2H, *J* = 5.7, 1.5 Hz), 0.99 (s, 9H), 0.98 (s, 9H), 0.21 (s, 6H), 0.20 (s, 6H); ^13^C{^1^H} NMR (125 MHz, CDCl_3_): δ 147.9, 147.4, 146.9, 146.6, 131.7, 130.9, 125.3, 124.3, 122.8, 120.9, 120.7, 116.4, 108.2, 105.7, 101.0, 84.9, 82.6, 25.9, 22.9, 18.5, 18.4, −4.0, −4.1; (ESI, pos) *m/z* (%): 523(9) [M + H]+; HRMS (APCI, pos) calculated for C_30_H_40_O_4_Si_2_ [M + H]^+^ 521.2538, found 521.2532.

(***E***)**-5-**(**5-phenylpent-1-en-4-yn-1-yl**)**benzo(*d*)(1,3)dioxole** (**1EG**) [32]: Prepared from **2EG** following the general procedure described for **1′AA** to afford 12 mg (20% yield) of **1EG** as a yellow oil. IR(ATR, neat): cm^−1^ 2916, 2895, 2212, 1502, 1488, 1444, 1279, 1248, 1167, 1124, 1099, 1035, 952, 927, 756; ^1^H NMR (400 MHz, CD3Cl): δ 7.46–7.44 (m, 2H), 7.31–7.29 (m, 3H), 6.93 (d, 1H, *J* = 1.5 Hz), 6.82 (dd, 1H, *J* = 8.0, 1.5 Hz), 6.75 (d, 1H, *J* = 8.0 Hz), 6.61 (dd, 1H, *J* = 15.6, 1.5 Hz), 6.08 (dt, 1H, *J* = 15.6, 5.7 Hz), 5.95 (2, 2H), 3.34 (dd, 2H, *J* = 5.7, 1.7 Hz); ^13^C{^1^H} NMR (125 MHz, CDCl_3_): δ 148.0, 147.0, 131.6, 131.0, 128.2, 127.8, 123.7, 122.5, 120.8, 108.2, 105.7, 101.0, 86.8, 82.8, 22.9; (ESI, pos) *m/z* (%): 263(3) [M + H]^+^.

**General Procedure—Deprotection for Catechol-Containing Products: Rooperol** (**1AA**) [14]: In a round-bottomed flask containing 44 mg of the silylester **1′AA** (0.06 mmol) in 1 mL of dry DMF under Ar was placed 14 mg (0.24 mmol, 4 equivalent) of anhydrous KF. The flask was placed in an ice bath, and 0.2 mL of a solution prepared as a 1:100 dilution of 33% HBr/AcOH in DMF was added by syringe. After stirring for 1.5 h in the ice bath, the reaction mixture was diluted with EtOAc and washed with H_2_O and brine. The organic layer was dried over Na_2_SO_4_, filtered, and evaporated. The residue was subjected to flash chromatography 25% EtOAc/Hex + 1% MeOH) to afford 11 mg (65% yield) of catechol rooperol (**1AA**) as a yellow oil. ^1^H NMR (400 MHz, *d_6_*-acetone): δ 6.93 (1H, brs), 6.89 (1H, d, *J =* 1.7 Hz), 6.80 (1H, dd, *J =* 8.0, 1.8 Hz), 6.74–6.77 (3H, m), 6.55 (1H, dt, *J =* 15.7, 1.7 Hz), 6.05 (1H, dt, *J =* 15.7, 5.7 Hz), 3.25 (2H, dd, *J =* 5.7, 1.8 Hz). ^13^C NMR (100 MHz, *d*_6_-acetone) δ 145.6, 145.0, 144.8, 130.9, 129.4, 123.7, 121.5, 118.3, 118.1, 155.2, 115.1, 114.9, 112.6, 84.1, 82.6, 22.1. IR (thin film) 3019, 1666, 1514, 1388, 1215, 755. MS (ESI^-^) *m/z*: 281 [M -H]^–^, 100); HRMS (ESI^-^) calculated for C_17_H_13_O_4_ [M − H]^-^ 281.0819, found 281.0819.

(***E***)**-4-**(**5-**(**benzo(*d*)(1,3)dioxol-5-yl**)**pent-4-en-1-yn-1-yl**)**benzene-1,2-diol** (**1EA**): Prepared from **1′EA** following the general procedure described for **1AA** to afford 2 mg (18% yield) of **1EA** as a colorless oil. IR(ATR, neat): cm^−1^ 2919, 2850, 2220, 1599, 1518, 1445, 1294, 1249, 1196, 1110, 1033, 962, 920, 817, 768; ^1^H NMR (500 MHz, CD3Cl): δ 6.96–6.92 (m, 3H), 6.82–6.79 (m, 2H), 6.75 (d, 1H, *J* = 8.0 Hz), 6.59 (dt, 1H, *J* = 15.6, 1.6 Hz), 6.06 (dt, 1H, *J* = 15.6, 5.7 Hz), 5.95 (s, 2H), 5.30 (s, 1H), 5.18 (s, 1H), 3.30 (dd, 2H, *J* = 5.7, 1.7 Hz); ^13^C{^1^H} NMR (125 MHz, CD3COCD_3_): δ 147.9, 147.0, 143.8, 143.0, 131.7, 130.9, 125.3, 122.7, 120.8, 118.6, 116.3, 115.3, 108.2, 105.6, 101.0, 85.1, 82.3, 22.9; (ESI, pos) *m/z* (%): 295(4) [M + H]^+^; HRMS (APCI, pos) calculated for C_18_H_14_O_4_ [M + H]^+^ 295.0965, found 295.0962.

**General Procedure—Deprotection for Phenol- and Resorcinol-Containing Products:** (***E***)**-4,4’-**(**pent-1-en-4-yne-1,5-diyl**)**diphenol** (**1BB**) [33]: To a solution of 10 mg (0.019 mmol) of **1′BB** in 0.2 mL of freshly distilled THF was added 4.7 µL (0.082 mmol) of AcOH. The solution was cooled in an ice bath, and 82 µL (0.082 mmol) of a 1M solution of TBAF in THF was added. After 20 min, EtOAc (5 mL) and water (5 mL) were added to the reaction mixture. The layers separated, and the aqueous layer was extracted 1 x EtOAc (5 mL). The combined organic layers were washed 1 x brine (5 mL). The combined aqueous layers were then back-extracted 1 x DCM (10 mL). The combined organics layers were dried over Na_2_SO_4_, filtered, evaporated, and subjected to flash chromatography 50% EtOAc/hexanes to afford 4 mg (71%) of **1BB** as a colorless oil. IR(ATR, neat): cm^−1^ 2955, 2851, 2224, 1602, 1506, 1238, 965, 831; ^1^H NMR (500 MHz, CD3Cl): δ 7.25–7.22 (m, 4H), 6.73–6.71 (m, 4H), 6.58 (d, 1H, *J* = 15.7 Hz), 6.07 (dt, 1H, *J* = 15.7, 5.7 Hz), 3.26 (dd, 2H, *J* = 5.7, 1.7 Hz); ^13^C{^1^H} NMR (125 MHz, CDCl_3_): δ 158.5, 158.0, 133.9, 131.9, 130.3, 128.4, 122.7, 116.3, 116.2, 116.0, 85.5, 83.6, 23.3; MS (ESI, neg) *m/z* (%): 249(100) [M − H]^–^.

(***E***)**-5,5’-**(**pent-1-en-4-yne-1,5-diyl**)**bis**(**benzene-1,3-diol**) (**1CC**): Prepared from **1′CC** following the general procedure described for **1BB** to afford 10 mg (42% yield) of **1CC** as a colorless oil. IR(ATR, neat): cm^−1^ 2960, 2925, 2853, 2220, 1587, 1503, 1441, 1340, 1299, 1142,997, 837; ^1^H NMR (500 MHz, CD3COCD_3_): δ 8.34 (s, 2H), 8.14 (s, 2H), 6.58 (dt, 1H, *J* = 15.7, 1.8 Hz), 6.44 (d, 2H, *J* = 2.2 Hz), 6.43 (d, 2H, *J* = 2.2 Hz), 6.35 (t, 1H, *J* = 2.2 Hz), 6.26 (t, 1H, *J* = 2.2 Hz), 6.20 (dt, 1H, *J* = 15.7, 5.6 Hz), 3.31 (dd, 2H, *J* = 5.6, 1.8 Hz); ^13^C{^1^H} NMR (125 MHz, CD3COCD_3_): δ 159.6, 159.4, 140.1, 132.2, 125.9, 125.0, 110.8, 105.7, 104.0, 102.8, 86.6, 83.7, 23.0; (ESI, pos) *m/z* (%): 283(6) [M + H]^+^; HRMS (APCI, pos) calculated for C_17_H_14_O_4_ [M + H]^+^ 283.0965, found 283.0965.

**Cancer Cell Cytotoxicity Assays.** HeLa, H460, and A549 cells were obtained from ATCC and grown in RPMI culture medium supplemented with 10% heat inactivated FBS (Life Technologies code 10270106). Cell culture media were supplemented with 4 mM glutamine (Lonza code BE17–605E), 100 μg/mL gentamicin (Lonza code 17-5182), and P/S (200 units/mL and 200 μg/mL) (Lonza code 17-602E) at 37 °C with 5% CO_2_. The effect of the investigated compounds on cell proliferation was determined by MTT (3-(4,5-dimethylthiazol-2-yl)-2,5-diphenyltetrazolium bromide) assay. The compounds were dissolved in DMSO at a concentration of either 10 or 50 mM prior to cell treatment. The cells were trypsinized and seeded at various cell concentrations depending on the cell type. The cells were grown for 24–72 h, treated with test compounds at required concentrations, and incubated for 72 h in 100 or 200 μL media depending on the cell line used. Three replicates were performed. Cells treated with 0.1% DMSO were used as a negative control. The GI_50_ corresponds to the concentration of the compound of interest that reduces by 50% the growth of the cancer cell line of interest after having cultured it for 72 h in the presence of the compound in comparison to the untreated control condition.

## Figures and Tables

**Table 1:** Cancer Cell Cytotoxicity of Rooperol and Analogues.

Compound	HeLa (GI_50_, µM)	H460 (GI_50_, µM)	A549 (GI_50_, µM)
Rooperol (**1AA**)	18 ± 2	18.8 ± 0.7	26.0 ± 0.1
**1BB**	33.2 ± 0.7	*nd*	*nd*
**1CC**	77 ± 10	*nd*	*nd*
**1DD**	>500	221.8 ± 0.1	291.7 ± 0.1
**1EE**	112 ± 3	61.5 ± 0.1	28.4 ± 0.1
**1FF**	217 ± 16	*nd*	*nd*
**1EA**	38 ± 8	*nd*	*nd*
**1EG**	77± 12	*nd*	*nd*

## Supplementary Materials

The following supporting information can be downloaded at: https://www.mdpi.com/article/10.3390/molecules27061792/s1, Supporting Figures: NMR spectra of all compounds.
