# Supplementary material for: Scalable Synthesis and Cancer Cell Cytotoxicity of Rooperol and Analogues
Source: Molecules. 2022 Mar 9;27(6):1792. doi: 10.3390/molecules27061792 (PMC8949049; doi:10.3390/molecules27061792)
Supplement: Supplementary file 1 [file molecules-27-01792-s001.zip › molecules-1536112-supplementary.pdf]

Supporting Information

Scalable Synthesis and Cancer Cell Cytotoxicity of Rooperol and Analogues.

Zachary T. Schwartz[1§], Peter D. Theisen[1‡§], Olaf T. Bjornstahl[2], Mary Rodebaugh[1], Mauricio A. Jemal[1,3], Dallas Lee[1], Spencer D. Shelton[1], Zhenze Zhao[1], Liqin Du[1], and Sean M. Kerwin [1,2,3*]

[1] Dept. of Chemistry & Biochemistry, Texas State University, San Marcos, TX 78666
[2] Division of Chemical Biology and Medicinal Chemistry, University of Texas at Austin, Austin, TX, 78712
[3] Materials Science, Engineering, and Commercialization Program, Texas State University, San Marcos, TX, 78666

‡ Deceased August 27, 2018
§ These authors made equal contributions to the work.
* Correspondence: smk89@txstate.edu; Tel.: +1 5122452056

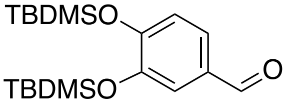
TBDMSO
TBDMSO
O

8A

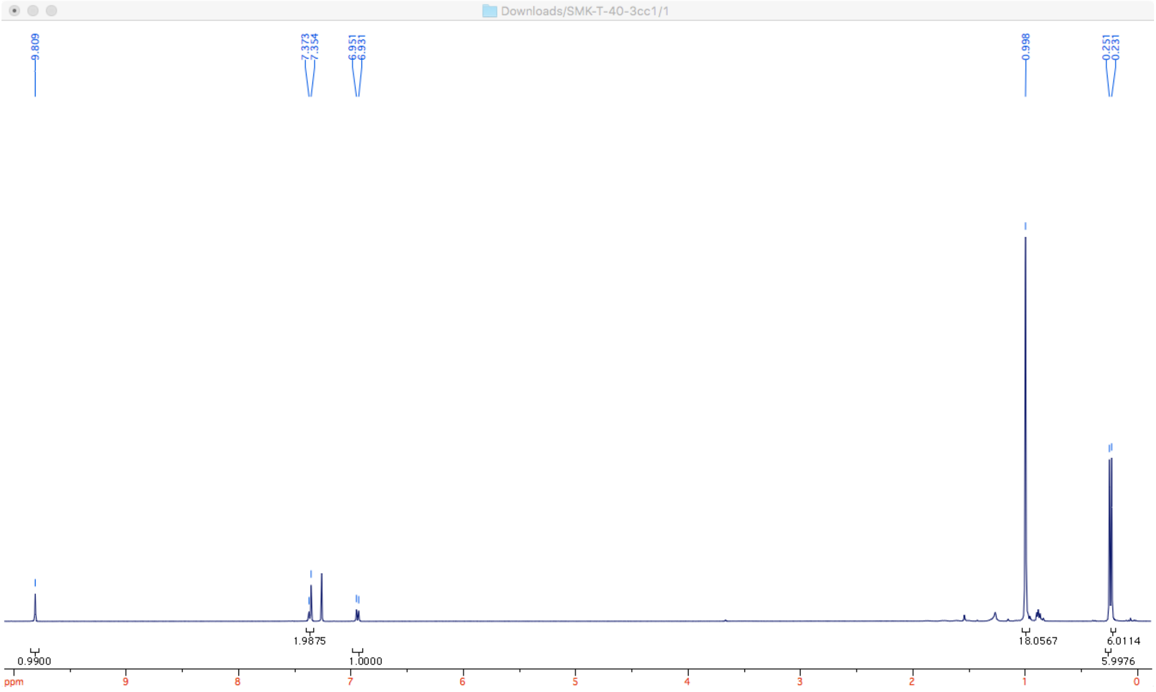
Downloads/SMK-T-40-3cc1/1
9.809
7.373
7.354
6.951
6.931
0.998
0.251
0.231
0.9900
1.9875
1.0000
18.0567
6.0114
5.9976
ppm
9
8
7
6
5
4
3
2
1
0

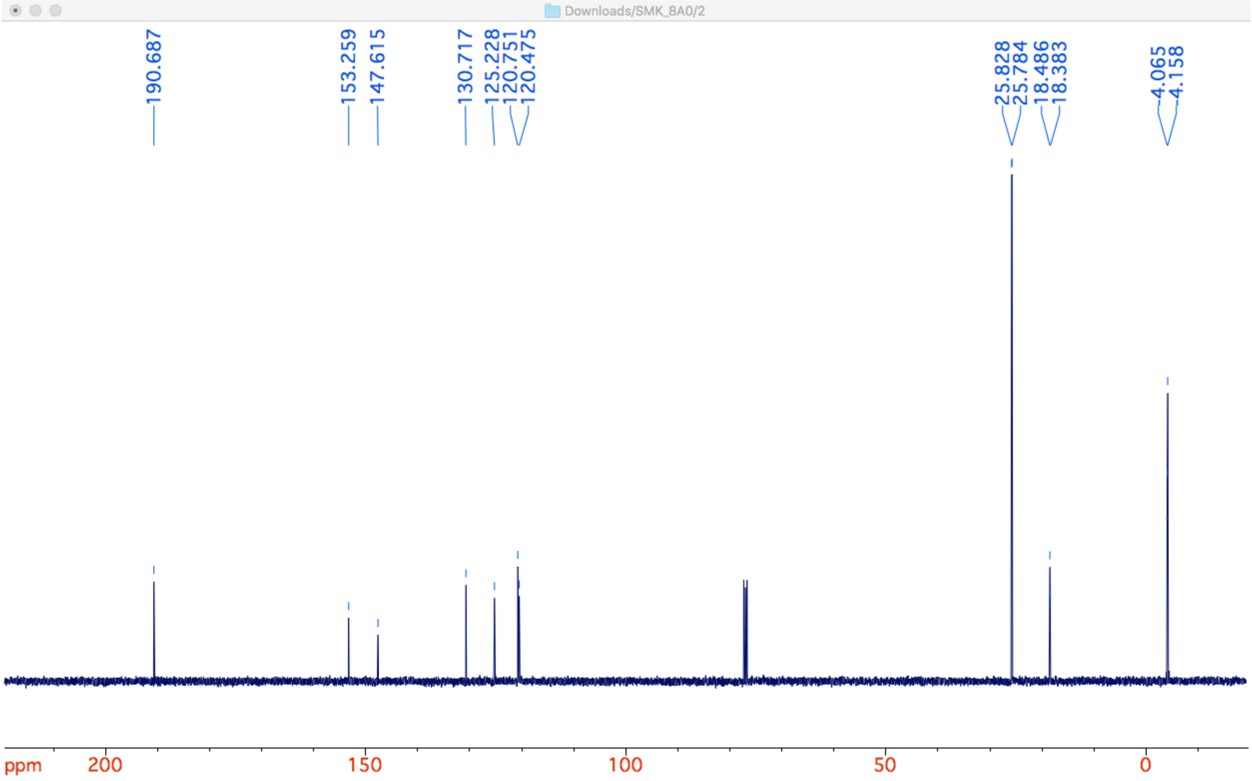
Downloads/SMK_8A0/2
190.687
153.259
147.615
130.717
125.228
120.751
120.475
25.828
25.784
18.486
18.383
-4.065
-4.158
ppm
200
150
100
50
0

Spectrum RT 1.07 - 1.25 (15 scans) - Background Subtracted 0.00 - 1.07
SMK-8A0 2021.11.12 14:15:33 ;
Intensity APCI + Max: 1.8E9

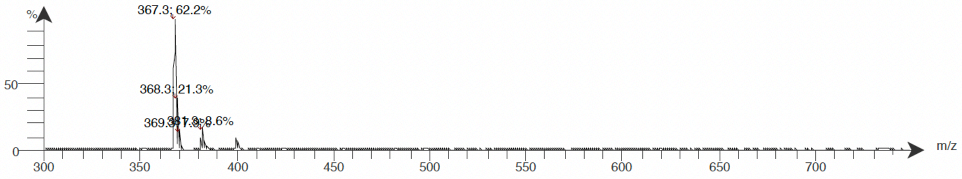

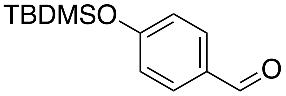
TBDMSO
O

8B

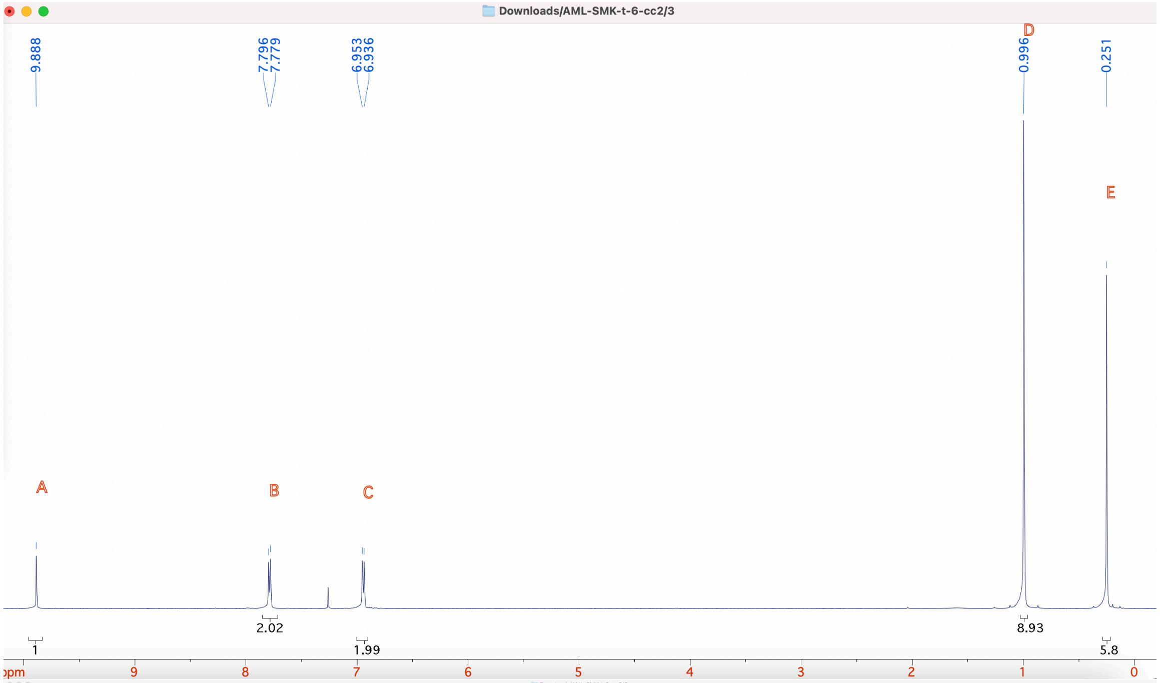
Downloads/AML-SMK-t-6-cc2/3
9.888
7.796
7.779
6.953
6.936
0.996
0.251
A
B
C
D
E
2.02
8.93
1
1.99
5.8
ppm
9
8
7
6
5
4
3
2
1
0

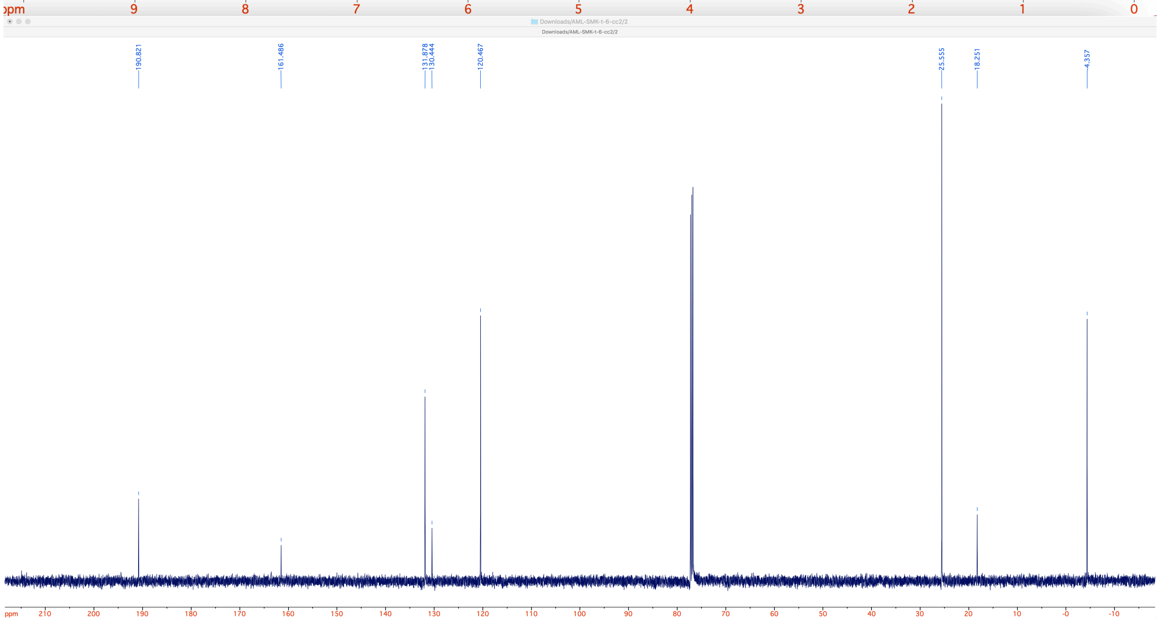
190.821
161.486
131.878
130.444
120.467
25.555
18.251
-4.357
ppm
210
200
190
180
170
160
150
140
130
120
110
100
90
80
70
60
50
40
30
20
10
-0
-10

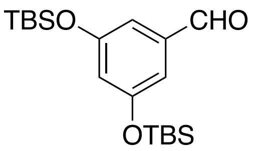
TBSO
CHO
OTBS

8C

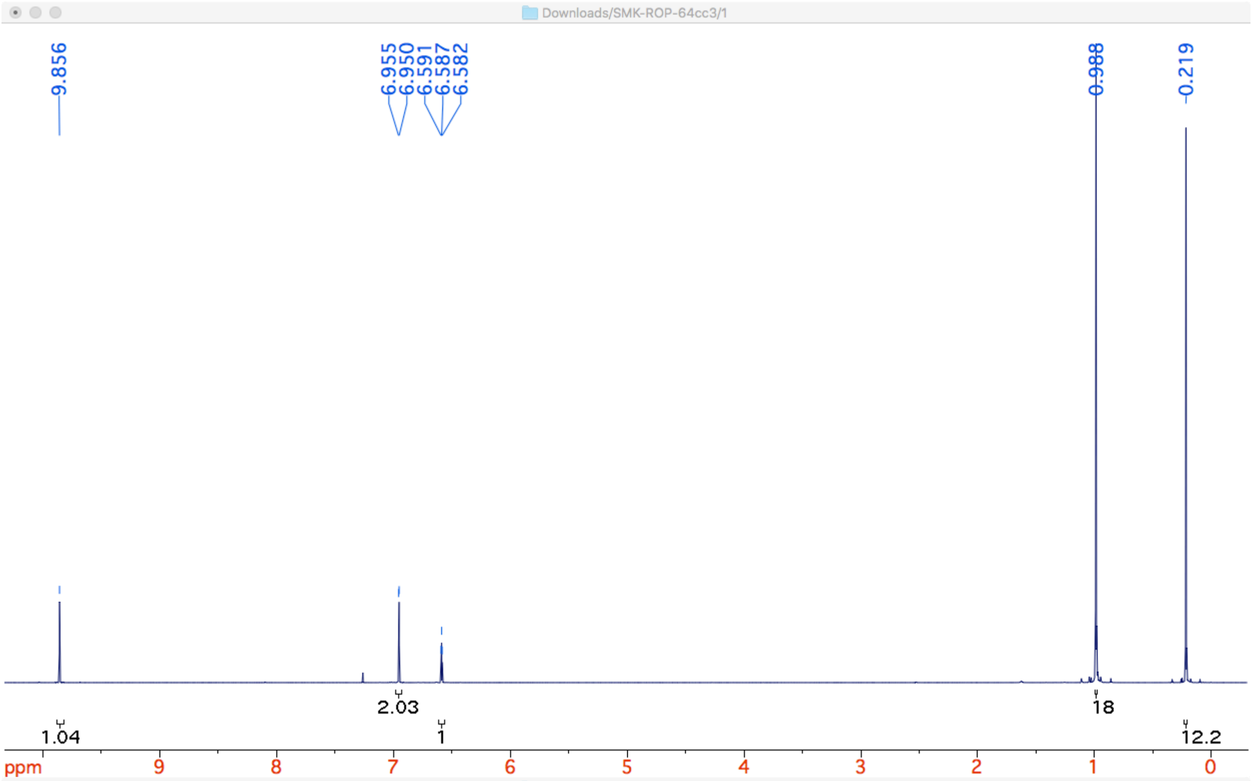
Downloads/SMK-ROP-64cc3/1
9.856
6.955
6.950
6.591
6.587
6.582
0.988
0.219
1.04
2.03
1
18
12.2
ppm
9
8
7
6
5
4
3
2
1
0

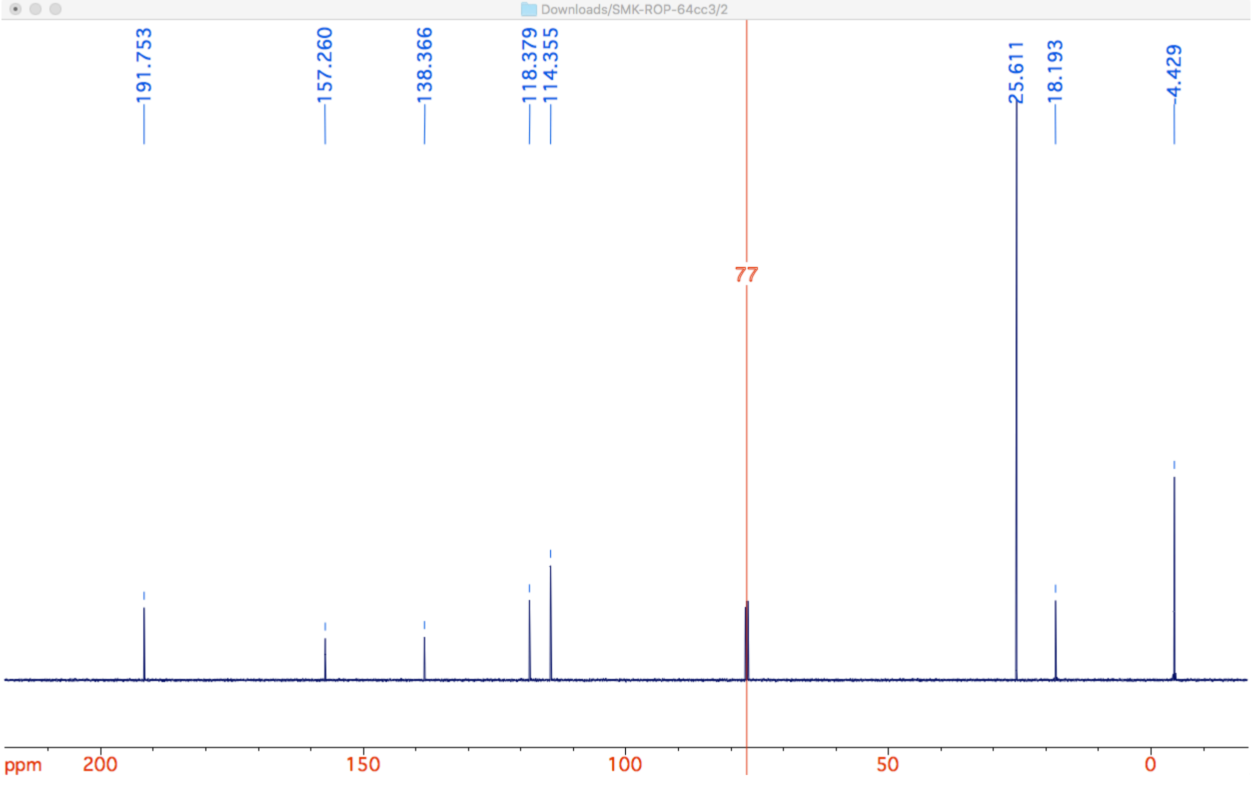
Downloads/SMK-ROP-64cc3/2
191.753
157.260
138.366
118.379
114.355
77
25.611
18.193
-4.429
ppm
200
150
100
50
0

Spectrum RT 1.08 - 1.26 (13 scans) - Background Subtracted 0.00 - 1.08
SMK-ROP-64-8A4_APCI_inj1 2021.11.12 15:53:49 ;
APCI + Max: 1.6E9

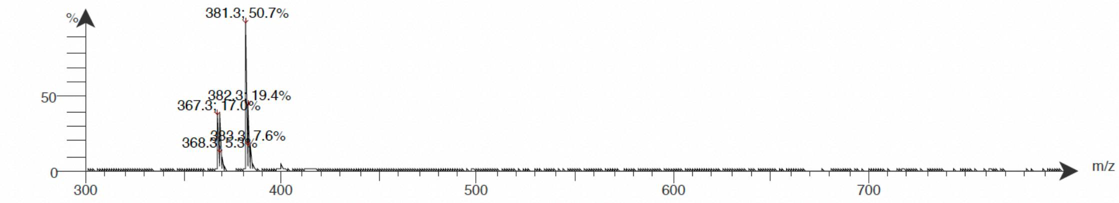

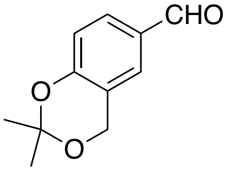
CHO
O
O

8F

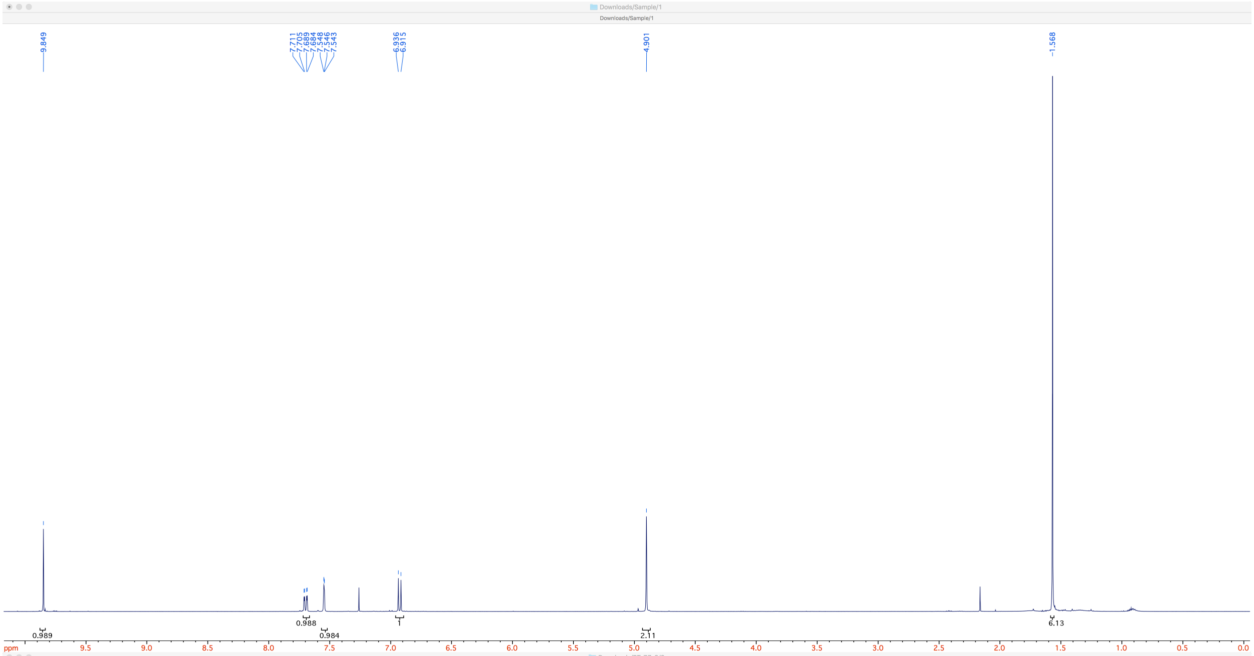
Downloads/Sample/1
9.849
7.711
7.705
7.689
7.684
7.548
7.546
7.543
6.936
6.915
4.901
1.568
0.989
0.988
0.984
1
2.11
6.13
ppm
9.5
9.0
8.5
8.0
7.5
7.0
6.5
6.0
5.5
5.0
4.5
4.0
3.5
3.0
2.5
2.0
1.5
1.0
0.5
0.0

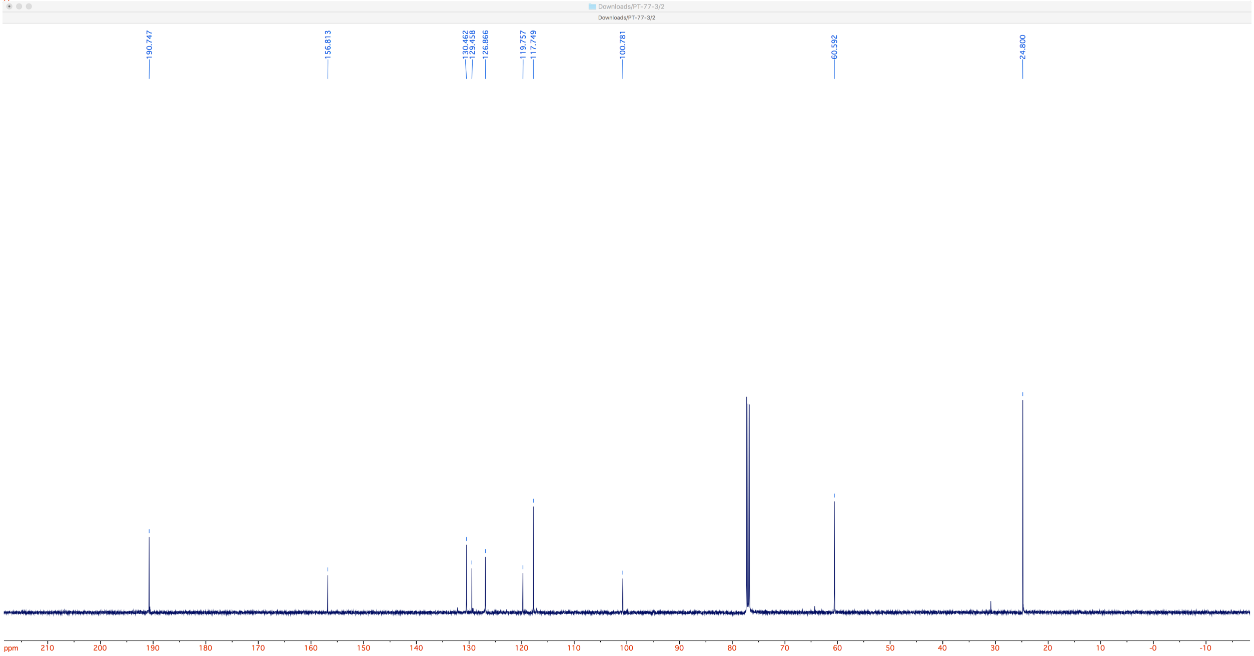
Downloads/PT-77-3/2
190.747
156.813
130.462
129.458
126.866
119.757
117.749
100.781
60.592
24.800
ppm
210
200
190
180
170
160
150
140
130
120
110
100
90
80
70
60
50
40
30
20
10
0
-10

Background RT 1.06 - 1.22 {29 scans}
PT-83-4_8C4_inj3.datx 2021.10.29 11:46:57 ;
ESI + Max: 2E8

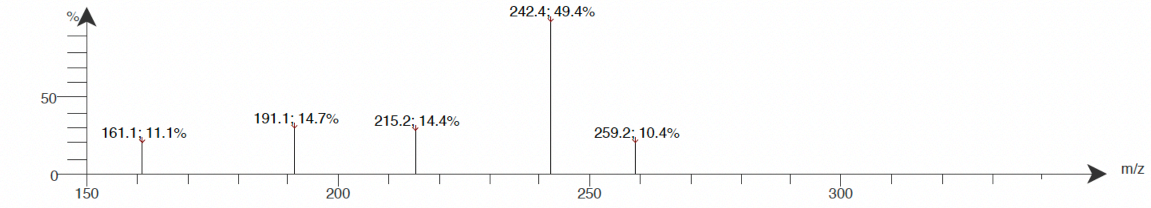

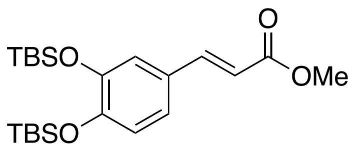
TBSO
TBSO
O
OMe

4A

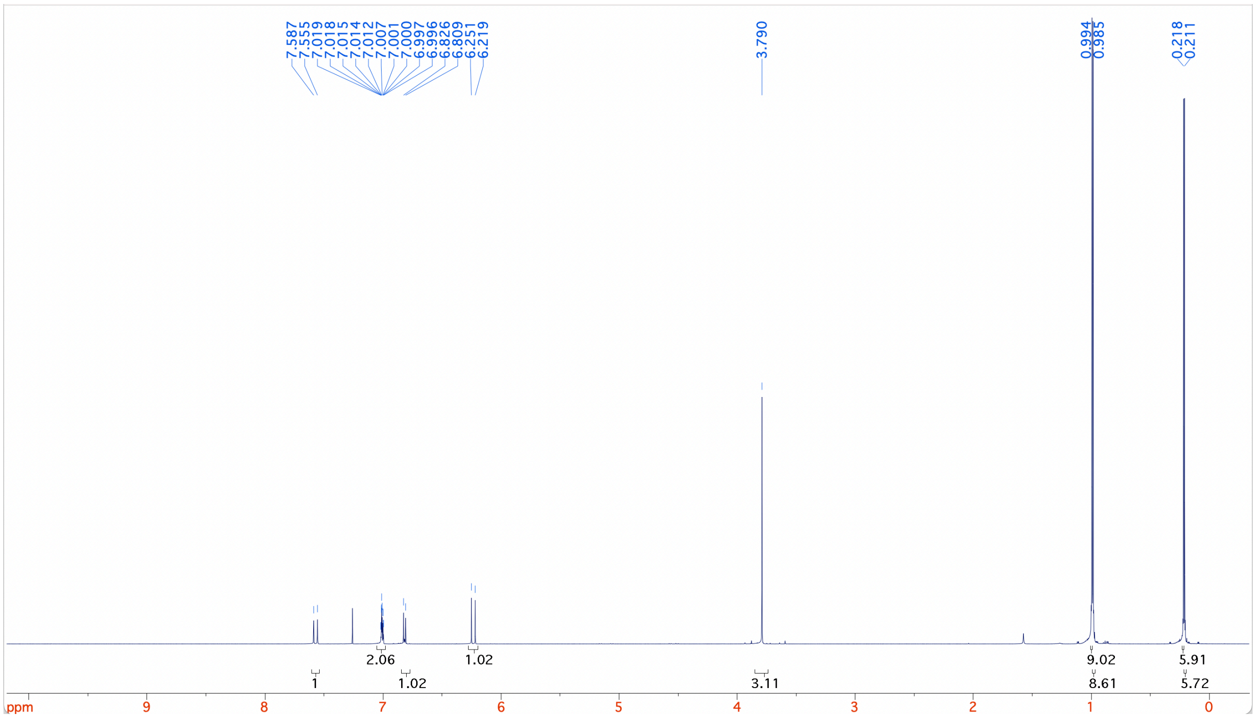
7.587
7.555
7.019
7.018
7.015
7.014
7.012
7.007
7.001
7.000
6.997
6.996
6.826
6.809
6.251
6.219
3.790
0.994
0.985
0.218
0.211
2.06
1.02
1
1.02
3.11
9.02
8.61
5.91
5.72
ppm
9
8
7
6
5
4
3
2
1
0

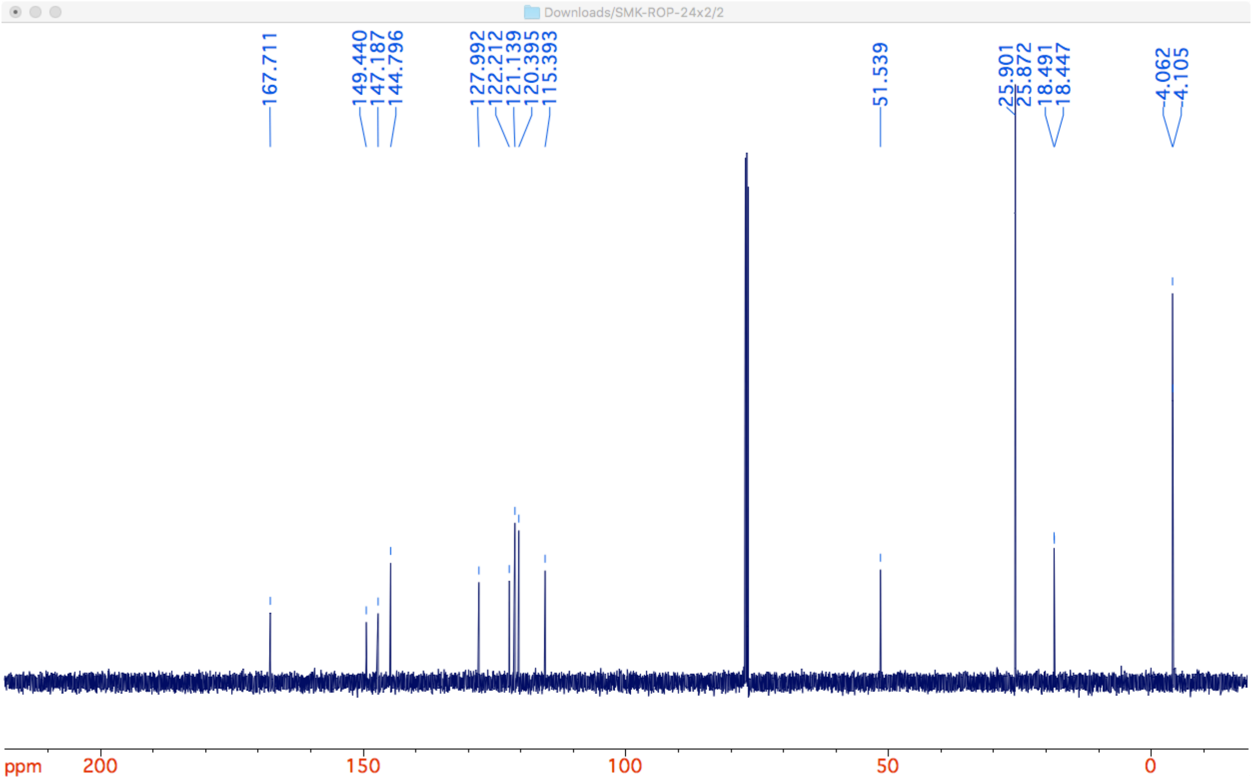
Downloads/SMK-ROP-24x2/2
167.711
149.440
147.187
144.796
127.992
122.212
121.139
120.395
115.393
51.539
25.901
25.872
18.491
18.447
-4.062
-4.105
ppm
200
150
100
50
0

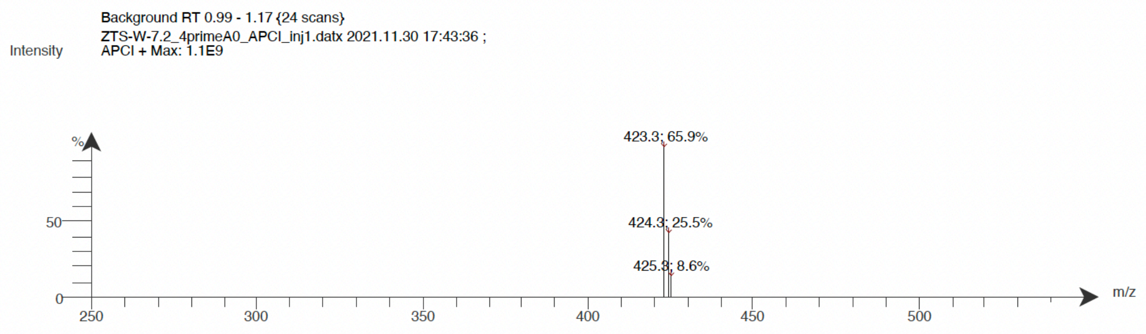
Background RT 0.99 - 1.17 {24 scans}
ZTS-W-7.2_4primeA0_APCI_inj1.datx 2021.11.30 17:43:36 ;
Intensity
APCI + Max: 1.1E9
%
50
0
423.3; 65.9%
424.3; 25.5%
425.3; 8.6%
250
300
350
400
450
500
m/z

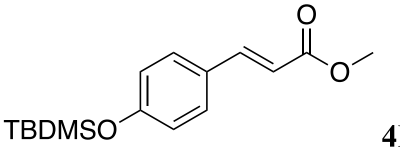
TBDMSO
O
O

4B

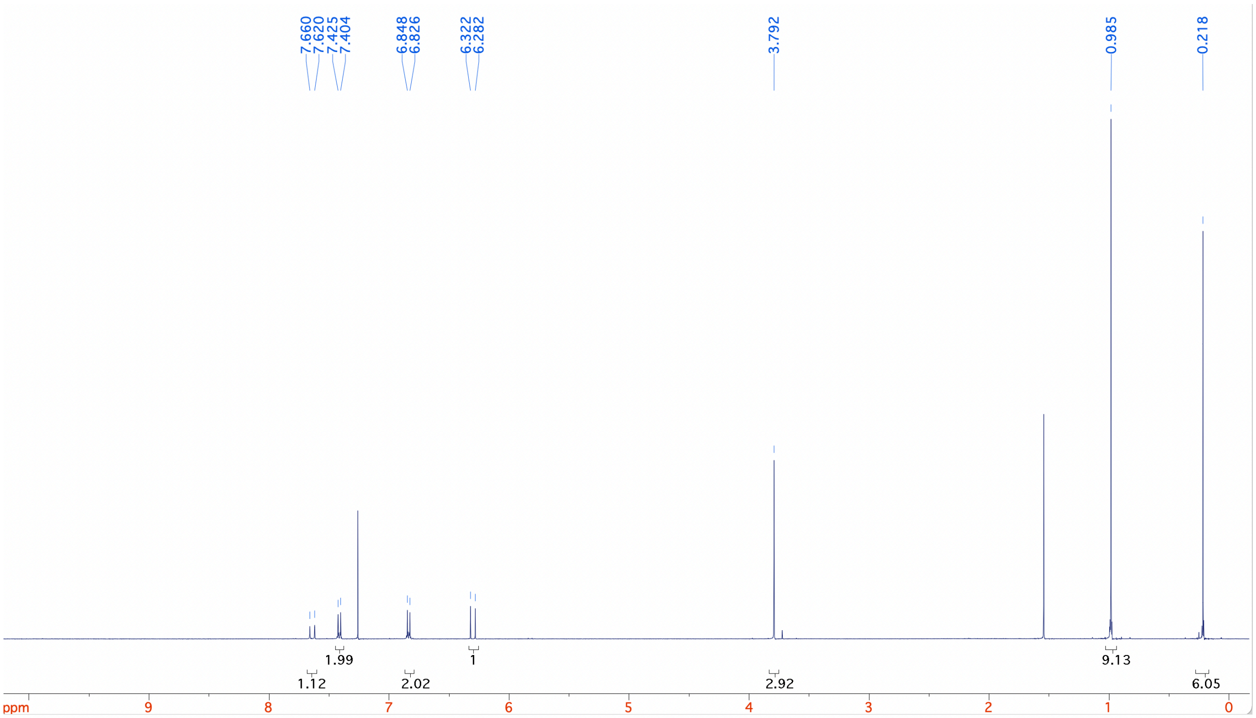
7.660
7.620
7.425
7.404
6.848
6.826
6.322
6.282
3.792
0.985
0.218
1.99
1
9.13
1.12
2.02
2.92
6.05
ppm
9
8
7
6
5
4
3
2
1
0

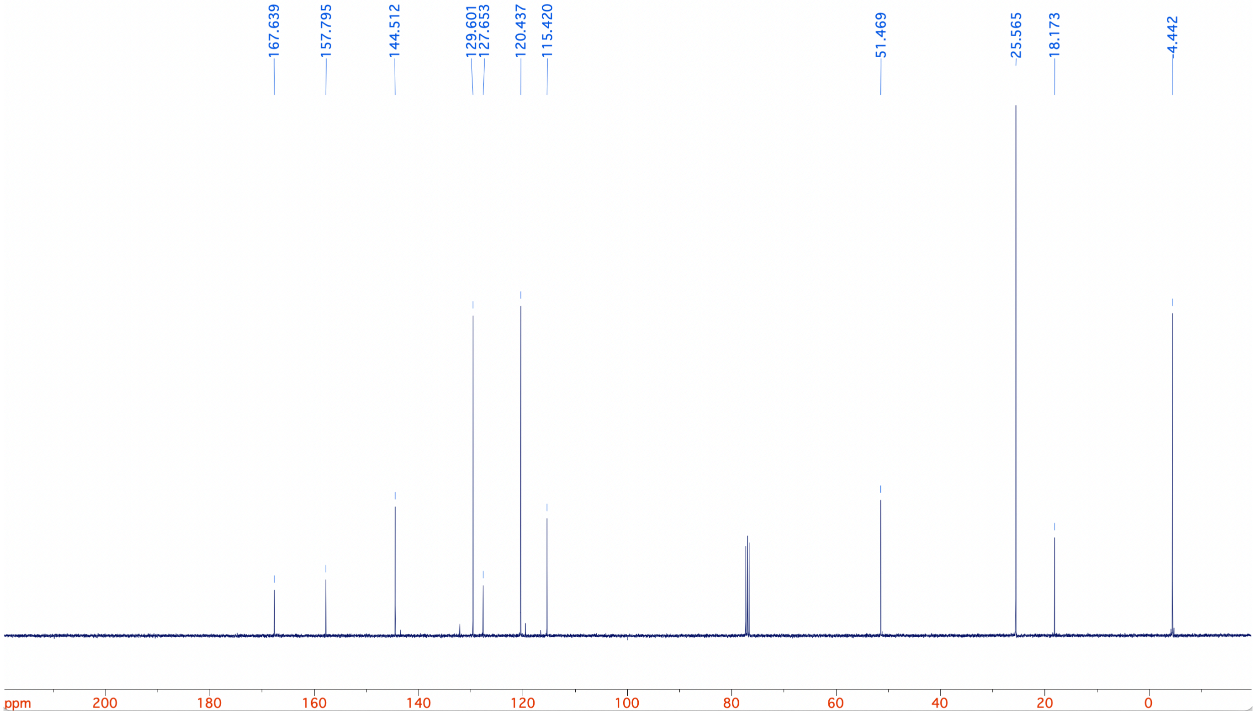
167.639
157.795
144.512
129.601
127.653
120.437
115.420
51.469
25.565
18.173
-4.442
ppm
200
180
160
140
120
100
80
60
40
20
0

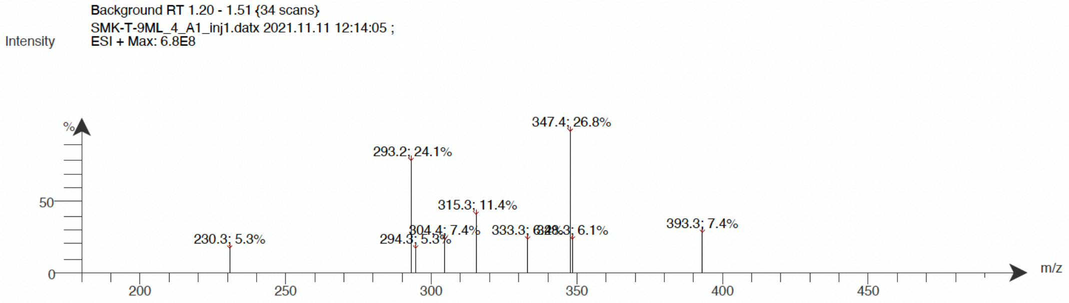
Background RT 1.20 - 1.51 (34 scans)
SMK-T-9ML_4_A1_inj1.datx 2021.11.11 12:14:05 ;
Intensity
ESI + Max: 6.8E8
%
50
0
347.4; 26.8%
293.2; 24.1%
315.3; 11.4%
230.3; 5.3%
294.3; 5.3%
304.4; 7.4%
333.3;
348.3; 6.1%
393.3; 7.4%
200
250
300
350
400
450
m/z

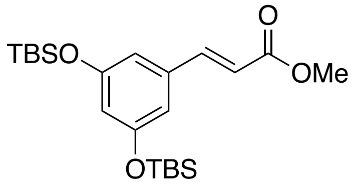

4C

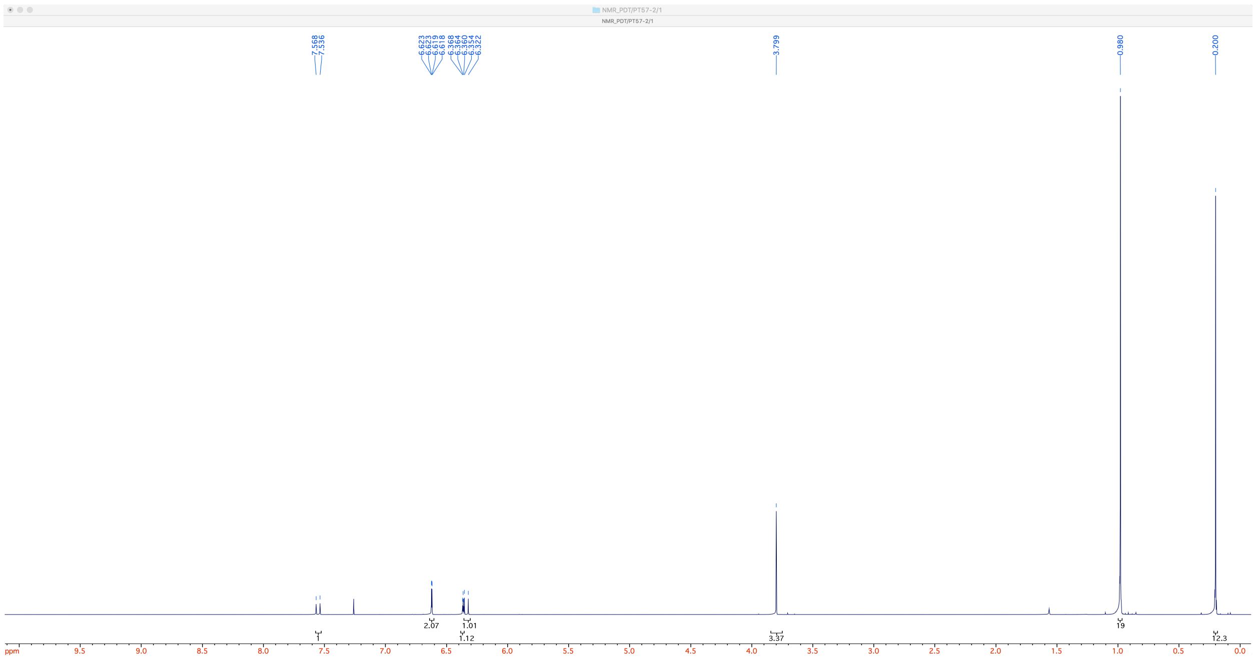

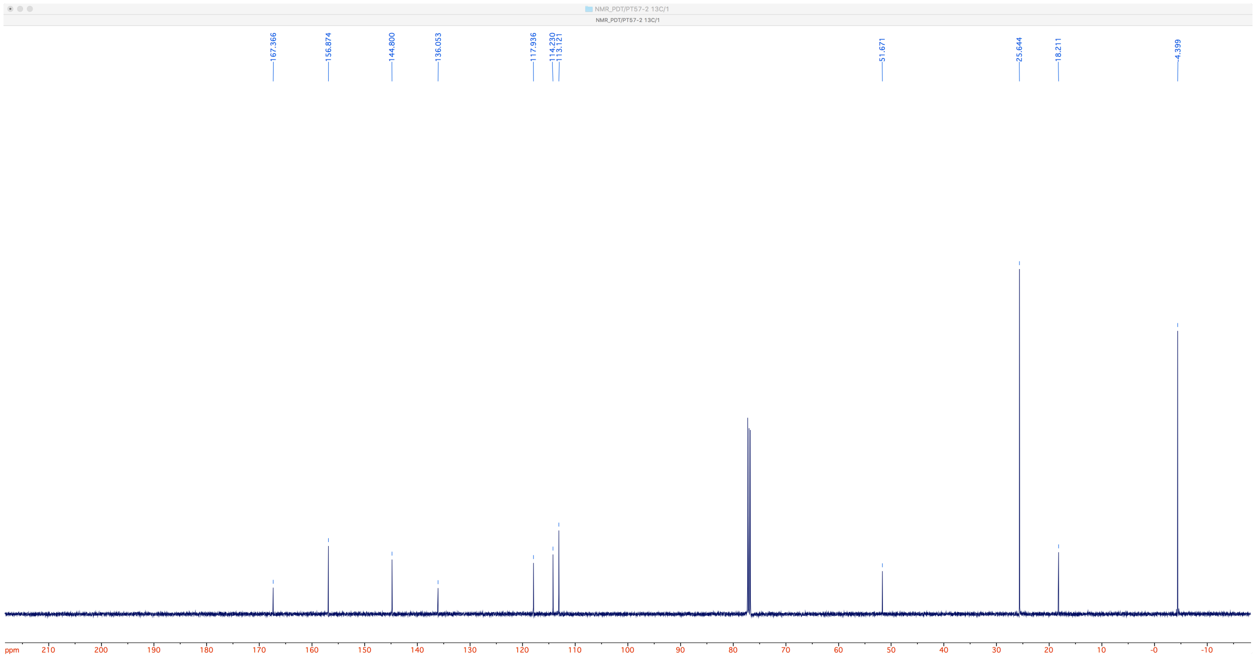

Spectrum RT 1.07 - 1.30 {17 scans} - Background Subtracted 0.00 - 1.07
KeMIS_188A_ACPI_inj1 2021.11.12 13:59:30 ;
Intensity APCI + Max: 1.9E9

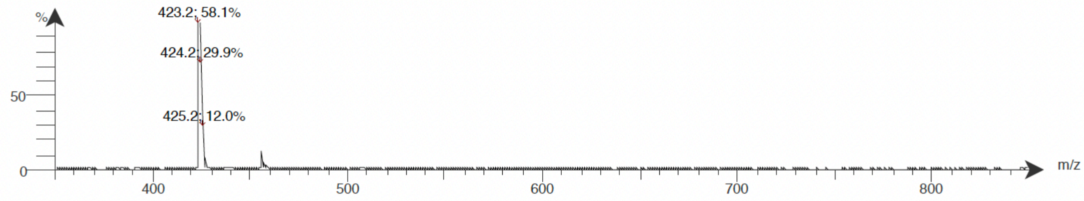

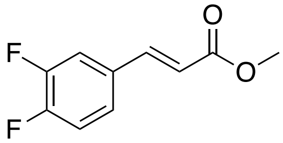

4D

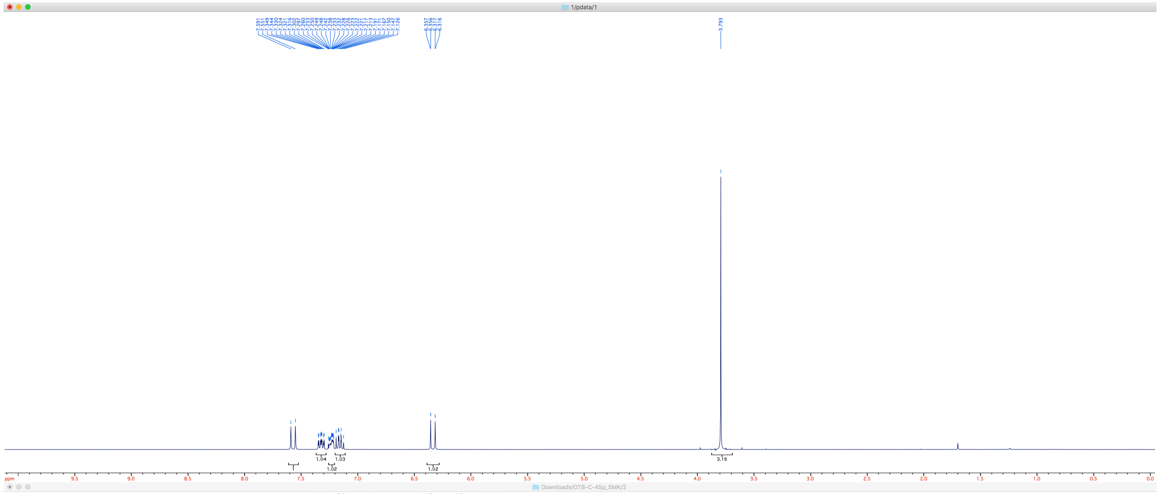

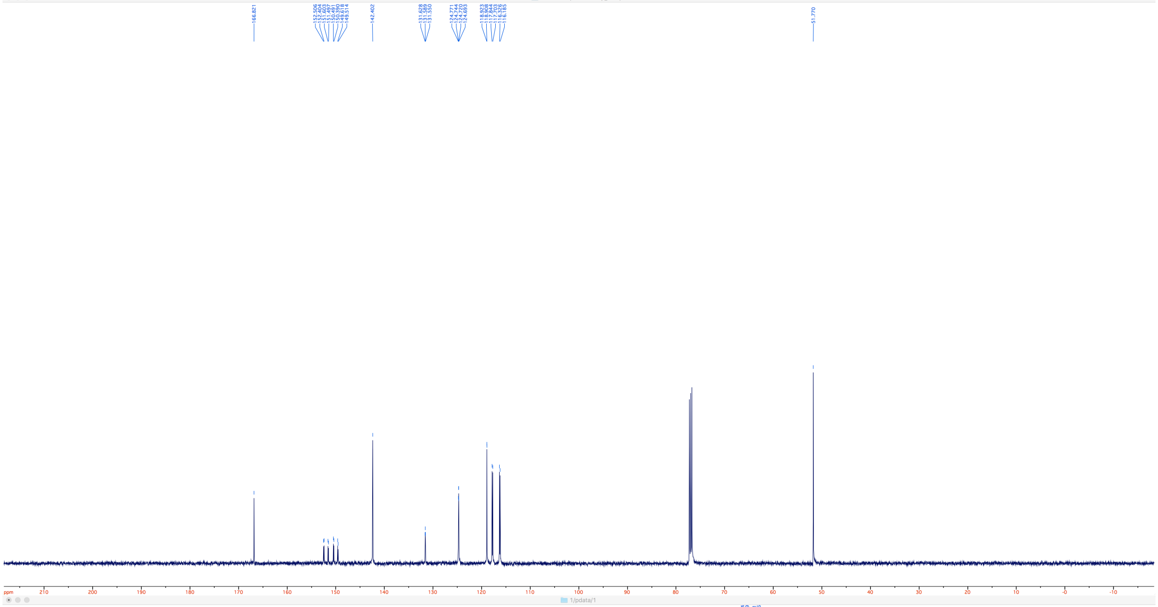

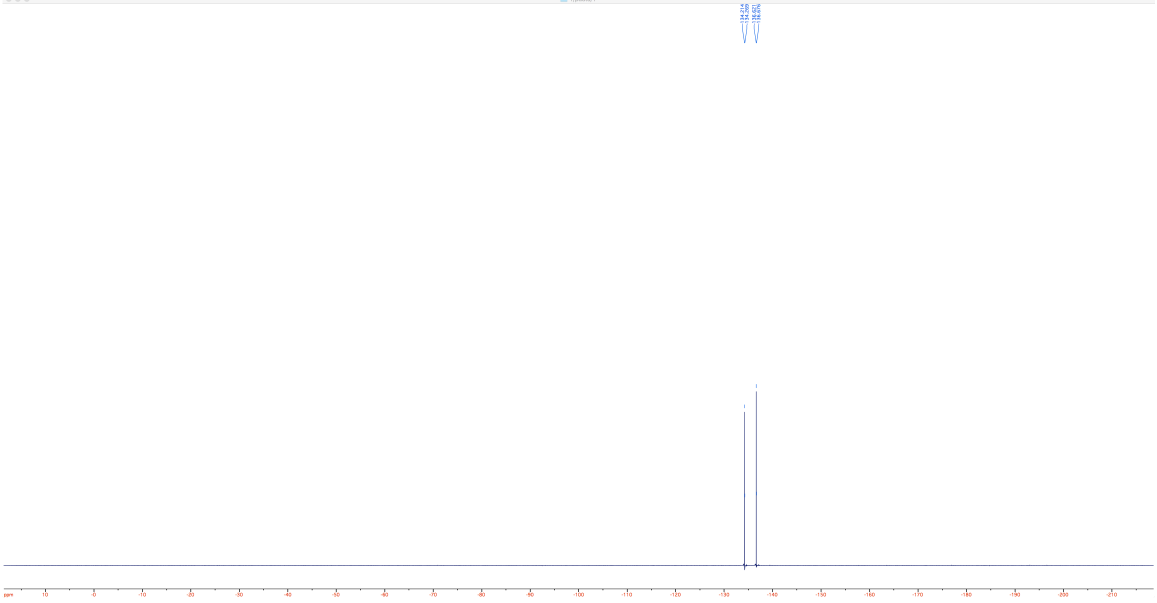

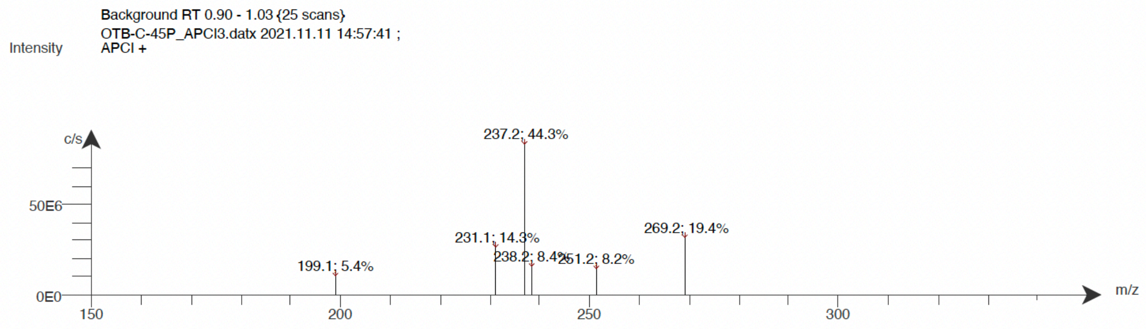

Background RT 0.90 - 1.03 {25 scans}
OTB-C-45P_APCI3.datx 2021.11.11 14:57:41 ;
APCI +
Intensity
c/s
50E6
0E0
237.2; 44.3%
231.1; 14.3%
238.2; 8.4%
251.2; 8.2%
269.2; 19.4%
199.1; 5.4%
150
200
250
300
m/z

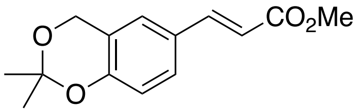
CO2Me
O
O

4F

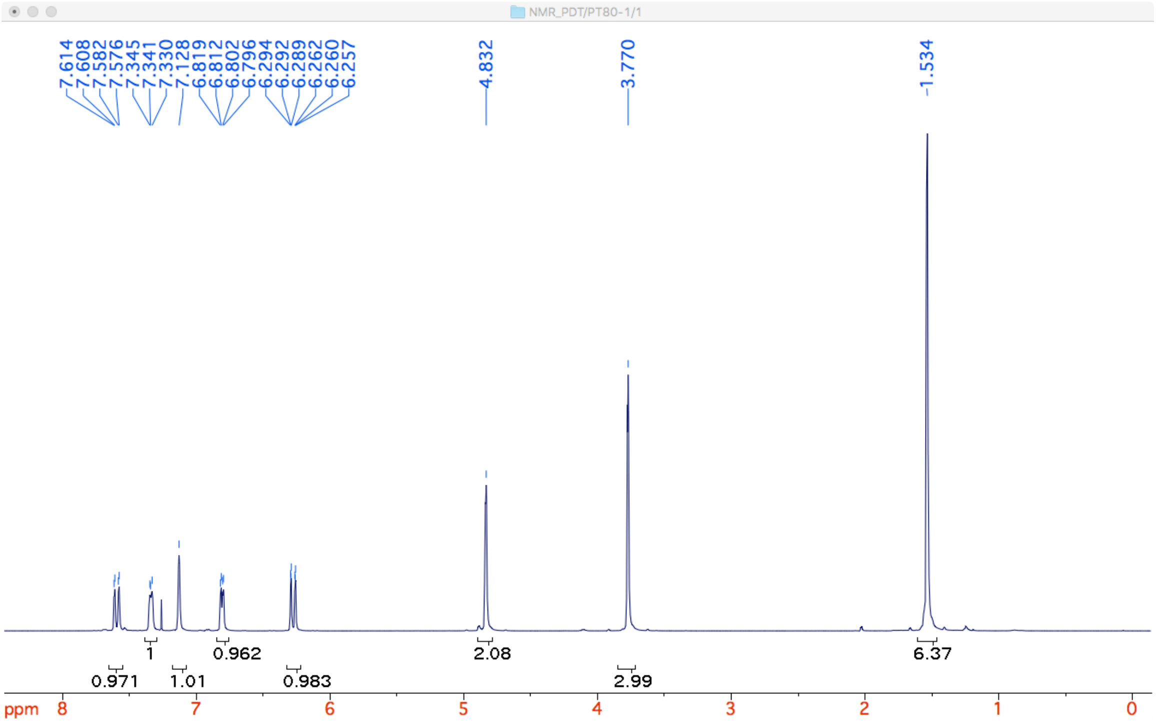
NMR_PDT/PT80-1/1
7.614 7.608 7.582 7.576 7.345 7.341 7.330 7.128 6.819 6.812 6.802 6.796 6.294 6.292 6.289 6.262 6.260 6.257
4.832
3.770
1.534
0.971 1 1.01 0.962 0.983 2.08 2.99 6.37
ppm 8 7 6 5 4 3 2 1 0

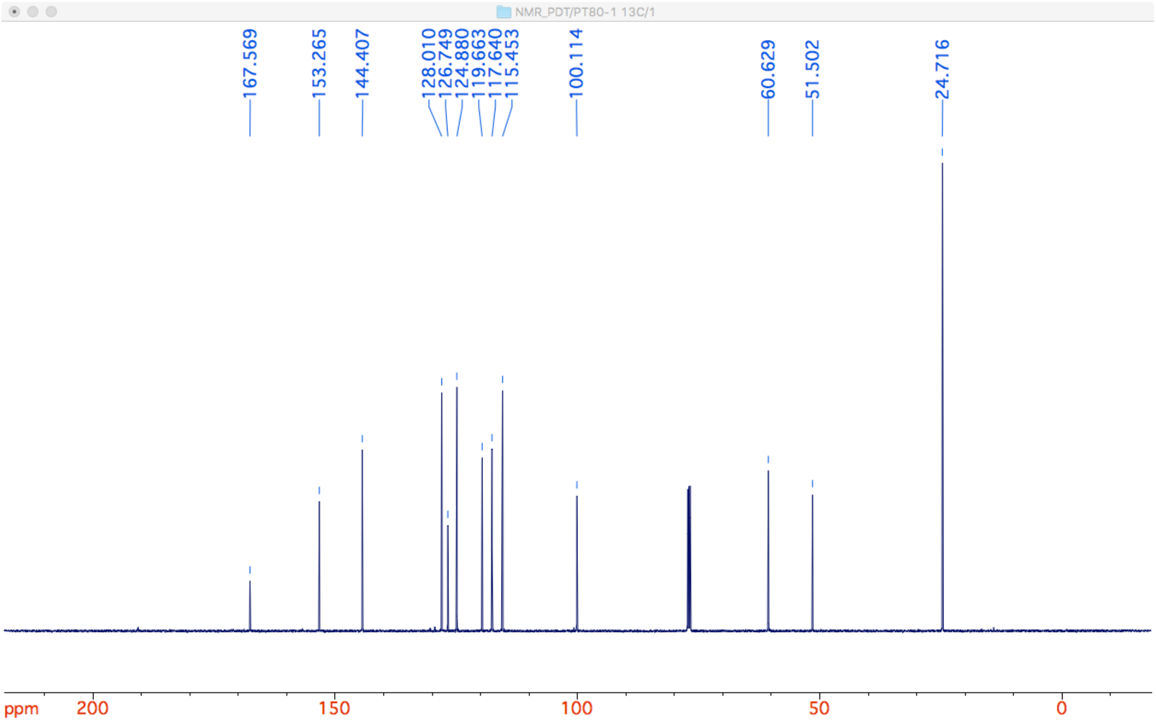
NMR_PDT/PT80-1 13C/1
167.569
153.265
144.407
128.010 126.749 124.880 119.663 117.640 115.453
100.114
60.629
51.502
24.716
ppm 200 150 100 50 0

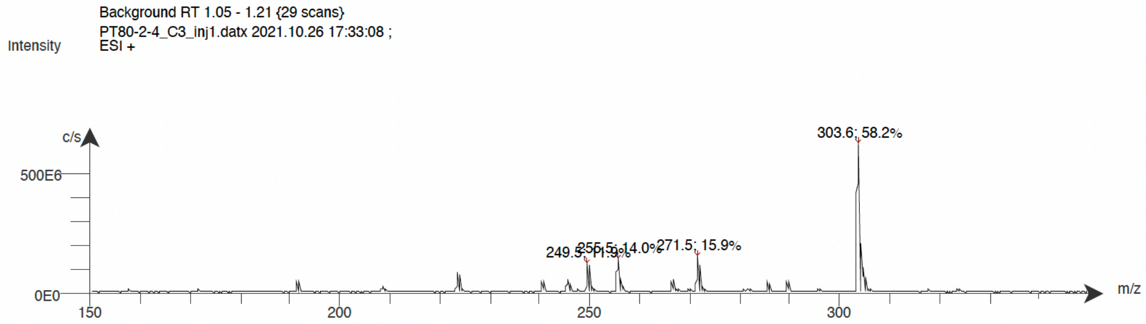

Background RT 1.05 - 1.21 {29 scans}
PT80-2-4_C3_inj1.datx 2021.10.26 17:33:08 ;
ESI +
Intensity
c/s
500E6
0E0
150
200
250
300
m/z
303.6; 58.2%
271.5; 15.9%
255.5; 14.0%

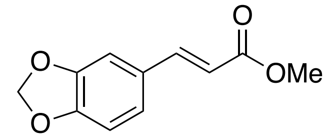

4E

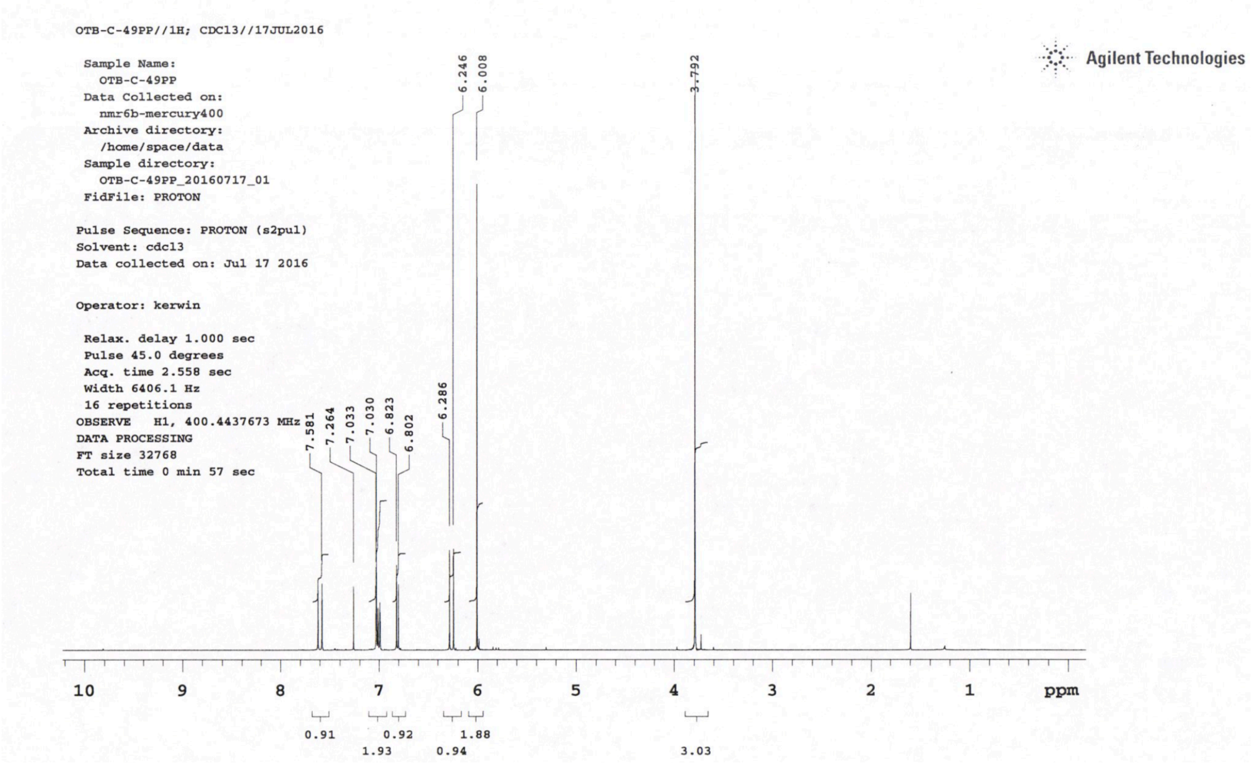
OTB-C-49PP//1H; CDCl3//17JUL2016
Sample Name:
OTB-C-49PP
Data Collected on:
nmr6b-mercury400
Archive directory:
/home/space/data
Sample directory:
OTB-C-49PP_20160717_01
FidFile: PROTON
Pulse Sequence: PROTON (s2pul)
Solvent: cdcl3
Data collected on: Jul 17 2016
Operator: kerwin
Relax. delay 1.000 sec
Pulse 45.0 degrees
Acq. time 2.558 sec
Width 6406.1 Hz
16 repetitions
OBSERVE H1, 400.4437673 MHz
DATA PROCESSING
FT size 32768
Total time 0 min 57 sec
Agilent Technologies
7.581
7.264
7.033
7.030
6.823
6.802
6.286
6.246
6.008
3.792
10
9
8
7
6
5
4
3
2
1
ppm
0.91
1.93
0.92
0.94
1.88
3.03

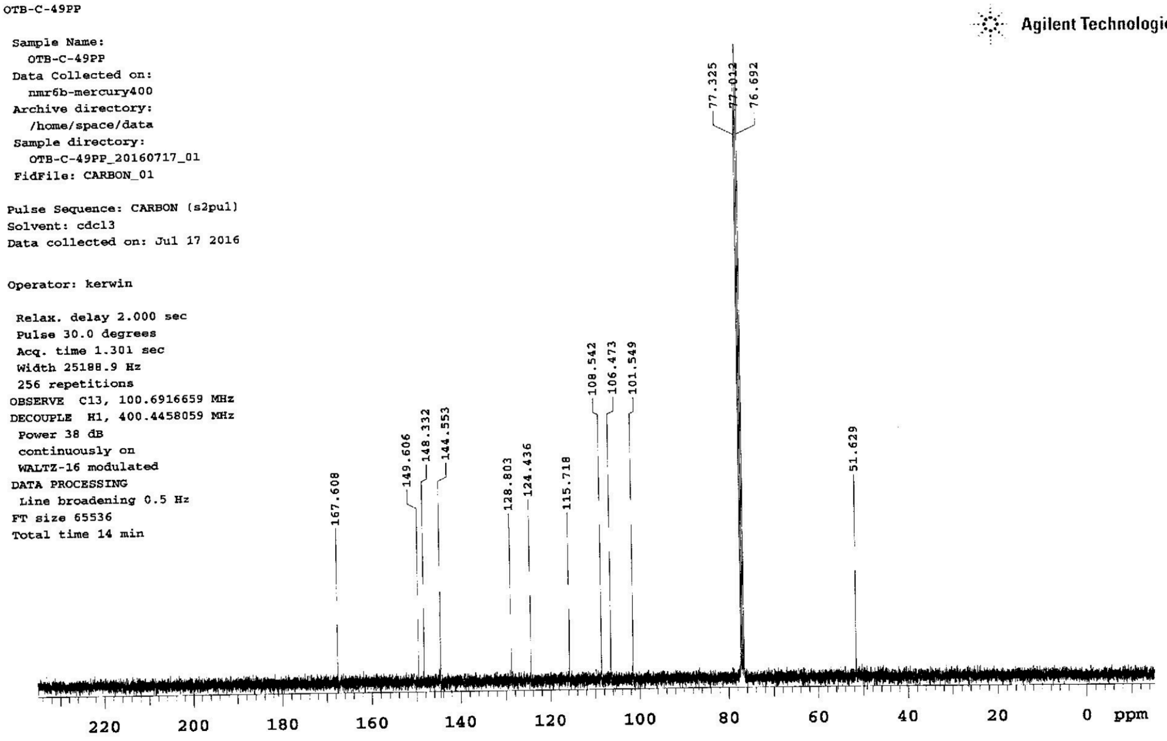
OTB-C-49PP
Sample Name:
OTB-C-49PP
Data Collected on:
nmr6b-mercury400
Archive directory:
/home/space/data
Sample directory:
OTB-C-49PP_20160717_01
FidFile: CARBON_01
Pulse Sequence: CARBON (s2pul)
Solvent: cdcl3
Data collected on: Jul 17 2016
Operator: kerwin
Relax. delay 2.000 sec
Pulse 30.0 degrees
Acq. time 1.301 sec
Width 25188.9 Hz
256 repetitions
OBSERVE C13, 100.6916659 MHz
DECOUPLE H1, 400.4458059 MHz
Power 38 dB
continuously on
WALTZ-16 modulated
DATA PROCESSING
Line broadening 0.5 Hz
FT size 65536
Total time 14 min
Agilent Technologies
77.325
77.012
76.692
167.608
149.606
148.332
144.553
128.803
124.436
115.718
108.542
106.473
101.549
51.629
220
200
180
160
140
120
100
80
60
40
20
0
ppm

Background RT 1.07 - 1.28 {36 scans}
SMK-4primeC5_ESIpos_inj1.datx 2021.12.09 14:10:11 ;
ESI +

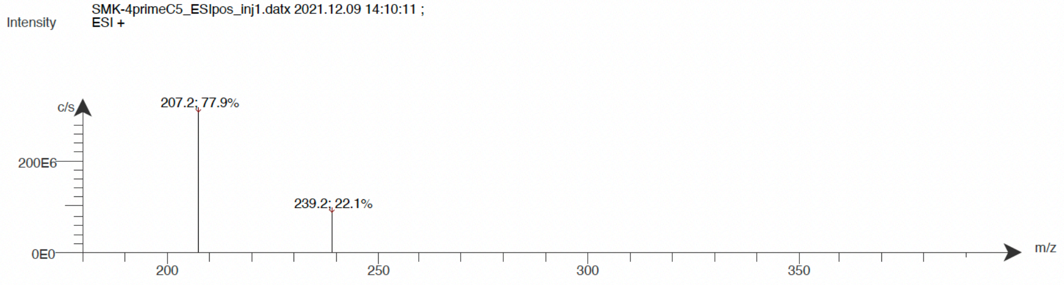

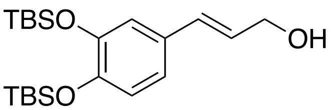

5A

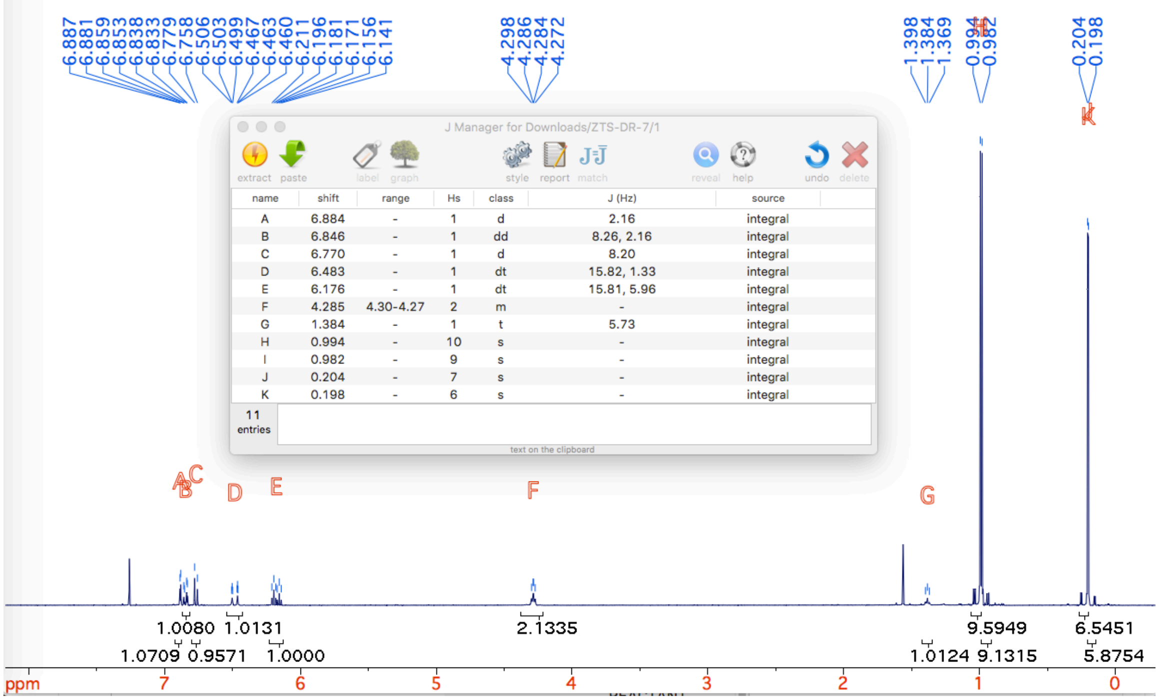

name	shift	range	Hs	class	J (Hz)	source
A	6.884	-	1	d	2.16	integral
B	6.846	-	1	dd	8.26, 2.16	integral
C	6.770	-	1	d	8.20	integral
D	6.483	-	1	dt	15.82, 1.33	integral
E	6.176	-	1	dt	15.81, 5.96	integral
F	4.285	4.30-4.27	2	m	-	integral
G	1.384	-	1	t	5.73	integral
H	0.994	-	10	s	-	integral
I	0.982	-	9	s	-	integral
J	0.204	-	7	s	-	integral
K	0.198	-	6	s	-	integral

11 entries

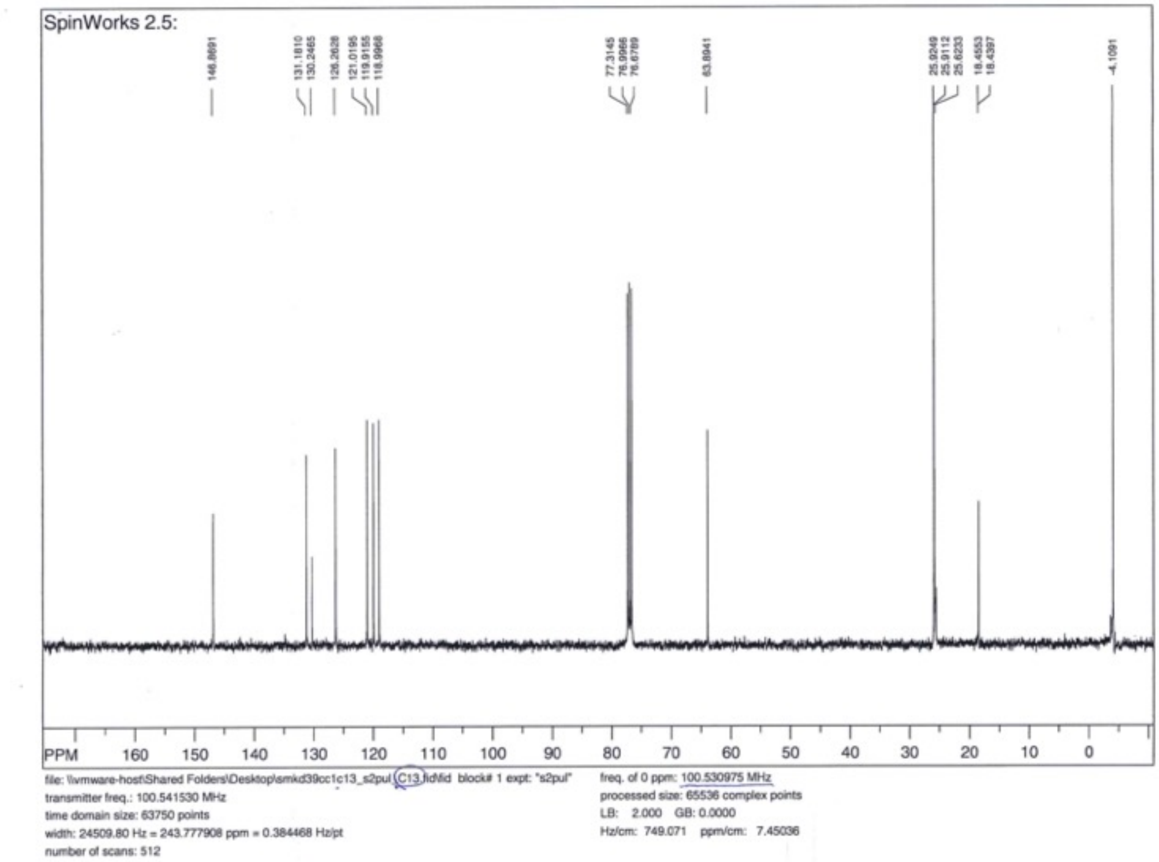

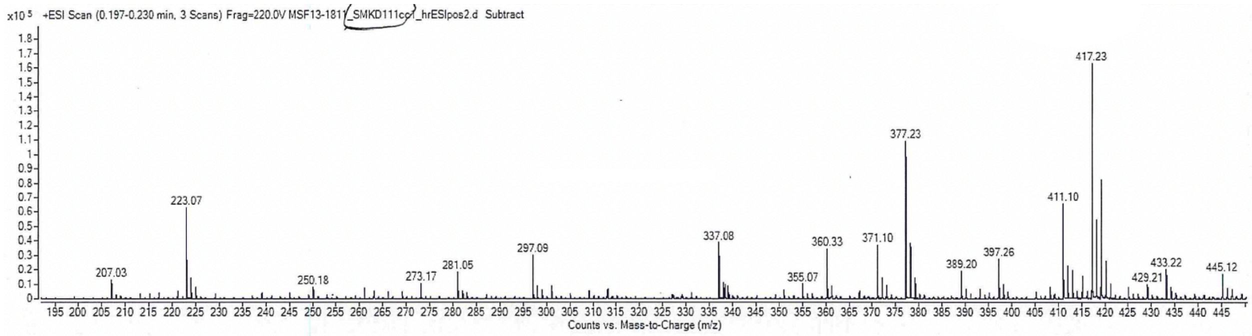

x10 5 +ESI Scan (0.197-0.230 min, 3 Scans) Frag=220.0V MSF13-181_SMKD111cc_hrESIpos2.d Subtract
417.23
377.23
411.10
223.07
337.08
360.33
371.10
297.09
397.26
389.20
433.22
445.12
429.21
281.05
207.03
273.17
355.07
250.18
Counts vs. Mass-to-Charge (m/z)

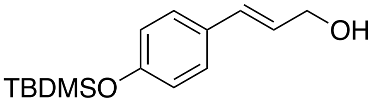

5B

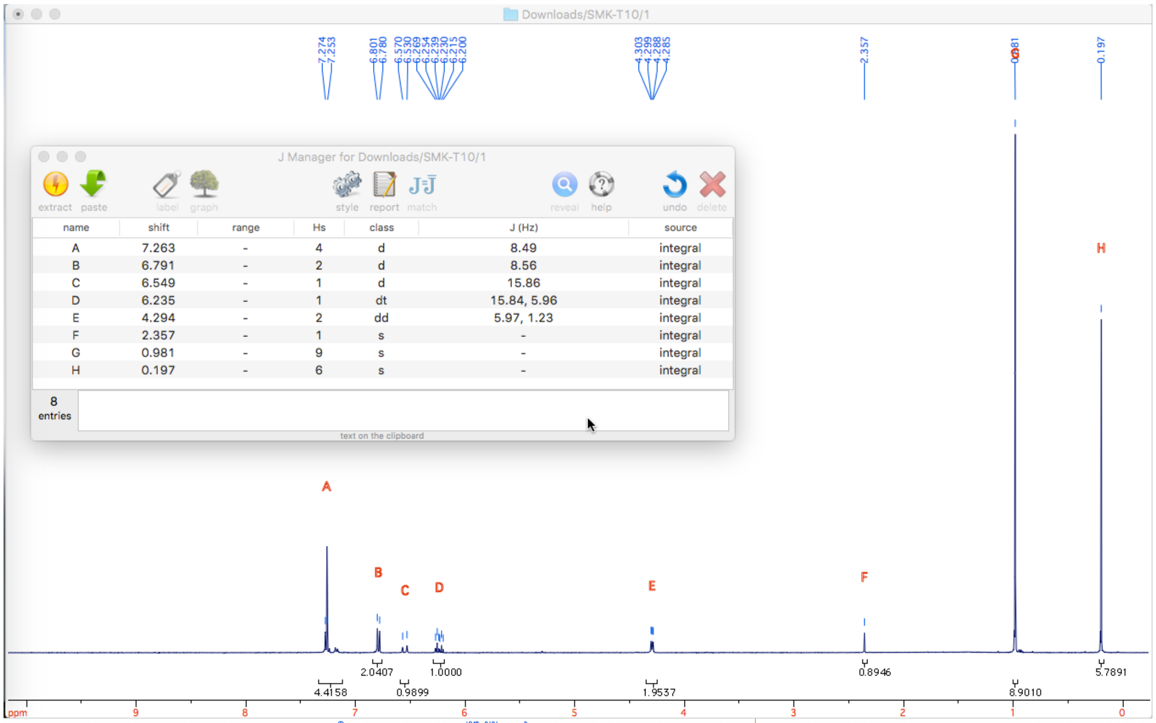

Downloads/SMK-T10/1
J Manager for Downloads/SMK-T10/1

name	shift	range	Hs	class	J (Hz)	source
A	7.263	-	4	d	8.49	integral
B	6.791	-	2	d	8.56	integral
C	6.549	-	1	d	15.86	integral
D	6.235	-	1	dt	15.84, 5.96	integral
E	4.294	-	2	dd	5.97, 1.23	integral
F	2.357	-	1	s	-	integral
G	0.981	-	9	s	-	integral
H	0.197	-	6	s	-	integral

8 entries

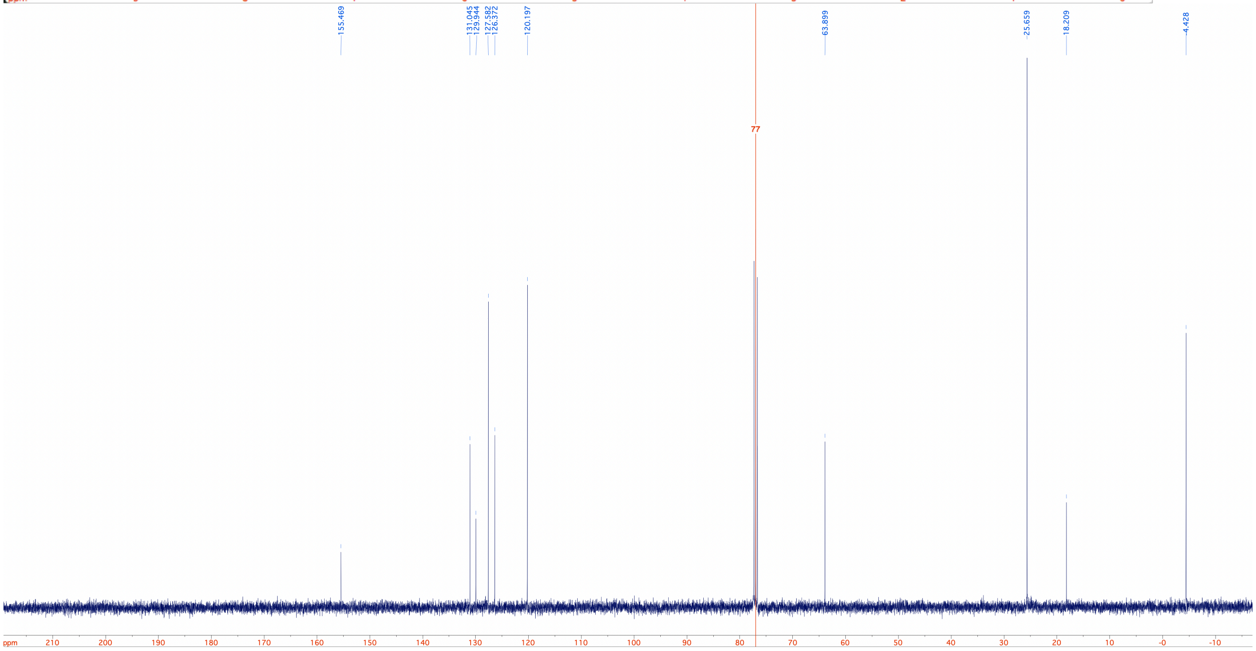

155.469
131.045
129.944
127.592
126.592
120.197
77
63.899
25.659
18.209
-4.428

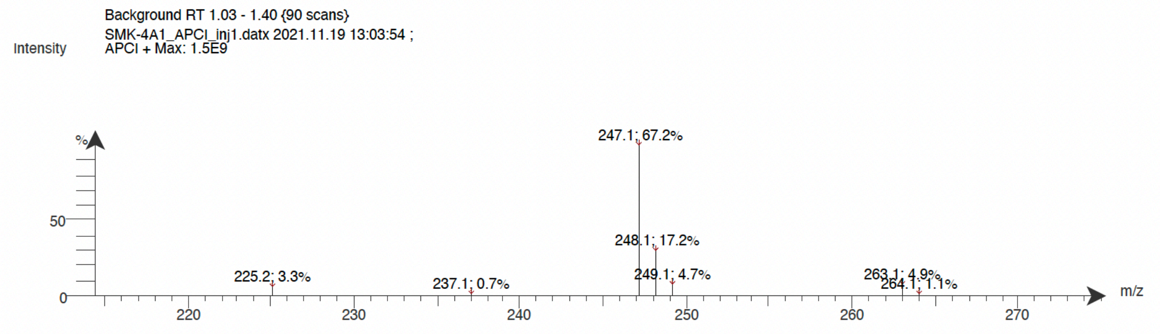

Background RT 1.03 - 1.40 {90 scans}
SMK-4A1_APCI_inj1.datx 2021.11.19 13:03:54 ;
Intensity
APCI + Max: 1.5E9
%
50
0
247.1; 67.2%
248.1; 17.2%
249.1; 4.7%
225.2; 3.3%
237.1; 0.7%
263.1; 4.9%
264.1; 1.1%
220
230
240
250
260
270
m/z

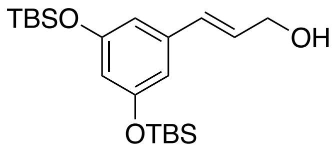

5C

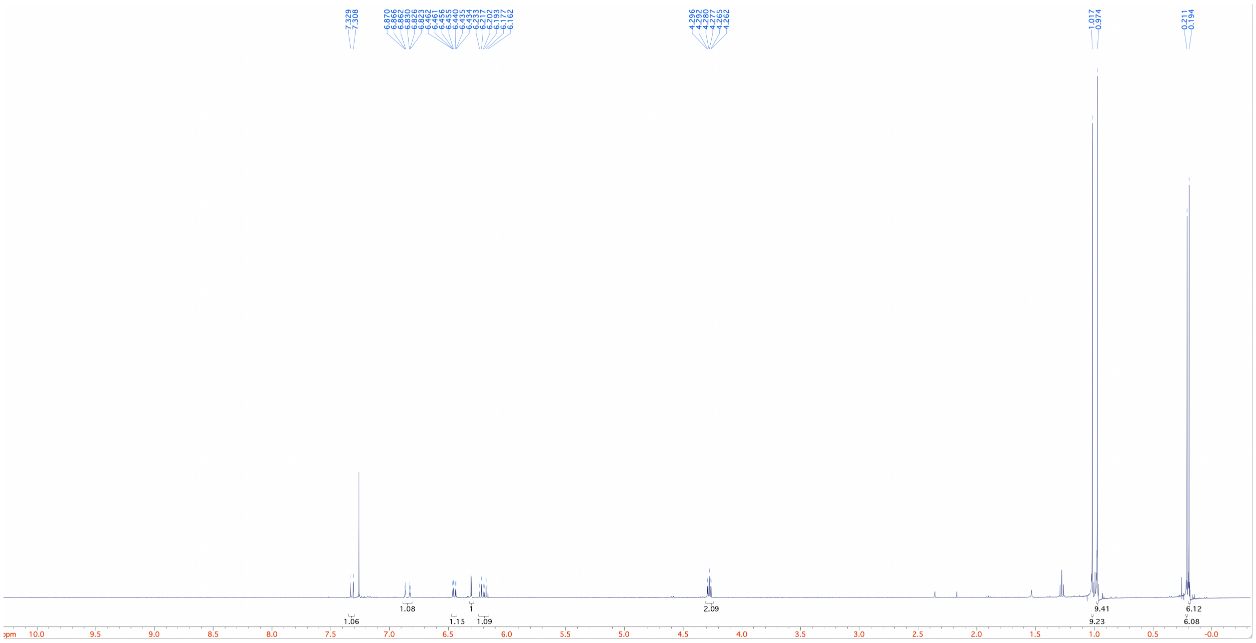

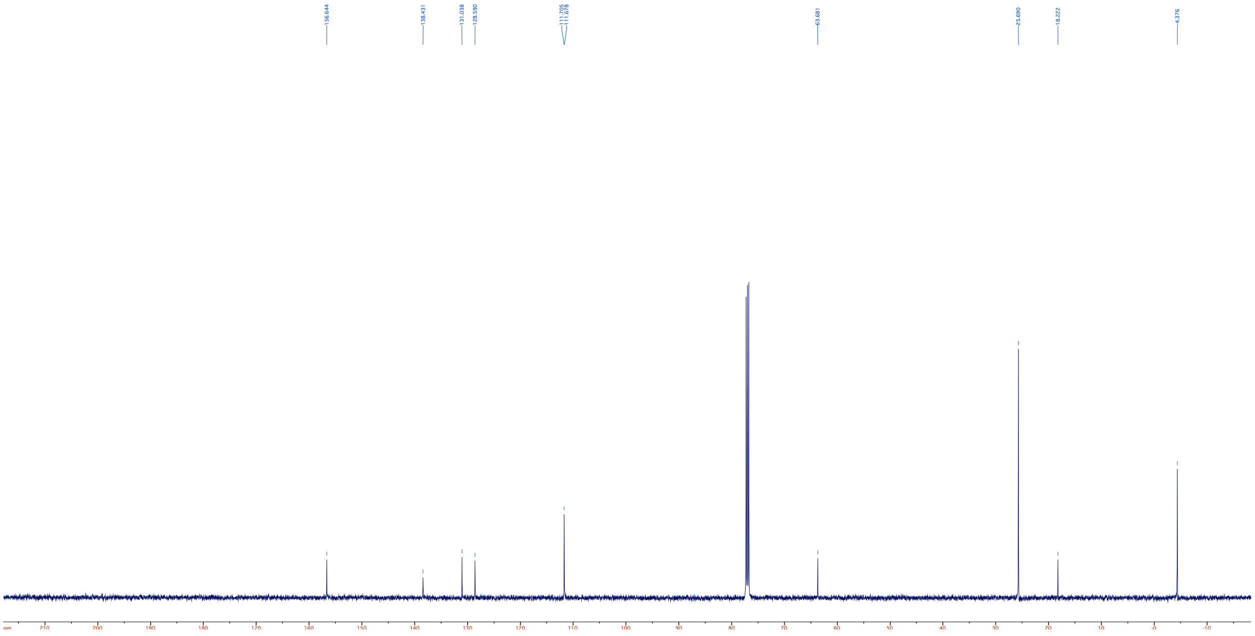

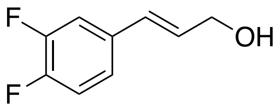

5D

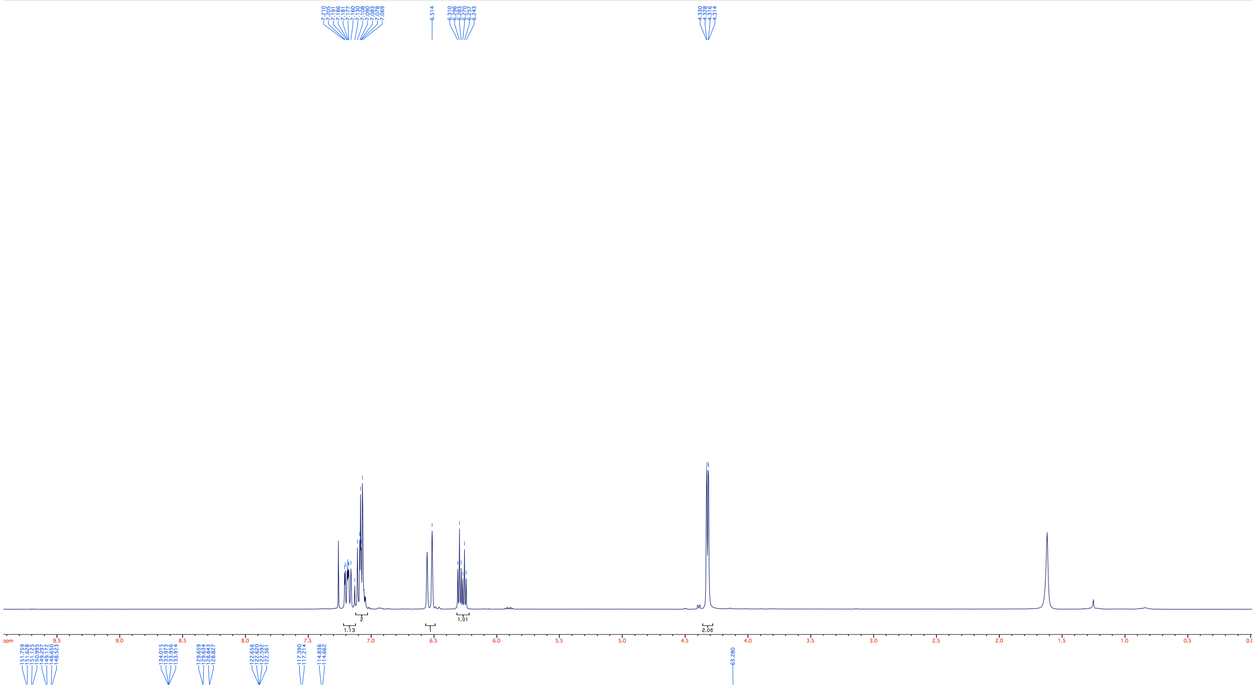

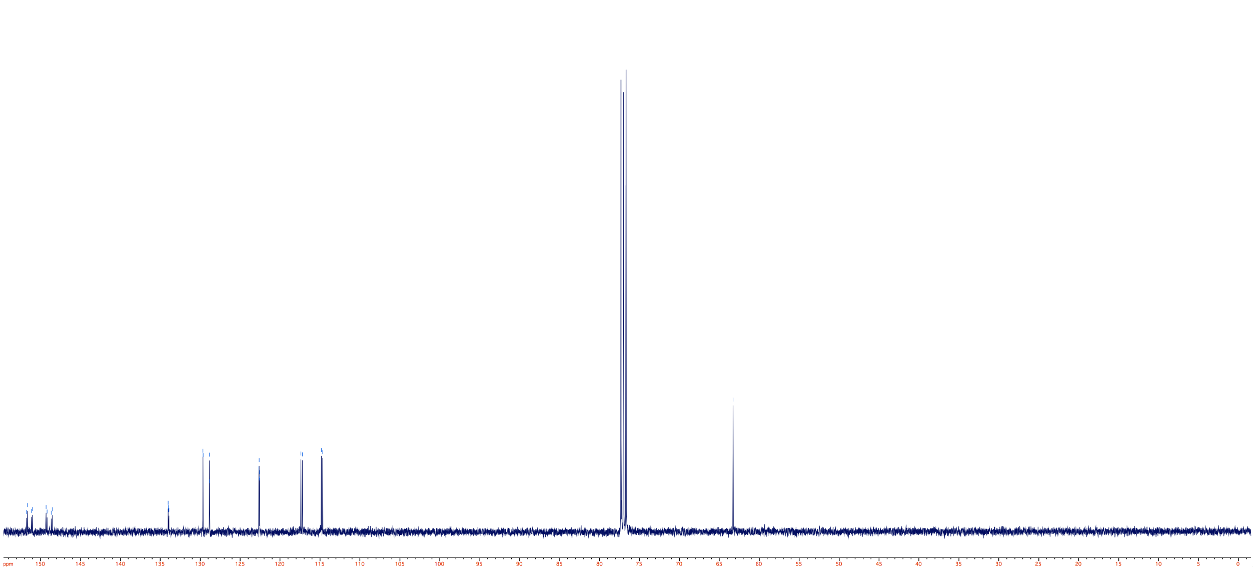

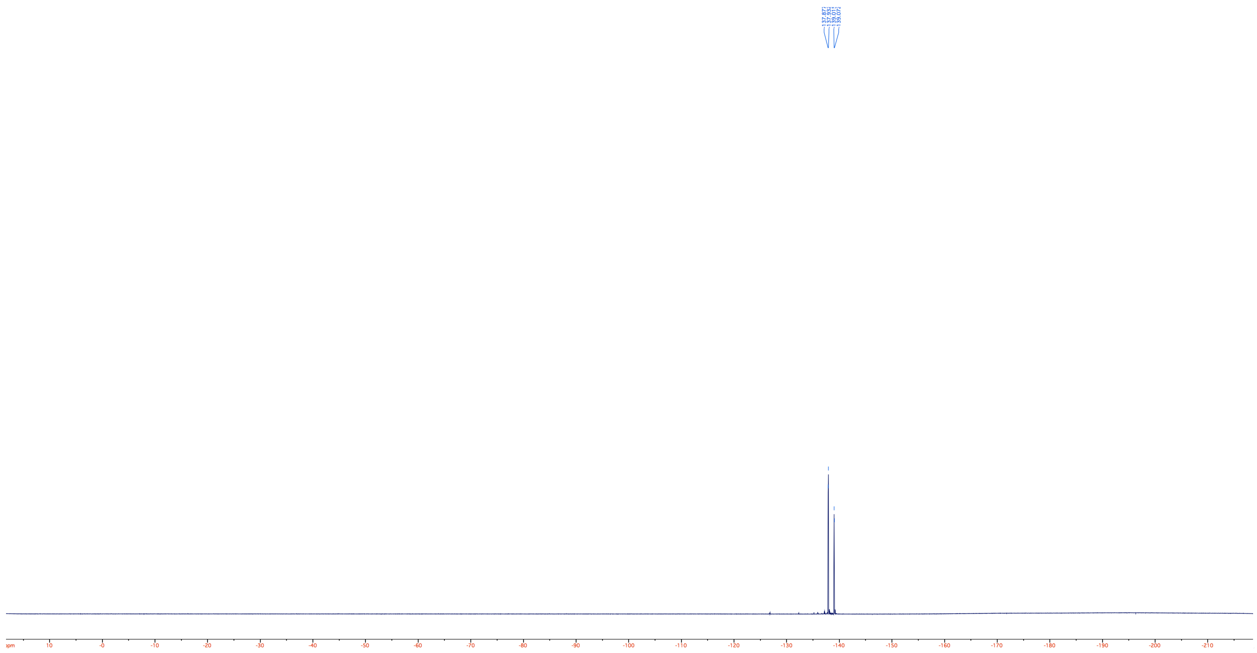

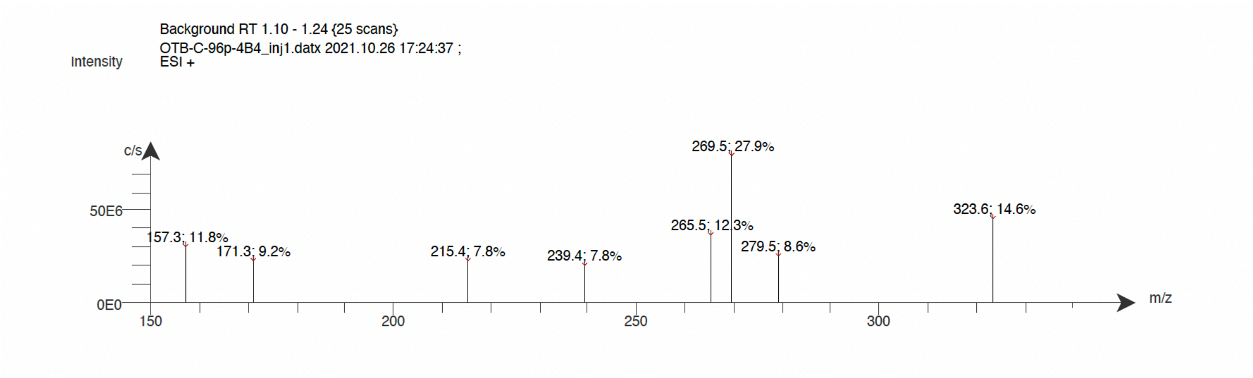

Background RT 1.10 - 1.24 {25 scans}
OTB-C-96p-4B4_inj1.datx 2021.10.26 17:24:37 ;
ESI +
Intensity
c/s
50E6
0E0
157.3; 11.8%
171.3; 9.2%
215.4; 7.8%
239.4; 7.8%
265.5; 12.3%
269.5; 27.9%
279.5; 8.6%
323.6; 14.6%
150
200
250
300
m/z

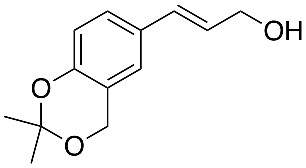

5F

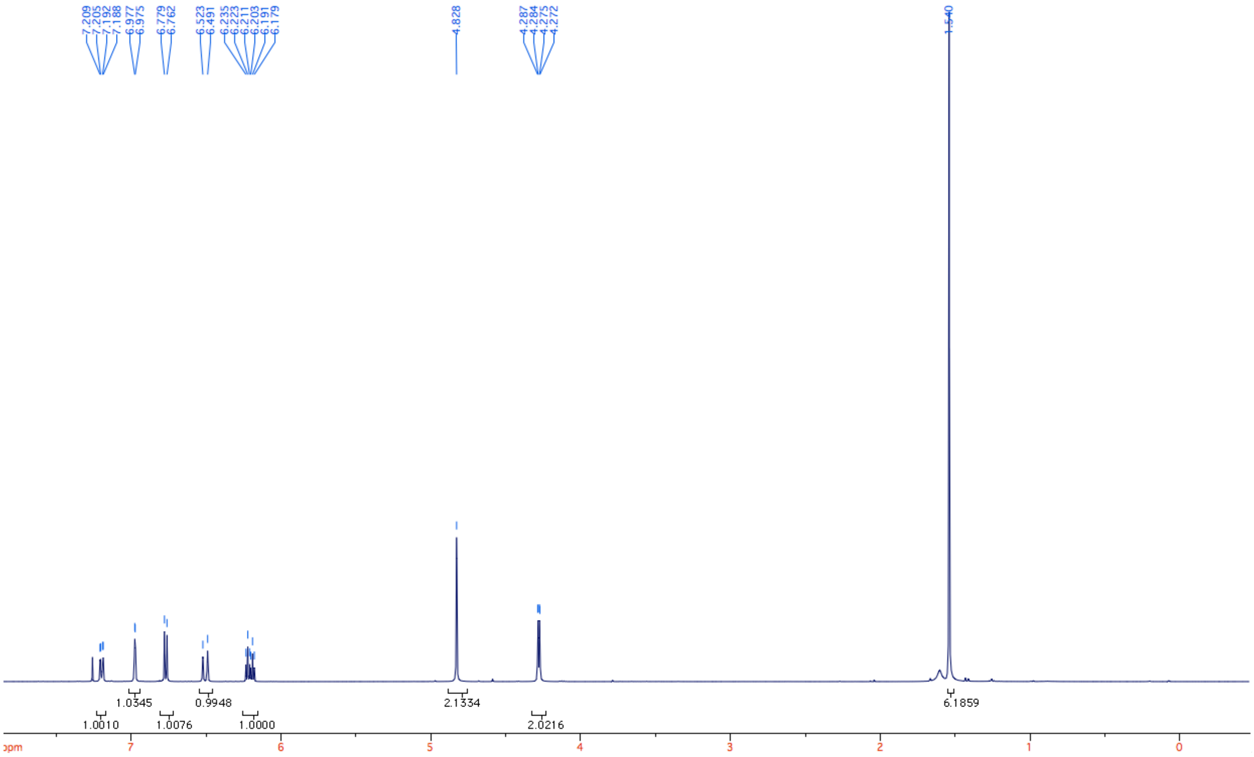
7.209
7.205
7.192
7.188
6.977
6.975
6.779
6.762
6.523
6.491
6.235
6.223
6.211
6.203
6.191
6.179
4.828
4.287
4.284
4.275
4.272
1.540
1.0010
1.0345
1.0076
0.9948
1.0000
2.1334
2.0216
6.1859
ppm
7
6
5
4
3
2
1
0

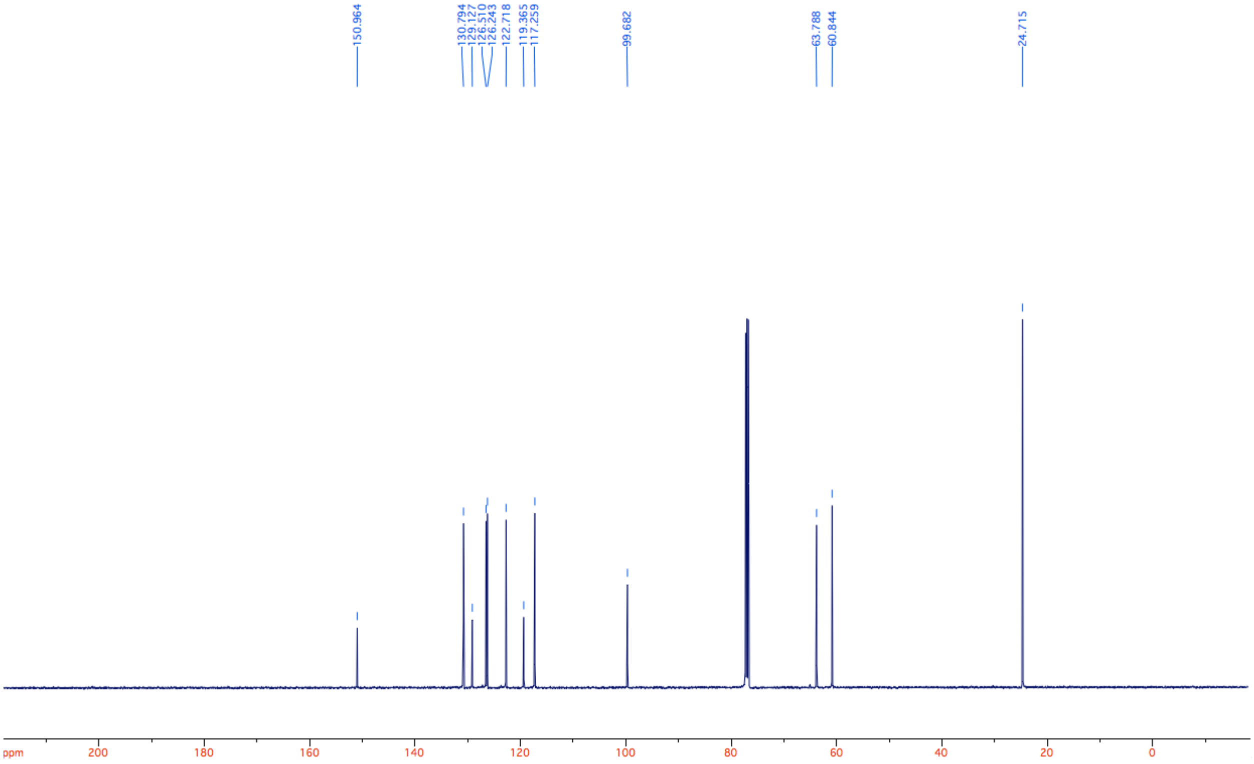
150.964
130.794
129.127
126.510
126.243
122.718
119.365
117.259
99.682
63.788
60.844
24.715
ppm
200
180
160
140
120
100
80
60
40
20
0

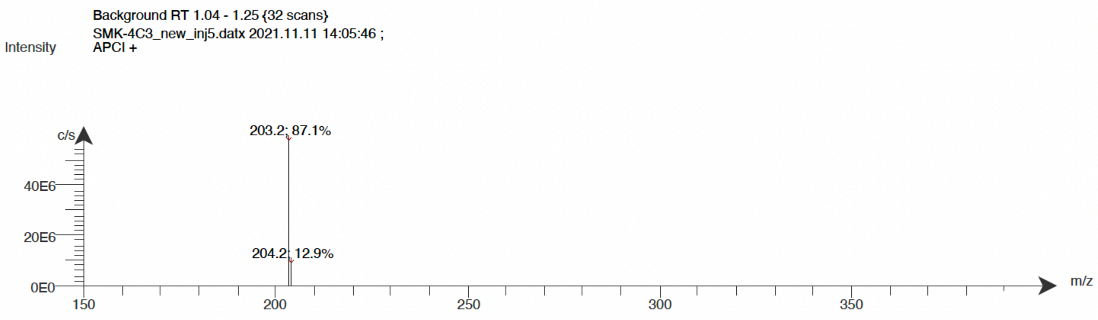
Background RT 1.04 - 1.25 {32 scans}
SMK-4C3_new_inj5.datx 2021.11.11 14:05:46 ;
Intensity
APCI +
c/s
40E6
20E6
0E0
203.2; 87.1%
204.2; 12.9%
150
200
250
300
350
m/z

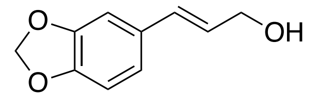
O
O
OH

5E

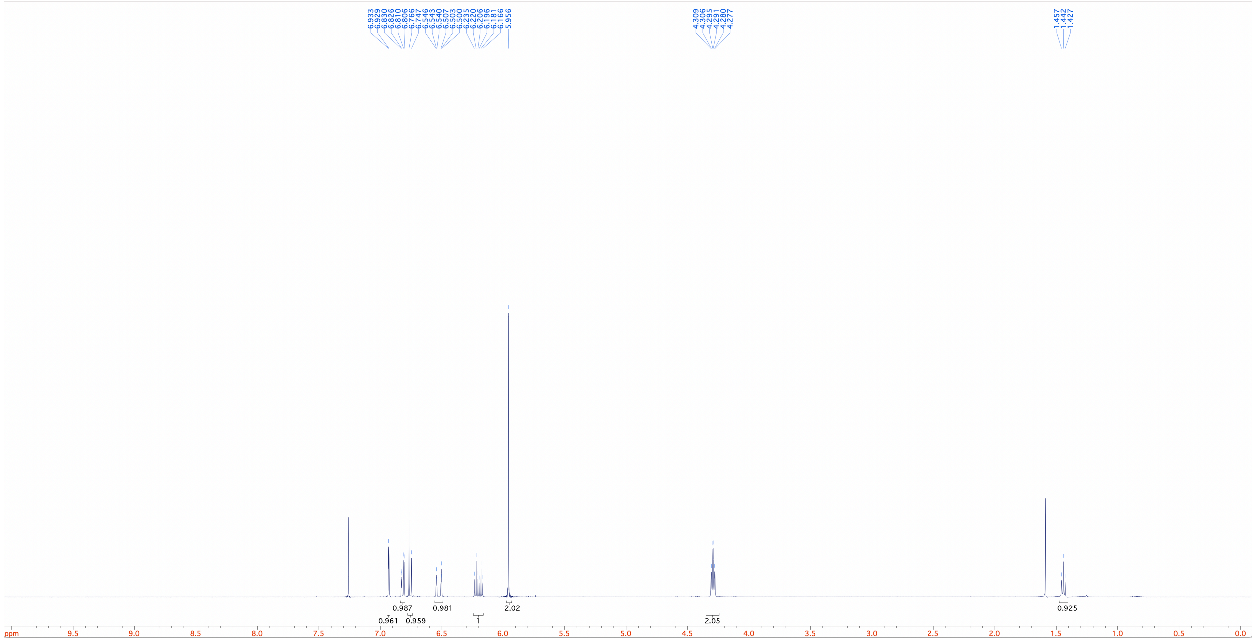
6.933
6.929
6.830
6.826
6.810
6.806
6.766
6.747
6.546
6.543
6.540
6.507
6.503
6.500
6.235
6.220
6.206
6.196
6.181
6.166
5.956
4.309
4.306
4.295
4.291
4.280
4.277
1.457
1.442
1.427
0.961
0.987
0.959
0.981
1
2.02
2.05
0.925
ppm
9.5
9.0
8.5
8.0
7.5
7.0
6.5
6.0
5.5
5.0
4.5
4.0
3.5
3.0
2.5
2.0
1.5
1.0
0.5
0.0

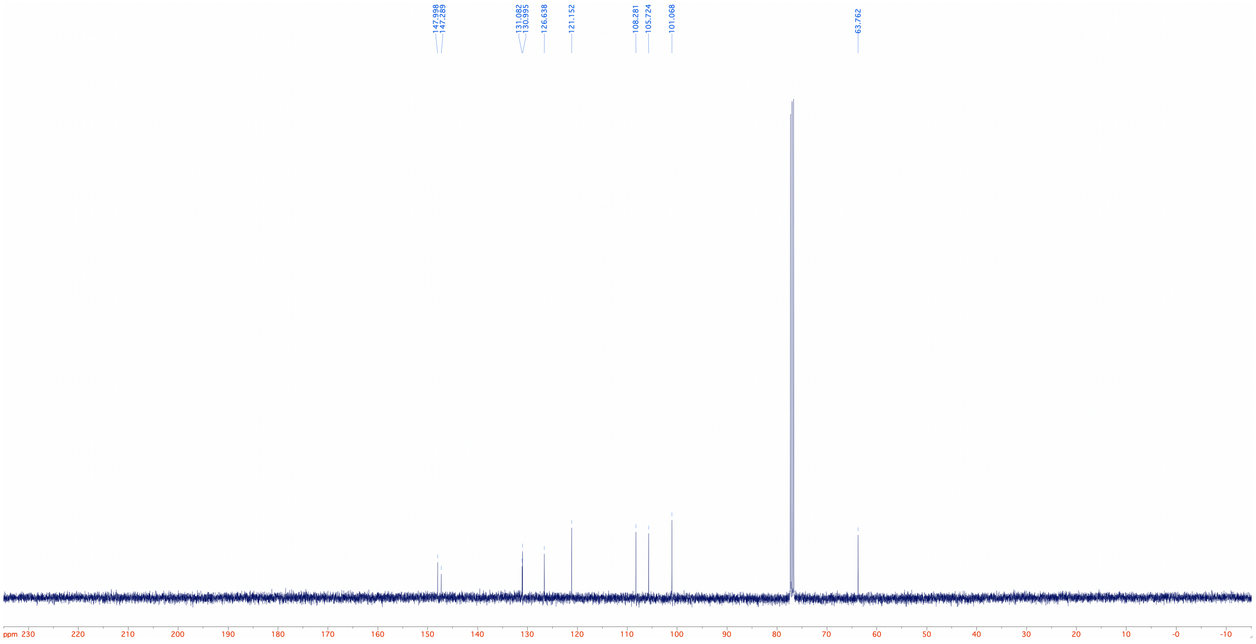
147.998
147.289
131.082
130.995
126.638
121.152
108.281
105.724
101.068
63.762
ppm 230
220
210
200
190
180
170
160
150
140
130
120
110
100
90
80
70
60
50
40
30
20
10
-0
-10

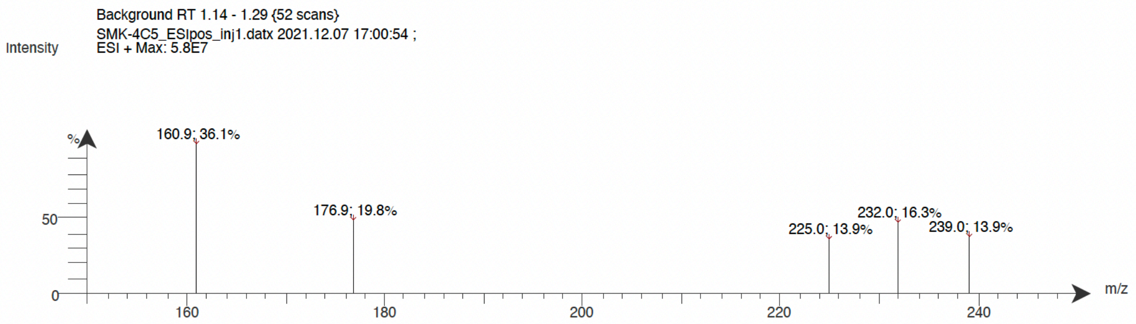

Background RT 1.14 - 1.29 {52 scans}
SMK-4C5_ESIpos_inj1.datx 2021.12.07 17:00:54 ;
Intensity
ESI + Max: 5.8E7
%
50
0
160.9; 36.1%
176.9; 19.8%
225.0; 13.9%
232.0; 16.3%
239.0; 13.9%
160
180
200
220
240
m/z

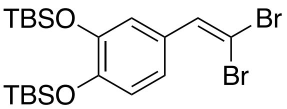

6A

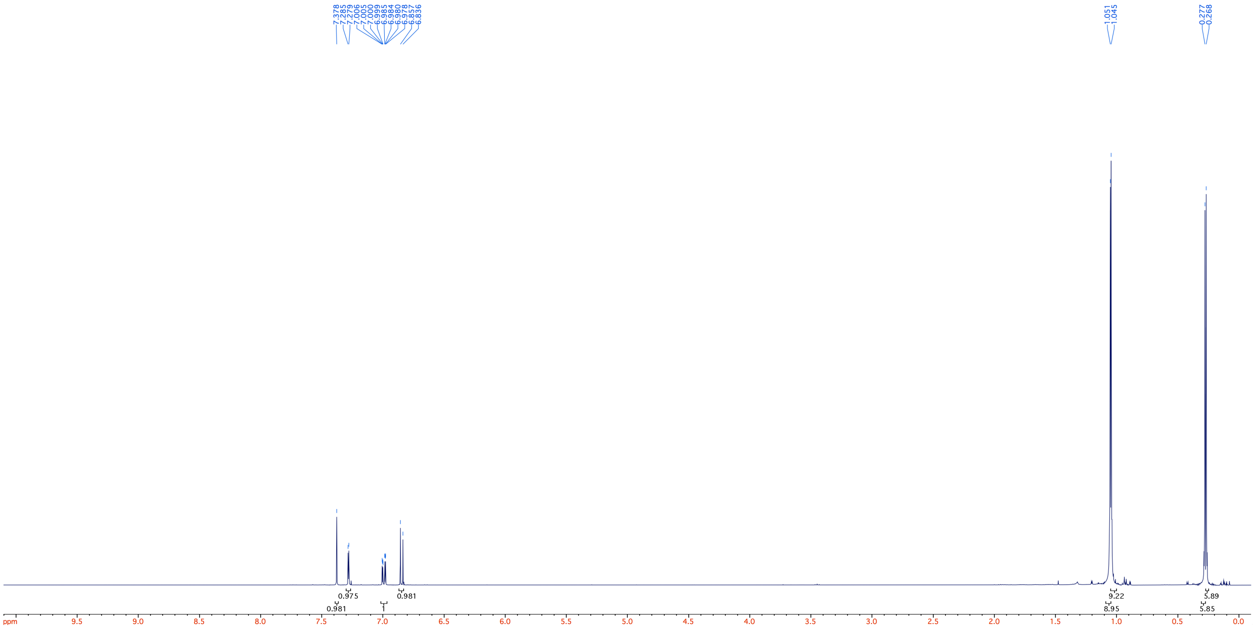

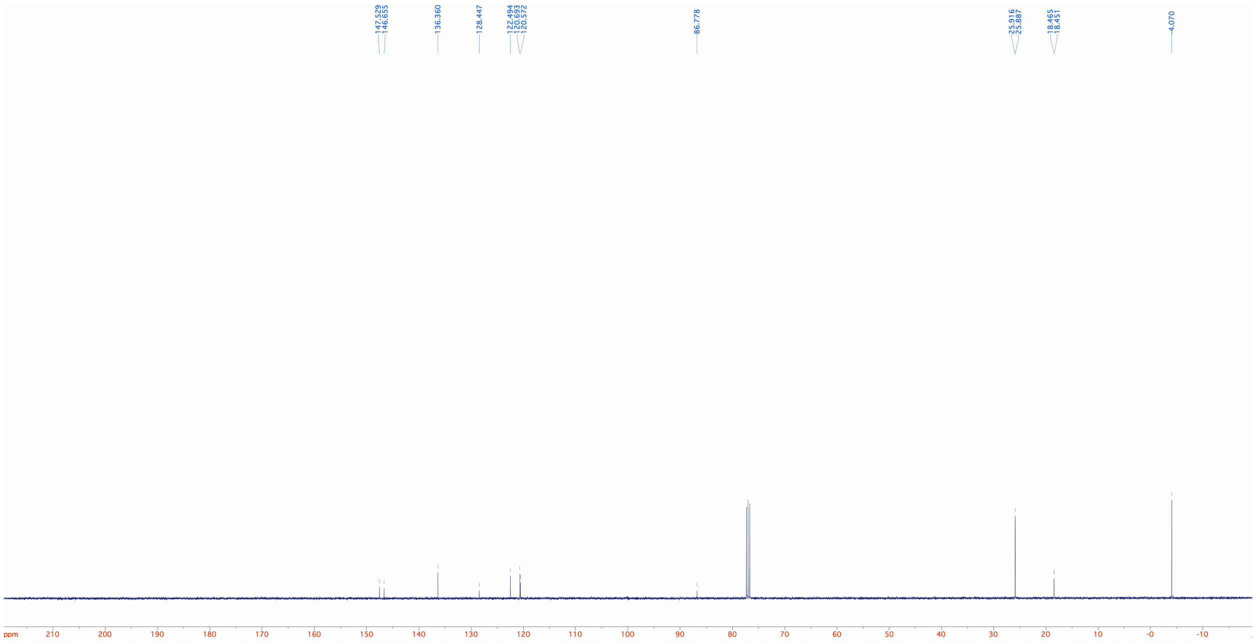

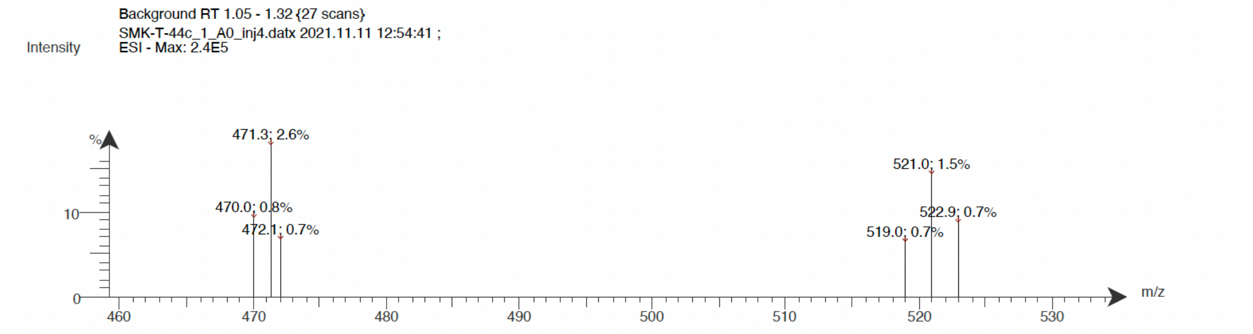
Background RT 1.05 - 1.32 {27 scans}
SMK-T-44c_1_A0_inj4.datx 2021.11.11 12:54:41 ;
ESI - Max: 2.4E5
Intensity
471.3; 2.6%
470.0; 0.8%
472.1; 0.7%
521.0; 1.5%
522.9; 0.7%
519.0; 0.7%
m/z

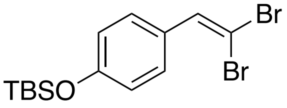
Br
Br
TBSO

6B

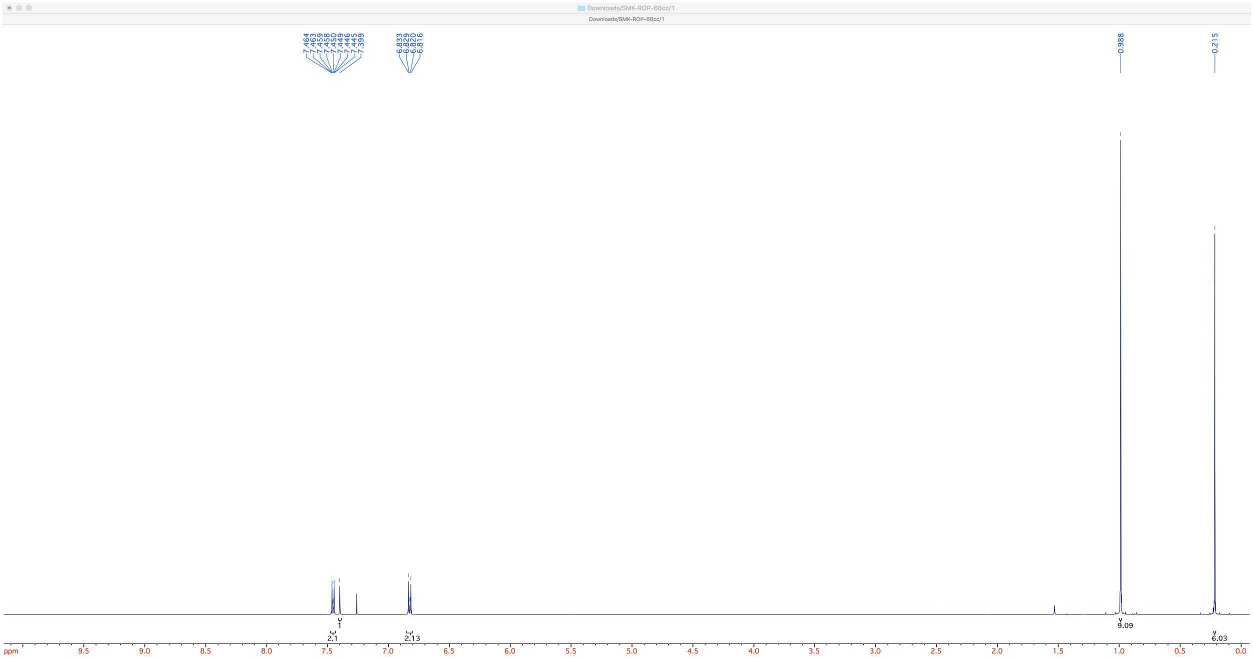
Downloads/SMK-ROP-66cc/1
7.464
7.463
7.459
7.458
7.450
7.449
7.446
7.445
7.399
6.833
6.829
6.820
6.816
0.988
0.215
2.1
1
2.13
9.09
6.03
ppm

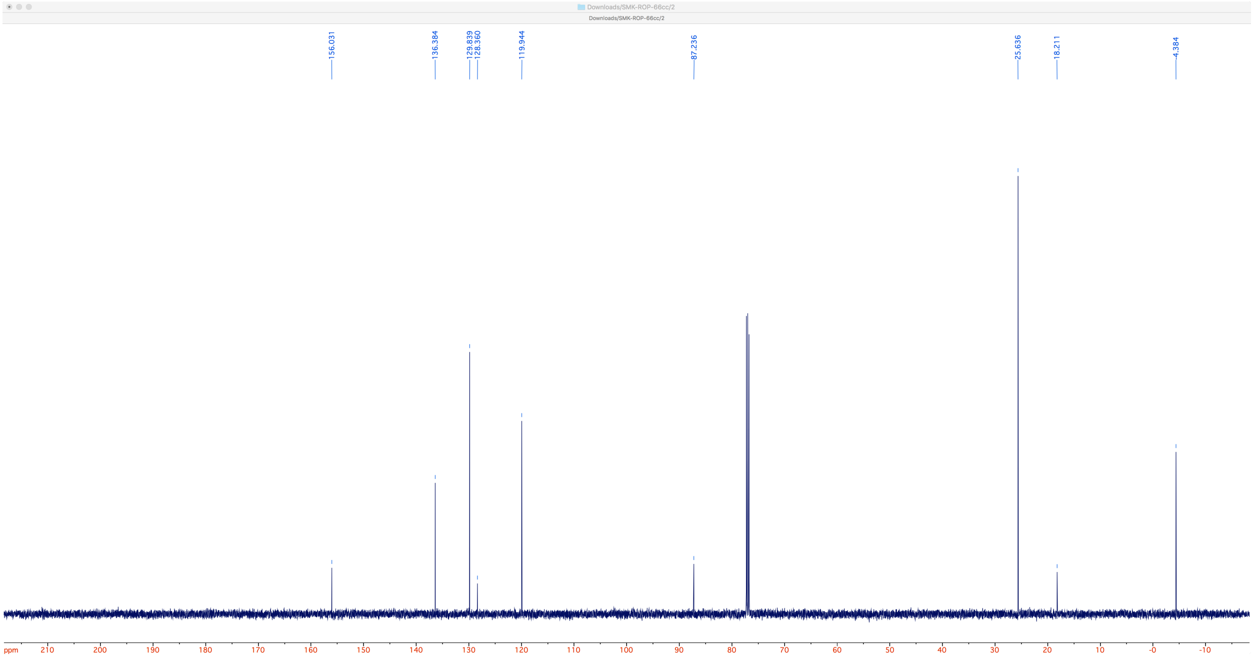
Downloads/SMK-ROP-66cc/2
156.031
136.384
129.839
128.360
119.944
87.236
25.636
18.211
-4.384
ppm

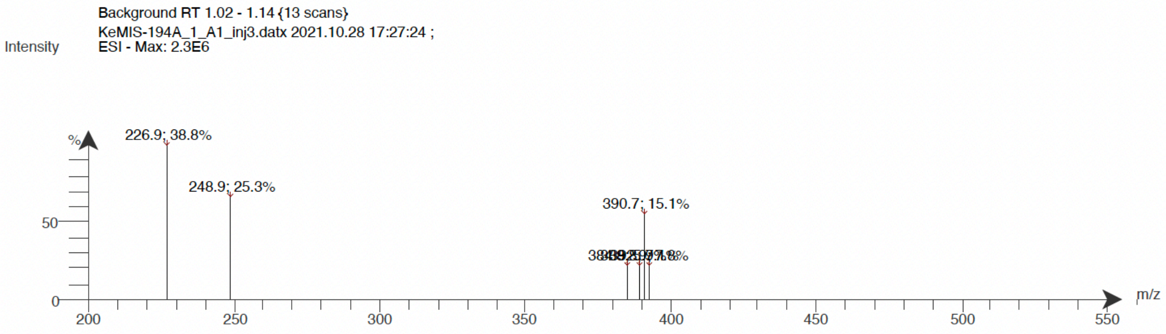
Background RT 1.02 - 1.14 {13 scans}
KeMIS-194A_1_A1_inj3.datx 2021.10.28 17:27:24 ;
Intensity
ESI - Max: 2.3E6
%
226.9; 38.8%
248.9; 25.3%
390.7; 15.1%
50
0
200
250
300
350
400
450
500
550
m/z

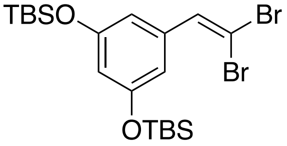
TBSO
Br
Br
OTBS

6C

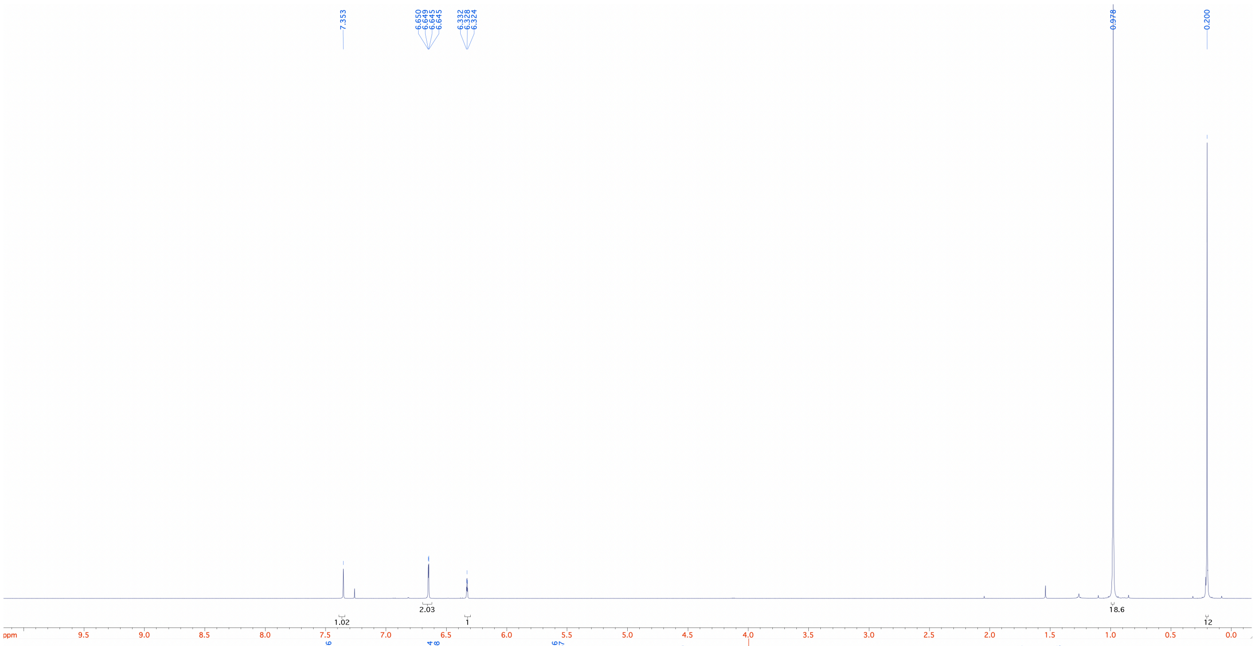
7.353
6.650
6.649
6.645
6.645
6.332
6.328
6.324
0.978
0.200
1.02
2.03
1
18.6
12
ppm

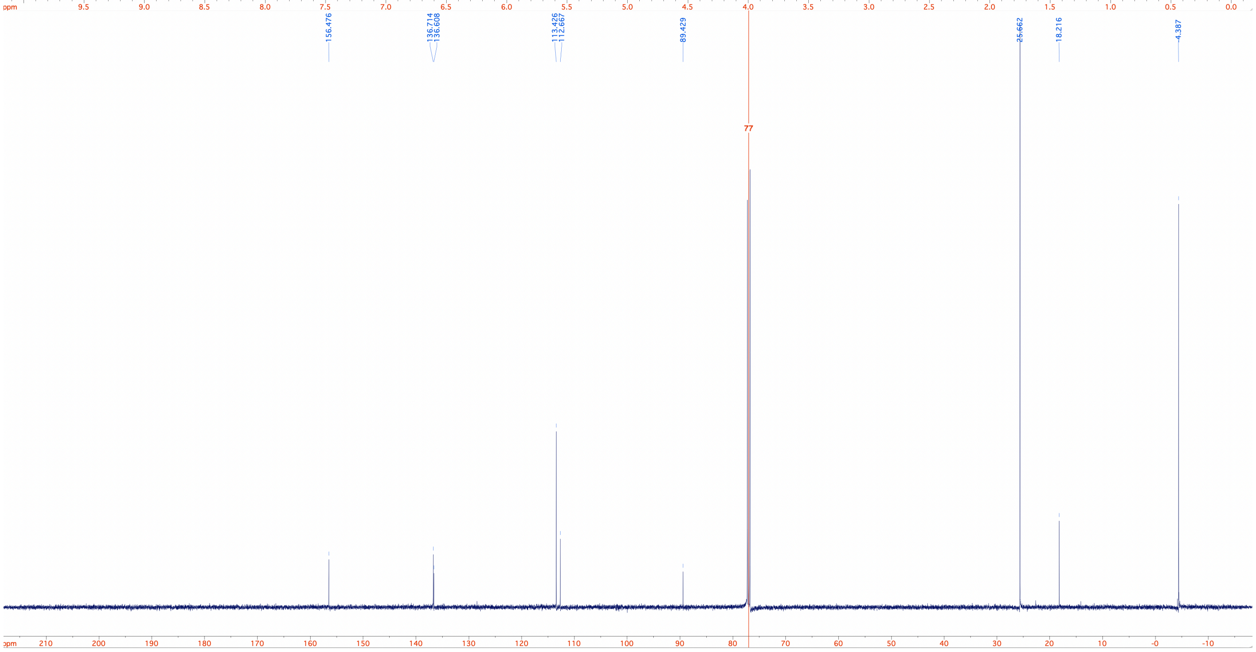
156.476
136.714
136.608
113.426
112.667
89.429
77
25.662
18.216
-4.387
ppm

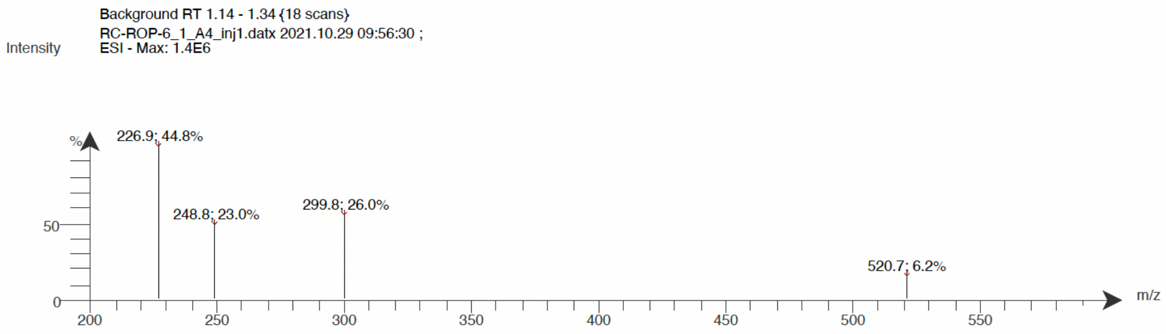
Background RT 1.14 - 1.34 {18 scans}
RC-ROP-6_1_A4_inj1.datx 2021.10.29 09:56:30 ;
Intensity
ESI - Max: 1.4E6
%
226.9; 44.8%
248.8; 23.0%
299.8; 26.0%
520.7; 6.2%
50
0
200
250
300
350
400
450
500
550
m/z

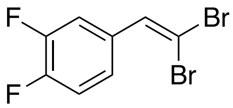

6D

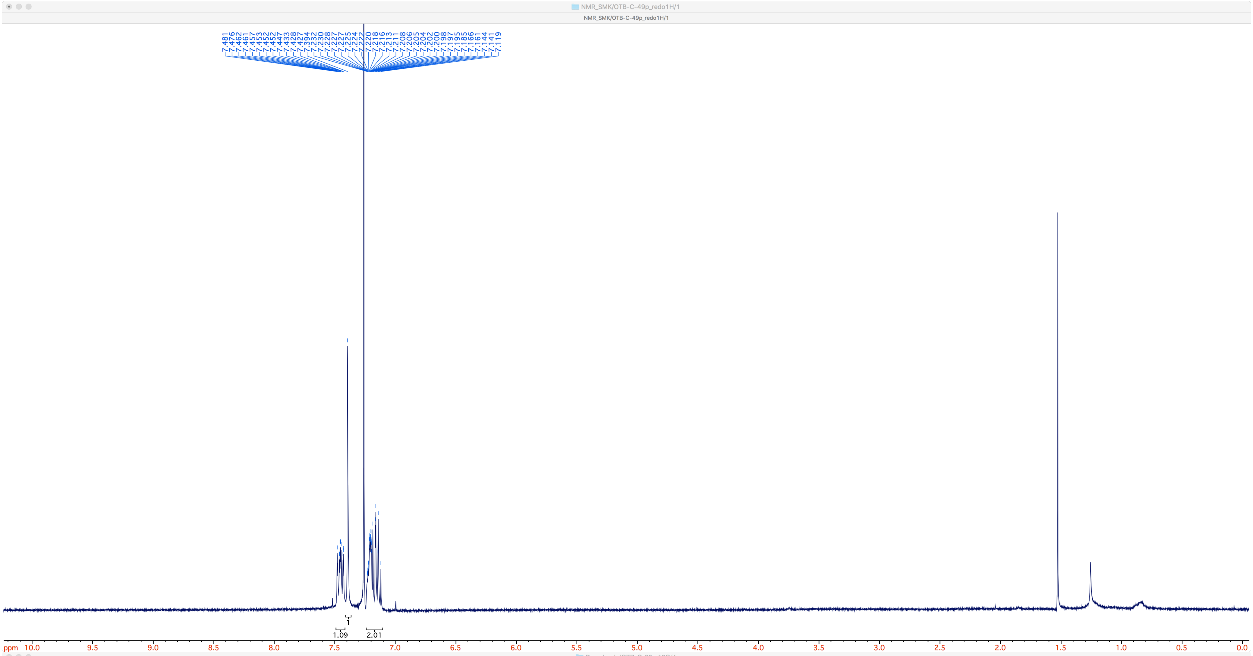

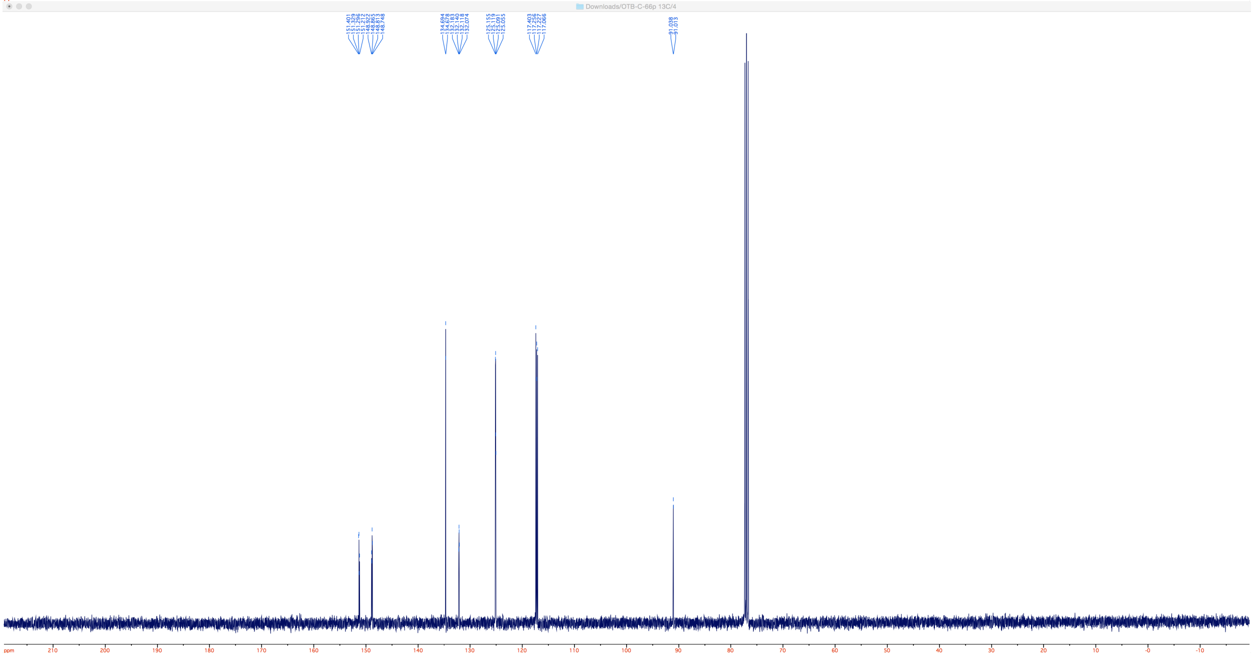

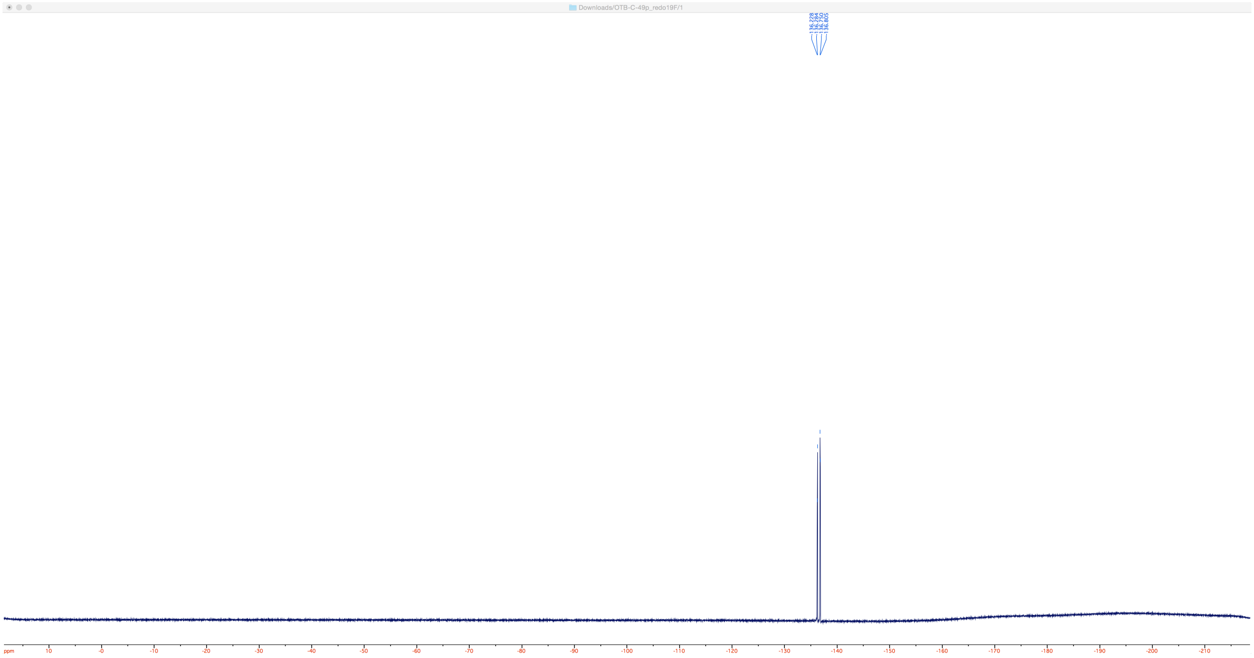
Downloads/OTB-C-49p_redo19F/1
136.228
136.284
136.750
136.805
ppm
10
0
-10
-20
-30
-40
-50
-60
-70
-80
-90
-100
-110
-120
-130
-140
-150
-160
-170
-180
-190
-200
-210

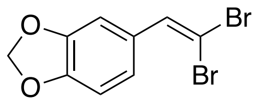

6E

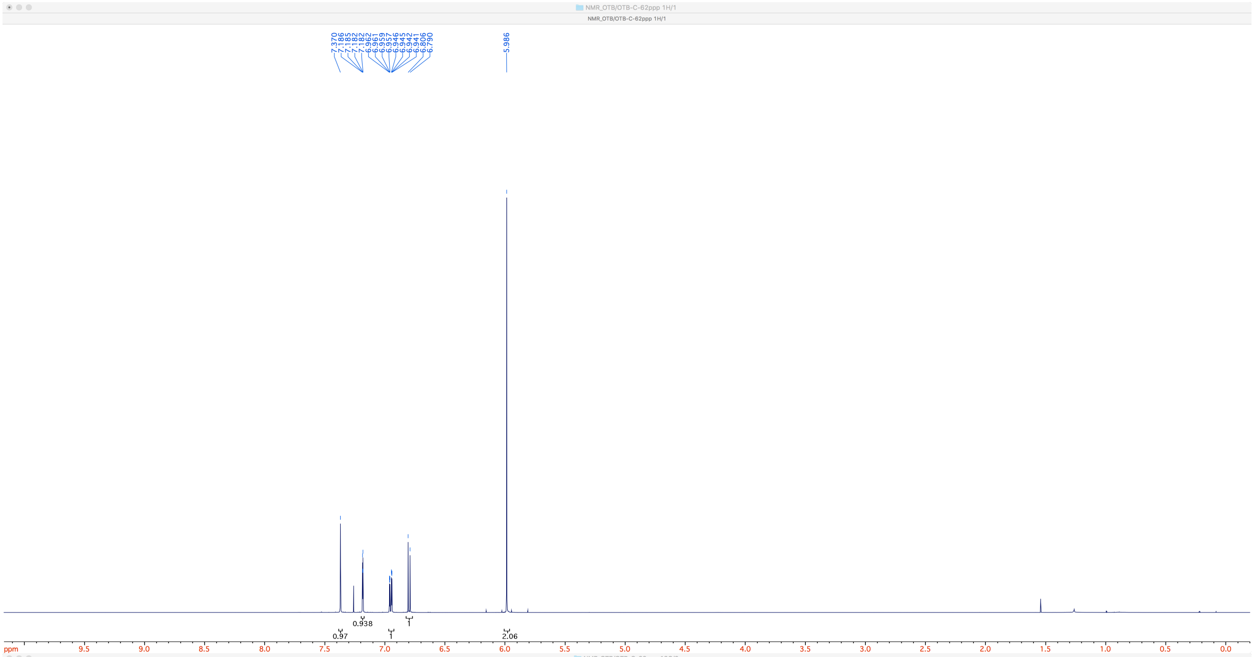

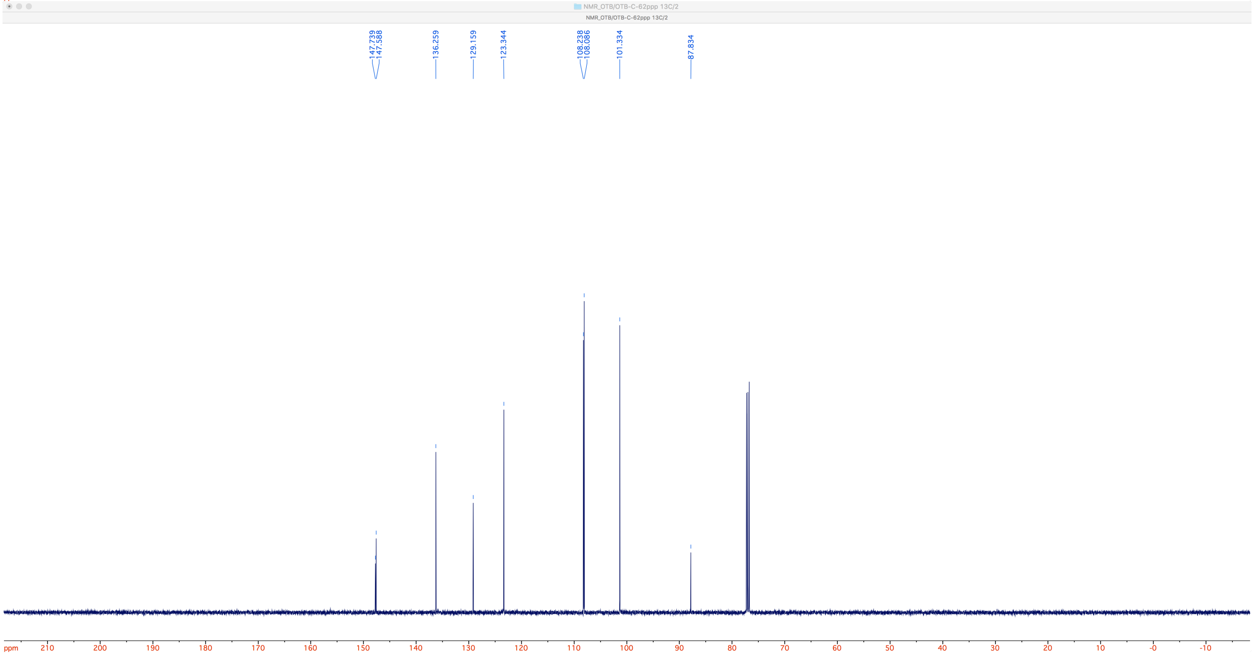

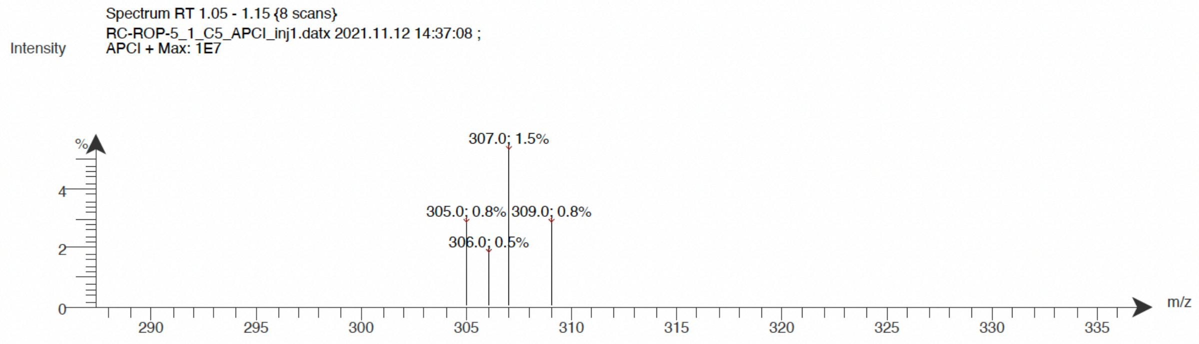
Spectrum RT 1.05 - 1.15 {8 scans}
RC-ROP-5_1_C5_APCI_inj1.datx 2021.11.12 14:37:08 ;
Intensity
APCI + Max: 1E7
%
307.0; 1.5%
305.0; 0.8%
309.0; 0.8%
306.0; 0.5%
m/z
290
295
300
305
310
315
320
325
330
335

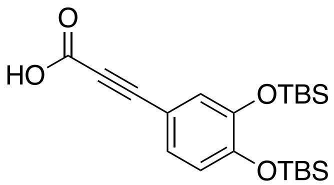

7A

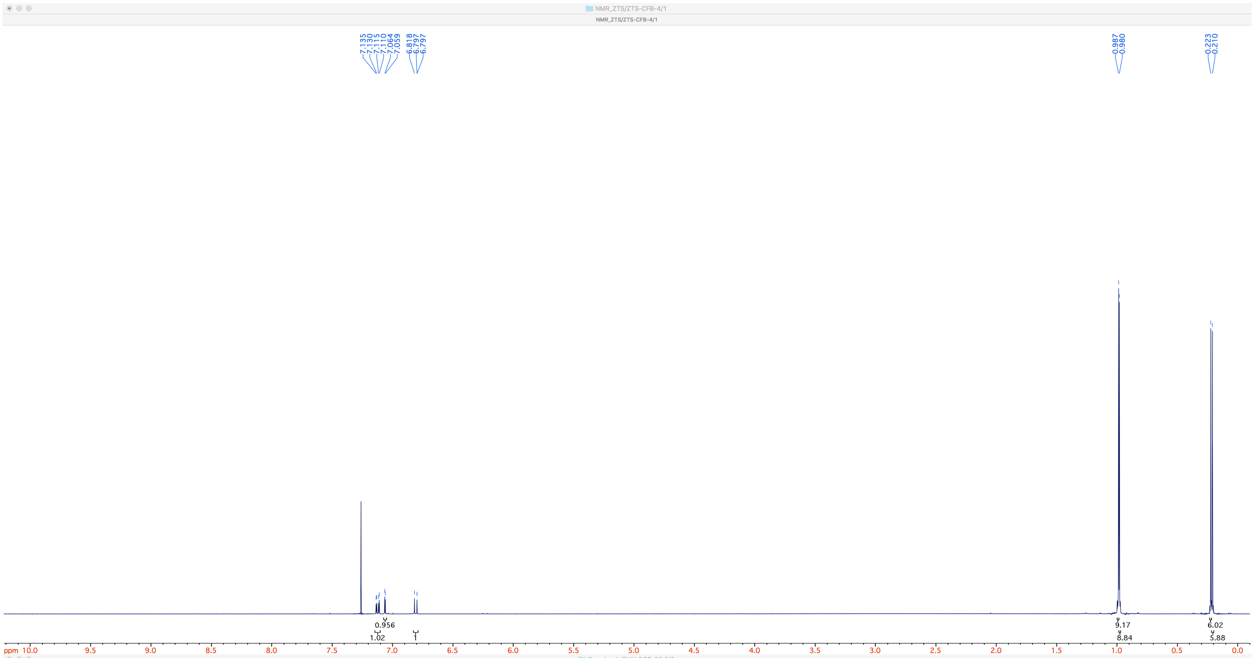

NMR_ZTS/ZTS-CF8-4/1
7.135
7.130
7.115
7.110
7.064
7.059
6.818
6.797
6.797
0.987
0.980
0.223
0.210
0.956
1.02
1
9.17
8.84
6.02
5.88
ppm

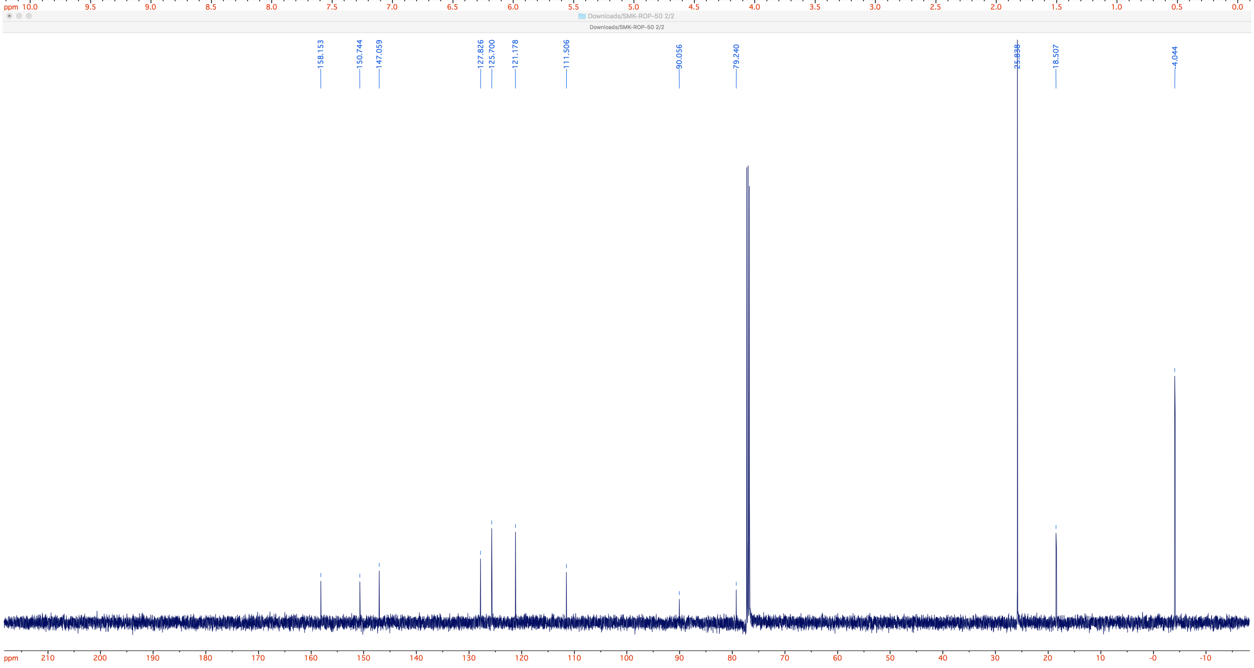

Downloads/SMK-ROP-50 2/2
158.153
150.744
147.059
127.826
125.700
121.178
111.506
90.056
79.240
25.838
18.507
-4.044
ppm

User Spectra

Fragmentor Voltage	Collision Energy	Ionization Mode
150	0	ESI

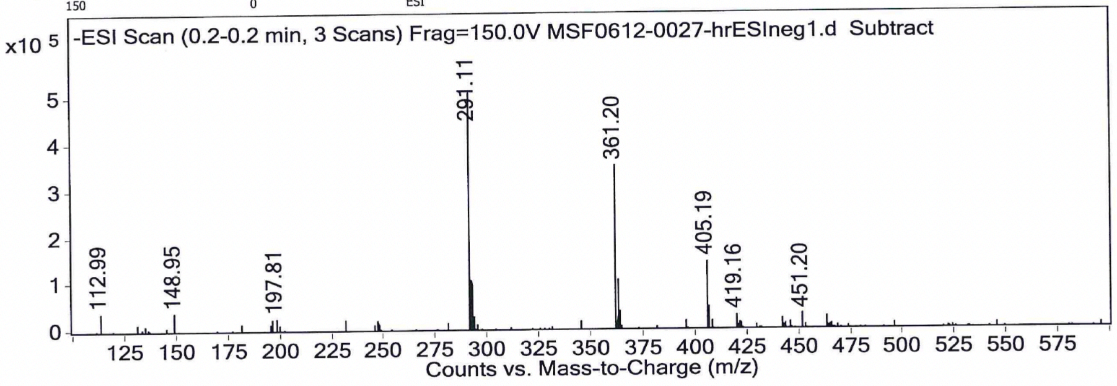

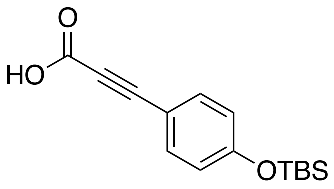
O
HO
OTBS

7B

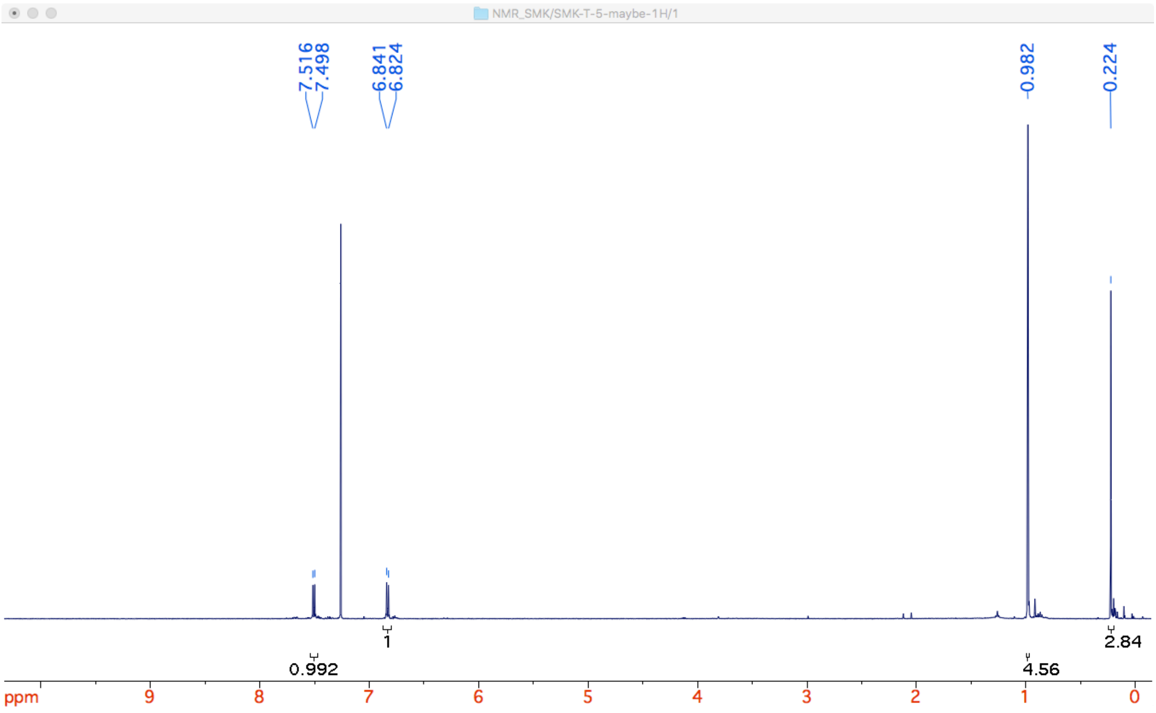
NMR_SMK/SMK-T-5-maybe-1H/1
7.516
7.498
6.841
6.824
0.982
0.224
0.992
1
4.56
2.84
ppm
9
8
7
6
5
4
3
2
1
0

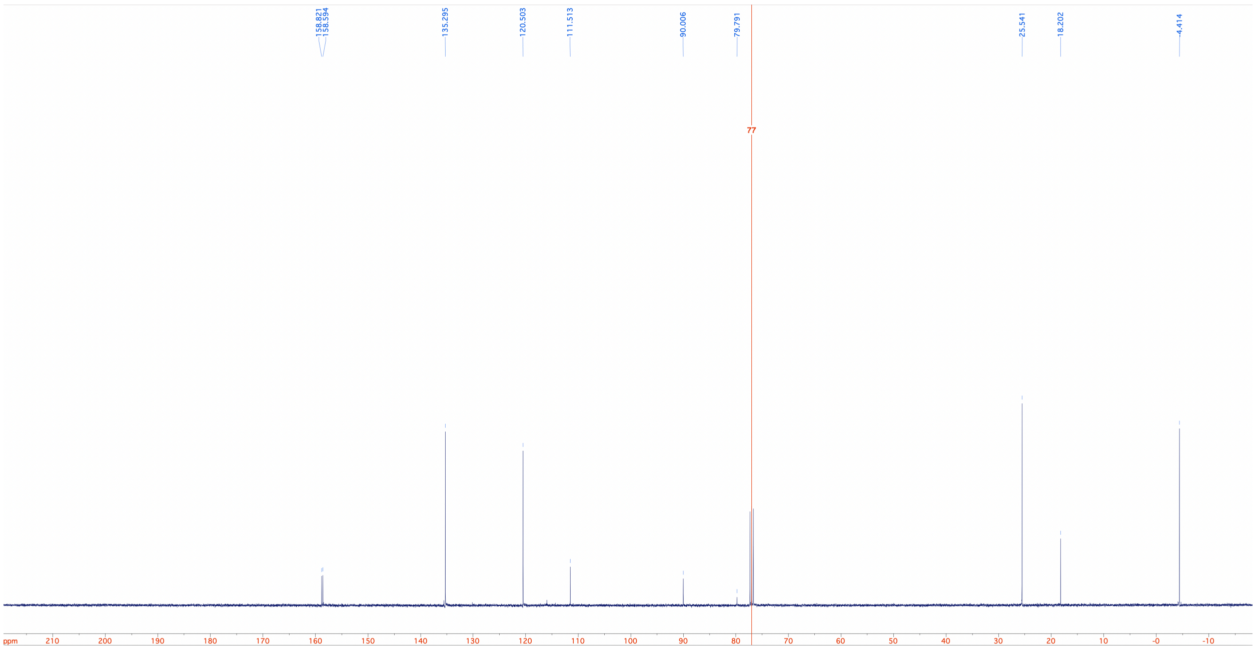
158.821
158.594
135.295
120.503
111.513
90.006
79.791
77
25.541
18.202
4.414
ppm

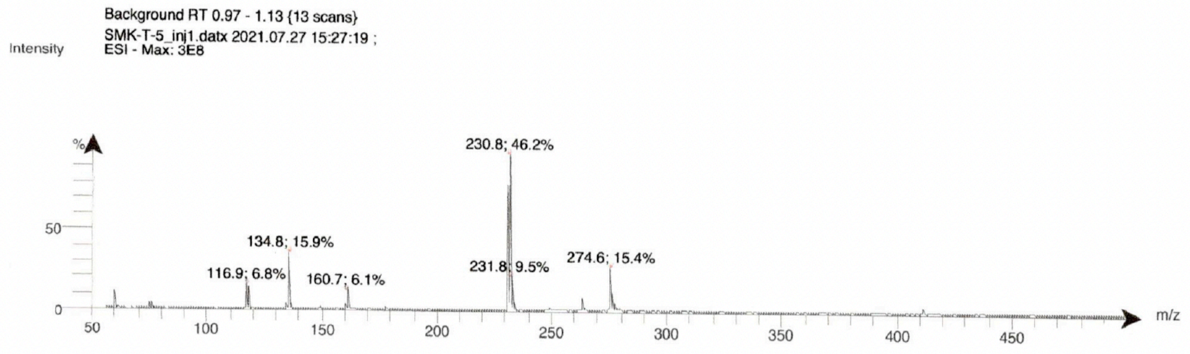
Background RT 0.97 - 1.13 {13 scans}
SMK-T-5_inj1.datx 2021.07.27 15:27:19 ;
ESI - Max: 3E8
Intensity
%
50
0
230.8; 46.2%
134.8; 15.9%
116.9; 6.8%
160.7; 6.1%
231.8; 9.5%
274.6; 15.4%
50
100
150
200
250
300
350
400
450
m/z

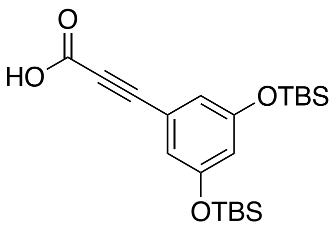

7C

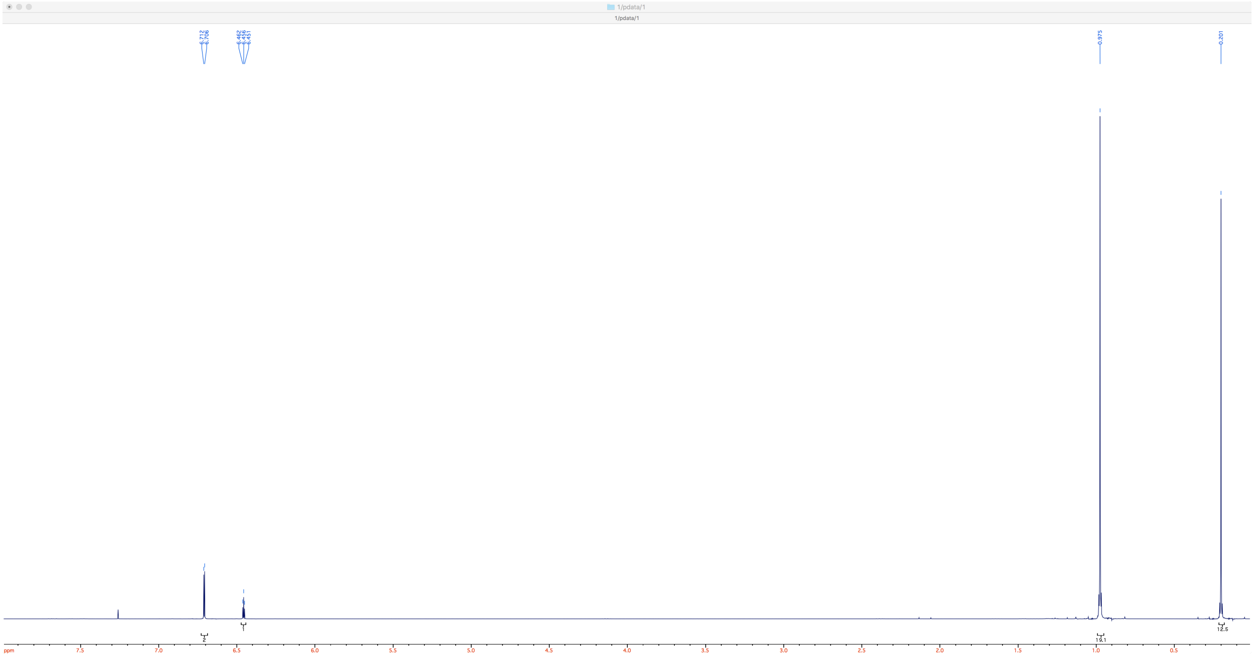

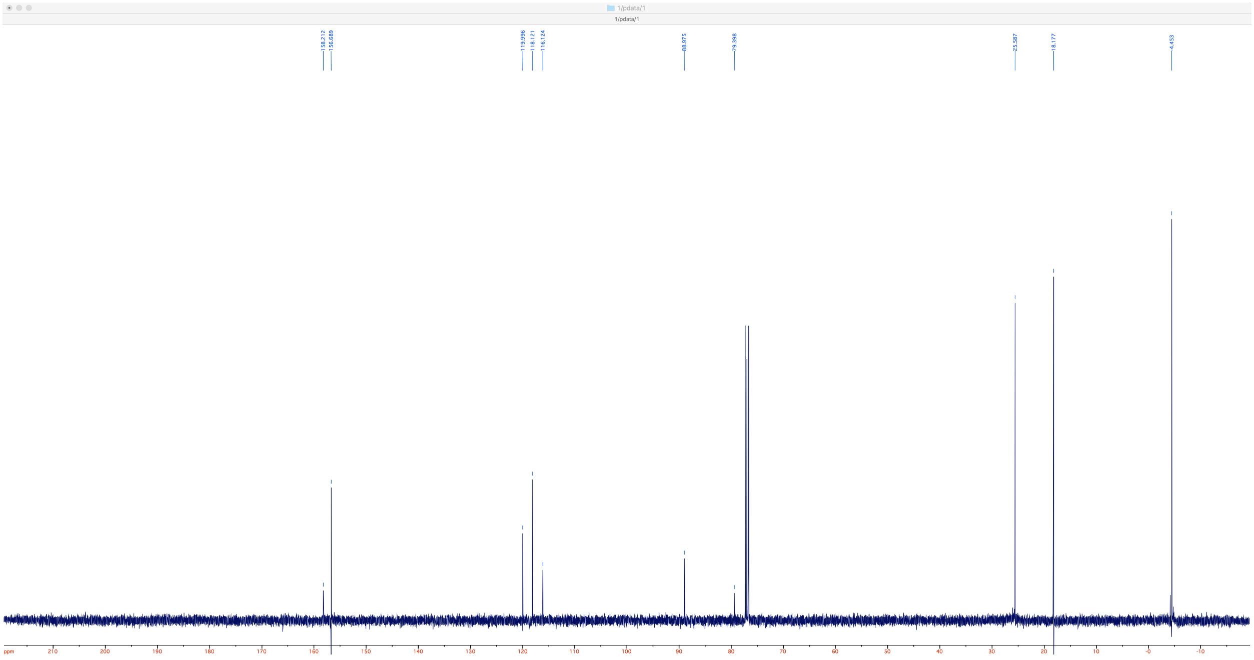

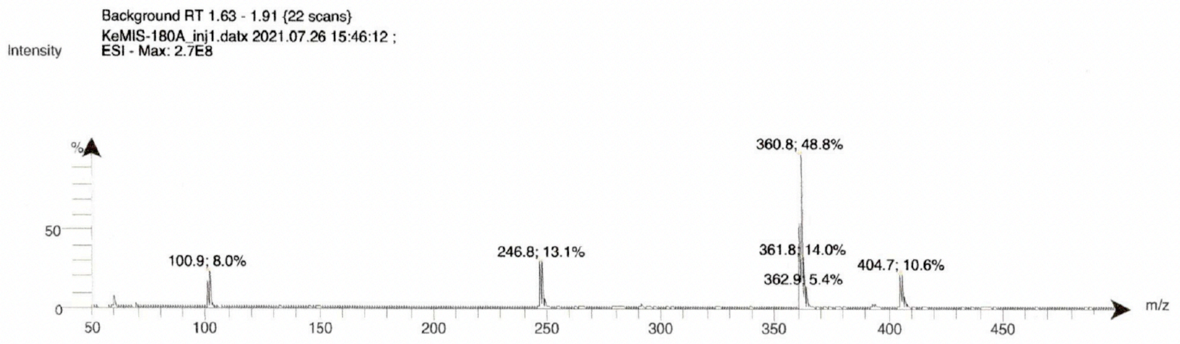

Background RT 1.63 - 1.91 {22 scans}
KeMIS-180A_inj1.datx 2021.07.26 15:46:12 ;
Intensity
ESI - Max: 2.7E8
%
50
0
100.9; 8.0%
246.8; 13.1%
360.8; 48.8%
361.8; 14.0%
362.9; 5.4%
404.7; 10.6%
50
100
150
200
250
300
350
400
450
m/z

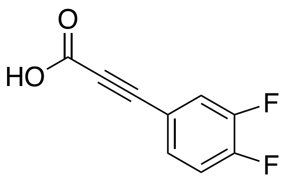

7D

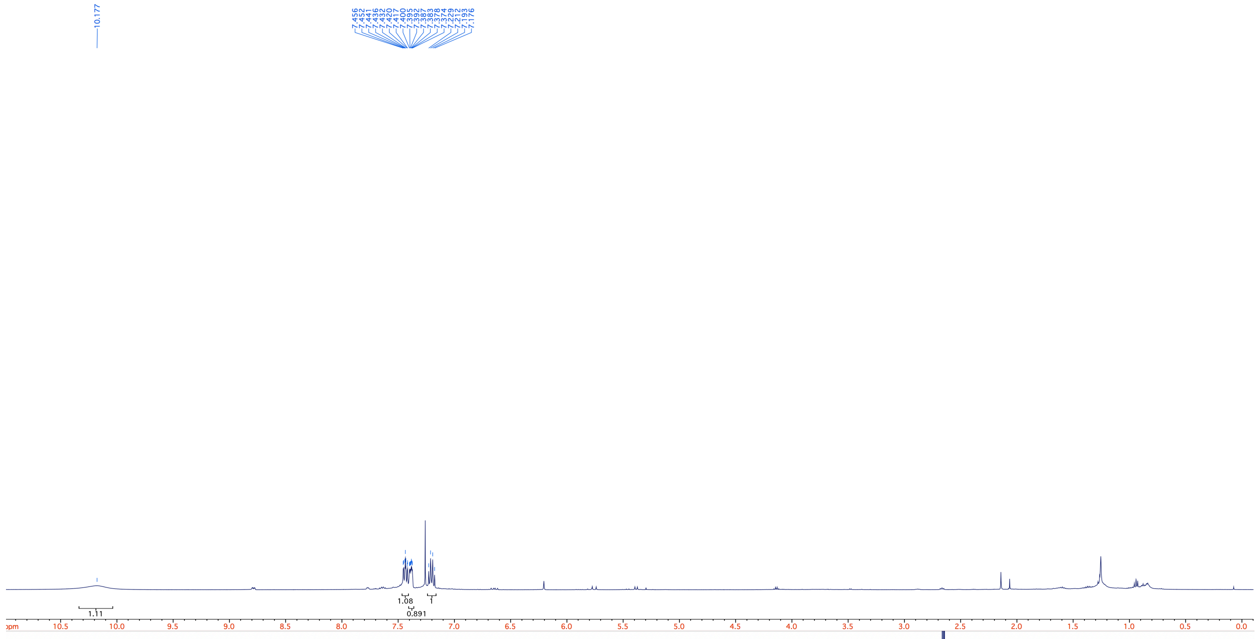

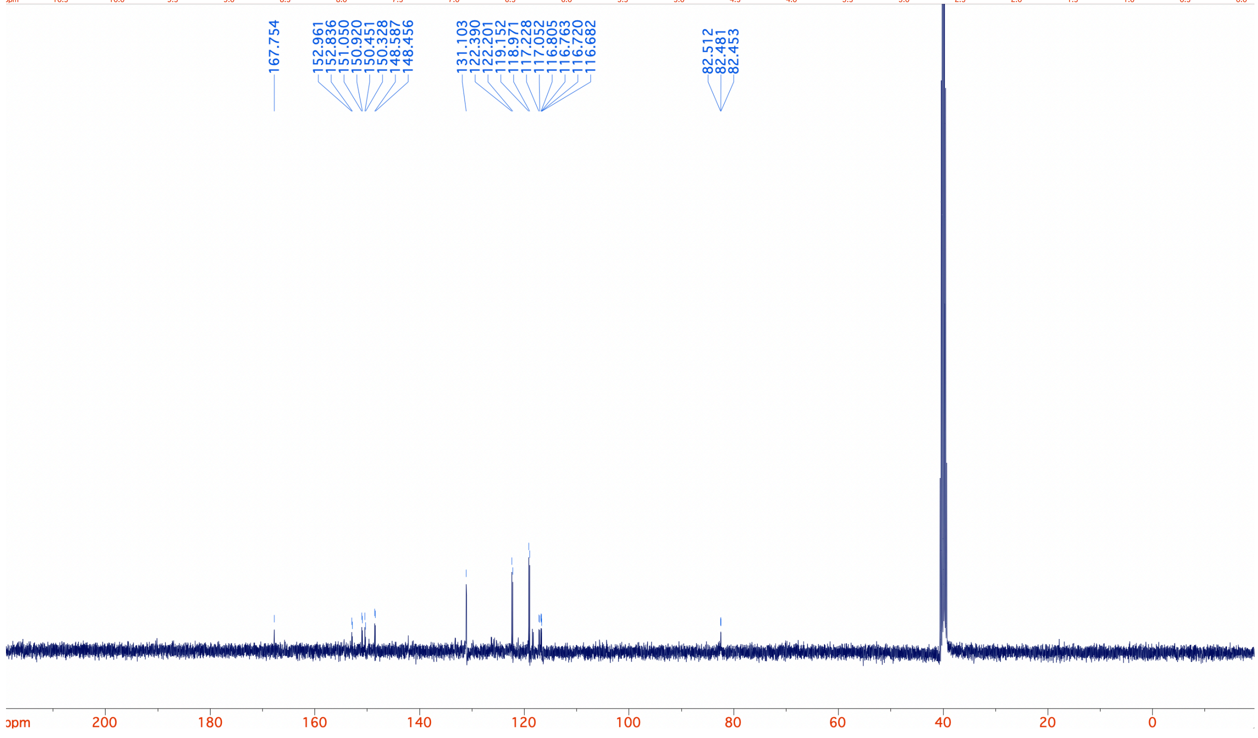

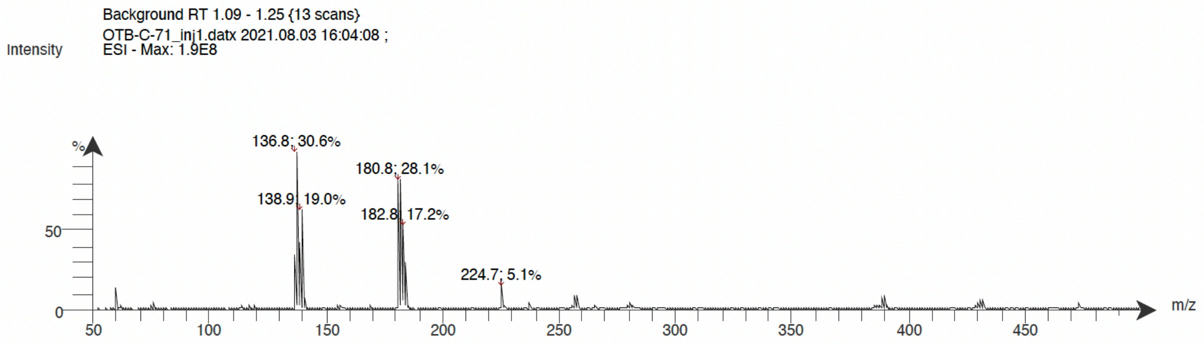

Background RT 1.09 - 1.25 {13 scans}
OTB-C-71_inj1.datx 2021.08.03 16:04:08 ;
Intensity
ESI - Max: 1.9E8
%
50
0
136.8; 30.6%
138.9; 19.0%
180.8; 28.1%
182.8; 17.2%
224.7; 5.1%
50
100
150
200
250
300
350
400
450
m/z

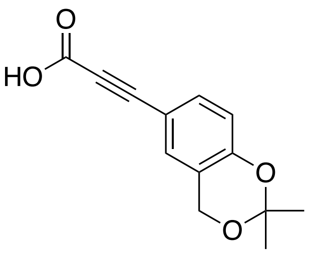
HO
O
O
O

7F

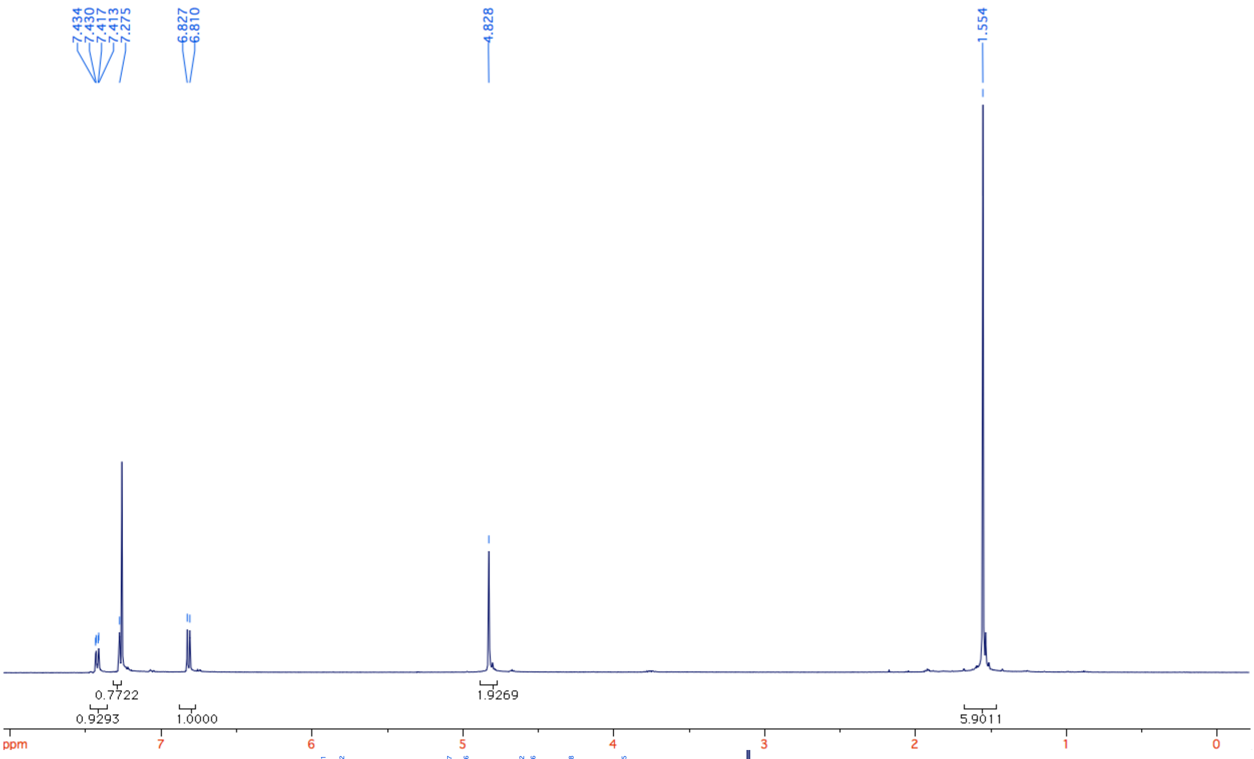
7.434
7.430
7.417
7.413
7.275
6.827
6.810
4.828
1.554
0.7722
0.9293
1.0000
1.9269
5.9011
ppm
7
6
5
4
3
2
1
0

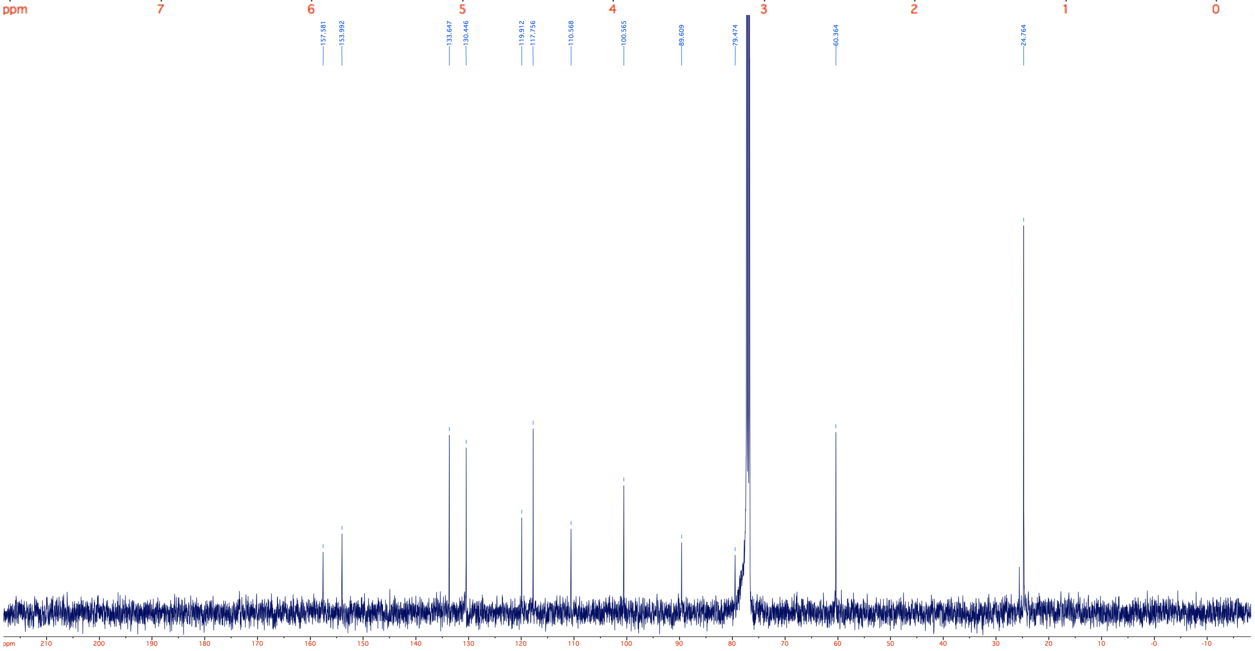
157.581
153.992
133.647
130.446
119.912
117.756
110.568
100.565
89.609
79.474
60.364
24.764

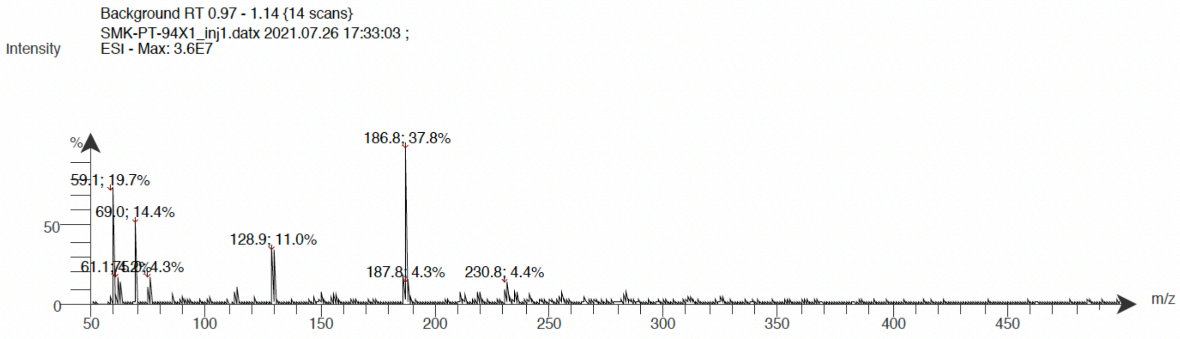
Background RT 0.97 - 1.14 {14 scans}
SMK-PT-94X1_inj1.datx 2021.07.26 17:33:03 ;
Intensity
ESI - Max: 3.6E7
%
186.8; 37.8%
59.1; 19.7%
69.0; 14.4%
128.9; 11.0%
4.3%
187.8; 4.3%
230.8; 4.4%
50
0
m/z
50
100
150
200
250
300
350
400
450

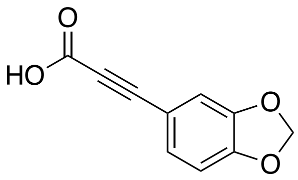
O
HO
O
O

7E

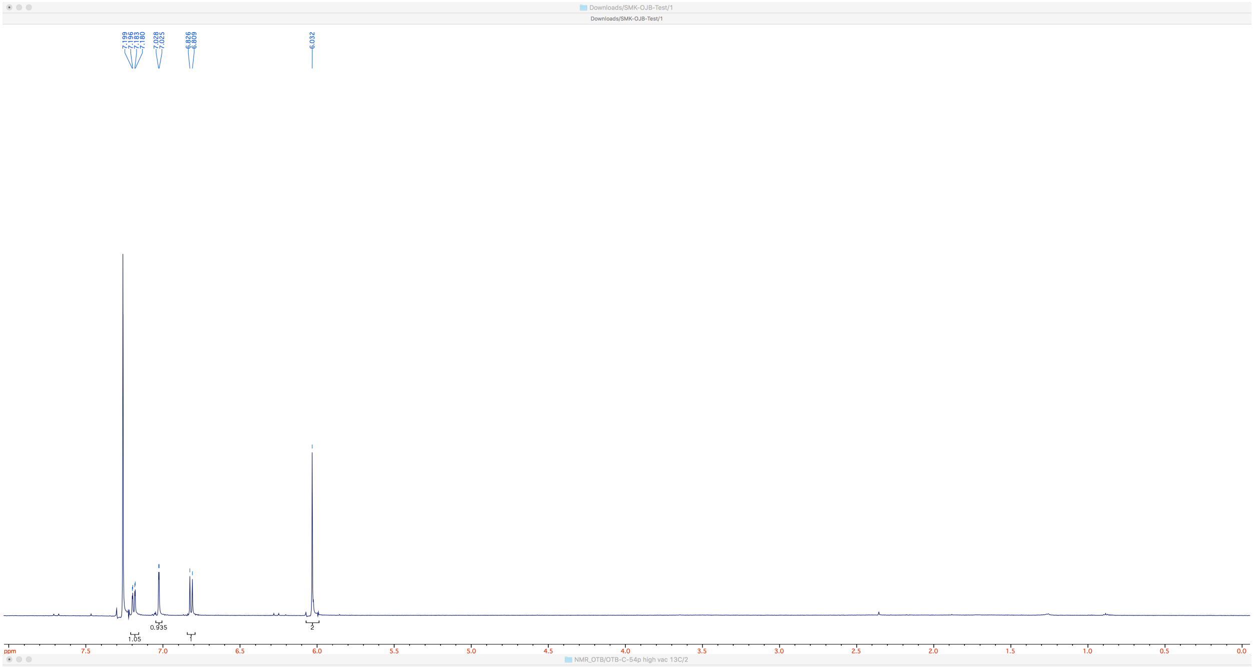
Downloads/SMK-OJB-Test/1
7.199
7.196
7.183
7.180
7.028
7.025
6.826
6.809
6.032
1.05
0.935
1
2
ppm
7.5
7.0
6.5
6.0
5.5
5.0
4.5
4.0
3.5
3.0
2.5
2.0
1.5
1.0
0.5
0.0

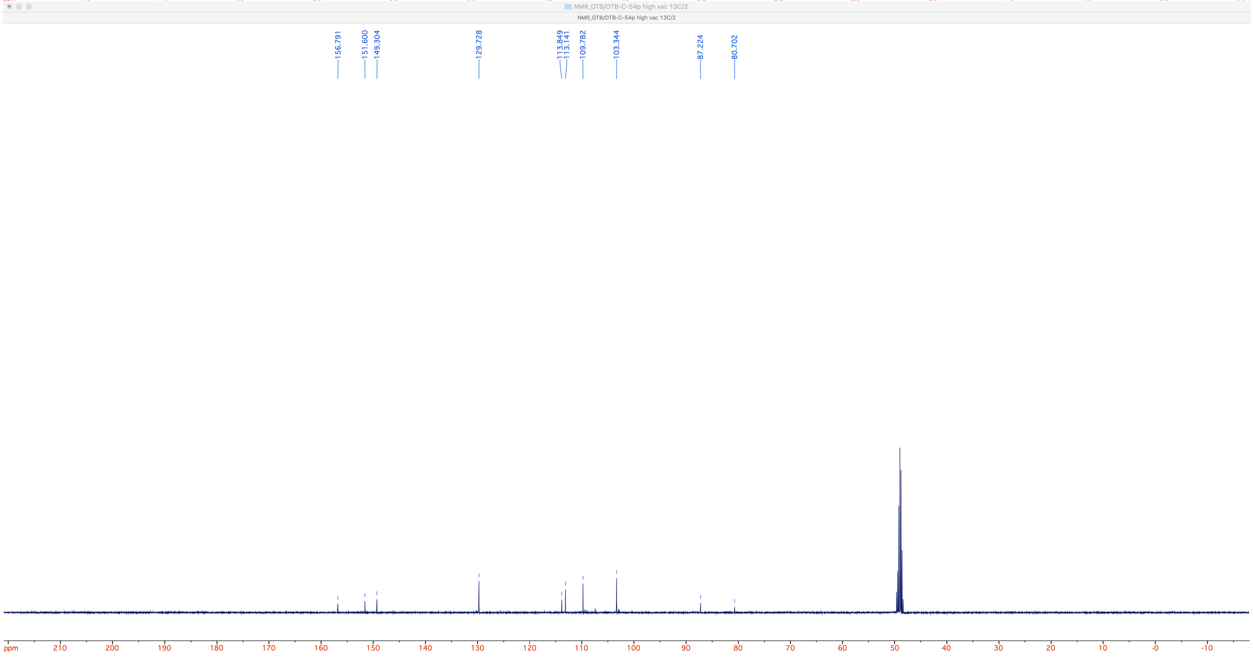
NMR_OTB/OTB-C-54p high vac 13C/2
156.791
151.600
149.304
129.728
113.849
113.141
109.782
103.344
87.224
80.702
ppm
210
200
190
180
170
160
150
140
130
120
110
100
90
80
70
60
50
40
30
20
10
-0
-10

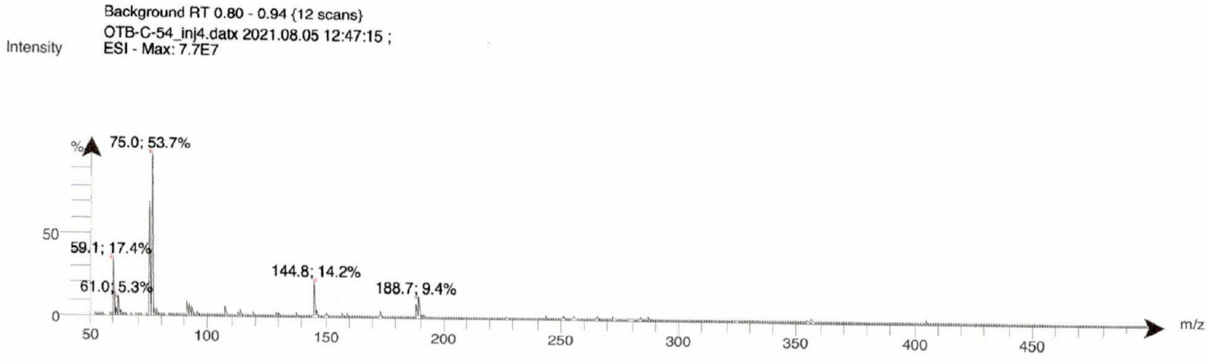

Background RT 0.80 - 0.94 {12 scans}
OTB-C-54_inj4.datx 2021.08.05 12:47:15 ;
ESI - Max: 7.7E7
Intensity
%
75.0; 53.7%
59.1; 17.4%
61.0; 5.3%
144.8; 14.2%
188.7; 9.4%
50
0
50
100
150
200
250
300
350
400
450
m/z

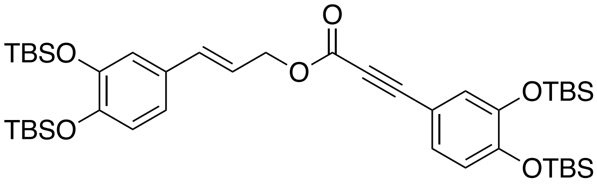

2AA

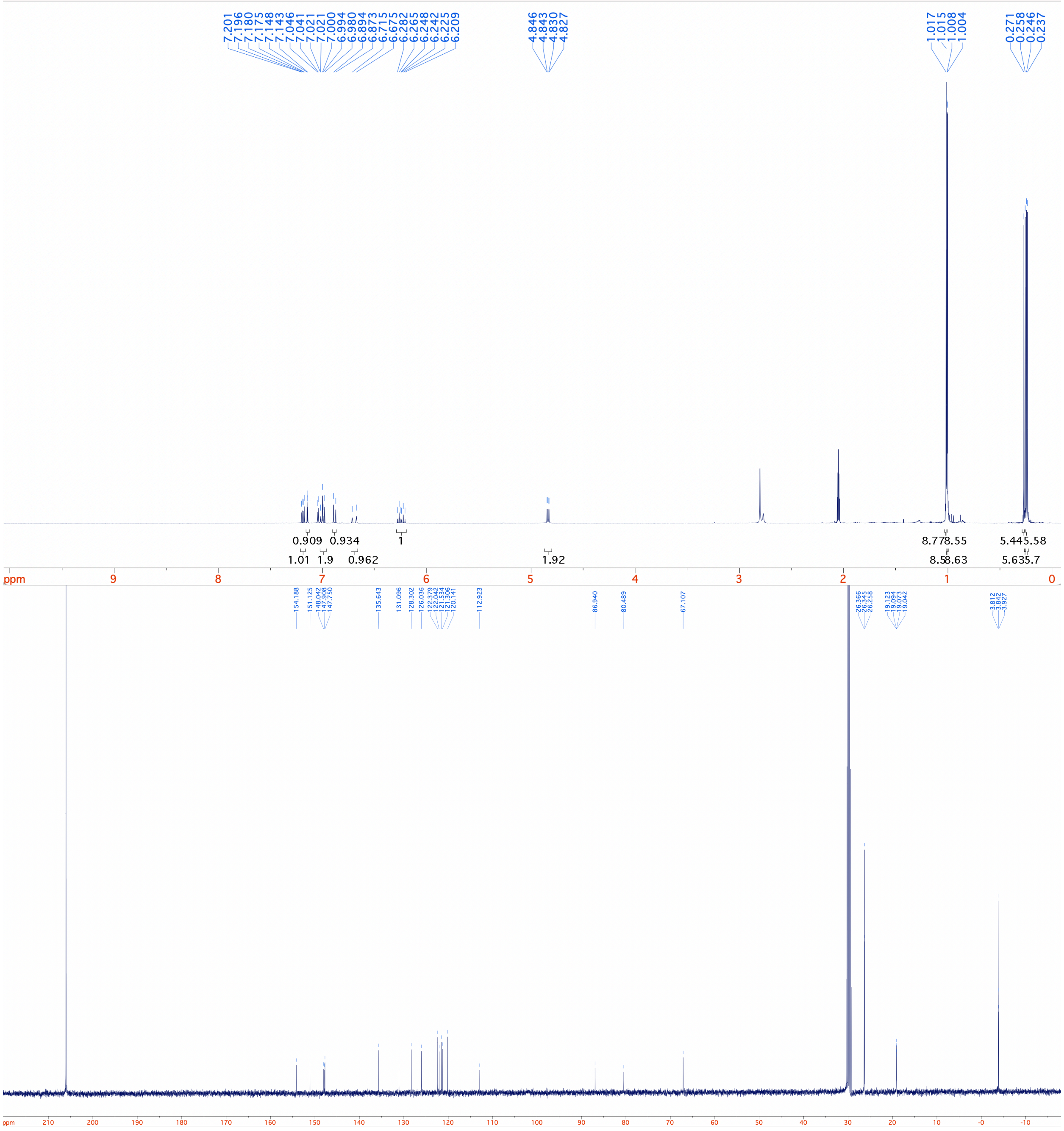

User Spectra

Fragmentor Voltage	Collision Energy	Ionization Mode
220	0	ESI

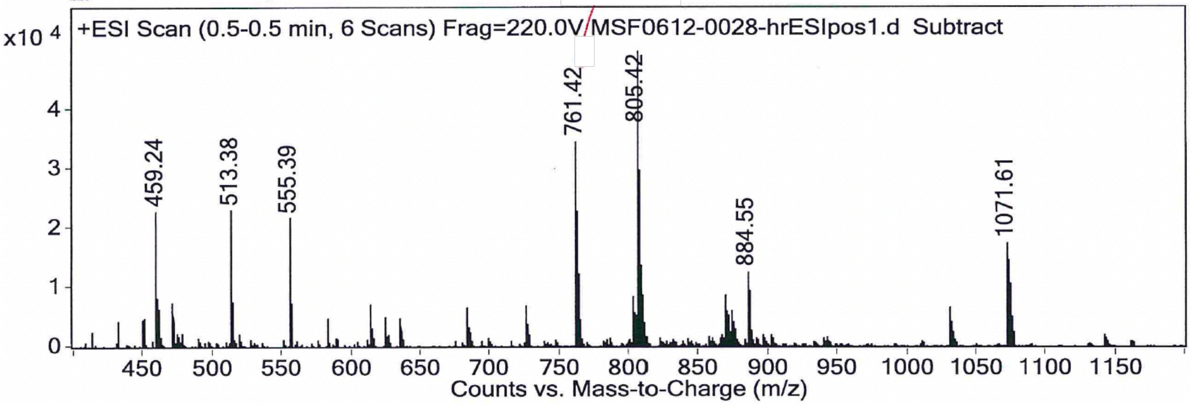

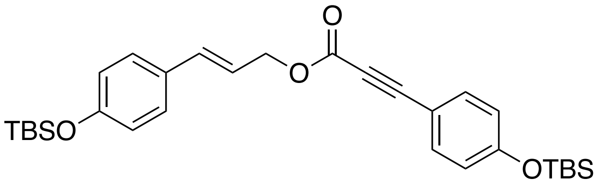

TBSO
O
O
OTBS

2BB

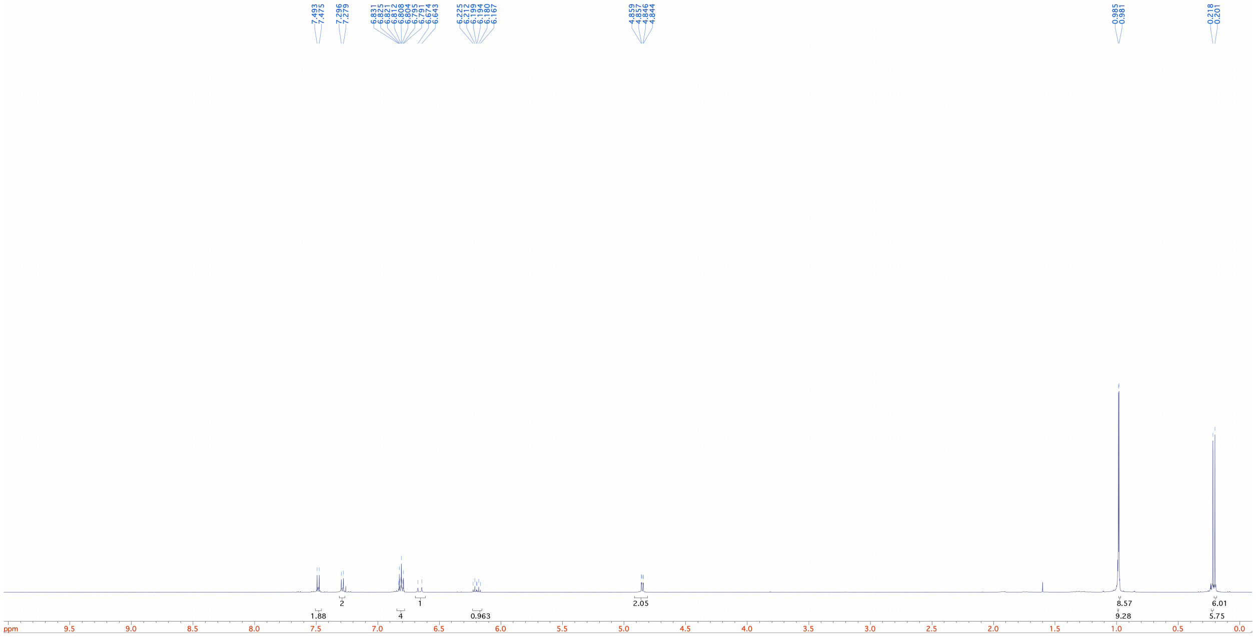

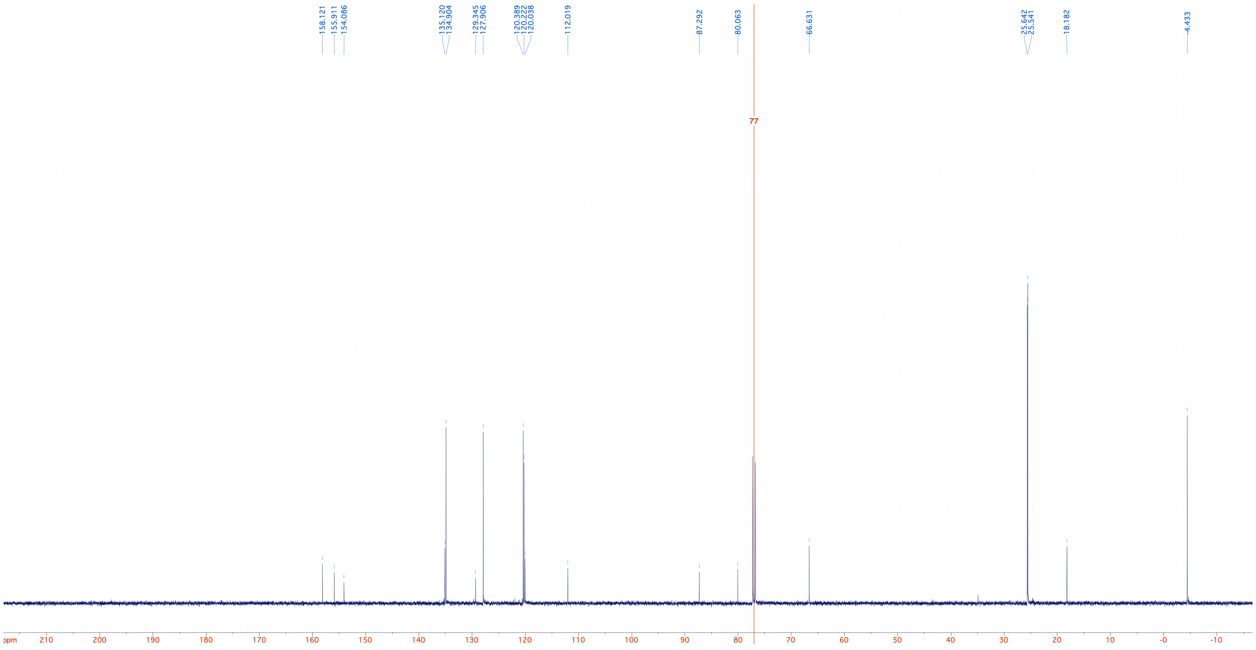

158.121
155.911
154.086
135.120
134.904
129.945
127.906
120.389
120.222
120.038
112.019
87.292
80.063
66.631
25.642
25.541
18.182
-4.433
77
ppm

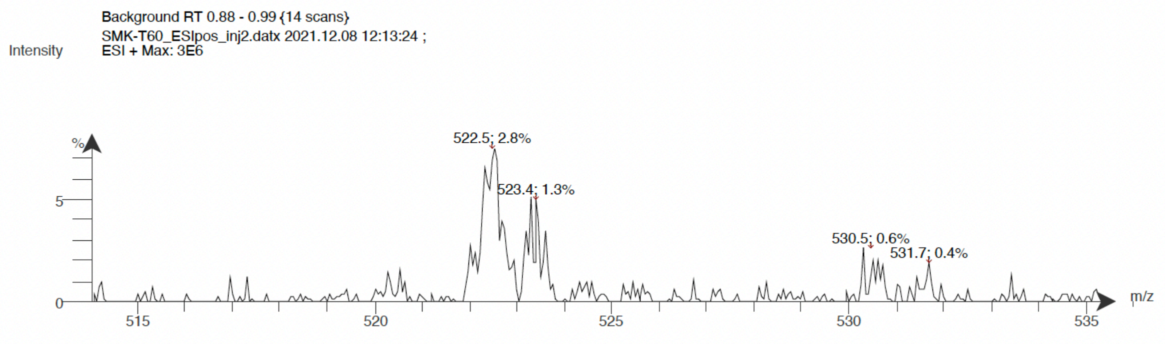
Background RT 0.88 - 0.99 {14 scans}
SMK-T60_ESIpos_inj2.datx 2021.12.08 12:13:24 ;
Intensity
ESI + Max: 3E6
%
5
0
522.5; 2.8%
523.4; 1.3%
530.5; 0.6%
531.7; 0.4%
515
520
525
530
535
m/z

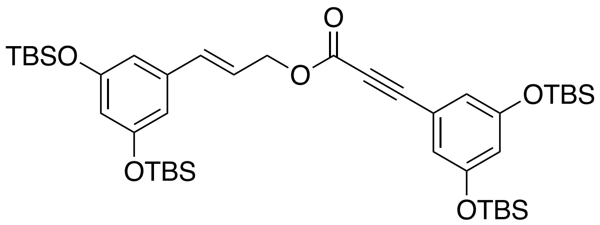

2CC

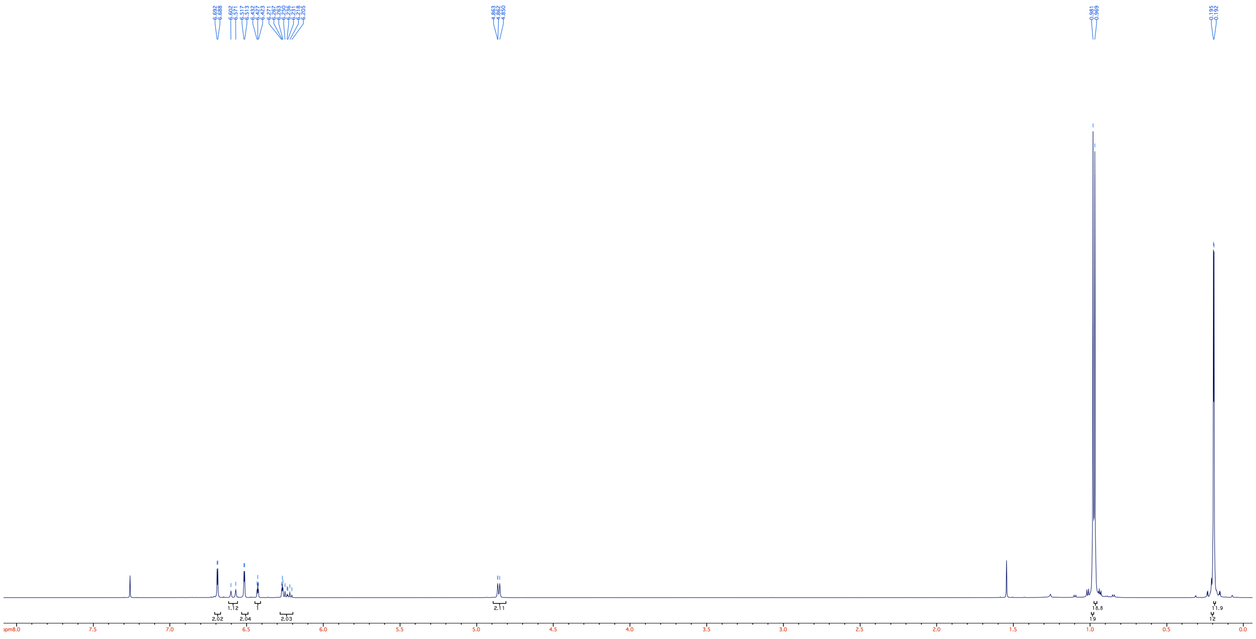

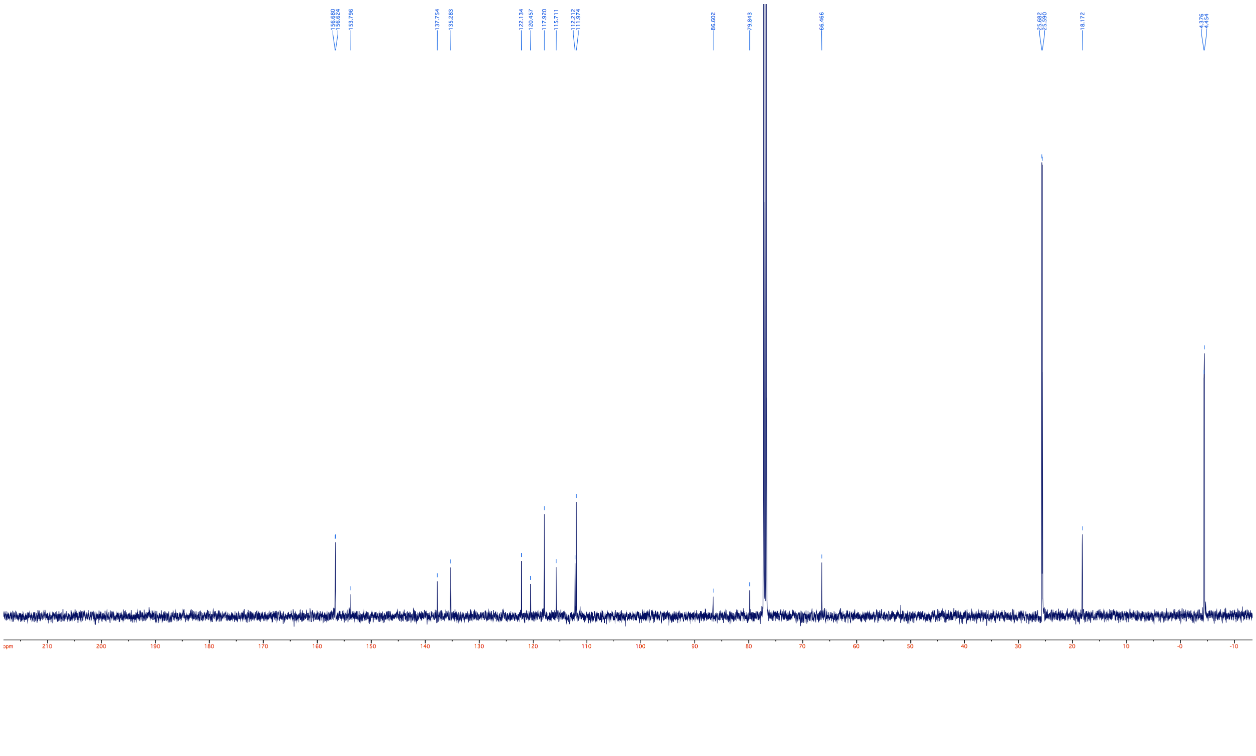

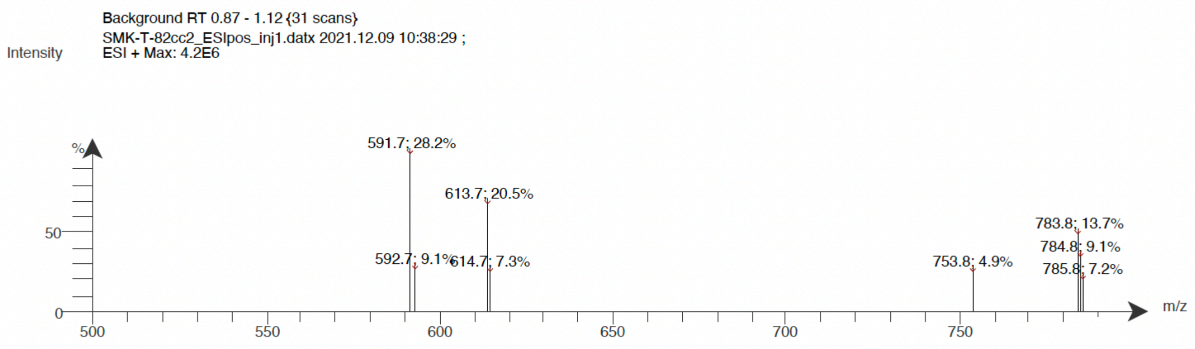
Background RT 0.87 - 1.12 {31 scans}
SMK-T-82cc2_ESIpos_inj1.datx 2021.12.09 10:38:29 ;
Intensity
ESI + Max: 4.2E6
%
50
0
591.7; 28.2%
613.7; 20.5%
592.7; 9.1%
614.7; 7.3%
753.8; 4.9%
783.8; 13.7%
784.8; 9.1%
785.8; 7.2%
500
550
600
650
700
750
m/z

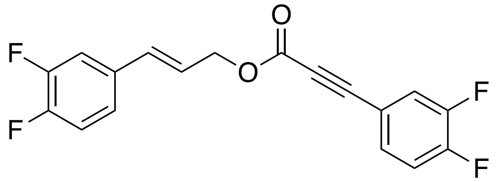

2DD

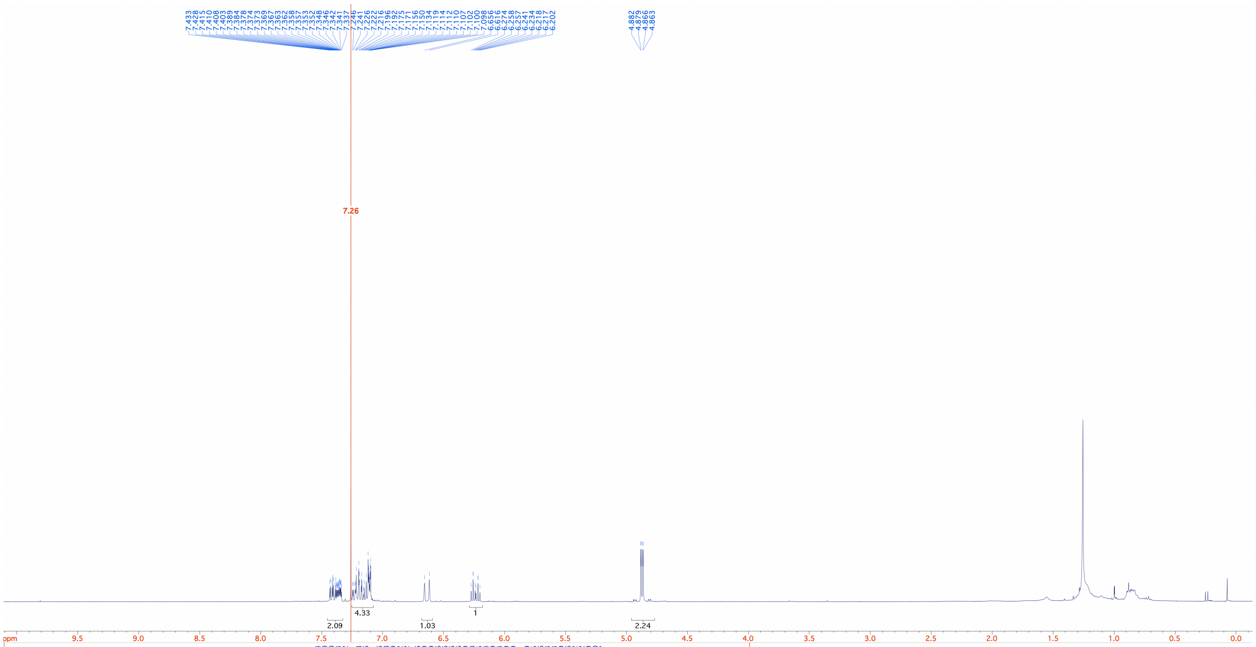

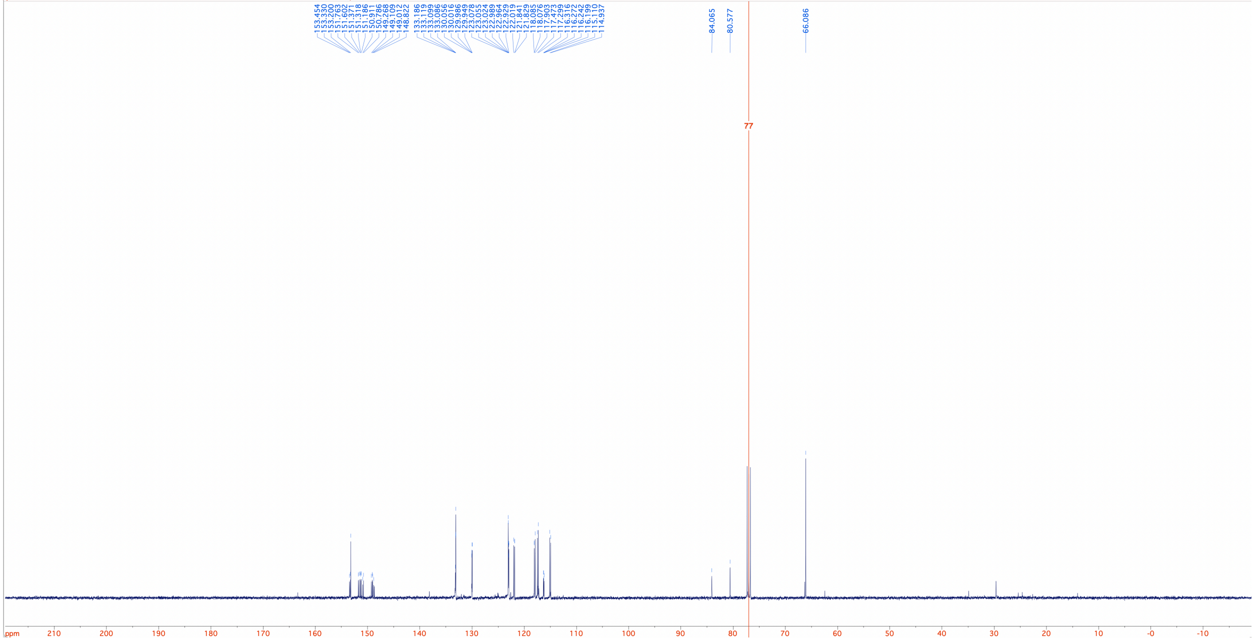

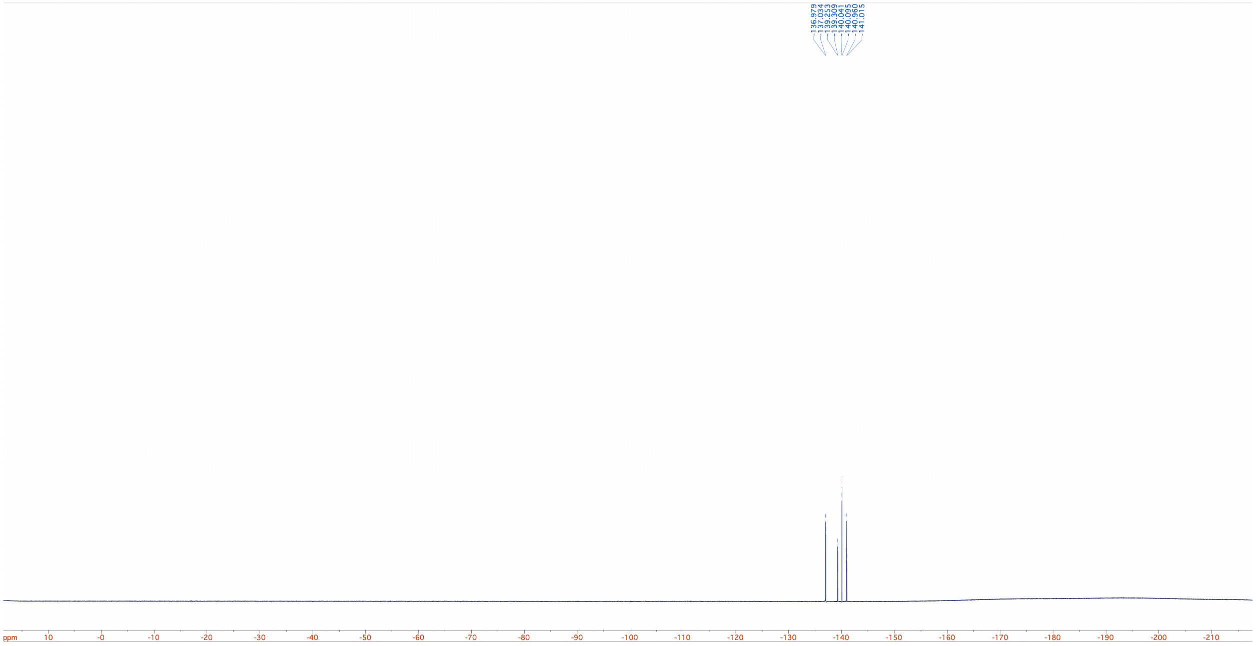
-136.979
-137.034
-139.253
-139.309
-140.041
-140.095
-140.960
-141.015
ppm
10
-0
-10
-20
-30
-40
-50
-60
-70
-80
-90
-100
-110
-120
-130
-140
-150
-160
-170
-180
-190
-200
-210

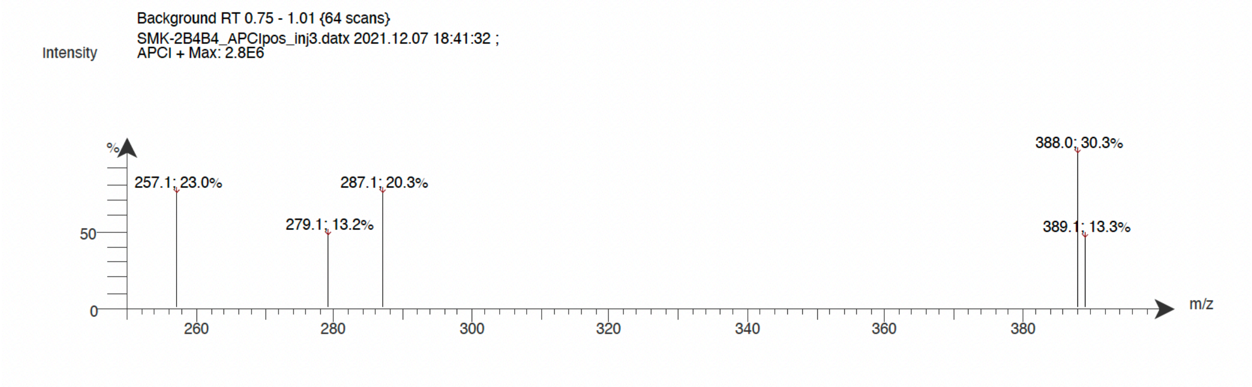
Background RT 0.75 - 1.01 (64 scans)
SMK-2B4B4_APCIpos_inj3.datx 2021.12.07 18:41:32 ;
APCI + Max: 2.8E6
Intensity
%
50
0
257.1; 23.0%
279.1; 13.2%
287.1; 20.3%
388.0; 30.3%
389.1; 13.3%
260
280
300
320
340
360
380
m/z

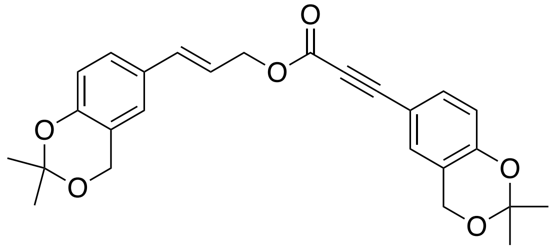

2FF

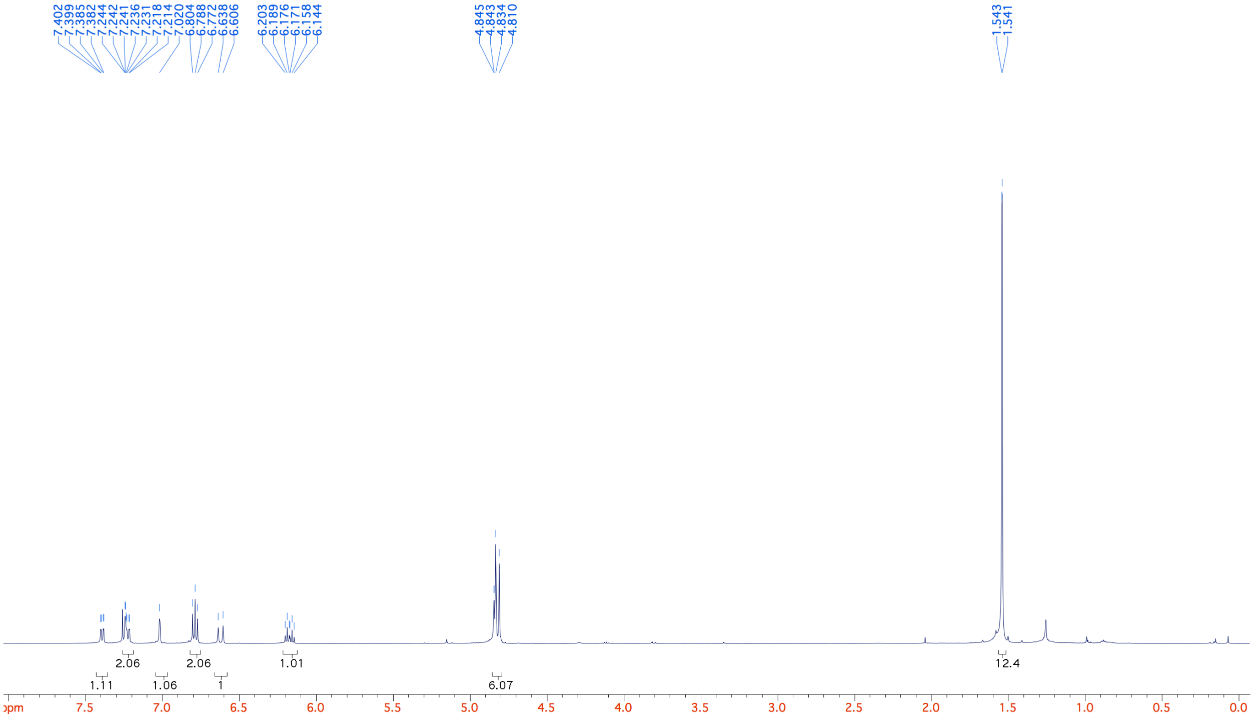
7.402
7.399
7.385
7.382
7.244
7.242
7.241
7.236
7.231
7.218
7.214
7.020
6.804
6.788
6.772
6.638
6.606
6.203
6.189
6.176
6.171
6.158
6.144
4.845
4.843
4.834
4.810
1.543
1.541
2.06
2.06
1.01
12.4
1.11
1.06
1
6.07
ppm
7.5
7.0
6.5
6.0
5.5
5.0
4.5
4.0
3.5
3.0
2.5
2.0
1.5
1.0
0.5
0.0

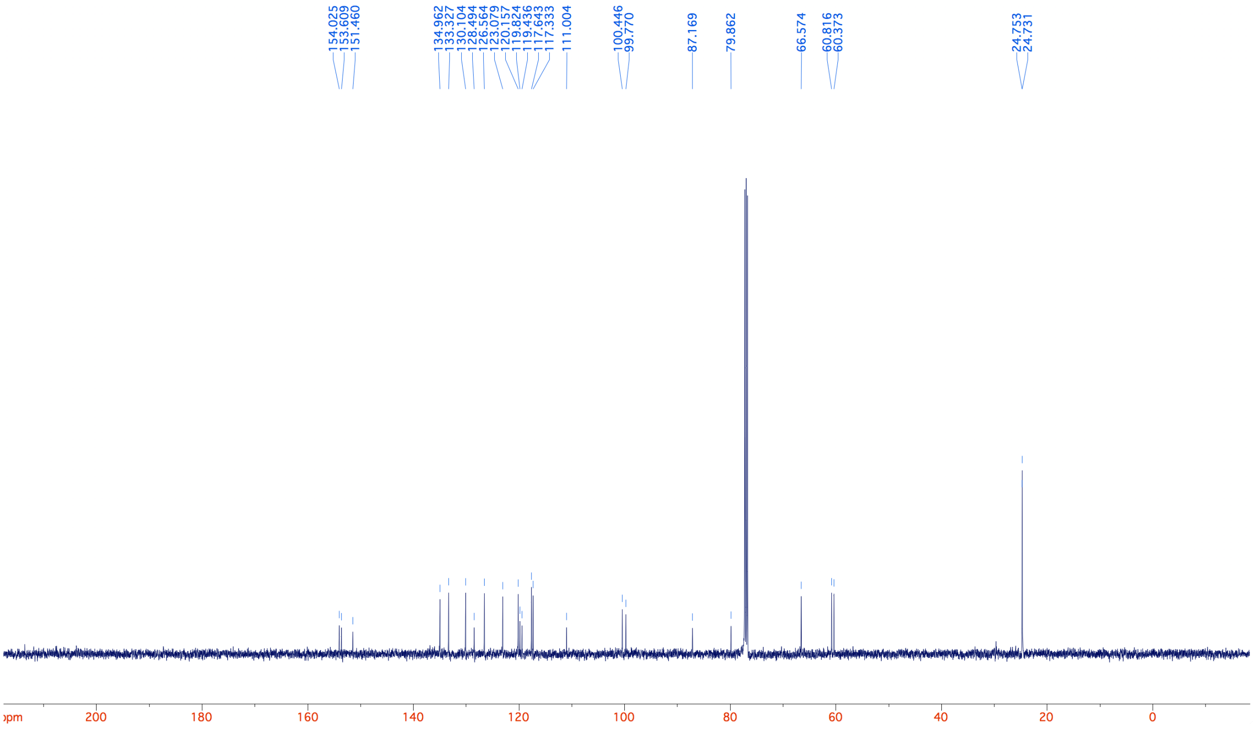
154.025
153.609
151.460
134.962
133.327
130.104
128.494
126.564
123.079
120.157
119.824
119.446
117.643
117.333
111.004
100.446
99.770
87.169
79.862
66.574
60.816
60.373
24.753
24.731
ppm
200
180
160
140
120
100
80
60
40
20
0

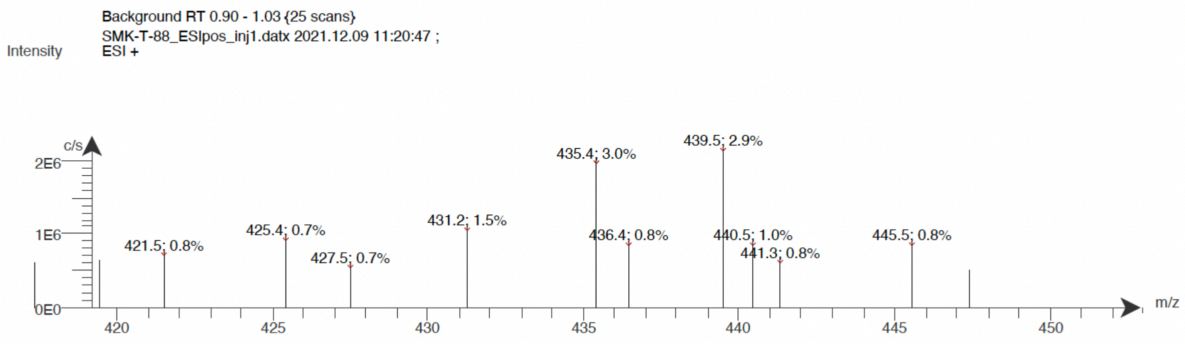
Background RT 0.90 - 1.03 {25 scans}
SMK-T-88_ESIpos_inj1.datx 2021.12.09 11:20:47 ;
Intensity
ESI +
c/s
2E6
1E6
0E0
421.5; 0.8%
425.4; 0.7%
427.5; 0.7%
431.2; 1.5%
435.4; 3.0%
436.4; 0.8%
439.5; 2.9%
440.5; 1.0%
441.3; 0.8%
445.5; 0.8%
420
425
430
435
440
445
450
m/z

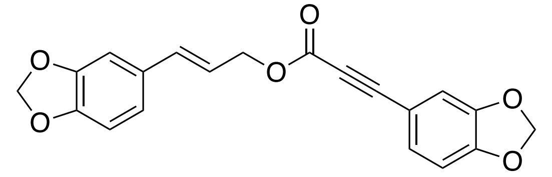
O
O
O
O
O
O

2EE

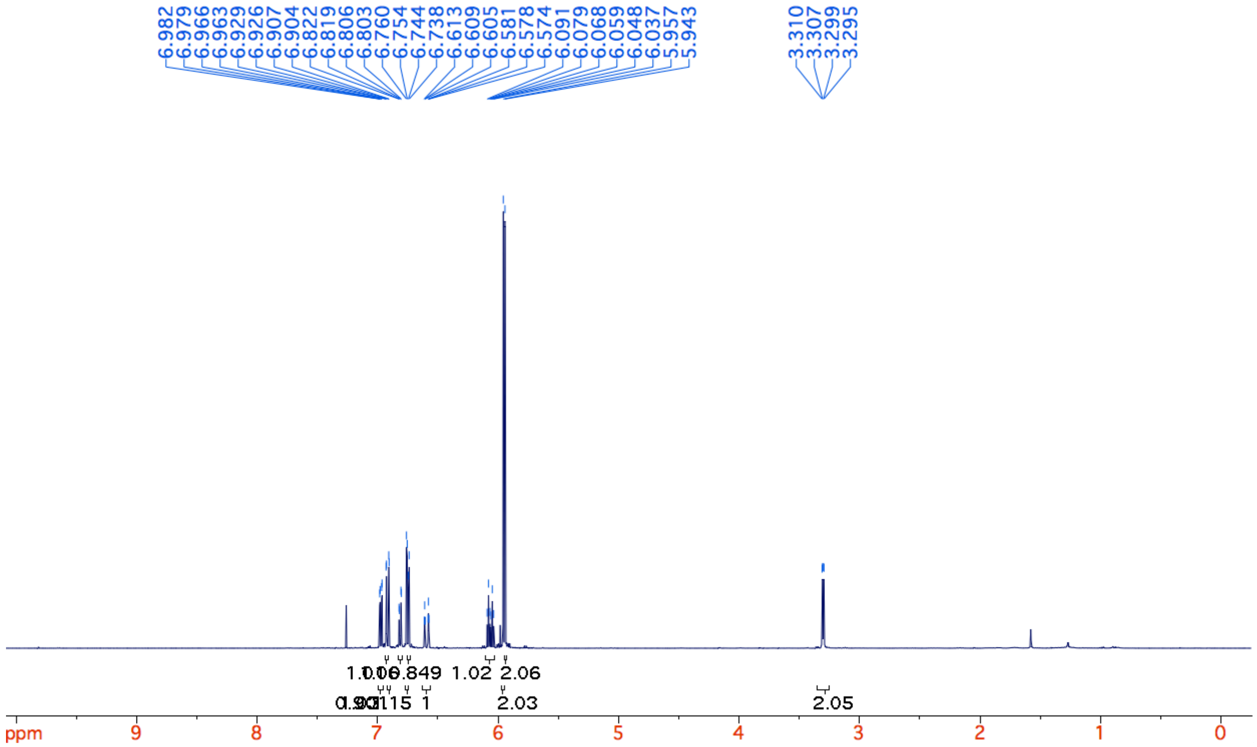
6.982
6.979
6.966
6.963
6.929
6.926
6.907
6.904
6.822
6.819
6.806
6.803
6.760
6.754
6.744
6.738
6.613
6.609
6.605
6.581
6.578
6.574
6.091
6.079
6.068
6.059
6.048
6.037
5.957
5.943
3.310
3.307
3.299
3.295
1.02
2.06
2.03
2.05
ppm
9
8
7
6
5
4
3
2
1
0

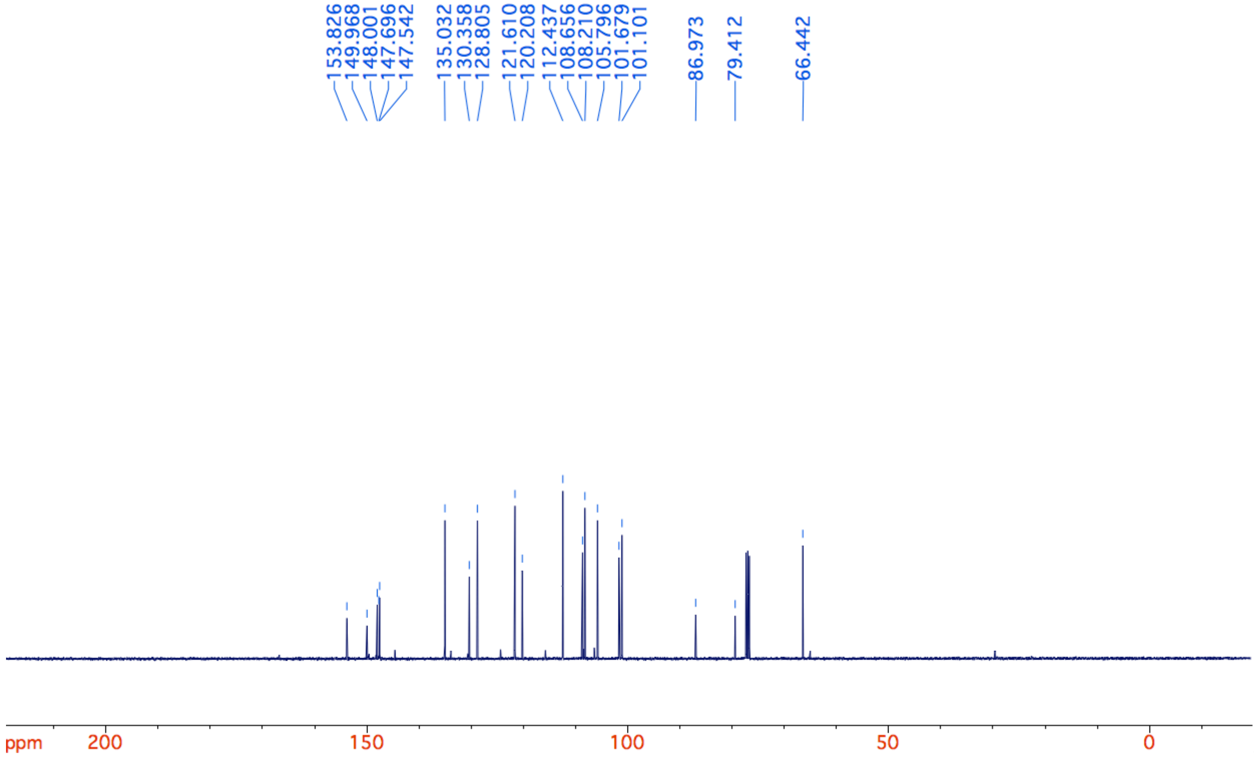
153.826
149.968
148.001
147.696
147.542
135.032
130.358
128.805
121.610
120.208
112.437
108.656
108.210
105.796
101.679
101.101
86.973
79.412
66.442
ppm
200
150
100
50
0

Background RT 1.07 - 1.32 {36 scans}
OTB-C81p_ESIpos_inj2.datx 2021.12.09 14:21:13 ;
ESI +

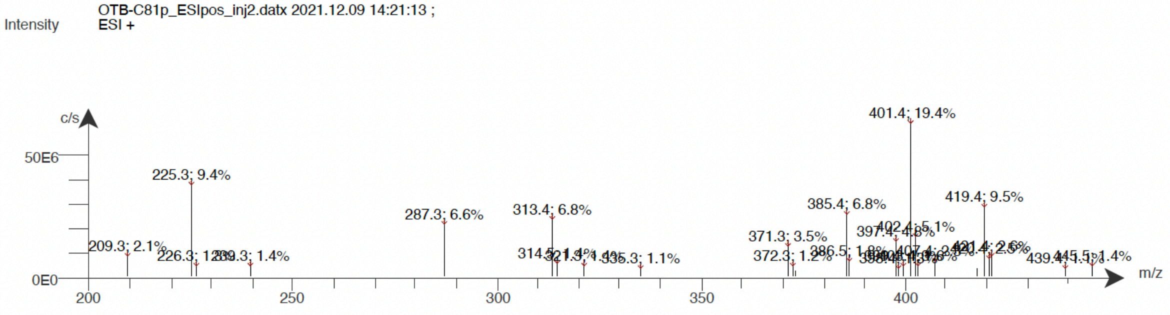

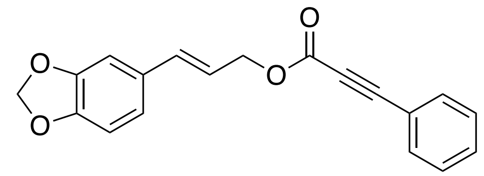

2EG

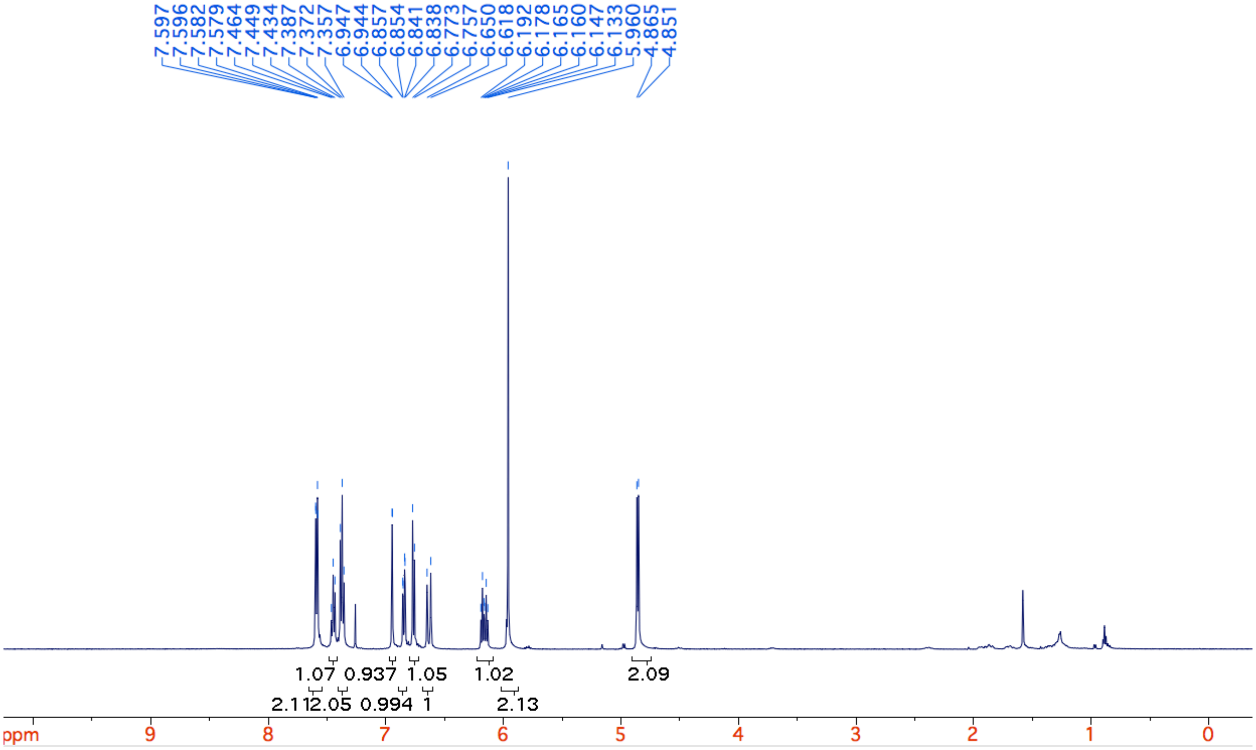
7.597 7.596 7.582 7.579 7.464 7.449 7.434 7.387 7.372 7.357 6.947 6.944 6.857 6.854 6.841 6.838 6.773 6.757 6.650 6.618 6.192 6.178 6.165 6.160 6.147 6.133 5.960 4.865 4.851
1.07 0.937 1.05 1.02 2.09
2.11 2.05 0.994 1 2.13
ppm 9 8 7 6 5 4 3 2 1 0

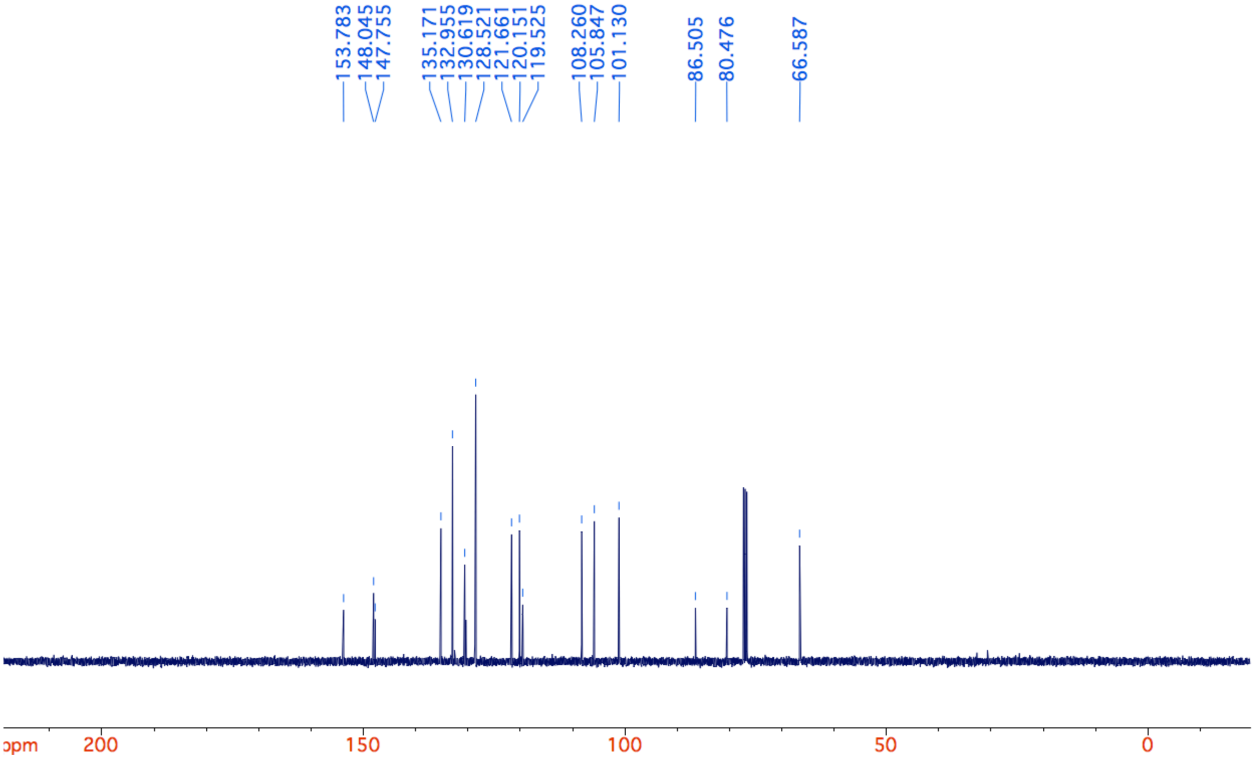
153.783 148.045 147.755 135.171 132.955 130.619 128.521 121.661 120.151 119.525 108.260 105.847 101.130 86.505 80.476 66.587
ppm 200 150 100 50 0

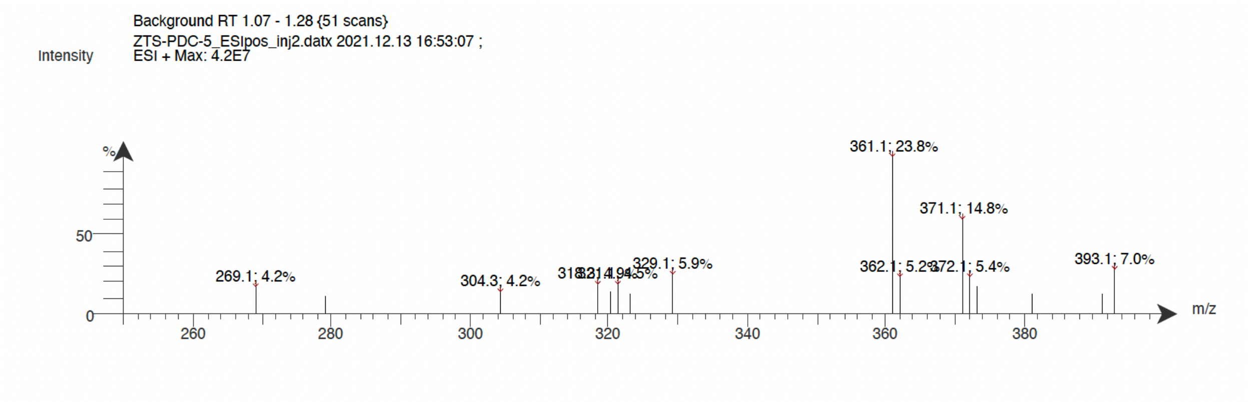
Background RT 1.07 - 1.28 {51 scans}
ZTS-PDC-5_ESIpos_inj2.datx 2021.12.13 16:53:07 ;
ESI + Max: 4.2E7
Intensity
%
50
0
269.1; 4.2%
304.3; 4.2%
329.1; 5.9%
361.1; 23.8%
371.1; 14.8%
362.1; 5.2%
372.1; 5.4%
393.1; 7.0%
260
280
300
320
340
360
380
m/z

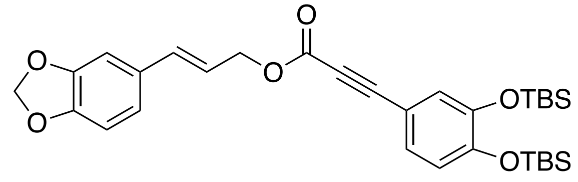
O
O
O
O
O
OTBS
OTBS

2EA

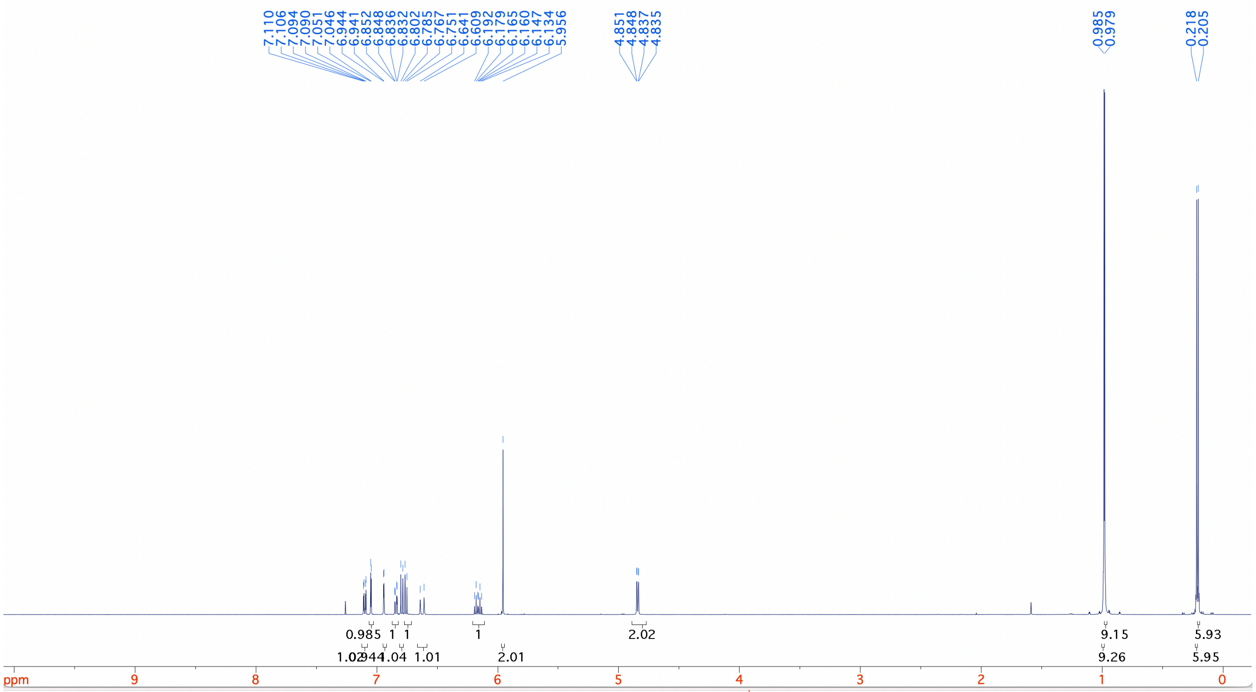
7.110
7.106
7.094
7.090
7.051
7.046
6.944
6.941
6.852
6.848
6.836
6.832
6.802
6.785
6.767
6.751
6.641
6.609
6.192
6.179
6.165
6.160
6.147
6.134
5.956
4.851
4.848
4.837
4.835
0.985
0.979
0.218
0.205
0.985
1
1
1
2.02
9.15
5.93
1.02
0.944
1.04
1.01
2.01
9.26
5.95
ppm
9
8
7
6
5
4
3
2
1
0

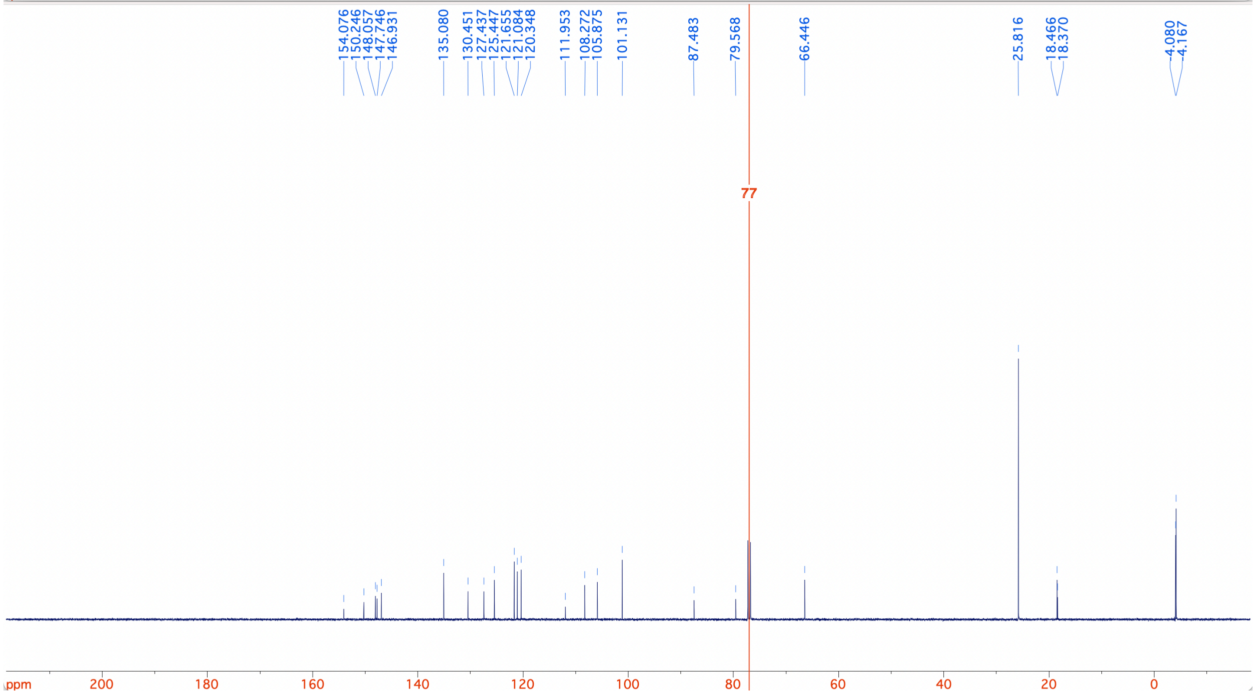
154.076
150.246
148.057
147.746
146.931
135.080
130.451
127.437
125.447
121.655
121.084
120.348
111.953
108.272
105.875
101.131
87.483
79.568
66.446
25.816
18.466
18.370
-4.080
-4.167
77
ppm
200
180
160
140
120
100
80
60
40
20
0

TIC
SMK-T-58_ESIpos_inj2.datx 2021.12.09 10:27:08 ;
Intensity ESI +

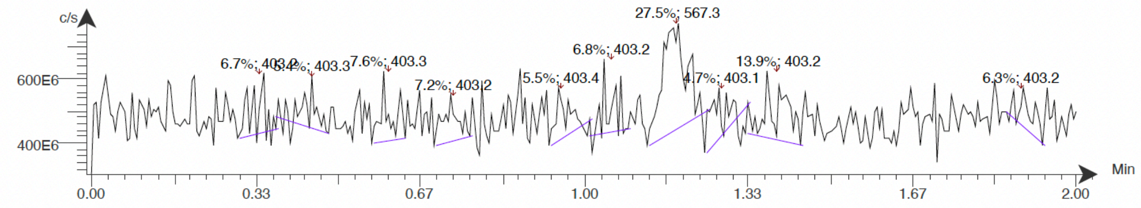

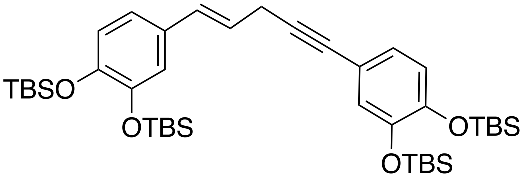
TBSO
OTBS
OTBS
OTBS

1'AA

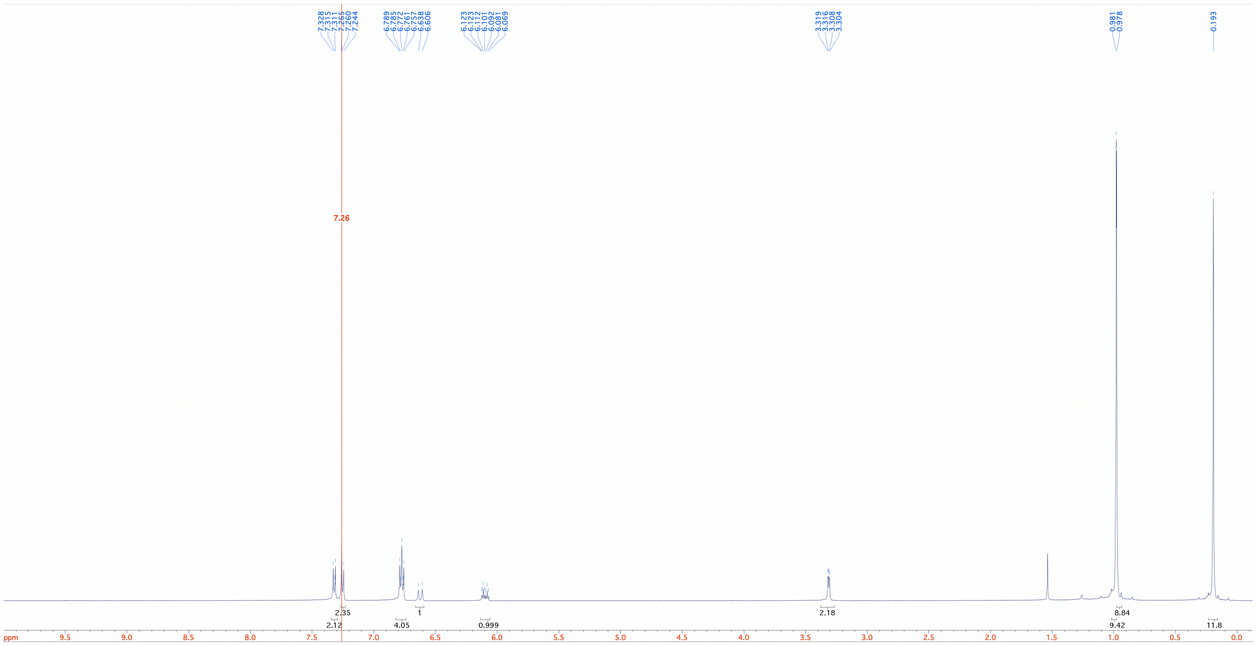
7.328
7.315
7.311
7.265
7.260
7.244
6.789
6.785
6.772
6.761
6.757
6.638
6.606
6.123
6.123
6.112
6.101
6.092
6.081
6.069
3.319
3.316
3.308
3.304
0.981
0.978
0.193
7.26
2.35
2.12
4.05
1
0.999
2.18
8.84
9.42
11.8
ppm
9.5
9.0
8.5
8.0
7.5
7.0
6.5
6.0
5.5
5.0
4.5
4.0
3.5
3.0
2.5
2.0
1.5
1.0
0.5
0.0

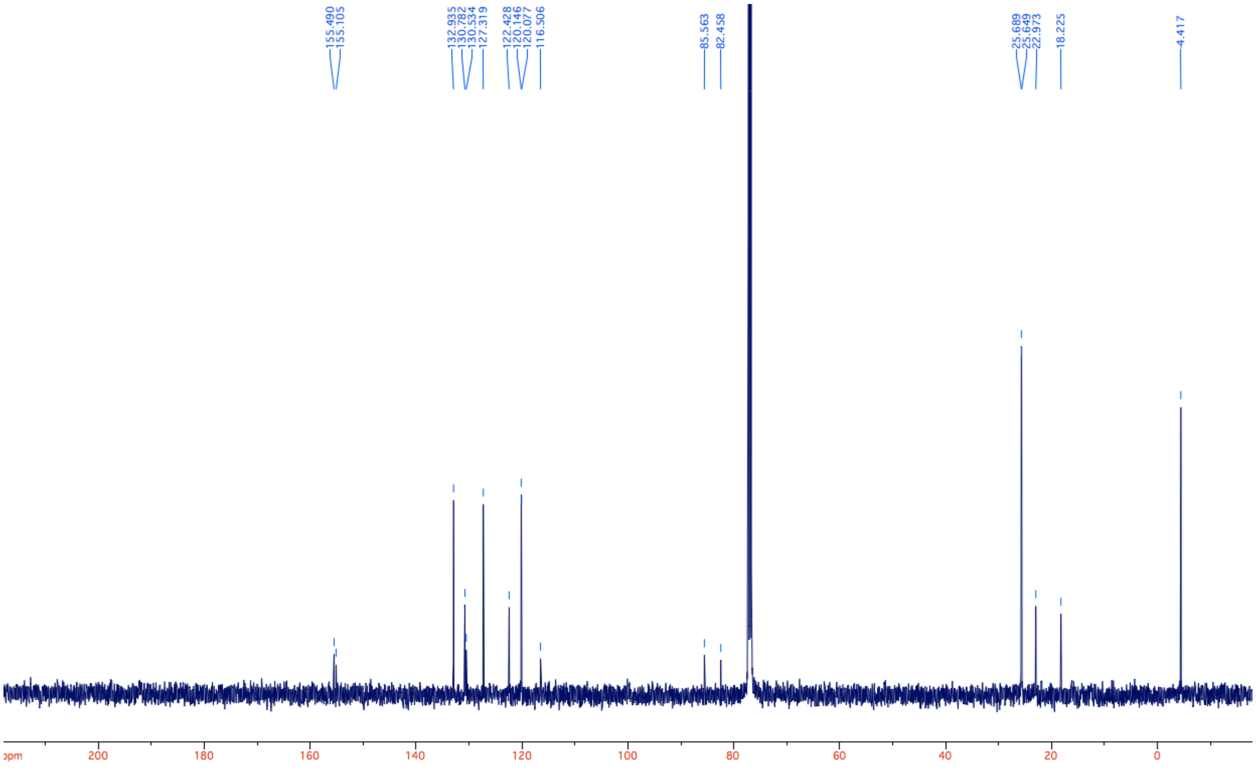
155.490
155.105
132.935
130.782
130.534
127.319
122.428
120.146
120.077
116.506
85.563
82.458
25.689
25.649
22.973
18.225
-4.417
200
180
160
140
120
100
80
60
40
20
0

User Spectra

Fragmentor Voltage	Collision Energy	Ionization Mode
220	0	ESI

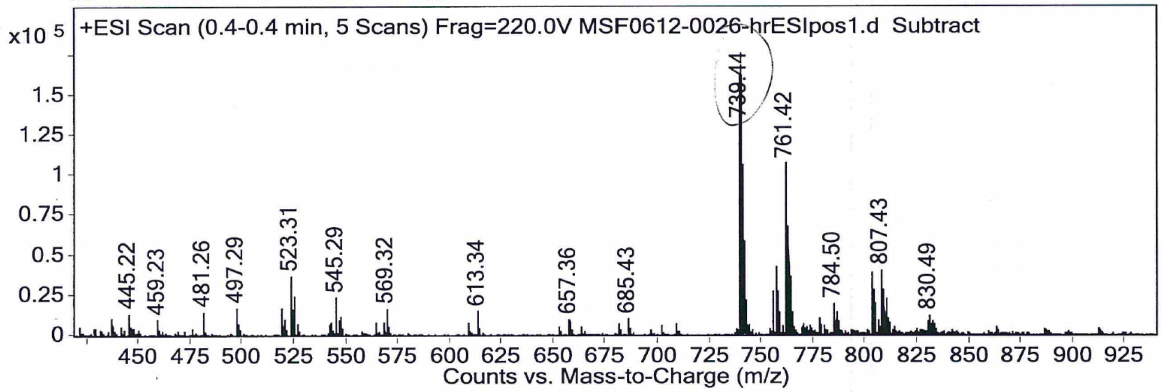

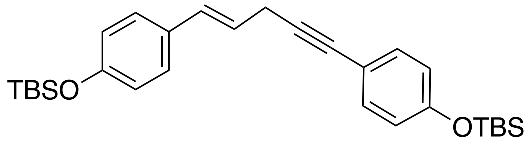
TBSO
OTBS

1’BB

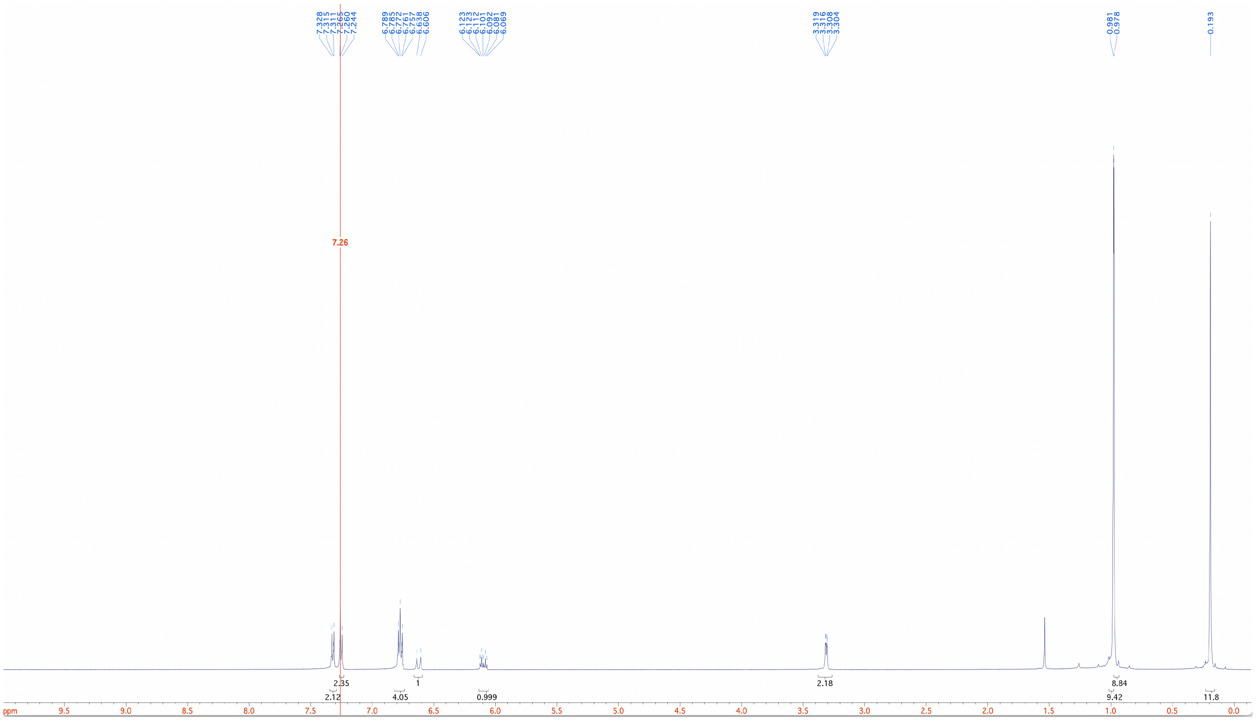
7.26
ppm
9.5
9.0
8.5
8.0
7.5
7.0
6.5
6.0
5.5
5.0
4.5
4.0
3.5
3.0
2.5
2.0
1.5
1.0
0.5
0.0

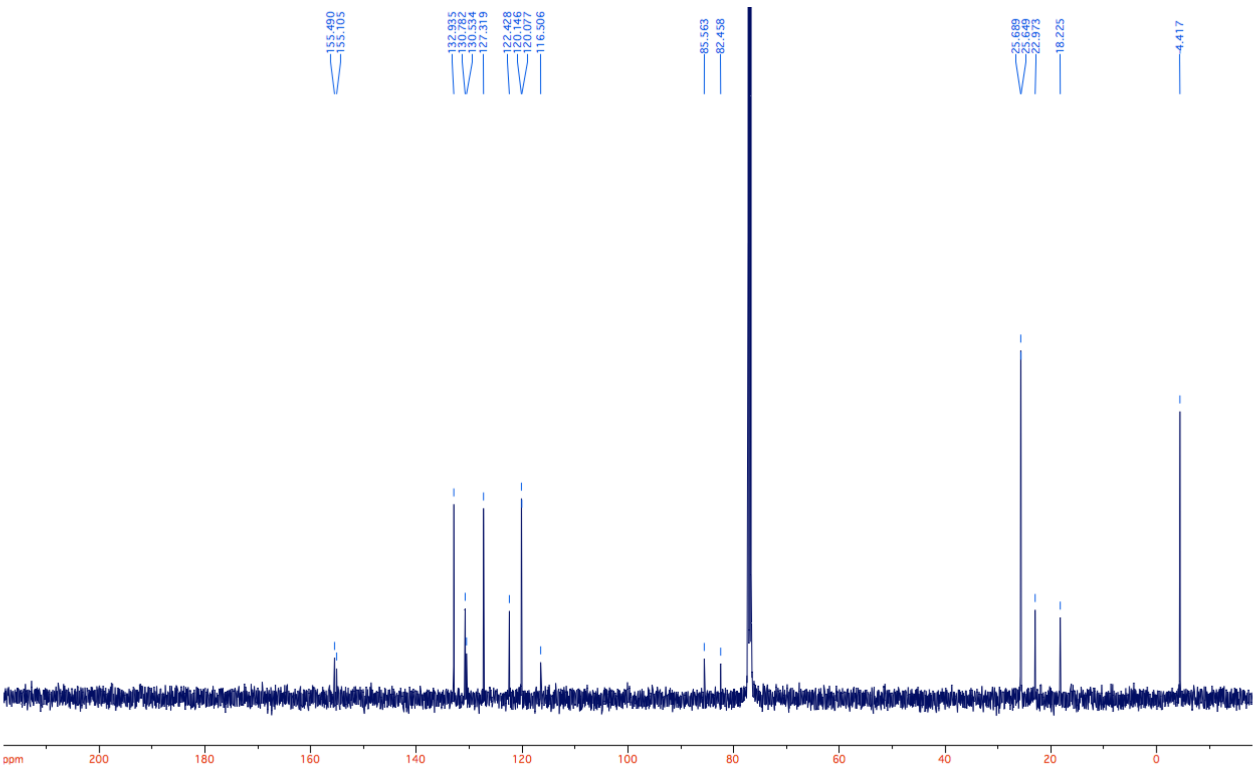
155.490
155.105
132.935
130.782
130.534
127.319
122.428
120.146
120.077
116.506
85.563
82.458
25.689
25.649
22.973
18.225
-4.417
ppm
200
180
160
140
120
100
80
60
40
20
0

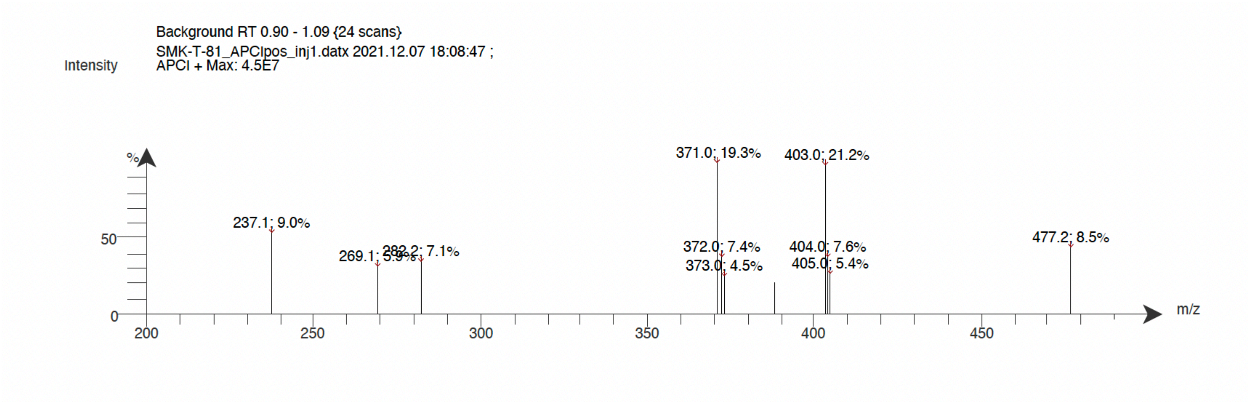

Background RT 0.90 - 1.09 {24 scans}
SMK-T-81_APCIpos_inj1.datx 2021.12.07 18:08:47 ;
Intensity
APCI + Max: 4.5E7
%
50
0
237.1; 9.0%
269.1; 5.9%
282.2; 7.1%
371.0; 19.3%
372.0; 7.4%
373.0; 4.5%
403.0; 21.2%
404.0; 7.6%
405.0; 5.4%
477.2; 8.5%
200
250
300
350
400
450
m/z

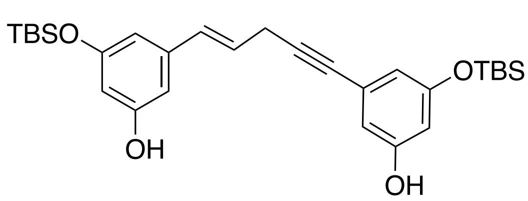

1'CC

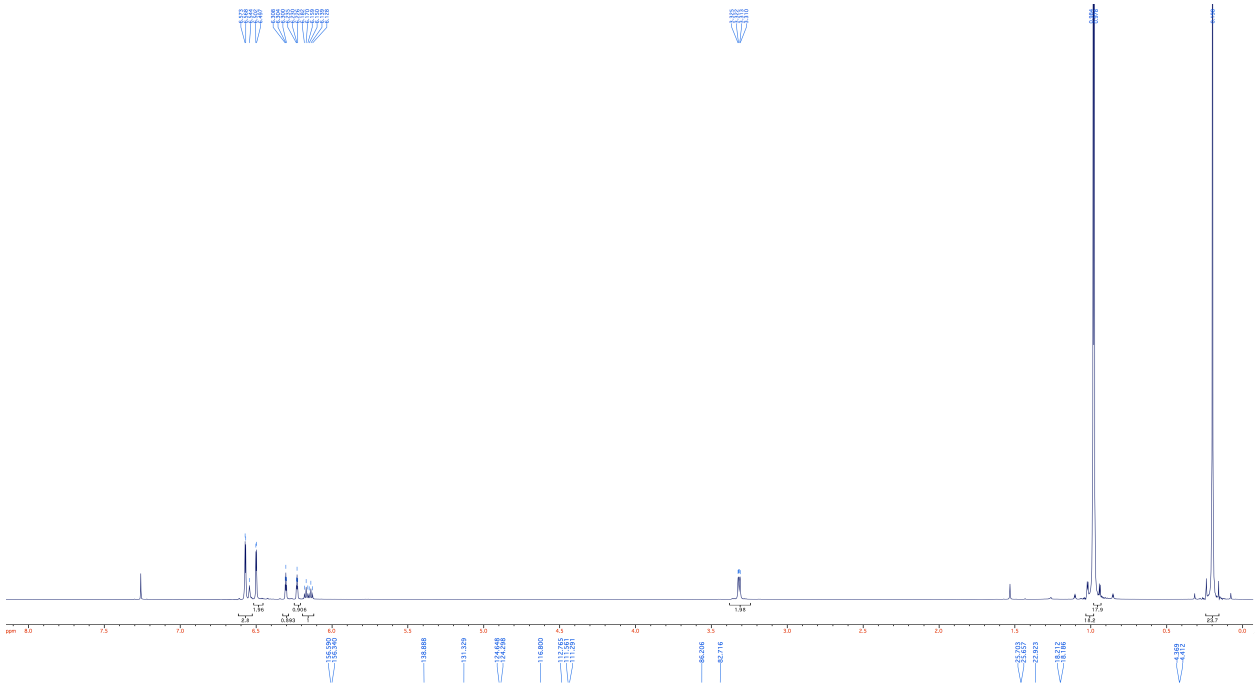

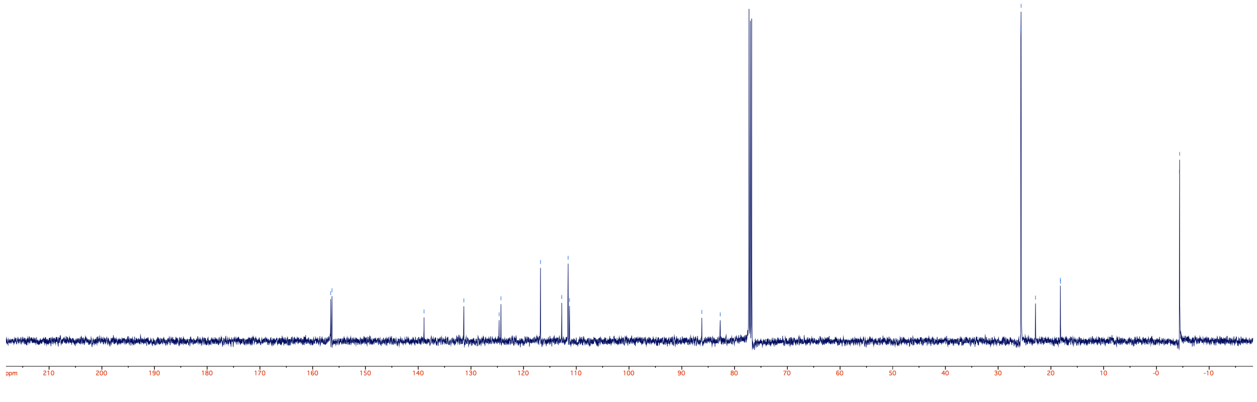

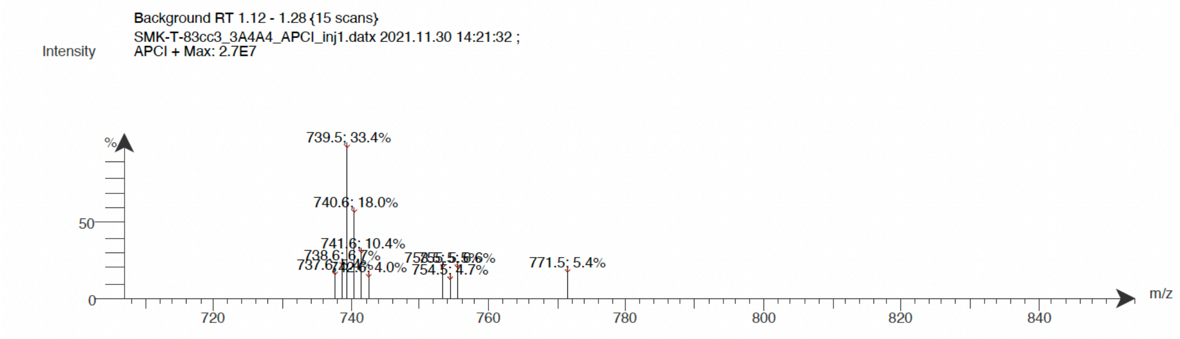

Background RT 1.12 - 1.28 (15 scans)
SMK-T-83cc3_3A4A4_APCI_inj1.datx 2021.11.30 14:21:32 ;
Intensity
APCI + Max: 2.7E7
%
50
0
739.5; 33.4%
740.6; 18.0%
741.6; 10.4%
738.6; 6.7%
742.6; 4.0%
754.5; 4.7%
771.5; 5.4%
720
740
760
780
800
820
840
m/z

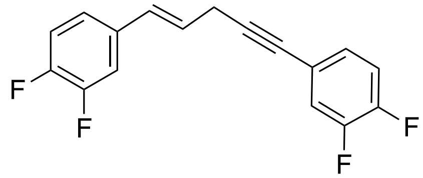
F
F
F
F

1DD

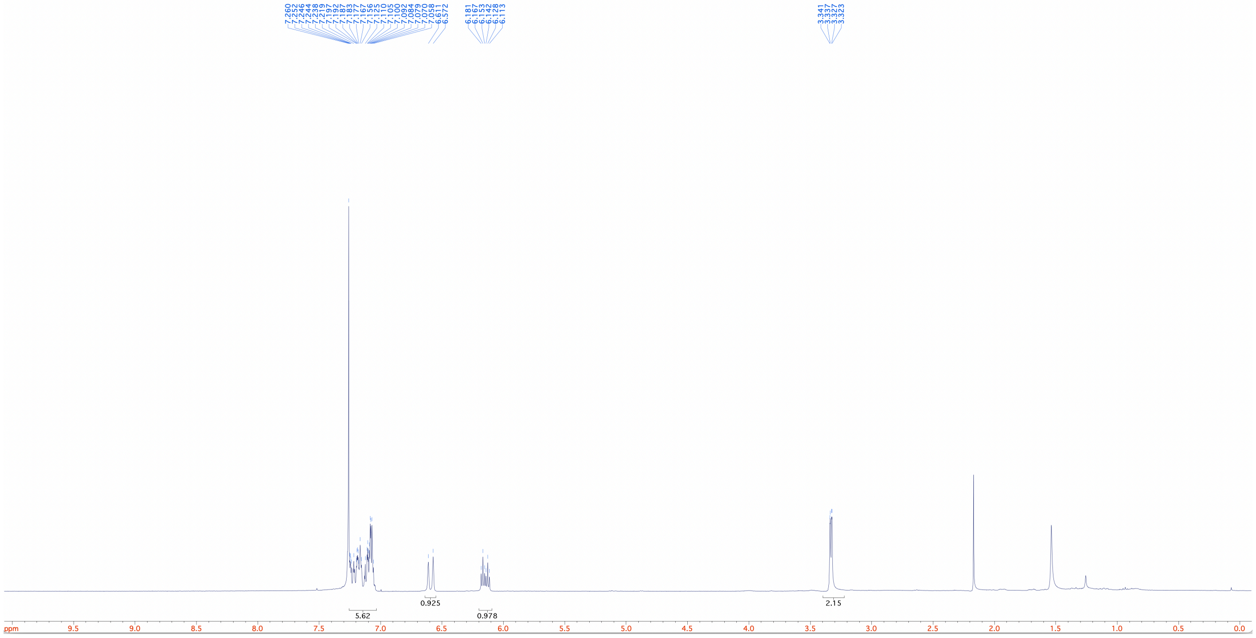
7.260
7.252
7.246
7.244
7.238
7.219
7.197
7.192
7.187
7.183
7.177
7.167
7.156
7.125
7.110
7.105
7.100
7.092
7.084
7.079
7.070
7.058
6.611
6.572
6.181
6.167
6.153
6.142
6.128
6.113
3.341
3.337
3.327
3.323
5.62
0.925
0.978
2.15
ppm
9.5
9.0
8.5
8.0
7.5
7.0
6.5
6.0
5.5
5.0
4.5
4.0
3.5
3.0
2.5
2.0
1.5
1.0
0.5
0.0

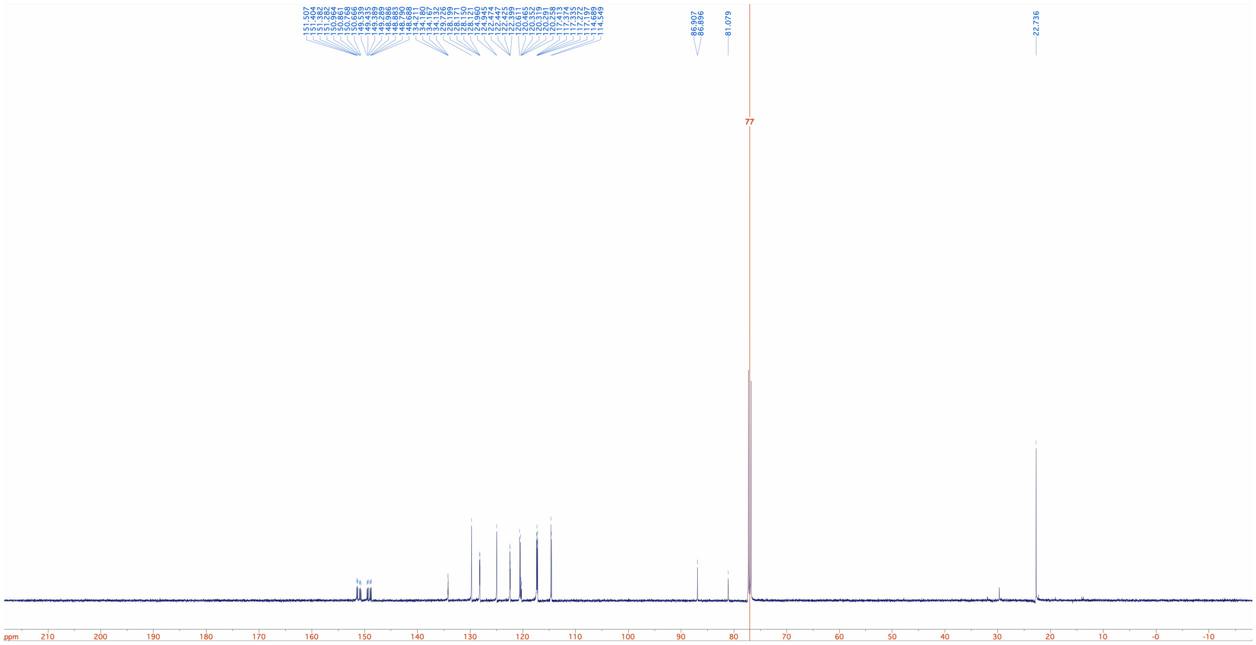
151.507
151.404
151.382
151.282
150.964
150.861
150.768
150.666
149.539
149.435
149.389
149.289
148.986
148.883
148.790
148.688
134.211
134.180
134.167
134.132
129.726
128.199
128.171
128.150
128.121
124.960
124.945
122.474
122.447
122.425
122.399
120.611
120.465
120.352
120.319
120.291
120.258
117.413
117.374
117.335
117.272
117.197
114.689
114.549
86.907
86.896
81.079
77
22.736
ppm
210
200
190
180
170
160
150
140
130
120
110
100
90
80
70
60
50
40
30
20
10
-0
-10

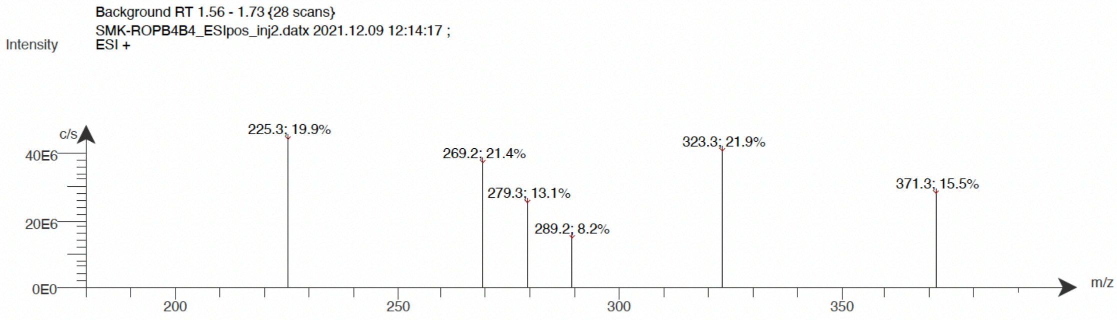
Background RT 1.56 - 1.73 {28 scans}
SMK-ROPB4B4_ESIpos_inj2.datx 2021.12.09 12:14:17 ;
Intensity
ESI +
c/s
40E6
20E6
0E0
225.3; 19.9%
269.2; 21.4%
279.3; 13.1%
289.2; 8.2%
323.3; 21.9%
371.3; 15.5%
200
250
300
350
m/z

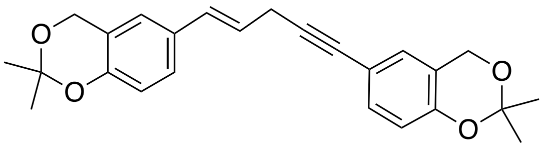

1FF

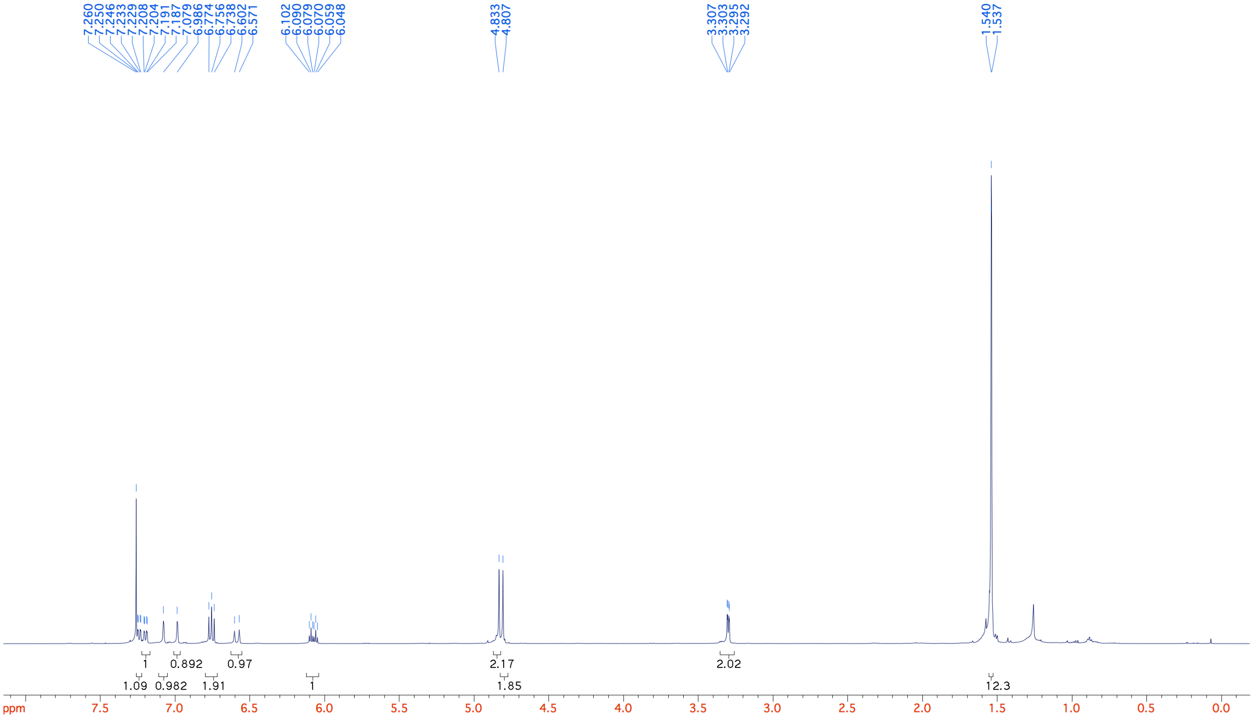

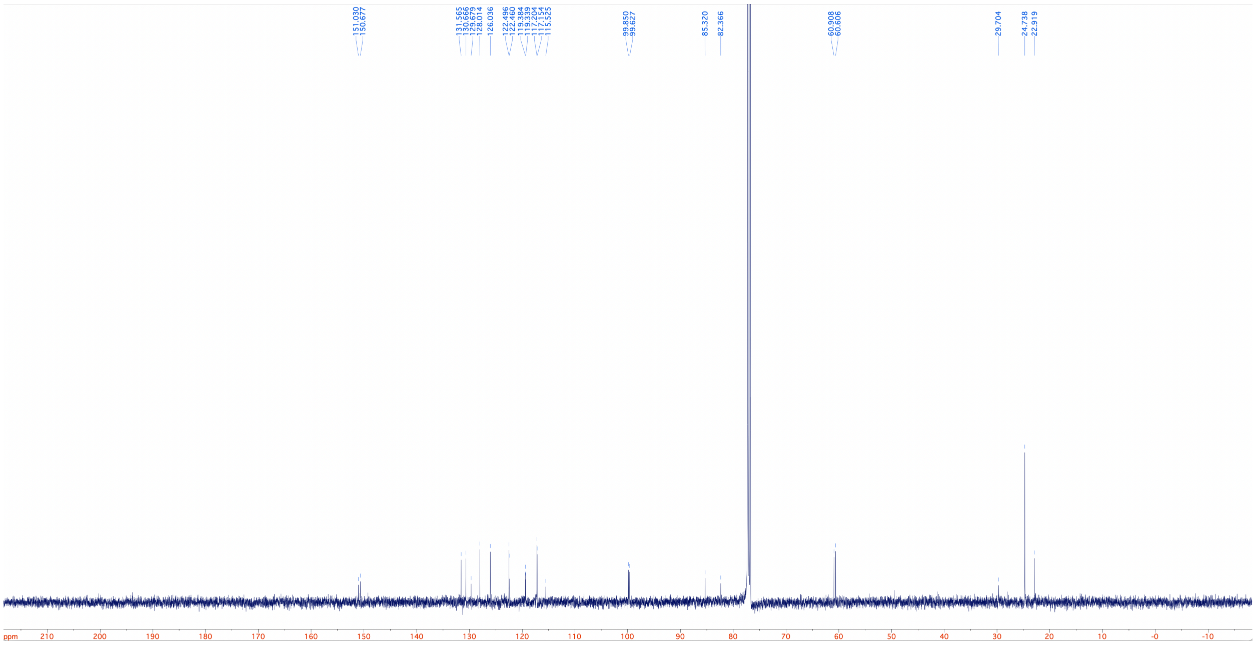

Background RT 0.93 - 1.16 {29 scans}
SMK-T-93_3C3C3_APCI_inj3.datx 2021.11.30 19:06:18 ;
Intensity APCI + Max: 2.5E7

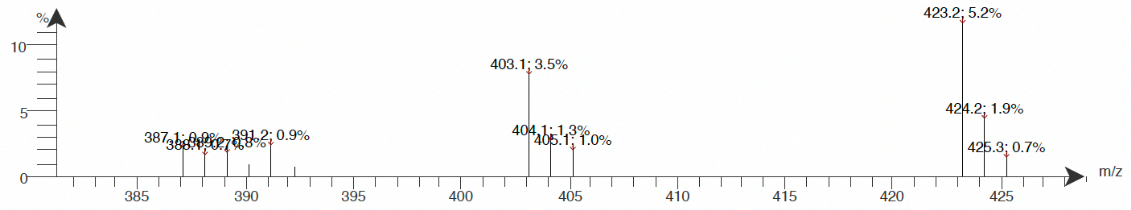

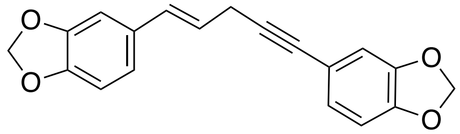

1EE

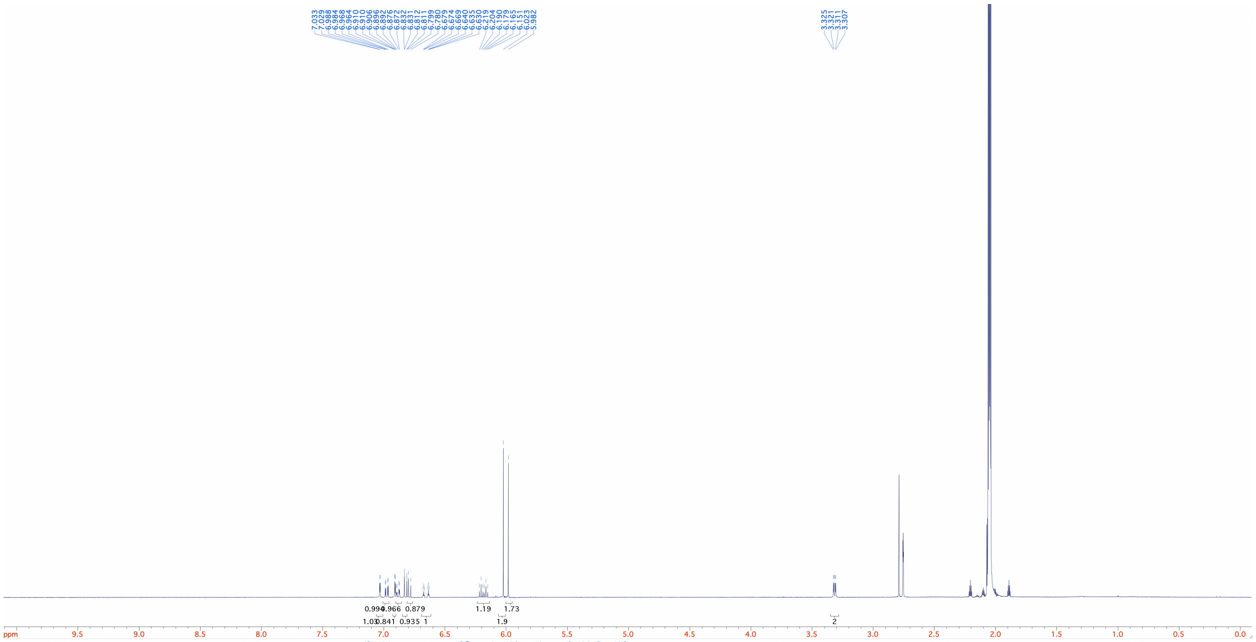

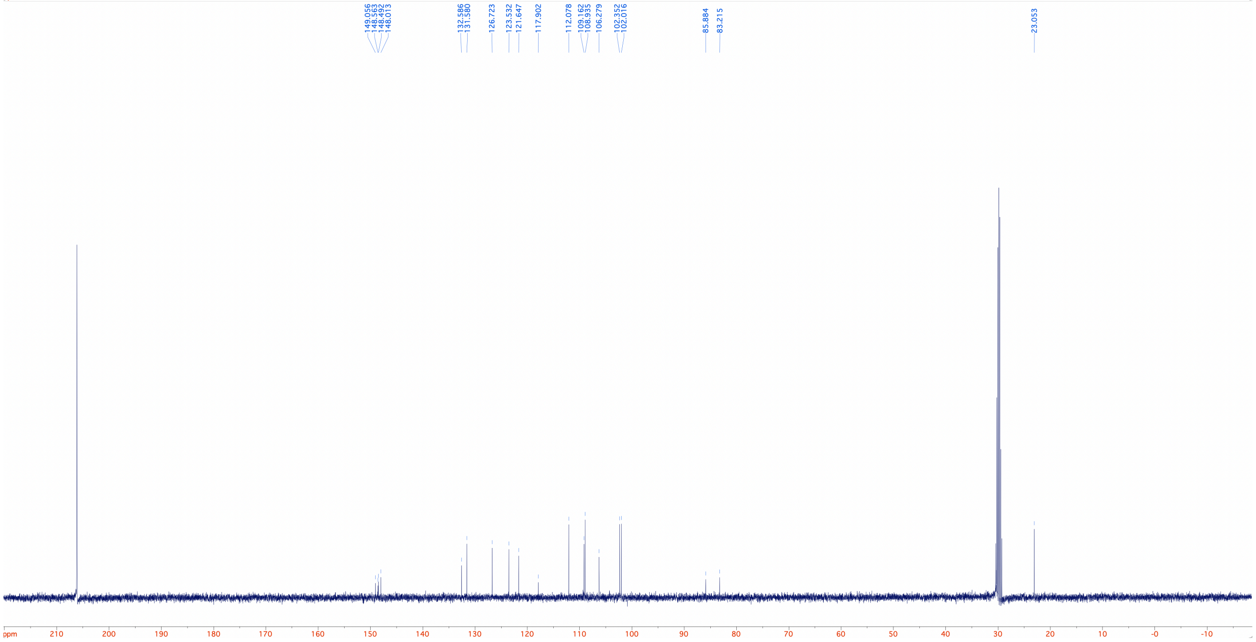

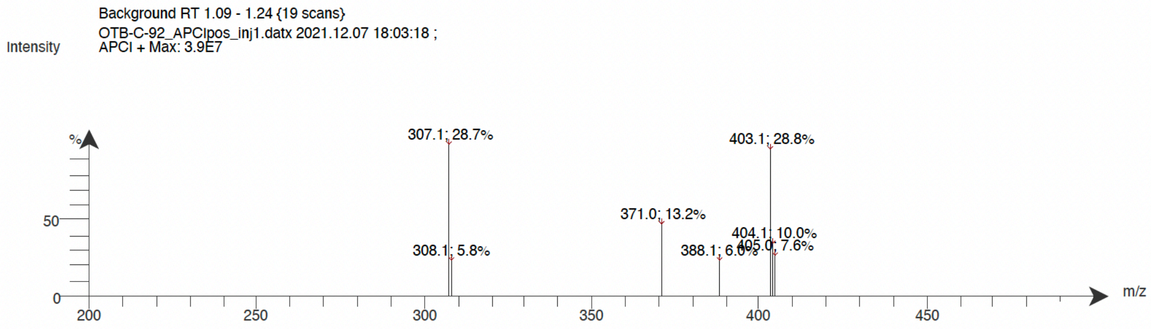
Background RT 1.09 - 1.24 (19 scans)
OTB-C-92_APCIpos_inj1.datx 2021.12.07 18:03:18 ;
Intensity
APCI + Max: 3.9E7
307.1; 28.7%
308.1; 5.8%
371.0; 13.2%
388.1; 6.0%
403.1; 28.8%
404.1; 10.0%
405.0; 7.6%
m/z

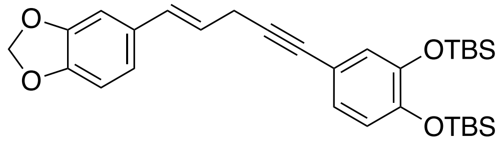

1’EA

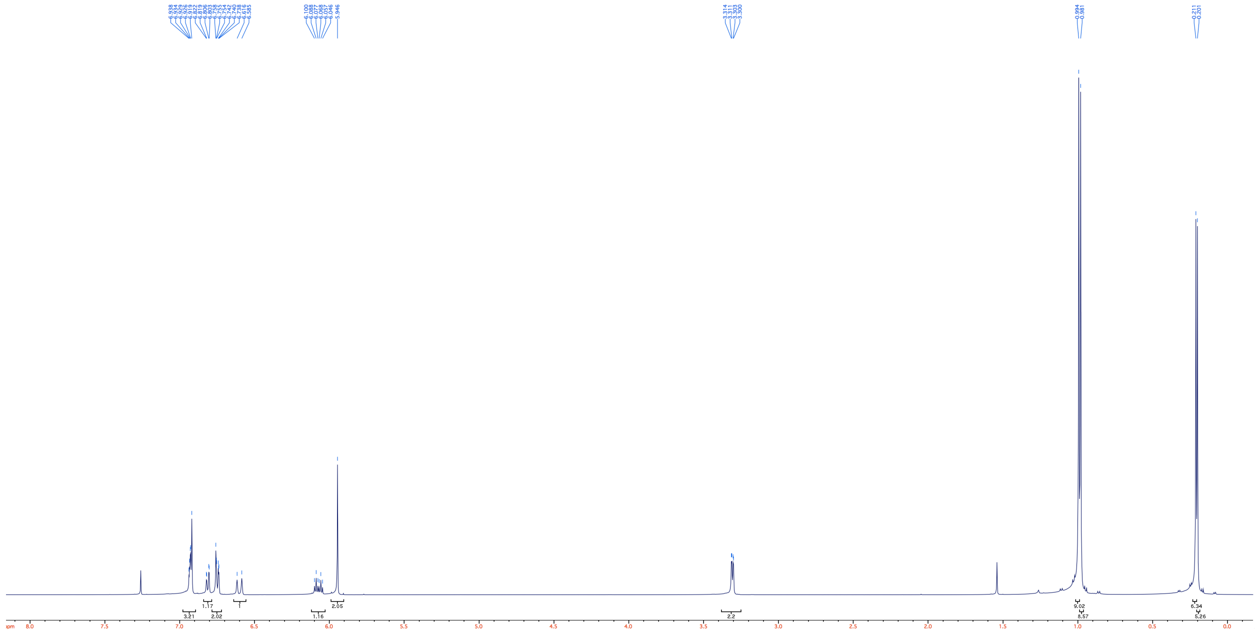

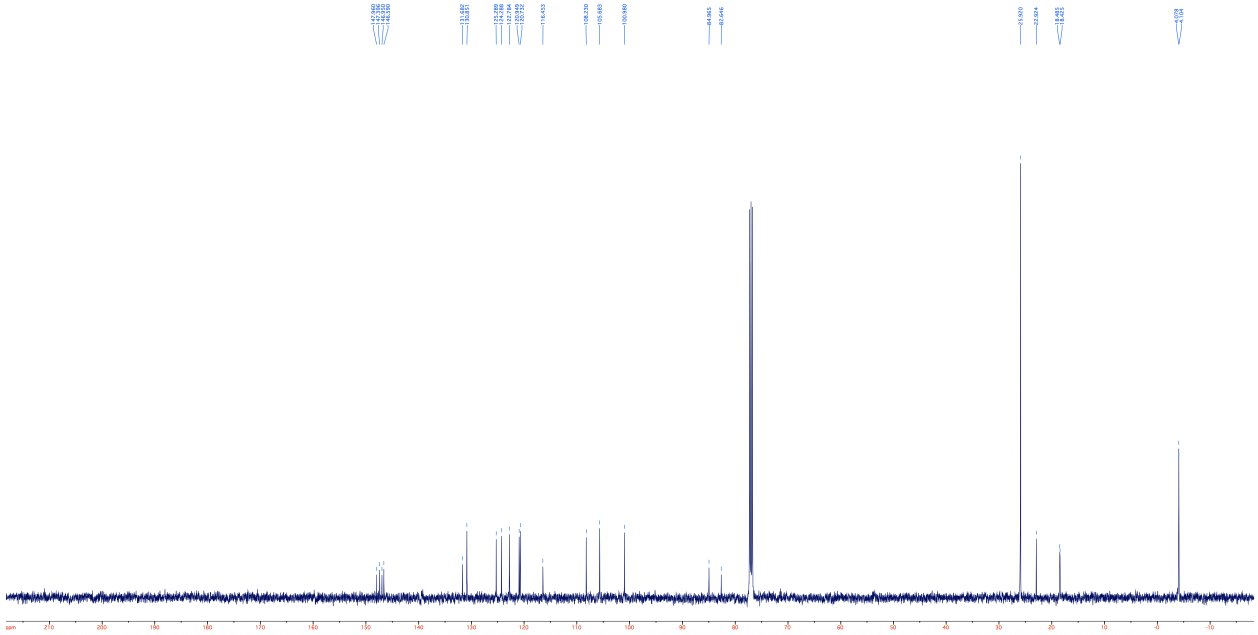

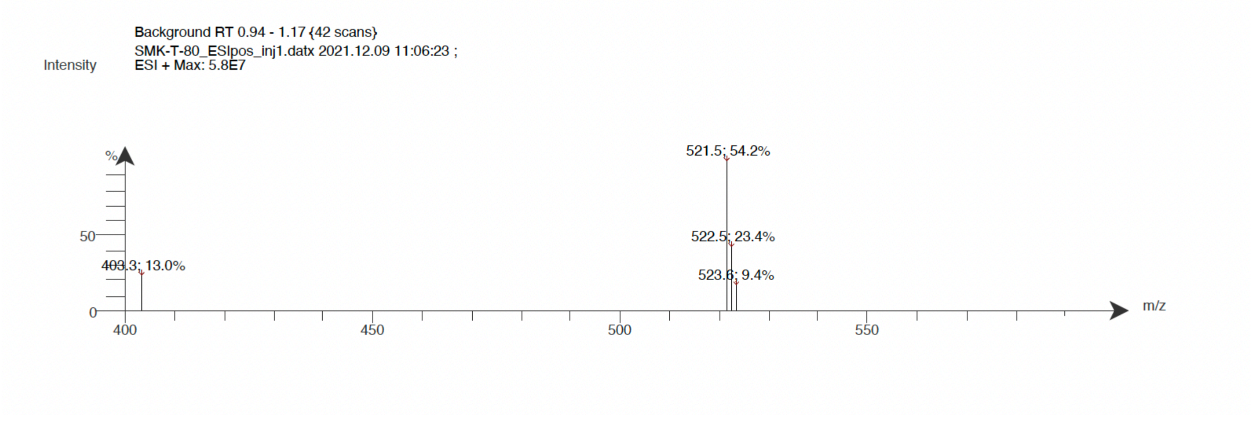
Background RT 0.94 - 1.17 {42 scans}
SMK-T-80_ESIpos_inj1.datx 2021.12.09 11:06:23 ;
ESI + Max: 5.8E7
Intensity
521.5; 54.2%
522.5; 23.4%
523.6; 9.4%
403.3; 13.0%
m/z

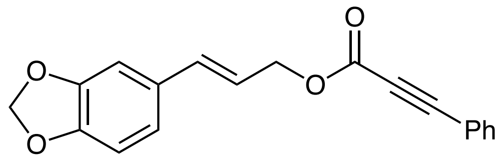

1EG

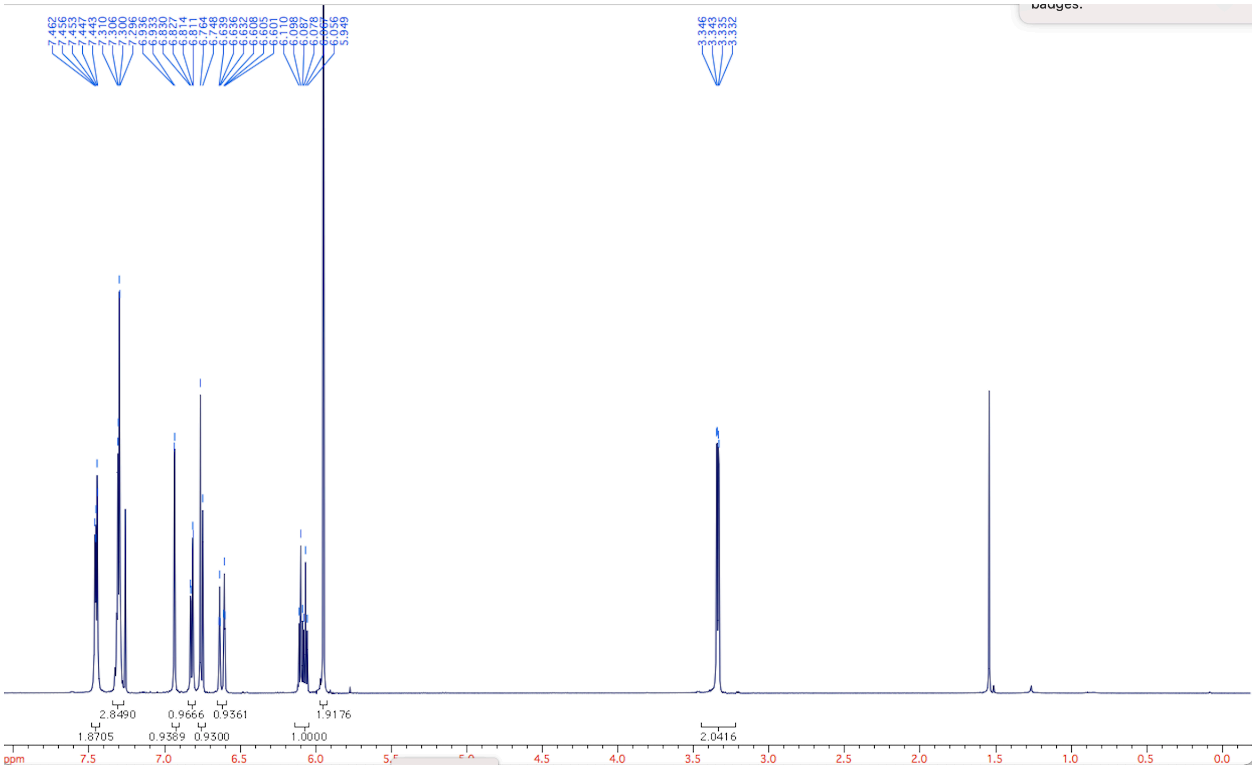

7.462
7.456
7.453
7.447
7.443
7.310
7.306
7.300
7.296
6.936
6.933
6.830
6.827
6.814
6.811
6.764
6.748
6.639
6.636
6.632
6.608
6.605
6.601
6.110
6.098
6.087
6.078
6.056
5.949
3.346
3.343
3.335
3.332
2.8490
0.9666
0.9361
1.9176
1.8705
0.9389
0.9300
1.0000
2.0416
ppm

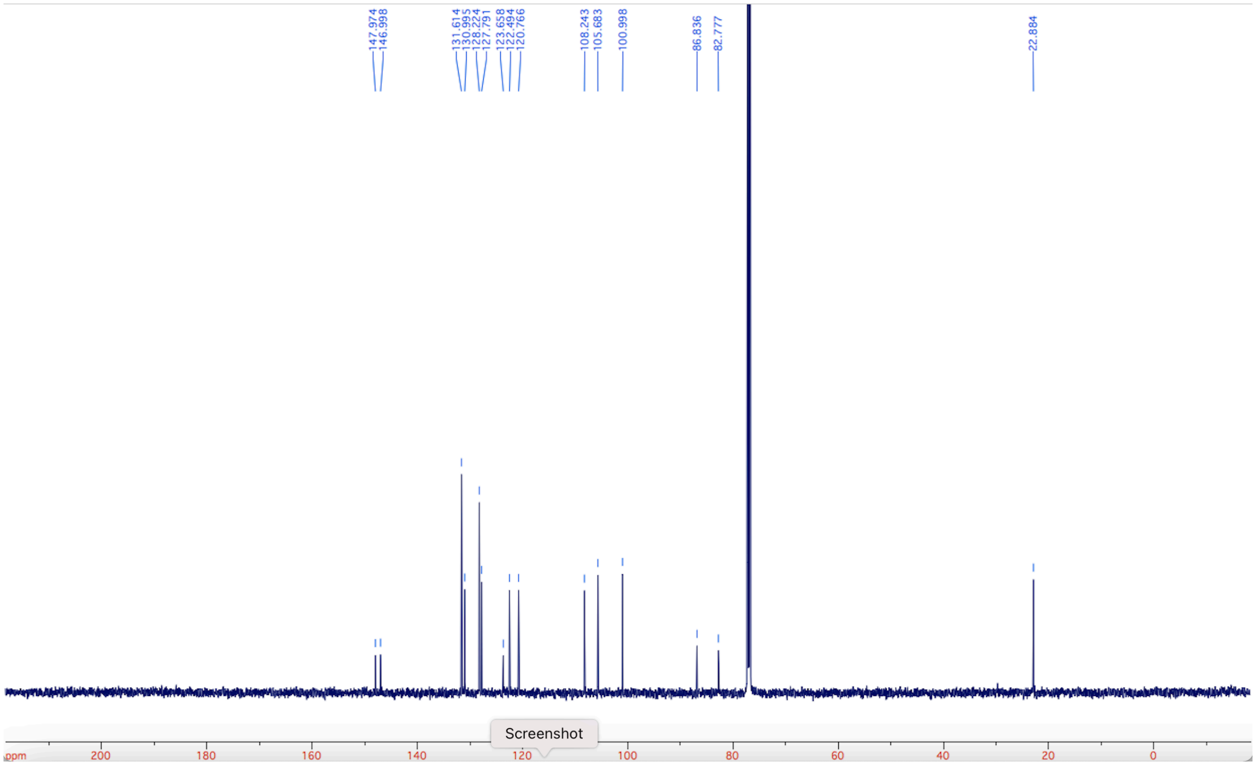

147.974
146.998
131.614
130.995
128.224
127.791
123.658
122.494
120.766
108.243
105.683
100.998
86.836
82.777
22.884
ppm

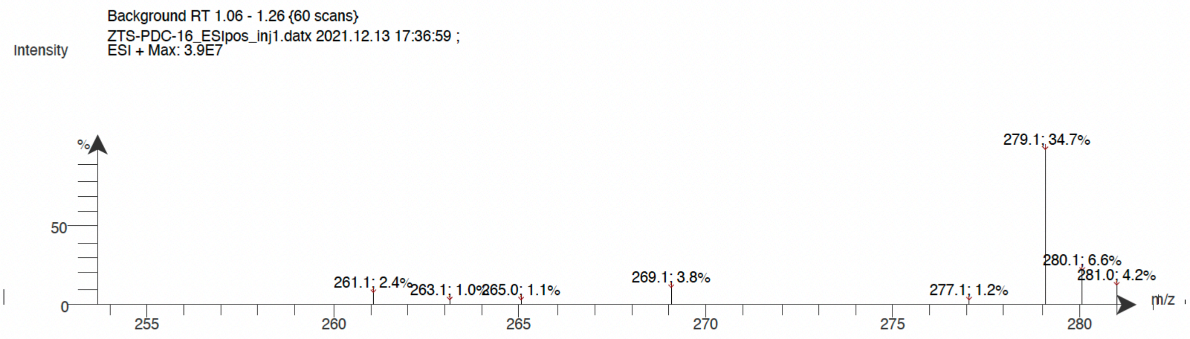
Background RT 1.06 - 1.26 {60 scans}
ZTS-PDC-16_ESIpos_inj1.datx 2021.12.13 17:36:59 ;
ESI + Max: 3.9E7
Intensity
%
50
0
279.1; 34.7%
280.1; 6.6%
281.0; 4.2%
261.1; 2.4%
263.1; 1.0%
265.0; 1.1%
269.1; 3.8%
277.1; 1.2%
255
260
265
270
275
280
m/z

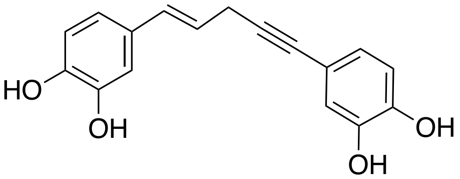

rooperol

1AA

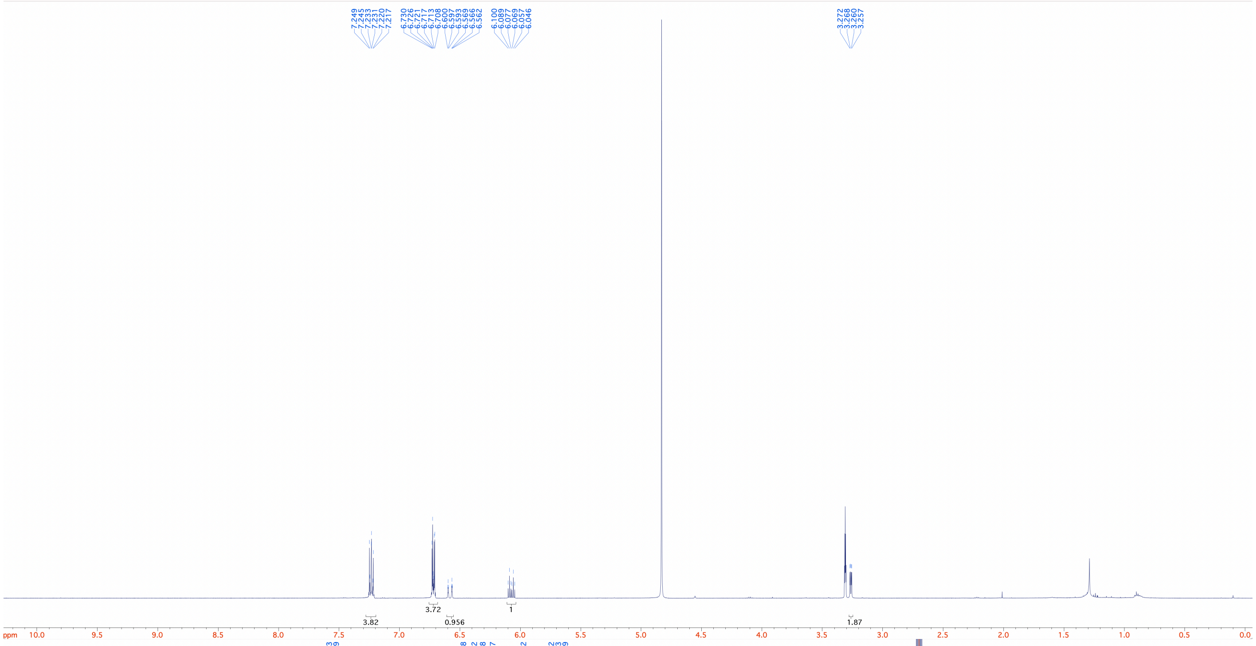

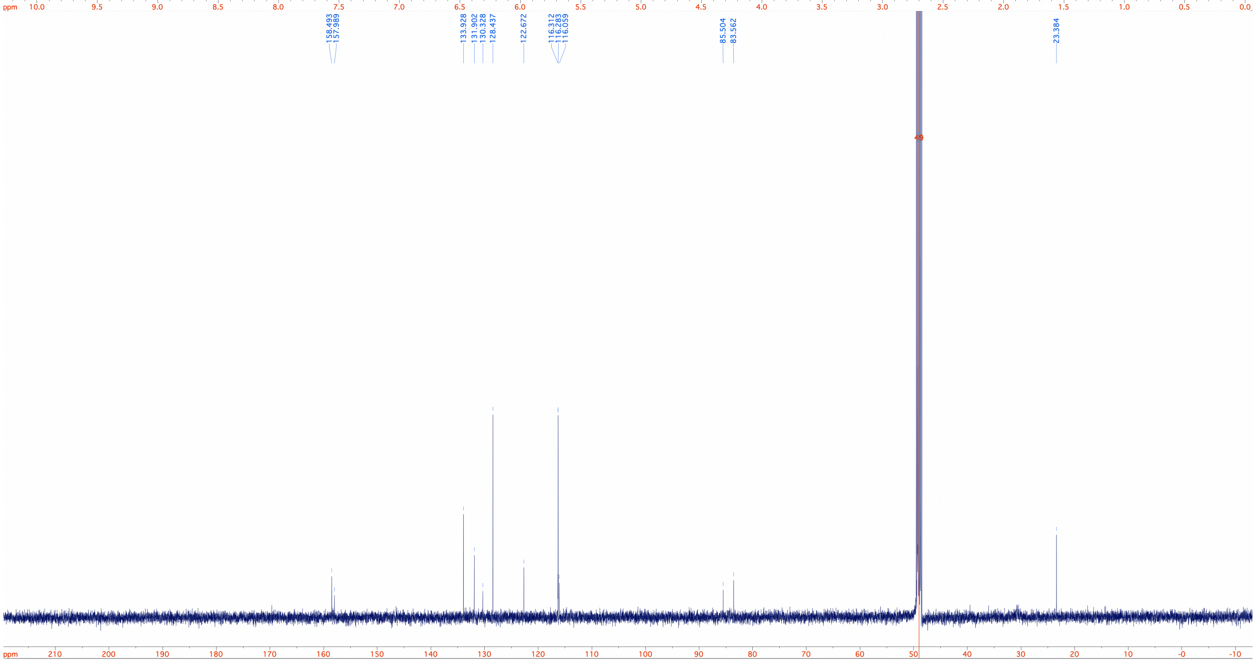

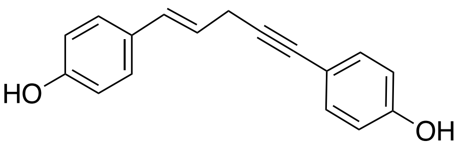
HO
OH

1BB

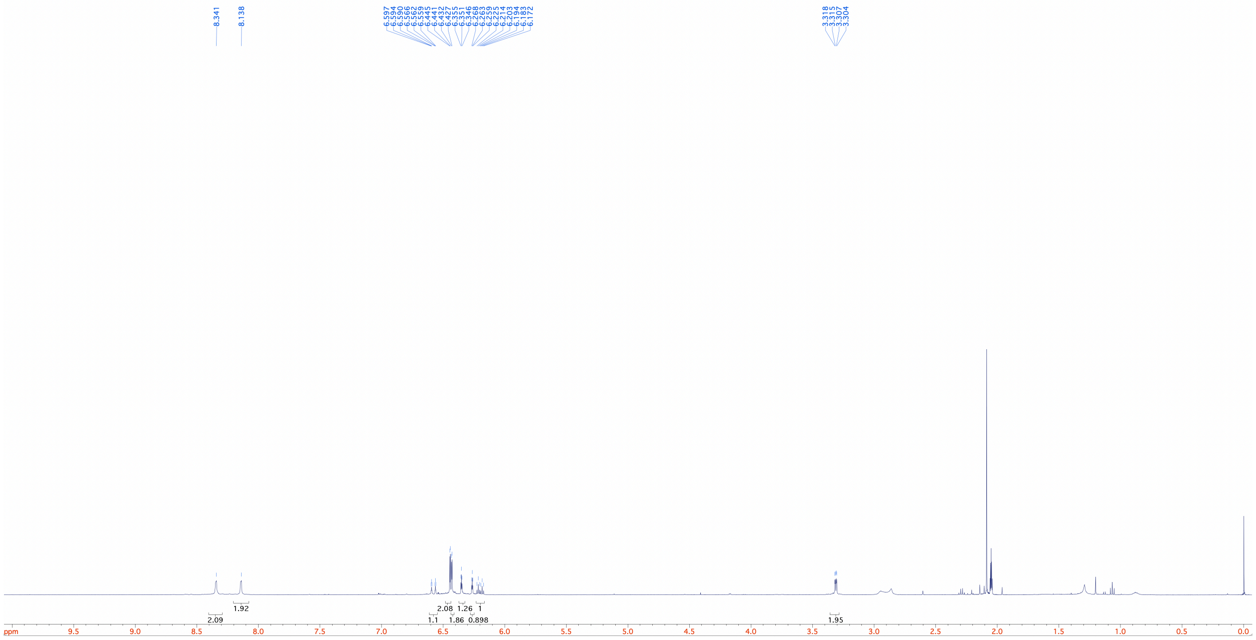
8.341
8.138
3.318
3.315
3.311
3.307
3.304
2.09
1.92
1.1
2.08
1.86
1.26
0.898
1
1.95
ppm

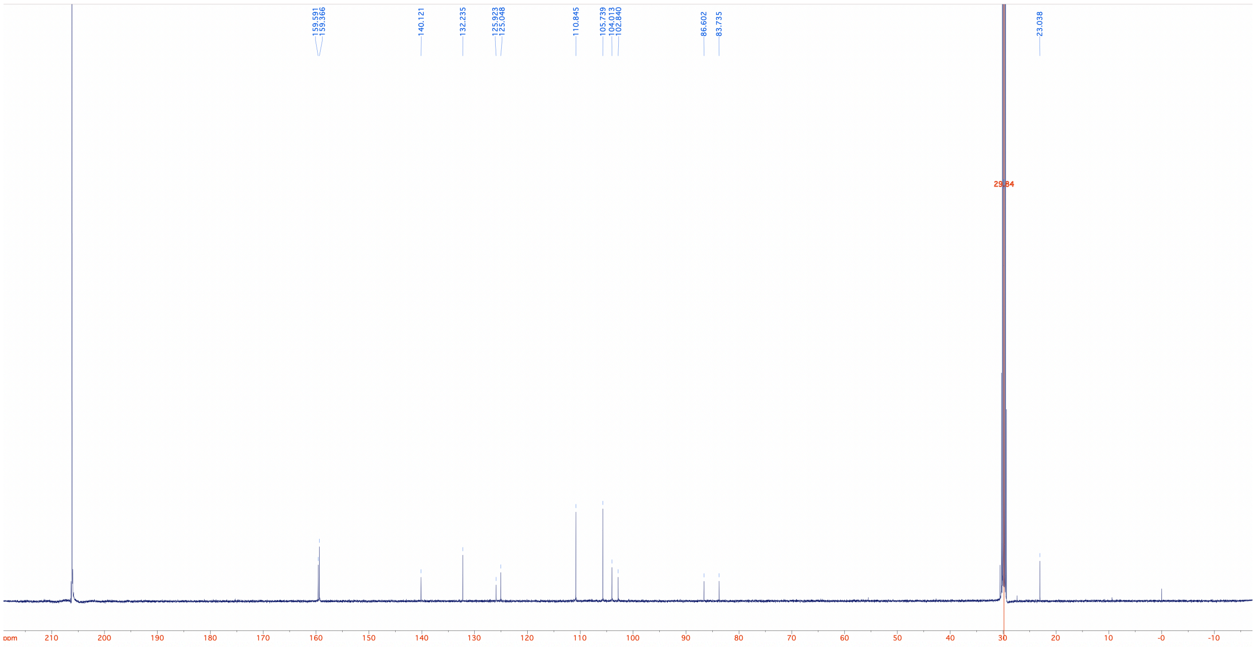
159.591
159.366
140.121
132.235
125.923
125.048
110.845
105.739
104.013
102.840
86.602
83.735
23.038
ppm

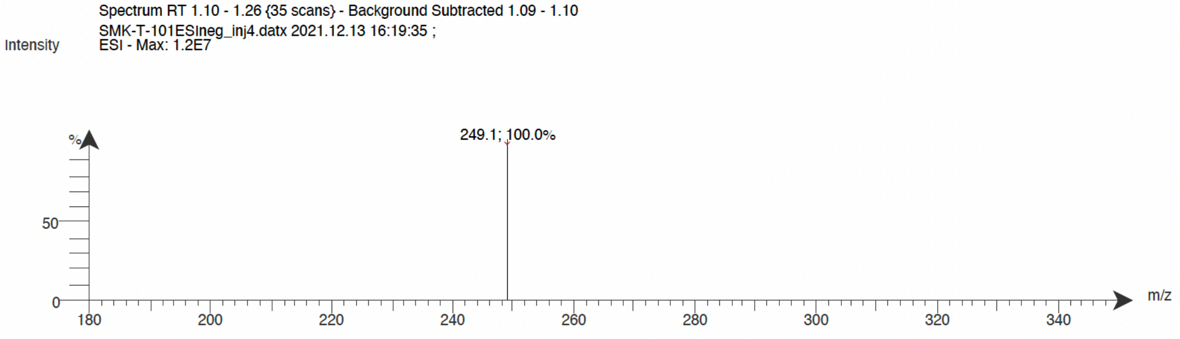
Spectrum RT 1.10 - 1.26 (35 scans) - Background Subtracted 1.09 - 1.10
SMK-T-101ESIneg_inj4.datx 2021.12.13 16:19:35 ;
ESI - Max: 1.2E7
Intensity
249.1; 100.0%
%
m/z

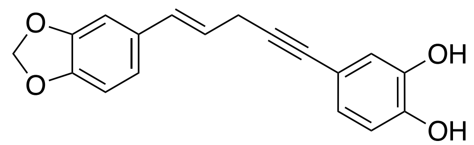

O
O
OH
OH

1EA

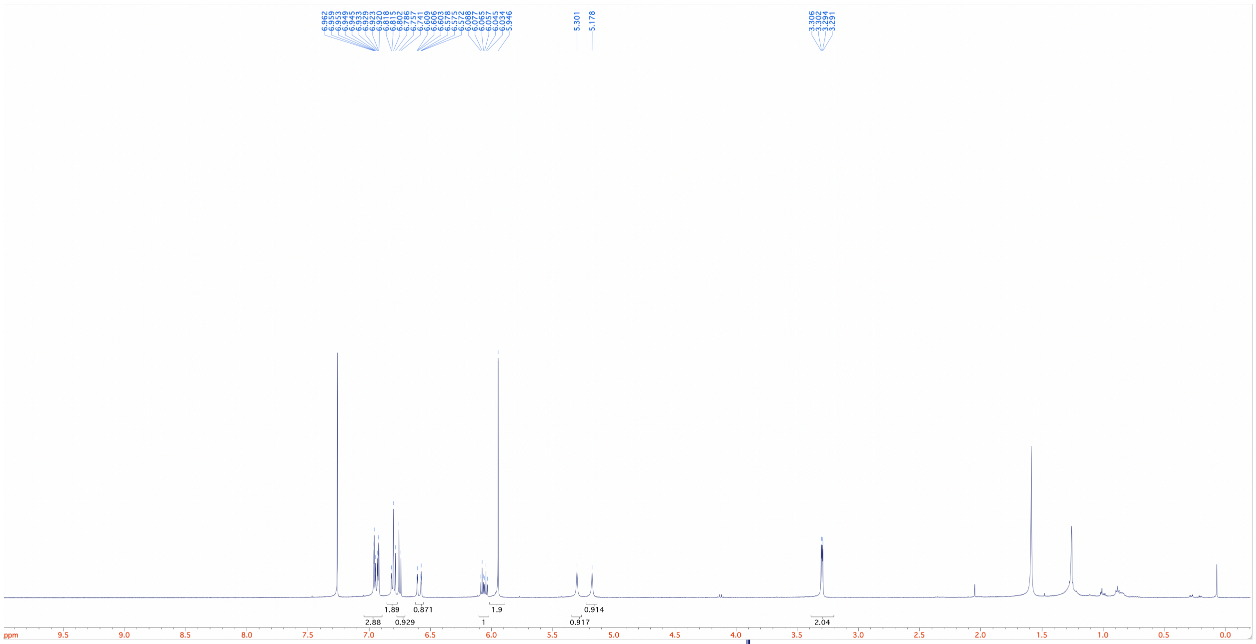

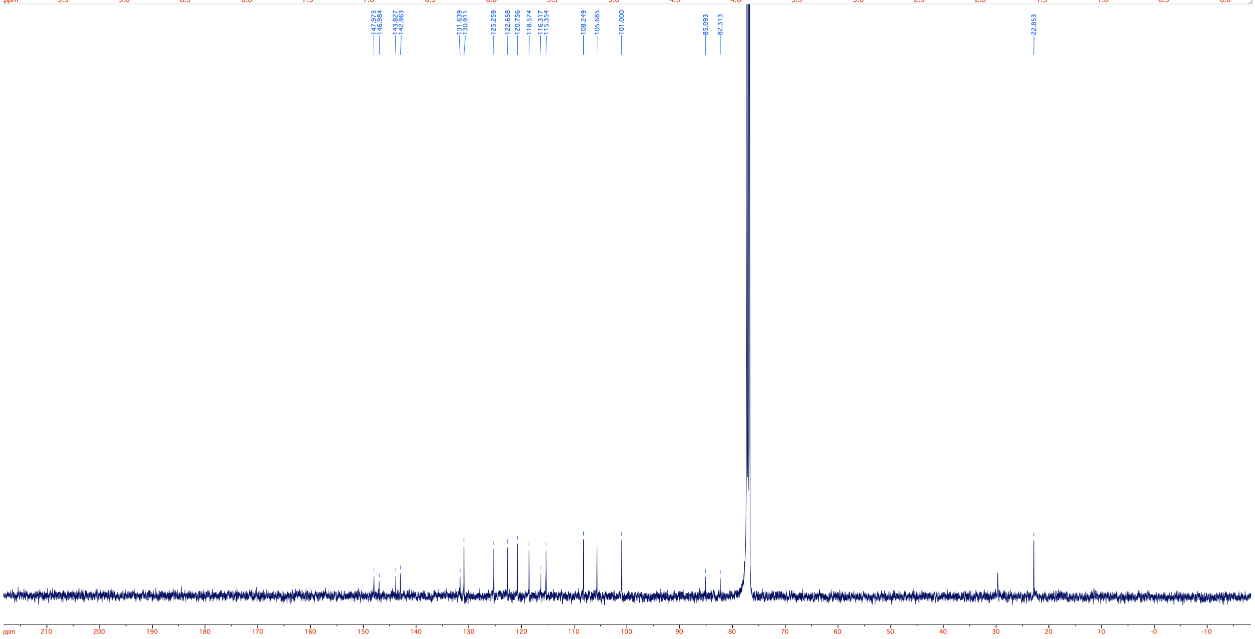

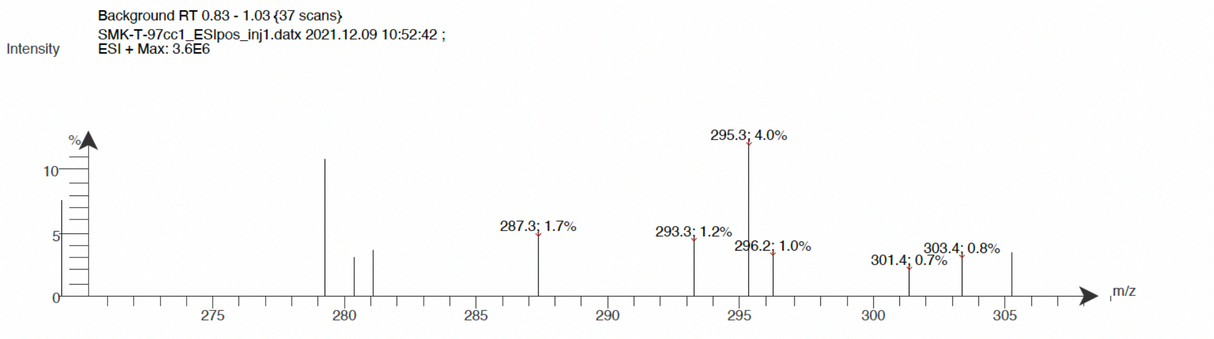

Background RT 0.83 - 1.03 {37 scans}
SMK-T-97cc1_ESIpos_inj1.datx 2021.12.09 10:52:42 ;
Intensity
ESI + Max: 3.6E6
%
295.3; 4.0%
287.3; 1.7%
293.3; 1.2%
296.2; 1.0%
301.4; 0.7%
303.4; 0.8%
m/z

Supplemental Figure 1. Dose-dependent cytotoxicity of rooperol and analogs in cancer cell lines. Cells were treated with a serial of dilutions of each compound in three replicates in 96-well plates for 96 h, and cell viability was measured using MTT assay. Shown in each figure is dose-dependent response of the indicated cells to the indicated compound, with the dotted line signifying the IC_{50}.

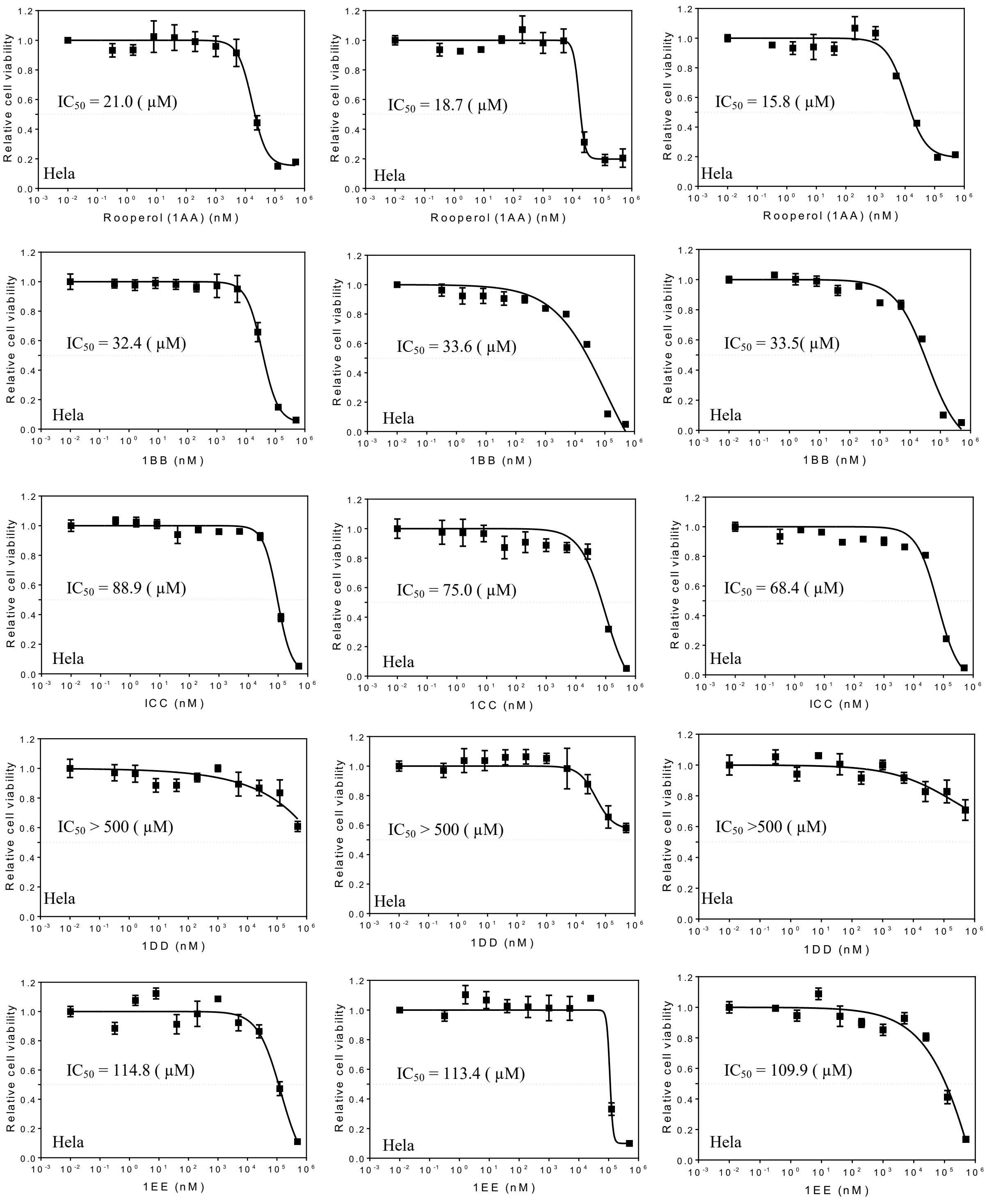

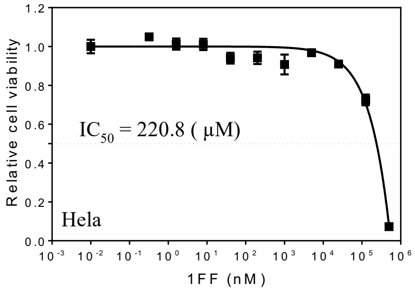
Relative cell viability
IC_{50} = 220.8 (μM)
Hela
1FF (nM)

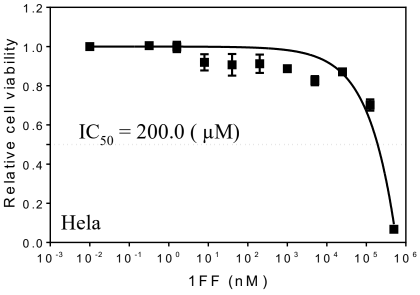
Relative cell viability
IC_{50} = 200.0 (μM)
Hela
1FF (nM)

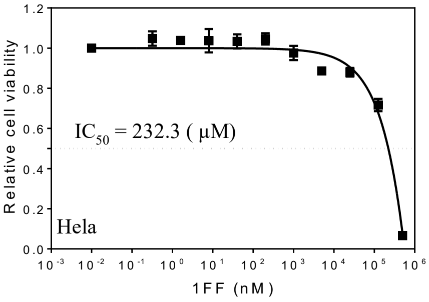
Relative cell viability
IC_{50} = 232.3 (μM)
Hela
1FF (nM)

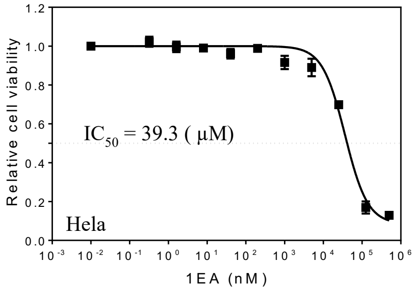
Relative cell viability
IC_{50} = 39.3 (μM)
Hela
1EA (nM)

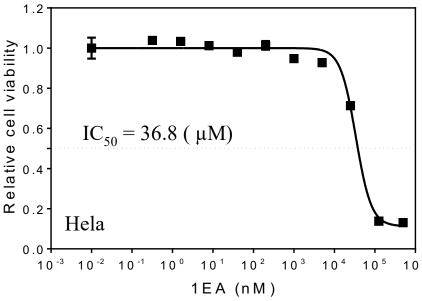
Relative cell viability
IC_{50} = 36.8 (μM)
Hela
1EA (nM)

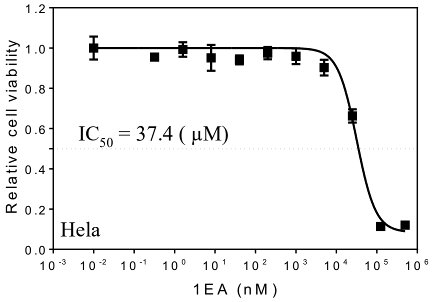
Relative cell viability
IC_{50} = 37.4 (μM)
Hela
1EA (nM)

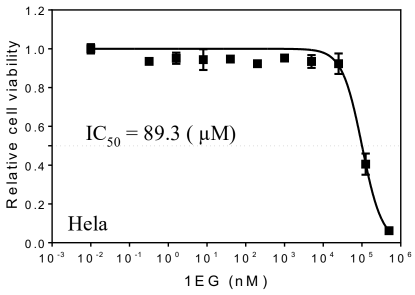
Relative cell viability
IC_{50} = 89.3 (μM)
Hela
1EG (nM)

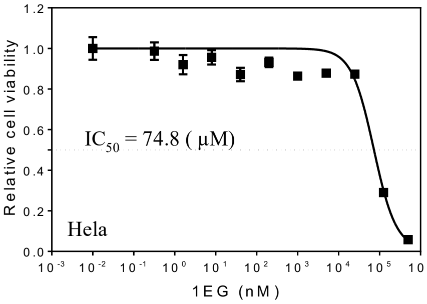
Relative cell viability
IC_{50} = 74.8 (μM)
Hela
1EG (nM)

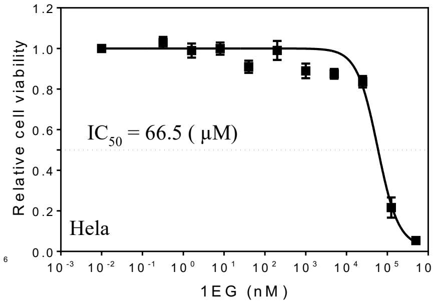
Relative cell viability
IC_{50} = 66.5 (μM)
Hela
1EG (nM)

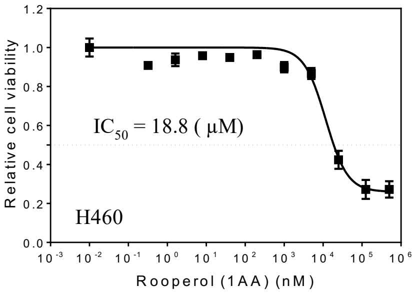
Relative cell viability
IC_{50} = 18.8 (μM)
H460
Rooperol (1AA) (nM)

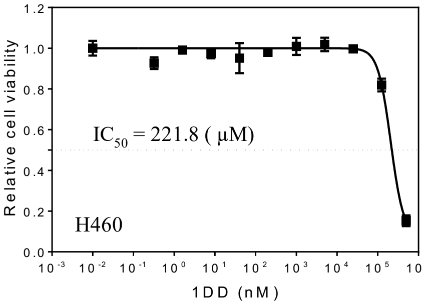
Relative cell viability
IC_{50} = 221.8 (μM)
H460
1DD (nM)

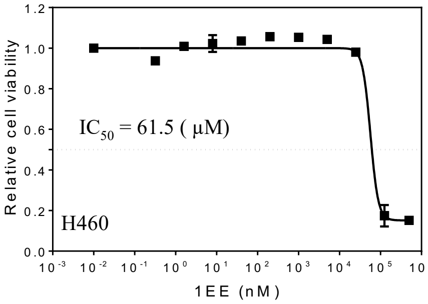
Relative cell viability
IC_{50} = 61.5 (μM)
H460
1EE (nM)

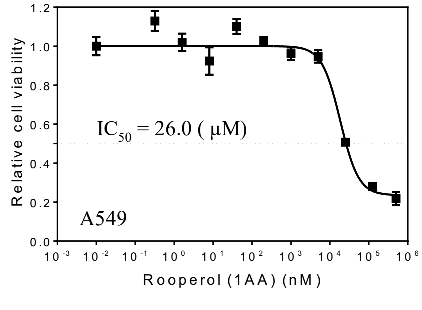
Relative cell viability
IC_{50} = 26.0 (μM)
A549
Rooperol (1AA) (nM)

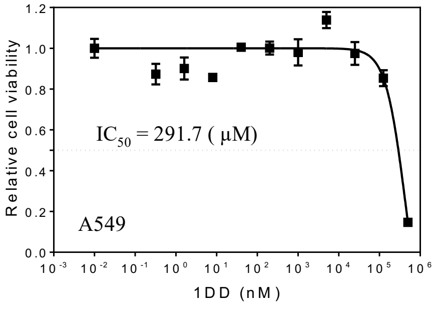
Relative cell viability
IC_{50} = 291.7 (μM)
A549
1DD (nM)

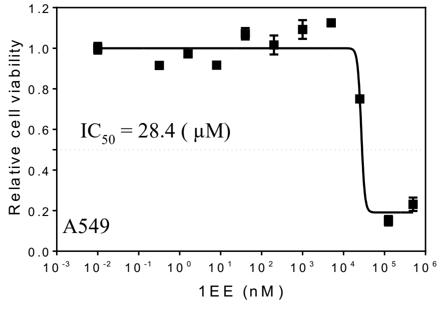
Relative cell viability
IC_{50} = 28.4 (μM)
A549
1EE (nM)
